# High Power Irradiance Dependence of Charge Species Dynamics in Hybrid Perovskites and Kinetic Evidence for Transient Vibrational Stark Effect in Formamidinium

**DOI:** 10.3390/nano12101616

**Published:** 2022-05-10

**Authors:** Rafal Rakowski, William Fisher, Joaquín Calbo, Muhamad Z. Mokhtar, Xinxing Liang, Dong Ding, Jarvist M. Frost, Saif A. Haque, Aron Walsh, Piers R. F. Barnes, Jenny Nelson, Jasper J. van Thor

**Affiliations:** 1 Life Science Department, Imperial College London, London SW7 2AZ, UK; rafal1rakowski@gmail.com; 2 Department of Physics, Imperial College London, London SW7 2AZ, UK; w.fisher17@imperial.ac.uk (W.F.); jarvist.frost@imperial.ac.uk (J.M.F.); piers.barnes@imperial.ac.uk (P.R.F.B.); jenny.nelson@imperial.ac.uk (J.N.); 3 Department of Materials, Imperial College London, London SW7 2AZ, UK; joaquin.calbo@uv.es (J.C.); a.walsh@imperial.ac.uk (A.W.); 4 School of Materials, University of Manchester, MSS Tower, Manchester M13 9PL, UK; engr.hasif@yahoo.com; 5 Department of Chemistry, Centre for Plastic Electronics, Imperial College London, London W12 0BZ, UK; xinxing.liang@imperial.ac.uk (X.L.); dong.ding11@imperial.ac.uk (D.D.); s.a.haque@imperial.ac.uk (S.A.H.)

**Keywords:** hybrid perovskite, Vibrational Stark effect, formamidinium, charge species dynamics, mid-infrared absorption spectroscopy

## Abstract

Hybrid halide perovskites materials have the potential for both photovoltaic and light-emitting devices. Relatively little has been reported on the kinetics of charge relaxation upon intense excitation. In order to evaluate the illumination power density dependence on the charge recombination mechanism, we have applied a femtosecond transient mid-IR absorption spectroscopy with strong excitation to directly measure the charge kinetics via electron absorption. The irradiance-dependent relaxation processes of the excited, photo-generated charge pairs were quantified in polycrystalline MAPbI_3_, MAPbBr_3_, and (FAPbI_3_)_0.97_(MAPbBr_3_)_0.03_ thin films that contain either methylamonium (MA) or formamidinium (FA). This report identifies the laser-generated charge species and provides the kinetics of Auger, bimolecular and excitonic decay components. The inter-band electron-hole (bimolecular) recombination was found to dominate over Auger recombination at very high pump irradiances, up to the damage threshold. The kinetic analysis further provides direct evidence for the carrier field origin of the vibrational Stark effect in a formamidinium containing perovskite material. The results suggest that radiative excitonic and bimolecular recombination in MAPbI_3_ at high excitation densities could support light-emitting applications.

## 1. Introduction

In recent years, hybrid organic-inorganic perovskites have led to the rapid development of solar cell technology with power conversion efficiencies exceeding 25% in 2020 [1]. The unique features of perovskites, namely, strong UV-VIS absorption, high yield of photo-excited free charge carriers, high carrier mobility, and low production cost make them very attractive materials for device applications. Their excellent optoelectronic properties predispose hybrid perovskites to implementations in photovoltaics, electronics [2], light-emitting devices with their broad tunability [2,3,4,5], including lasing [6,7,8], and in detection as well [9,10,11,12,13].

The crucial step in all of these applications is the creation of a significant charge carrier concentration band. Here, we investigated the kinetic properties of recombination pathways in selected hybrid perovskites using transient absorption (TA) in the mid-infrared spectral region with intense femtosecond visible excitation. The mid-IR measurements measure both free (electron and hole) and bound carriers (excitonic or polaronic states) dynamics as well as molecular vibrations [14]. Whilst the free charges play a vital role in photovoltaic materials, the bound charges are crucial in light-emitting devices and in the loss processes that could affect the photovoltaic performance. Other commonly used and complementary techniques are time-resolved photoluminescence spectroscopy (which does not resolve non-radiative Auger decay), and methods that measure only free carriers: time-resolved terahertz absorption spectroscopy and time-resolved microwave conductivity (e.g., suitable for a carrier mobility measurements). Establishing the actual time dependence and nature of physical relaxation processes helps to determine the suitability of a material for light-emitting applications. In addition, the kinetic analysis of the transient absorption arising from infrared active molecular vibrations provides insight into the structural dynamics of the material and the molecular response to the electronic dynamics.

Recent research has focused on the control of material characteristics of perovskites [15,16]. Recent experiments proposed that self-localized excitons are formed by free excitons, which are bound to phonons [17,18]. The exciton binding energy in hybrid perovskites was reported to be independent of temperature (hence excitation level), which allows the photogenerated excitons to be treated the same across a broad excitation energy range for a given material [17]. Photoluminescence studies of the trapped states showed that the states mainly recombine radiatively [18]. In MAPbI_3_ the onset of amplified spontaneous emission has been observed at a carrier density of 2 × 10^18^ cm^−3^ [7]. This indicates that the excitonic character of lasing of the perovskite is due to the high concentration of excitons at this carrier density. Using transient mid-IR absorption spectroscopy, the C=N stretching mode of formamidinium in addition to the continuum absorption of the optically injected carriers has been studied previously [14]. The observed spectral shift of the mode was proposed to result from the heating of the lattice [14]. Here, we provide kinetic evidence that the spectral shift is caused by the vibrational Stark effect. The dichroic polarization dynamics measurements for both excitons and photocarriers reported in anisotropic phases of hybrid perovskites (MAPbI_3_) at ambient temperature allowed the measurement of the exciton diffusion time constant and a photogenerated branching ratio of these charged species, which aid more advanced applications [19].

## 2. Results

We have investigated the dependence of the visible pump excitation irradiance (power density), varied by four orders of magnitude, on the electron absorption and charge recombination kinetics of the selected organic-inorganic perovskite films with the use of a femtosecond mid-IR transient absorption. The selected perovskite materials included single and mixed types: MAPbI_3_ (300 nm thick), MAPbBr_3_ (200 nm or ~500 nm thick), (FAPbI_3_)_0.97_(MAPbBr_3_)_0.03_ (400 nm or 600 nm thick) were fabricated by a spin-coating method using various procedures (details can be found in Section 5). The MAPbI_3_ represents the prototypical perovskite, the MAPbBr_3_ was chosen for its stability, and the (FAPbI_3_)_0.97_(MAPbBr_3_)_0.03_ material was selected to investigate the reduced second-order recombination. Their static visible and FTIR in-band spectra are presented in the Supplementary Materials, Figures S70–S76. For all the samples a pump wavelength at 539 nm was chosen. For the 500 nm MAPbBr_3_, a non-resonant excitation at 560 nm was also utilized [16]. As a probe, mid-IR pulses with a Gaussian spectral distribution centered at 1500 cm^−1^ or 1713 cm^−1^ (covering, respectively, a range of 1400–1600 cm^−1^ and 1600–1750 cm^−1^) have been applied. The time delays were selected at −10 ps, −5 ps, 0.5 ps, 1 ps, 2 ps, 5 ps, 10 ps, 20 ps, 30 ps, 40 ps, 50 ps, 100 ps, 500 ps, 1000 ps, and 1500 ps (positive sign meaning the pump beam comes first).

**Figure 1:**
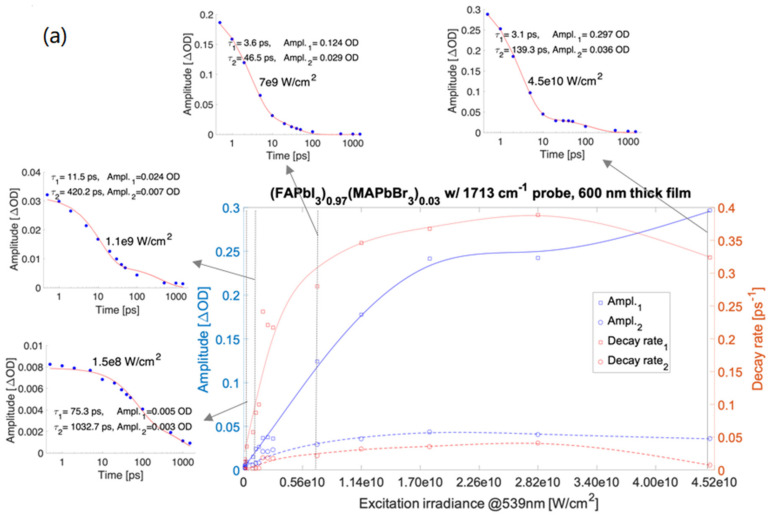

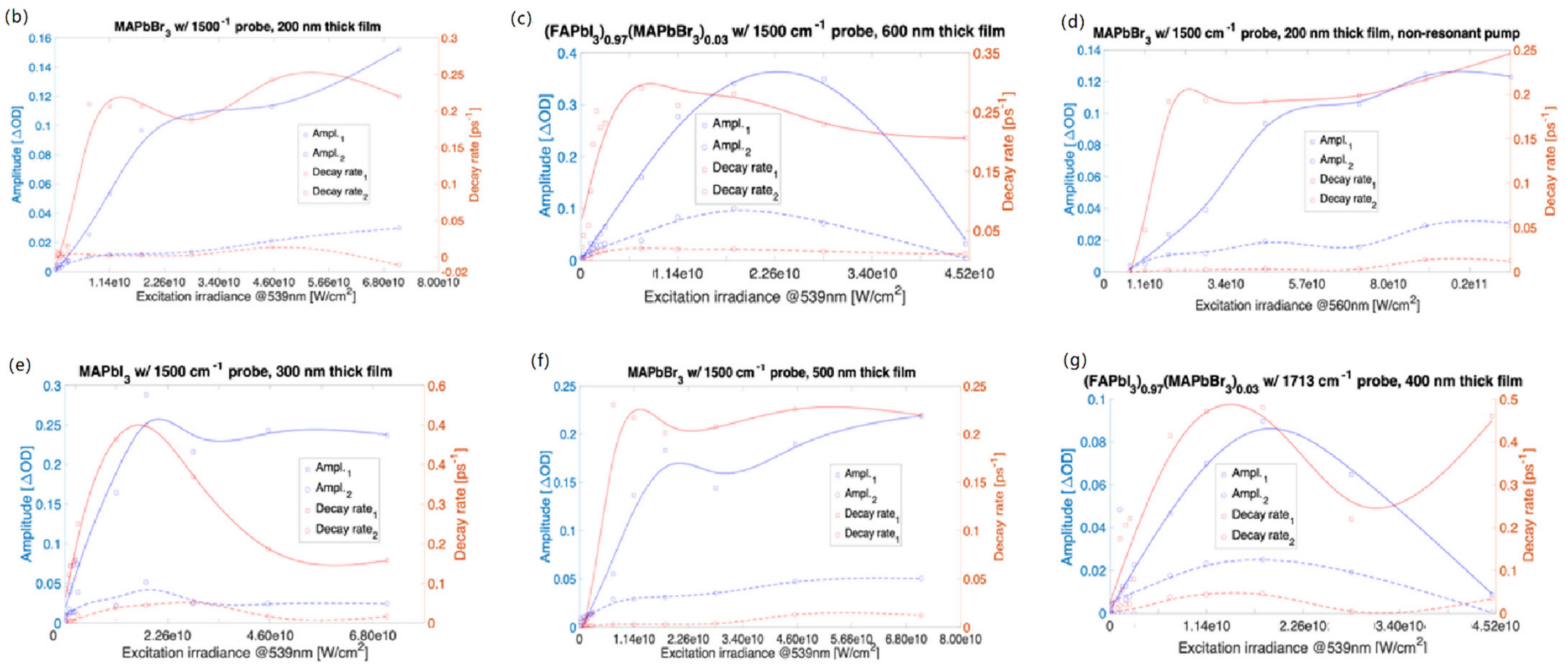
ΔOD amplitudes and decay rates of bi-exponential fitting to difference absorption spectra with a resonant 539 nm pump (also at non-resonant 560 nm (**d**)). (FAPbI_3_)_0.97_(MAPbBr_3_)_0.03_ with 1713 cm^−1^ probe—600 nm thick film, insets are kinetic traces taken at 1713 cm^−1^ for selected irradiances (**a**), MAPbBr_3_ with 1500 cm^−1^ probe—200 nm film (**b**), MAPbBr_3_ with 1500 cm^−1^ probe—500 nm film (**c**), MAPbBr_3_ with 1500 cm^−1^ probe and with a non-resonant 560 nm pump—200 nm thick film (**d**), MAPbI_3_ with 1500 cm^−1^ probe—300 nm film (**e**), (FAPbI_3_)_0.97_(MAPbBr_3_)_0.03_ with 1500 cm^−1^ probe—600 nm film (**f**), and (FAPbI_3_)_0.97_(MAPbBr_3_)_0.03_ with 1713 cm^−1^ probe—400 nm film (**g**). The experimental data are depicted with square and circle symbols. The corresponding curves are the smoothing spline fits applied to amplitudes and decay rates fitted to the kinetic bi-exponentials. An extended version of Figure 1a is presented in the Supplementary Materials, Figure S77. e10 represents 1 × 10^10^.

Single wavenumber kinetic traces of the transient absorption were fitted with bi-exponential decay components (see Supplementary Materials). The amplitudes of mid-IR transient absorption fits shown in Figure 1 varied in the range from ~1 m OD to 0.4 OD. Strikingly, the absorption amplitudes and decay rates vs. irradiance for the kinetic decay components show systematic differences between samples. The damage thresholds of all the materials were determined from the visible spot marks imprinted in the films at the highest applied irradiance (different for every material) and are also presented on the charts in Figure 1.

For the prototypical MAPbI_3_ (Figure 1e), the decay rates corresponding to both the shorter and longer-lived components (marked in red) rise with the irradiance changes up to an irradiance value of 1.8 × 10^10^ W/cm^2^, and for the greater values of irradiance, the rates decrease. For both the components, the amplitudes determined for measurements on MAPbI_3_ (in blue) grow with irradiance changes ranging from 1.5 × 10^8^ W/cm^2^ to 1.8 × 10^10^ W/cm^2^ for both the decay rate and amplitude dependence of irradiance and become independent of irradiance variations in the range of 1.8 × 10^10^ W/cm^2^ to 7.1 × 10^10^ W/cm^2^.

The irradiance dependence of the decay rates for (FAPbI_3_)_0.97_(MAPbBr_3_)_0.03_ is similar to that of MAPbI_3_. The amplitudes of transient absorption measured for (FAPbI_3_)_0.97_(MAPbBr_3_)_0.03_ as a function of irradiance increase for irradiance changes in the range of 9 × 10^7^ W/cm^2^ to 2 × 10^10^ W/cm^2^. A decrease of amplitudes shown in Figure 1f,g for irradiance in the range of 2 × 10^10^ W/cm^2^ to 4.5 × 10^10^ W/cm^2^ was caused by sample damage.

For the film thicknesses of both MAPbBr_3_ perovskites under study (200 nm and 500 nm, the decay rates of the shorter-lived component increase (as in the MAPbI_3_ case)) with irradiance in the range of 1.5 × 10^8^ W/cm^2^ to ~1.1 × 10^10^ W/cm^2^, and becomes weakly dependent on irradiance changes in the range of 1.2 × 10^10^ W/cm^2^ to 7.1 × 10^10^ W/cm^2^ (cf. Figure 1c,d). The 200 nm thick MAPbBr_3_ film excited at 10^11^ W/cm^2^ irradiance, below the damage threshold and using a non-resonant pump at 560 nm, noticeably shows a further increase of the shorter-lived component decay rate as a function of irradiance. For all MAPbBr_3_ samples (200 nm thick film excited at 560 nm or 539 nm, and 500 nm thick film pumped at 539 nm), the decay rates of the long-lived component (roughly spanning ~100 to ~1000 ps) weakly depend on irradiance changes. The amplitude values in the applied irradiance range of 1.5 × 10^8^ W/cm^2^ to 7.1 × 10^10^ W/cm^2^ were nearly 50% greater for a 500 nm thick film as compared to the 200 nm film (cf. Figure 1c,d). For a non-resonant pump, the applied range of irradiance was from 7 × 10^9^ W/cm^2^ to 1.1 × 10^11^ W/cm^2^.

Additionally, the difference spectra have been analyzed using the SVD and Global Analysis methods [20]. The SVD method extracts the time constants from fitting first-order exponentials to the global fit of time traces of orthogonal components in a least squares manner. From the analysis of the time constants and concentration profiles (signal amplitudes in time), a homogeneous model was inferred assuming that the recombination processes represented by components evolve sequentially. In the global analysis, the amplitudes that correspond to the time constants are determined using established methods [20]. The SVD and global analysis provide evidence from orthogonality for the association between the resonant vibrational molecular response and the carrier dynamics. It can be misleading to base the assignments on the time constants in comparison with the literature values (even when time constants significantly differ between the components) since the bimolecular and Auger recombination processes can change over the dominant character of the decay (by outrunning one another) in the applied excitation range depending on a perovskite material. We, therefore, also apply non-linear global fitting.

**Figure 2:**
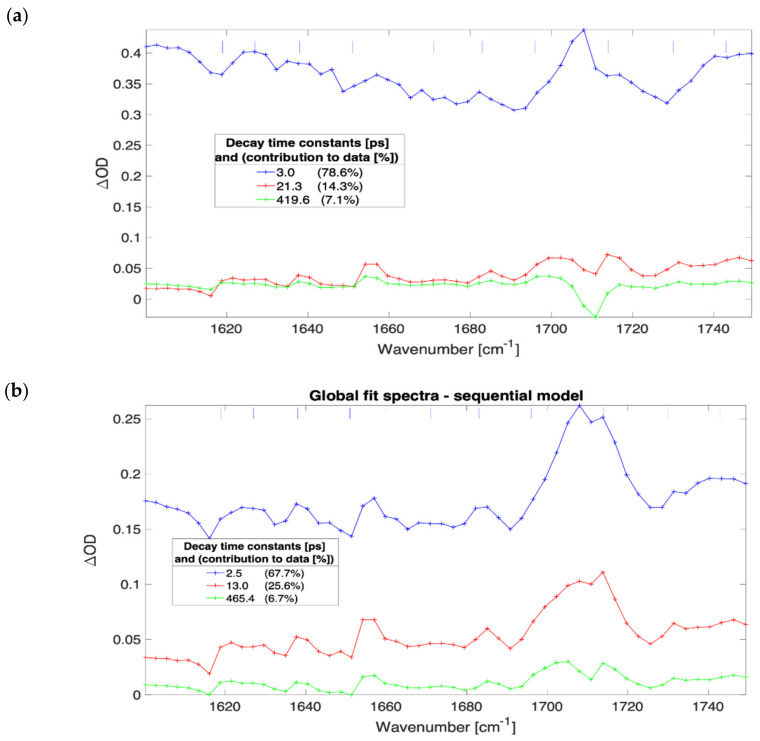

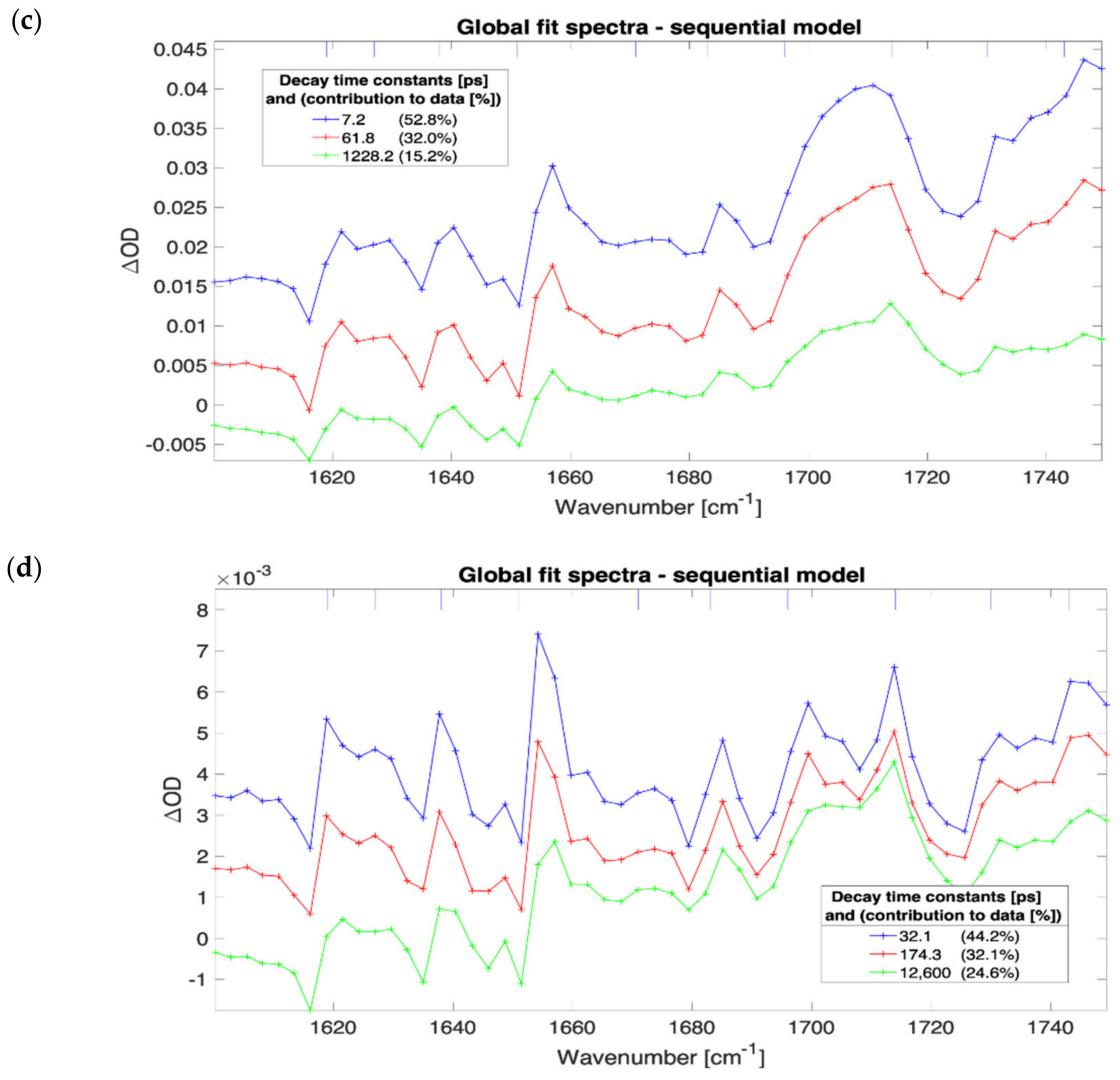
Results of three compartments sequential global fit to data for 600 nm thick film of (FAPbI_3_)_0.97_(MAPbBr_3_)_0.03_. The irradiance and SVD time constants; 4.5 × 10^10^ W/cm^2^, 3.0 ps, 21.3 ps, and 419.6 ps (**a**); 7.1 × 10^9^ W/cm^2^, 2.5 ps, 13.0 ps, and 465.4 ps (**b**); 9.1 × 10^8^ W/cm^2^, 7.2 ps, 61.8 ps, and 1228.2 ps (**c**); 9.1 × 10^7^ W/cm^2^, 32.1 ps, 174.3 ps, and 12,600.0 ps (**d**).

The evolution of the globally fitted time-independent difference transient absorption spectra as a function of irradiance for a 600 nm thick film of (FAPbI_3_)_0.97_(MAPbBr_3_)_0.03_ are presented in Figure 2. The FTIR spectra for this material showed a strong and narrow static absorbance line of 0.5 OD at 1713 cm^−1^ (see Supplementary Materials Figure S70) that corresponds to the C=N stretch in formamidinium [14,21]. At irradiance greater than 7 × 10^9^ W/cm^2^ and pumping at 539 nm wavelength the transient spectra feature a bleach for all the time delays in the investigated range between 0.5 ps to 1500 ps (cf. Supplementary Materials Figure S3.1). The bleach has been attributed to couplings between the low-frequency collective vibrations (phonons) to high-frequency vibrations (molecular modes) [22]. The MAPbI_3_ and MAPbBr_3_ samples exhibit much lower ground state absorbance bands of the C=N stretching mode, and transient absorption is not detected with the background of systematic modulations in the TA spectra (depicted in Figure 2 with vertical short blue lines). These amplitude modulations were found at frequencies that match atmospheric water absorption lines even under strongly purged conditions. The spectral imprinting is likely caused by pump-induced propagation effects and residual water absorption, but it did not prevent the continuum absorption analysis presented here.

The SVD analysis revealed three dominant time constants for every sample except MAPbBr_3_ for which four components were present (the Supplementary Materials contain the sample and irradiance specific concentration profile charts, presented in Figures S1–S69.5). The slowest and weakest third decay component with a time constant on the order of 1000 ps (assumed excitonic by matching against literature values) contributes the least to the transient spectra. The global analysis concentration profiles (shown in Supplementary Materials presented in Figures S1–S69.5) provided evidence for the sequential order of the exponentially decaying components.

**Figure 3:**
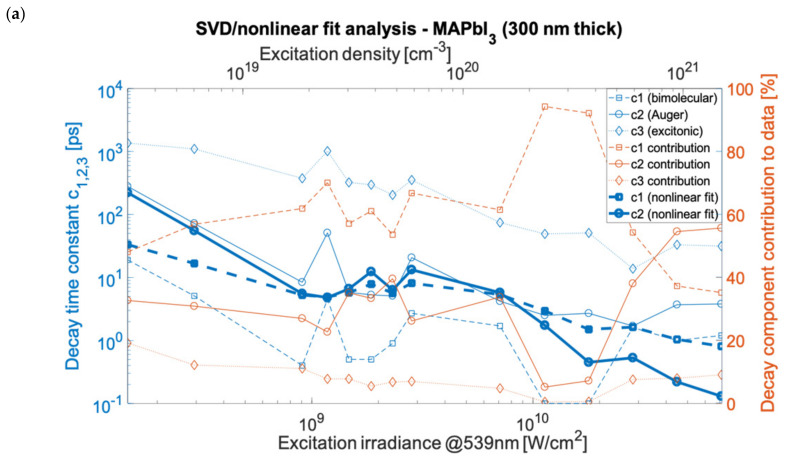

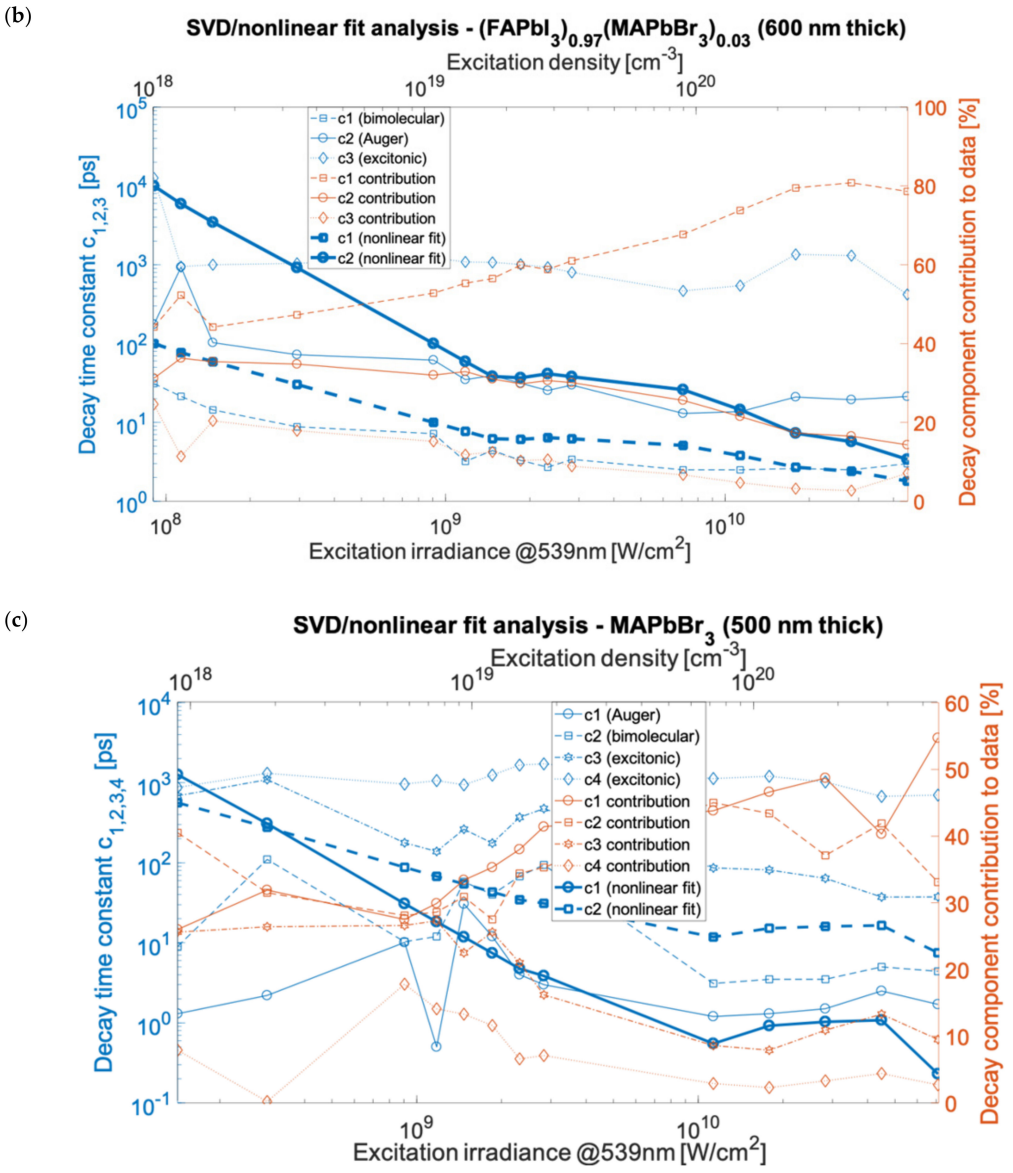
SVD analysis of kinetics for MAPbI_3_ with 1500 cm^−1^ probe (**a**), (FAPbI_3_)_0.97_(MAPbBr_3_)_0.03_ with 1713 cm^−1^ probe (**b**), and for MAPbBr_3_ with 1500 cm^−1^ probe (**c**). The decays time constants c_1,2,3,4_ and their contribution to data correspond to: c_1,2_—Auger or bimolecular recombination, c_3_—trapped-exciton recombination, c_4_—exciton/biexciton or polaron recombination (symbols depict measurement points).

Based on the decay constant values obtained using the nonlinear fit (compared with decay constants published elsewhere [23]), the time constants were calculated. These matched the various SVD results as well, as presented in Figure 3 using the estimated, sample, and excitation specific carrier densities (see Section 5). The Supplementary Materials contains the data and fitting results for the individual measurements shown in Figure 3. The nonlinear fitting of components was done assuming the first-order decay rate as imported from SVD analysis. The processes underlying the constants were attributed to the bimolecular inter-band electron-hole recombination, Auger decay process, and excitonic recombination. In the double hybrid perovskite case, the bimolecular recombination outruns Auger decay (nonlinear fit in Figure 3b) for all applied excitation ranges and dominated in the global (time-independent) spectra. The decay time constants were in the range of 4 ps to 10 ns for Auger decay and for bimolecular recombination in the range of 2 ps to 100 ps. However, for MAPbI_3_ (Figure 3a), the nonlinear time constants of bimolecular and Auger recombination intersect at around 10^9^ W/cm^2^, and for MAPbBr_3_ (Figure 3c), they intersect at 3 × 10^8^ W/cm^2^. At power densities below the crossover point, the bimolecular recombination overtakes the Auger decay. An additional fourth component was found in the linear SVD and global analysis for the measurements of the 500 nm film of MAPbBr_3_, which had a time constant in between the bimolecular and excitonic relaxation decays with a lifetime (depending on irradiance) in the range of 37.4 ps to 1093 ps. Those values match the lifetimes for free exciton, polaron, or bi-exciton (two bound excitons) dynamics, which can form under high excitation. These are responsible for the additional decay component found in this material. The exciton binding energy, which is above thermal energy in MAPbBr_3_, favors the creation and stability of polaronic and excitonic states. This is in contrast to MAPbI_3_, where the binding and thermal energies are comparable.

The SVD and global analysis provide the spectral analysis and fundamental time constants that characterize the time-dependent measurements assuming the decomposition of linear combinations. Subsequently, we applied the method of non-linear fitting to address the assignment of photoinduced processes. Using the nonlinear fitting (see in Section 5), we quantified the lifetimes (time constants) of the Auger recombination to be in the range of 2 ps to 280 ps (MAPbI_3_), 1.2 ps to 30.6 ps (MAPbBr_3_), and 13 ps to 934 ps ((FAPbI_3_)_0.97_(MAPbBr_3_)_0.03_). For bimolecular recombination, the corresponding lifetimes were 0.1 ps to 19 ps (MAPbI_3_), 3 ps to 110 ps (MAPbBr_3_), and 2.5 ps to 31.1 ps ((FAPbI_3_)_0.97_(MAPbBr_3_)_0.03_). For the excitonic recombination (from SVD analysis) the lifetimes were in the range of 13.8 ps to 1363 ps (MAPbI_3_), 674 ps to 1719 ps MAPbBr_3_), and 420 ps to 12.6 ns/1352 ps ((FAPbI_3_)_0.97_(MAPbBr_3_)_0.03_). For MAPbI_3_, the carrier concentration (electron-hole pair density) corresponding to the applied irradiance range of 9 × 10^7^ W/cm^2^ to 4.5 × 10^10^ W/cm^2^ has been estimated to be in the range of 3.1 × 10^18^ cm^−3^ to 1.5 × 10^21^ cm^−3^ (see Section 5). For MAPbBr_3_ and (FAPbI_3_)_0.97_(MAPbBr_3_)_0.03_ perovskites, the values of the excitation density were comparable, respectively, in the range of 0.9 × 10^18^ cm^−3^ to 4.5 × 10^20^ cm^−3^ and 10^18^ cm^−3^ to 5 × 10^20^ cm^−3^, depending on the film thickness and absorbance. The generated carrier density is linearly dependent on the irradiance only for the lower excitations in the applied range [23]. However, for irradiance greater than 2 × 10^8^ W/cm^2^ the dependence of the excitation density on irradiance becomes nonlinear as a result of multiphoton processes [23,24]. The recombination processes are associated with different orders in regard to the carrier density, namely, the third-order Auger process depends on the 3rd power of the density, bimolecular recombination is the second-order process and excitonic is the first-order process [23]. Moreover, in the applied density range of ~10^8^–10^11^ W/cm^2^, the ratio of the generated free charge carriers to excitons, as described by the Saha–Langmuir relation (valid in the linear regime of interaction and excluding many body processes), is expected to vary between ~50% (for the lowest irradiance) and ~1% (highest irradiance) [2,25]. Therefore, the presence of a specific recombination component in the applied high excitation density regime will show a different strength depending on irradiance. Taking into account the nonlinear photon-electron interaction yielding reduced excitation density, the minimal ratio condition is relaxed and greater than 1% for the highest irradiances in the applied ranges.

In MAPbI_3_, for the highest experimental excitation densities (irradiance > 2 × 10^10^ W/cm^2^) the Auger process dominates the transient absorption signals). For MAPbI_3_ and also (FAPbI_3_)_0.97_(MAPbBr_3_)_0.03_, supported by both the SVD and nonlinear analysis, for the irradiance range of ~10^9^ W/cm^2^ to ~10^10^ W/cm^2^, the recombination rate dependencies become weakly dependent on irradiance. For measurements made of MAPbBr_3_, the time constants of bimolecular and Auger decays weakly increase with irradiance in the range of 10^10^ W/cm^2^ to 4 × 10^10^ W/cm^2^. For irradiance less than 10^9^ W/cm^2^ in MAPbBr_3_, we observed a significant discrepancy between the decay time constant values in irradiance dependencies of the SVD and nonlinear analyses. The Auger decay constants measured using the nonlinear fit were: for MAPbI_3_ k_3_ = 5 × 10^−28^ cm^6^/s, for MAPbBr_3_ k_3_ = 10^−27^ cm^6^/s, and for (FAPbI_3_)_0.97_(MAPbBr_3_)_0.03_ k_3_ = 10^−28^ cm^6^/s. The bimolecular decay constants were estimated: for MAPbI_3_ k_2_ = 10^−8^ cm^3^/s, for MAPbBr_3_ k_2_ = 2 × 10^−9^ cm^3^/s, and for (FAPbI_3_)_0.97_(MAPbBr_3_)_0.03_ k_2_ = 10^−8^ cm^3^/s. The quantum conversion yield of absorbed photon density per optically injected carrier concentration was measured to be 10% at irradiance greater than 10^10^ W/cm^2^ due to nonlinear absorption.

It is noted that the percentage contribution (presented in Figure 3) to the TA signal corresponding to a particular relaxation component as a function of time particularly seen in MAPbI_3_ is complex. It is seen that a density at which the dominant contribution to TA spectra changes over from one to another recombination channel happens at a higher irradiance than the kinetic lifetimes change-over retrieved from SVD analysis. In the case of MAPbI_3_, for irradiance smaller than 10^10^ W/cm^2^ the excitonic and Auger contributions decrease with increasing irradiance, whereas bimolecular contribution increases. For irradiance in the range of ~10^10^ W/cm^2^ to ~3 × 10^10^ W/cm^2^ we observed a significant rise in the contribution of bimolecular recombination to TA spectra after which follows the dominating contribution change-over (Figure 3a). For measurements of (FAPbI_3_)_0.97_(MAPbBr_3_)_0.03_, the increasing contribution to transient absorption of bimolecular recombination in the irradiance range of 10^8^ W/cm^2^ to 2 × 10^10^ W/cm^2^ becomes independent of irradiance at the highest measurement points (cf. Figure 3b). This indicates that the onset of the change-over process is only enabled at the highest excitations. For measurements made of MAPbBr_3_, the contribution of the Auger process is less than 10% greater than that of the bimolecular recombination for most of the applied irradiance range.

## 3. Discussion

### 3.1. Vibrational Stark Effect

The evaluation of the SVD and global fit results provide evidence that the vibrational response of the C=N mode is kinetically linked to the continuum absorption. This evidence is taken from the homogeneous global fitting, which proves that kinetically, the C=N mode response at an early time has a fixed amplitude relative to the electron absorption continuum absorption. Furthermore, due to the frequency dispersion of the measurement, the spectral position and shape are correlated in the same way. The correlation is, therefore, shown on the basis of orthogonality assuming linear combination following the SVD procedure. The data for both film thicknesses (400 nm and 600 nm) formamidinium containing samples were collected in the same range of irradiance. For the 400 nm sample, the difference spectra were clearer around the C=N mode as compared to 600 nm film spectra since they did not exhibit the imprinted bleach (only a relatively weak for the highest excitation powers). The feature is persistently present at all time delays with the amplitudes stronger at earlier times and vanishing after 1 ns, and stronger (relative amplitudes compared to continuum spectra amplitudes) at higher irradiances. For each applied excitation power the measurements made of 400 nm thick film, the C=N mode feature was systematically blue shifted by 5 cm^−1^ up to 100 ps. Although the C=N absorption of 0.5 OD was measured in FTIR at 1713 cm^−1^, the transient feature was measured at 1711 cm^−1^ mainly due to a detector channel dispersion corresponding to 3 cm^−1^. Therefore, the bleach was measured at 1706 cm^−1^ and induced absorption at 711 cm^−1^. The analysis of C=N for 600 nm film was problematic since the spectral shift is clearly observed only for times shorter than 10 ps and only for the two strongest excitation irradiances. In these measurements, the main cause was likely the presence of an atmospheric absorption line located within the C=N feature region. Moreover, there is an imprinted bleach (strong for this sample) that obscures the precise readout of the mode peak. In a single scan that was recorded at high excitation with a stationary sample, the measured spectral shift was blue shifted by any 4 cm^−1^.

**Figure 4:**
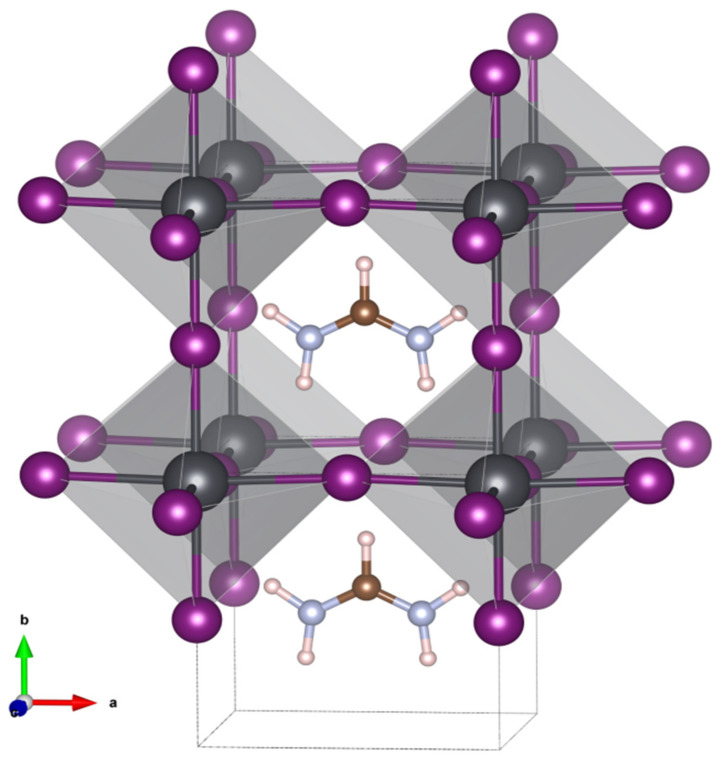
Model of the cubic crystal structure for the black formamidinium lead iodide, α-[HC(NH_2_)_2_]PbI_3_, at 298 K [26].

A harmonic frequency calculation addressed the vibrational Stark effect (VSE) observed for the asymmetric stretching vibration of the formamidinium group. The anisotropic field effect was evaluated for Cartesian coordinates that correspond to the crystallographic directions of the crystal structure reported. Specifically, a cubic space group number 221, Pm3m was determined for FAPbI_3_ at room temperature. In this model, the crystallographic b direction is along the C-H bond of the formamidium group which is directed into the cubic face [26] (see Figure 4).

**Figure 5:**
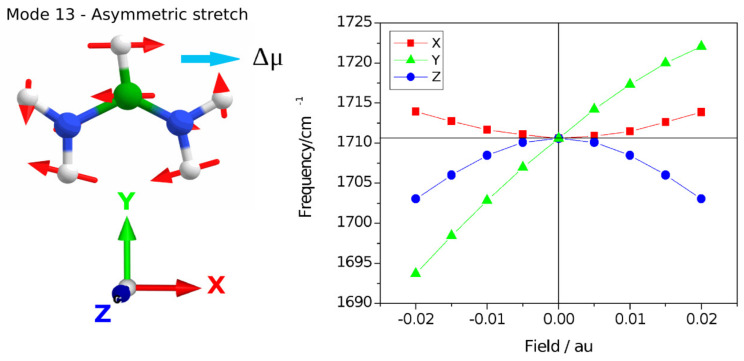
Calculation of the vibrational Stark effect on the asymmetric stretching mode at the b3lyp/6-311+g(d,p) level. The transition dipole moment for mode 13, Δμ, is indicated corresponding to the direction of the N-N vector. The harmonic frequencies following geometry re-optimization are scaled by 0.967 and shown for application of fields in the X, Y, and Z directions separately.

Harmonic frequencies were calculated following respective geometry re-optimization of coordinates using DFT at the b3lyp/6-311+g(d,p) level with the application of external electric field X, Y, and Z directions (see Figure 5). Using Gaussian-16 [27] the units of the applied field were chosen between −0.02 au and 0.02 au, where 1 au field equals 5.142 × 10^11^ V/m. The vibrational Stark effect was found to be dominated by the field-dependent geometry changes. The static dipole moment is calculated to be 0.5056 Debye with an out-of-plane vector with a dominating component in the Z direction normal to the N-C-N plane but a minor component in the Y direction along the C-H axis (0.0043, −0.1807, −0.4722).

The calculations show that applying fields in the X and Z directions results in a quadratic VSE, where application in the Z-direction creates a downshift irrespective of inversion of the field direction. Similarly, a field in the X direction shows a quadratic behavior with a weak upshifting of the frequency independent of the field direction. The VSE with Y-direction is dominated by the linear component and the direction of the frequency shift inverts with the inversion of the direction of the field, the negative direction showing a slightly stronger Stark tuning rate. It is well known that the VSE for most systems has six physical contributions and may be evaluated on the basis of perturbation theory [28]. Here we focus on the conclusion that the experimentally observed VSE is a 5 cm^−1^ upshift of the frequency of the asymmetric stretching mode that our calculations indicate corresponds to the application of a net electric field in the Y direction along the C-H bond vector. This is particularly interesting with regard to the cubic crystal structure, as the three-fold symmetry elements in all directions cause the permutations of the field to be equal to the isotropic case for interactions that have a rank of two. We consider the magnitude of the Stark tuning rate obtained in the Y direction which is approximately 10.3 MV/cm per wavenumber. At 1713 cm^−1^, the five wavenumbers experimental difference is Δhν = 9.925 × 10^−23^ C.V. Then the difference dipole equals 9.925 × 10^−23^ C.V/5.142 × 10^9^ V/m = 1.93∙10^−32^ C m, so Δμ = 0.0058 Debye. This is a reasonable value, compared for instance with the nitrile stretch which is associated with a 0.041 Debye dipole moment [29]. The five inverse wavenumber experimental upshift, therefore, reports on an experienced Field of ~50 MV/cm. The effective screening and dielectric medium imply that the generated field in a vacuum could be several times greater. A structural interpretation considers for instance a point change of an elementary charge at a 6 Å distance, that would yield a field of 3.99 × 10^9^ N/C = 3.99 × 10^9^ V/m (~40 MV/cm). This is comparable to the Gaussian calculation and suggests that the VSE reports on the field-effect of a single elementary charge on the length scale of the crystallographic asymmetric unit with a positive value of the direction of the field. This result may inform theoretical work on microscopic field theory in the presence of crystal symmetry. In the space group 221, cubic, there are 48 Wyckoff transforms that synthesize the symmetry equivalent coordinates and should enter a multipole and shielding calculation following simulations of charge dynamics. A microscopic theory of charge distribution can therefore be informed by this experimental study that provides an estimate of the field magnitude and direction along the C-H bond vector.

### 3.2. Generated Carrier-Species Dynamics

The experimental data have been processed using three complementing ways; fitting bi-exponentials to difference spectra kinetics at a selected single wavenumber (to provide the peak amplitudes and kinetic traces of the transients), performing SVD analysis for all TA spectra (yielding for the linear components the time constants and percentage share in the spectra) or nonlinearly fitting the decay constants to transient spectra (resolving decay components and deriving Auger and bimolecular time constants).

The Drude model has been successfully utilized in some classes of semiconductors [30]. As explained in the Methods paragraph, using refs. [5,6,10], carrier densities were calculated to be in the range of 10^18^ cm^−3^ to 5 × 10^20^ cm^−3^ (corresponding to fluence in the range of 23 µJ/cm^2^ to 1120 µJ/cm^2^ or irradiance of 9 × 10^7^ W/cm^2^ to 4.5 × 10^10^ W/cm^2^). Since the experimentally determined carrier concentration range falls in the range of carrier concentration that the Drude model is considered to be valid (model’s breakdown threshold ~10^21^ cm^−3^), the free carrier absorption in perovskite data can be described by the model [30,31,32]. This model assumes an inverse quadratic (~*ω*^−2^) dependence of absorption on the photon frequency that results in relatively flat continuum absorption spectra in the mid-IR range [13]. In this work, we have characterized the bimolecular (inter-band electron-hole), many-body Auger, as well as exciton recombination kinetics. The experimental pump fluences exceed the linear interaction limit (~60 µJ/cm^3^) hence reducing the generated carrier number to absorbed photon number ratio [23,33] (Supplementary Materials). The measurements resolve the dynamics up to 2 ns after excitation. A long-lived, trap-assisted monomolecular recombination (with a lifetime ~100 ns) [23] dominantly contributes to transients in hybrid perovskites in the PV regime range [17,34] and relatively weakly for the experimental excitation ranges. Therefore, we could not observe the monomolecular recombination. High excitation irradiance used in the experiment results in the predominant contribution of the Auger process to TA spectra amplitudes in Figure 3, due to its third power dependence on free carrier concentration. Also, this is evidenced in the rising contribution of the Auger component spectrum under an irradiance increase presented for a series of irradiance in the spectra evolution of Figure 1.

The bound states, which are enabled via Coulomb interaction, can be a mix of various species with a majority of free and self-localized (self-trapped) excitons with their mutual population ratio dynamically changing following photoexcitation [17]. Small polarons can also be transiently generated to form self-trapped excitons [35]. For experimental carrier excitation densities, the majority of the generated carriers upon excitation according to the Saha–Langmuir relation are excitons [36]. The relation predicts a temperature-dependent ratio of free carriers to excitons after their concentrations reach equilibrium [19,37,38]. The Saha–Langmuir relationship depends on the perovskite material-specific exciton binding energy (energy needed to ionize the exciton into a free electron-hole pair) and the sample temperature. The Auger processes depend strongly on the excitation density and thus in MAPbI_3_ at irradiance around 10^9^ W/cm^2^, they overtake bimolecular recombination, inferred from the nonlinear fit (Figure 3a). In addition, in MAPbI_3_ for the excitation density greater than ~10^18^ cm^−3^ corresponding to ~4 × 10^8^ W/cm^2^ irradiance, the bound states of charge can disintegrate back into the free carriers (Mott transition) creating a plasma mixture [2]. This has been proposed to be driven by an increased screening of Coulombic bounding of exciton due to an increased number of the surrounding other carriers and lattice carrier-phonon interactions upon increasing the excitation power [2].

The interconversion between different recombination processes can be the reason for the discrepancy in the nonlinear decay order [39,40]. Taking into account the interconversion between photo-generated species in the Saha–Langmuir equation makes a chemical equilibrium difficult to reach [17]. The excitonic recombination is expected to be the first-order process (decay rates independent of the excitation level), however, we observed in MAPbI_3_ a monotonic drop of the rate constants that were assigned to exciton decay, with the slope resembling the second-order bimolecular decay. Other processes can also be present, for instance, phonon generation, exciton trapping, and at high excitation multi-exciton binding or exciton dissociation [2]. It was reported that biexcitons (bimolecular process) with a 0.4 ns lifetime have been formed for a FAPbI_3_ film, which has a comparable exciton binding energy to that of MAPbI_3_ [17,41] at excitations corresponding to irradiances applied in the experiment. This is consistent with the observed near-quadratic lifetime dependence on irradiance [42]. For the double hybrid perovskite, a very high excitation ground state absorption of 3.75 OD is believed to prevent the change-over in the kinetics of the two dominant recombination processes before sample damage occurs.

Organic semiconductors support the formation of a small exciton (Frenkel), with its size contained within interatomic separation due to the high effective mass of such exciton and low dielectric constant of the material [41]. In contrast, the inorganic semiconductor will lead to generating a large exciton (Wannier–Mott), spanning several unit cells in real space due to associated low effective mass and high dielectric constant (which efficiently screens exciton’s Coulomb bond) [2]. However, the excitons found in hybrid perovskite materials are mostly of the Wannier–Mott type [2,43], which becomes clear given the photo-generated charge carriers are created in an inorganic part of perovskites. Hybrid perovskite materials support exciton creation at room temperature since the estimated free exciton binding energy E_b_ using TA techniques [19] is greater (35 meV for MAPbI_3_ and 115 meV for MAPbBr_3_) than the thermal energy (k_B_T = 26 meV at 300 K) [44]. Although, the binding energy values vary in the literature. By using a four-way-mixing technique the binding energy of 16 meV (less than thermal energy) for free exciton and of 29 meV for defect-trapped exciton was reported [45], suggesting the trapped excitons exist next to free carriers in these materials. Hybrid perovskites are known for their polar character of organic cation, and the electron-phonon coupling is characteristic of polar materials [46,47]. Since self-trapping of the exciton requires activation energy to overcome—the resulting binding energies of those species are higher, making them thermally more stable than free excitons [18]. Therefore, the self-trapping of Wannier–Mott excitons is mediated by the presence of electron/exciton couplings to LO phonon and other modes in existence [35]. The strong coupling of excitons to the crystal vibrations (optical phonons) localizes exciton to a unit cell [48], which eases an experimental identification of the localized photo-generated species. Optical phonons emerge at early times (on a femtosecond time scale) after the excitation (in addition they can derive from the Auger process) [34]. Thus, self-trapping emerges on a picosecond or even femtosecond time scale and therefore in thin polycrystalline films quenches free excitons at room temperature and above [3,49]. The lifetime of the localized excitons is on the order of a nanosecond. The net electric field of the trapped excitons can explain the origin of the observed vibrational Stark effect in C=N mode, which we calculated to act at ~0.6 nm distance (comparable to the lattice constant dimension). This is to be compared with the estimated Bohr radii of ~20 Å for both MAPbI_3_ and MAPbBr_3_ free excitons [50]. This observation together with the lifetimes that were determined supports an argument that self-localized (polarizing) excitons are dominantly present in the samples under our experimental conditions. The self-trapping that was suggested to be a precursor of crystal defect generation is likely responsible for the sample damage observed at very high irradiances [51]. The homogenous broadening of free excitons found in photoluminescence Stokes-shifted spectra at low temperatures also stems from electron to phonon coupling [49,52]. This broadening can be advantageous in light-emitting applications (that match our experimental excitation range) [53]. However, a very strong electron-phonon interaction (measured by the Huang–Rhys factor [54]) will result in non-radiative decay via releasing phonons, in turn too weak coupling will prevent trapping, limiting the applicable excitation range window [53]. The indirect bimolecular recombination pathway (via the Rashba effect) that is believed to slow down Langevin e-h recombination in bulk perovskites is also mediated by phonons [55]. Finally, a nonlinear absorption regime can facilitate transitions within the exciton’s energy ladder. The above-described processes should be taken into consideration to predict light emission conditions for the hybrid perovskites.

**Figure 6:**
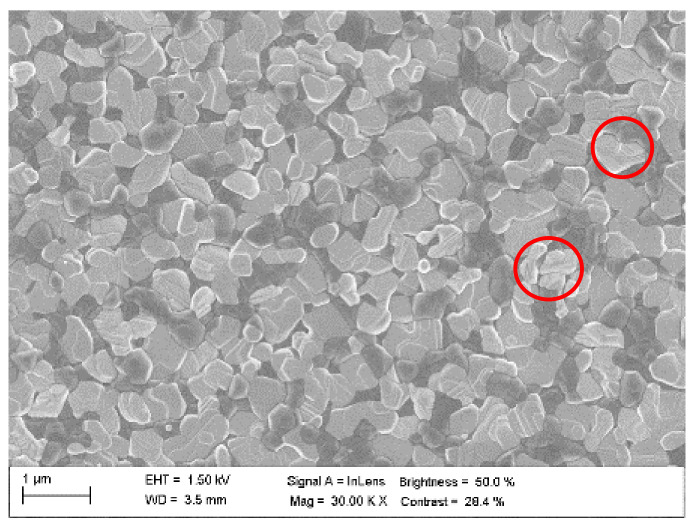
PbI_2_ defect passivation of (FAPbI_3_)_0.97_(MAPbBr_3_)_0.03_ film (symbols depict lighter perovskite domains with greater PbI_2_ passivation).

The applied two-step deposition process during the fabrication of the doubly hybrid perovskite aimed at leaving in the film some amount of PbI_2_ precursor unreacted with the perovskite phase. There was no further optimization and the material represents typical fabrication procedures and results. Figure 6 shows the SEM photos of this film with visible crystalline domains. The purpose of PbI_2_ addition was to passivate defects (reduce the defect density) to mitigate (slow down) the bimolecular recombination. The defects are located in the bandgap, hence they create additional recombination channels (mainly non-radiative) that speed up the free carrier recombination. The impact of the passivation is evidenced in Figure 3, where for irradiance greater than 10^10^ W/cm^2^ the bimolecular recombination lifetimes for (FAPbI_3_)_0.97_(MAPbBr_3_)_0.03_ are around one order of magnitude greater as compared to MAPbI_3_ (actually the lifetimes of all decay components are evidently longer). Excitons may also be trapped by impurities and defects that bind them (impurity-excitons) [2,35,45]. Oxygen may act as an example of such impurity in wet perovskite films. Impurity-excitons are associated with giant oscillator strength, which determines their non-local nature. Defect-related trapping was found to be concentrated at the grain boundaries [56]. Thus, it depends on material morphology, which gives a way to control the trapping in the fabrication process to support a specific application. Small grains, due to a greater Coulomb screening, favor free carriers, and the large ones a formation of exciton [41]. We note that the crystal grain dimensions vary between roughly 100 nm and 500 nm (comparable with the film thickness), which together with the obtained results suggest the exciton generation. The actual ratio of impurity/defect to phonon-trapped excitons is material-specific and depends on excitation parameters.

## 4. Conclusions

We show that mid-IR spectroscopy, which is complementary to customary THz spectroscopy, enables the study of excitonic states in semiconductors. Reaching high excitation powers and investigating carrier dynamics above the PV regime in combination with the Stark-shifted vibration mode made it possible to assign the different charge species dynamics in selected hybrid perovskites. That aids the literature debate in understanding photophysical processes regarding free and bound photo-generated species in these new materials. We confirm at high fluences the recent observation of the vibrational Stark shift in the C=N mode of formamidinium [14].

By broadly varying the excitation intensity, we also address the light-emission properties of hybrid perovskites. Materials that exhibit excitonic recombination in the photovoltaic (PV) range are favorable in light-emitting devices because their quantum yields depend linearly on the exciton density as opposed to the quadratic dependence of bimolecular recombination on the free charge density [41]. Since photoluminescence is realized through bimolecular and excitonic recombination (cross-sections being greater for excitons), the quantum yield of luminescence can be enhanced by using higher excitation irradiance since that supports this decay channel. In [41] it is emphasized that for light-emitting devices the radiative recombination densities should be kept above the trap densities and below the Auger densities. Although in the PV regime (at excitation density below 10^15^ cm^−3^) the reduction of the bimolecular recombination would be desirable, the opposite is beneficial at higher excitations in the light-emitting applications and lasing perovskite regime. We have shown that in the prototypical MAPbI_3_ only at irradiance >3.5 × 10^10^ W/cm^2^ the Auger process overtakes the bimolecular recombination and contributes more to the transient absorption spectra.

Using the nonlinear fitting, the measured Auger decay constant values in studied perovskite materials corresponded to those reported in the subject’s literature. However, the second-order bimolecular constants for MAPbI_3_ and (FAPbI_3_)_0.97_(MAPbBr_3_)_0.03_ were one to two orders greater than their literature counterparts, whereas for MAPBr_3_ the values were consistent. These measured values were validated with the use of the linear global and SVD analysis. The reason for the discrepancy between the obtained and literature decay constants can originate from the production setup of the samples being the same for both MAPbI_3_ and the double hybrid. Interestingly, in these samples, the faster bimolecular decay dominated the Auger process at very high irradiances (up to the damage thresholds) making them from this point of view promising materials for light-emitting devices.

Perhaps, another applicable window of induced charge pair densities for (FAPbI_3_)_0.97_(MAPbBr_3_)_0.03_ perovskite is located above significant Auger recombination densities, where trapped excitons can profoundly decay radiatively and compete with the third-order recombination. However, at the elevated excitations, an efficient cooling process overcoming the drawback of “phonon bottleneck” and damage mechanisms will need to be resolved. Intended by tailored composition engineering the defects passivation in the double hybrid perovskite (aimed at PV applications) has reached its goal, resulting in the slowing down of the bimolecular recombination up to an order of magnitude compared to MAPbI_3_ and MAPbBr_3_.

## 5. Methods

*Femtosecond mid-IR transient absorption instrument*. An output of a laser producing 70 fs pulses at 800 nm and generating at 1 kHz (Spitfire Pro, Spectra-Physics, Santa Clara, CA, USA) was split to produce pump and probe beams. The visible pump ~250 fs (FWHM) pulses were generated by pumping an optical parametric amplifier (TopasC, Light Conversion, Vilnius, Lithuania). The irradiance (power density) of the pump beam was attenuated by applying a reflective neutral density filter with an optical density chosen from the discrete range of 0.1 to 3.5. The visible pump was modulated at 500 Hz with an optical chopper (Thorlabs, Newton, MA, USA). For every time delay measurement point 1000 pairs of pump-on and pump-off spectra were averaged. The probe beamline consisted of another TopasC followed by a difference frequency generator (Light Conversion) to further down-convert the created near-IR pulse to the mid-IR region. The beam then was split to create a signal and reference beams. The grating spectrometers (Triax 190, Horiba, Kyoto, Japan) were equipped with mercury cadmium telluride 128-pixel linear detectors (Infrared Systems Development Corporation, Winter Park, FL, USA). The signal beam was focused into a 75 µm (FWHM) in diam. spot onto a sample by an off-axis parabolic mirror. A spherical lens was used to focus the pump beam into a 300 µm (FWHM) in the diam. spot to provide possibly uniform interaction conditions for the probe. The samples were stationary during data collection. The timing (delay between pump and probe arrival times on a sample) was controlled by a long delay stage placed in the pump beamline. The signals (transient spectra) were recorded, outliers were filtered out from the saved data, and referenced absorbance difference signals (pump-on minus pump-off) were processed in the LabView environment. The data were then post-processed in Matlab and the Toolbox. A 0.5 mm thick Ge disk was placed in the sample interaction plane to estimate time zero (crossing time of probe and pump beams). The instrument was purged with dry air.

*Sample preparation*. The samples of MAPBr_3_ with 200 nm and 500 nm thick films were fabricated in a bit different ways. In the case of 200 nm film the methylammonium lead iodide (MAPbBr_3_), a precursor solution was prepared by making an equimolar mixture of lead bromide (PbBr_2_) and methylammonium bromide (MABr) and then dissolving in a mixture of dimethylmethanamide (DMF) and dimethylsulfoxide (DMSO) (4:1 by volume). The mixture of precursor and solvents was stirred at 50 °C for one hour. The final solution had a molar concentration of 0.4 M and was infiltrated with a 0.45 µm filter prior to spin coating. A 2mm thick CaF_2_ window was treated with oxygen plasma before transferring to a glovebox where 50 µL of MAPBr_3_ solution was deposited on the window, spin-coated, and then treated with the commonly used anti-solvent technique [57,58]. The window was then spun at 3000 rpm (accelerated at 3000 rev min^−1^ s^−1^) for 20 s. At 10 s, 500 µL of diethyl ether (the anti-solvent of choice) was deposited into the solution during the cycle. The film was then annealed by transferring the window to an 80 °C hotplate for 2 min, and then to a 100 °C hotplate for further 10 min. To encapsulate the MAPbBr_3_ film a second CaF_2_ window (1 mm thick) was used. This window was attached to the window with the perovskite deposited on it by the use of a narrow ring of SurlynTM ionomer [59]. A 500 nm thick film of MAPbBr_3_ was deposited onto a CaF_2_ window by spin-coating in a nitrogen atmosphere glovebox. First, a thin layer of PEDOT:PSS with Capstone FS-31 surfactant was spin-coated for the perovskite to wet the window. The precursor (1.25 M) was made by dissolving PbBr_2_ and MABr in a mixed solvent of DMSO and GBL with a volume ratio of 3:7, then it was stirred for 12 h. The precursor was spin-coated at 4000 rpm for 30 s, while toluene was dripped at 15 s. The film was then annealed for 10 min at 100 °C. The two CaF_2_ windows were encapsulated by applying glue to the edges. To prepare the MAPbI_3_ film, 1.0M PbI_2_ (50 μL, TCI chemicals) was spin-coated on CaF_2_ windows at 2000 rpm for 30 s and dried at 70 °C for 30 min. After cooling at room temperature, the PbI_2_ film was dipped into a 10 mg/mL MAI solution and further dried at 70 °C for another 30 min. (FAPbI_3_)_0.97_(MAPbBr_3_)_0.03_ film samples were produced with two thicknesses 400 nm and 600 nm. A CaF_2_ window (2 mm thick) was cleaned sequentially with detergent, acetone, ethanol, and IPA and further treated under UV ozone. Then, 1.3 M PbI_2_ (50 µL, TCI chemicals) in DMF:DMSO (Sigma Aldrich, St Louis, MO, USA, 9.5:0.5 *v*:*v*) was spin-coated onto cleaned CaF_2_ at 1500 rpm for 30 s, and then annealed at 70 °C for 1 min, after PbI_2_ had cooled down to room temperature, the mixture solution of FAI:MABr:MACl (100 µL, 60 mg:6 mg in 1 mL IPA) was spin-coated onto the PbI_2_ at a spin rate of 1500 rpm for 30 s, and thermal annealing of 150 °C for 15 min was processed to form a black and dense (FAPbI_3_)_1−x_(MAPbBr_3_)_x_ perovskite. A small amount of MAPbBr_3_ was added to FAPbI_3_ to improve the perovskite phase stabilization and PV efficiency via increasing the formation energy of non-radiative defects [17]. The windows with MAPbI_3_ and (FAPbI_3_)_0.97_(MAPbBr_3_)_0.03_ perovskites were sealed with another CaF_2_ window (thickness: 1 mm), applying the surlyn using a heat gun at 180 °C beforehand. The prepared samples were loaded in a Harrick holder mounted in the Lissajous sample rotator.

*Linear SVD and global analysis*. The linear kinetic analysis of the transient spectra has been carried out using the toolbox [20], which is a publicly available Matlab module (user manual included). The toolbox decomposes the input data matrix into a linear combination of unique orthogonal components followed by fitting the sum of linear exponentials to the processed data (with the number of exponentials equal to the number of found components which represent 99% of data—the remaining data being noise) from which the decay rates/time constants and concentration profiles (for a selected interaction model) are extracted. In the processing, a rectangular data matrix (spectra vs. time) was imported into a graphical user interface. The method decomposes a matrix of difference spectra taken at selected times (i.e., experimental delays between pump and probe) into a number of linearly independent components (that represent all meaningful input data) found in the dimensionality reduction. Then with the SVD option, the solutions for the kinetic traces of the entire spectral range are tested against a number range of significant components that reproduce well experimental signals. SVD method reduces dimensionality by fitting a set of significant components to the complete experimental data and also reduces noise in data. Once the number of significant components is identified the time constants and components’ contribution to spectra is computed. The accuracy of the time constant determination benefits from the global fitting of orthogonal components that represent the full experimental amplitudes. Next, those time constants are input as fixed and spectrally shared values (associated amplitudes can vary spectrally though) into the global analysis for fitting the model related generated concentration profiles to all transient spectra resulting in time-independent component resolved spectra (while in SVD the analysis is done for a kinetic (time) trace at a selected wavenumber, in the global analysis a similar fitting is done for imported and fixed SVD constants and for the entire data spectral range simultaneously).

*Nonlinear fitting*. The nonlinear analysis was performed by indirectly fitting the third-order polynomial of excitation carrier density (each order representing the key recombination process) to the modified (to adapt to experimental conditions) recombination rate equation applied in [42]. Since the ratio of generated carrier to absorbed photon densities (quantum yield) is unknown at specific irradiance, the model allows the computation of only products of the third-order decay constant with quantum yield squared and second-order decay constant with quantum yield. However, at lower experimentally applied irradiances, the quantum yield equals one (linear could be used to estimate quantum yields at higher (nonlinear absorption) irradiances. The experimental transmission change utilized in [42] was converted and expressed as the difference absorbance signal measured in our experiment. The injected carrier density was expressed by the estimated absorbed photon number using the pump absorption initial condition [17]. To get the two nonlinear decay rates, the second-order decay constant is multiplied by the estimated injected carrier density and the quantum yield, and the third-order decay constant by carrier density squared and quantum yield squared. The fit was done for an extracted time trace at an arbitrarily chosen wavenumber. The resultant polynomial terms were then fitted to the time derivative of the transient signal using the Lsqcurvefit fitting solver of Matlab. The time constant in the first-order term was taken from SVD since the nonlinear fitting is relatively insensitive to its changes. To overcome the numerical challenge of fitting a relatively small number of the searched third-order decay rate, the MultiStart solver was additionally run to optimize (prior to executing the main fitting solver) a closer range of initial input decay rates for the main solver to better converge.

## Data Availability

Not applicable.

## Supplementary Materials

The following supporting information can be downloaded at: https://www.mdpi.com/article/10.3390/nano12101616/s1. Global analysis of experiments in Figure 3 (Figures S1–S69), FTIR spectra of samples (Figures S70–S72), UV-VIS spectra of samples (Figures S73–S76), extended version of Figure 1 (Figures S77).
