# Supplementary material for: High Power Irradiance Dependence of Charge Species Dynamics in Hybrid Perovskites and Kinetic Evidence for Transient Vibrational Stark Effect in Formamidinium
Source: Nanomaterials (Basel). 2022 May 10;12(10):1616. doi: 10.3390/nano12101616 (PMC9146680; doi:10.3390/nano12101616)
Supplement: Supplementary file 1 [file nanomaterials-12-01616-s001.zip › nanomaterials-1619257-supplementary.pdf]

Supporting Information

High Power Irradiance Dependence of Charge Species Dynamics in Hybrid Perovskites and Kinetic Evidence for Transient Vibrational Stark Effect in Formamidinium

Rafal Rakowski [1], William Fisher [2], Joaquín Calbo [3], Muhamad Z. Mokhtar[4], Xinxing Liang [5], Dong Ding [5], Jarvist M. Frost[2], Saif A. Haque [5], Aron Walsh [3], Piers R. F. Barnes [2], Jenny Nelson [2] and Jasper J. van Thor [1,*]

[1] Life Science Department, Imperial College London, London SW7 2AZ, UK; rafal1rakowski@gmail.com
[2] Department of Physics, Imperial College London, London SW7 2AZ, UK; w.fisher17@imperial.ac.uk (W.F.); jarvist.frost@imperial.ac.uk (J.M.F.); piers.barnes@imperial.ac.uk (P.R.F.B.); jenny.nelson@imperial.ac.uk (J.N.)
[3] Department of Materials, Imperial College London, London SW7 2AZ, UK; joaquin.calbo@uv.es (J.C.); a.walsh@imperial.ac.uk (A.W.)
[4] School of Materials, University of Manchester, MSS Tower, Manchester M13 9PL, UK; engr.hasif@yahoo.com
[5] Department of Chemistry, Centre for Plastic Electronics, Imperial College London, London W12 0BZ, UK; xinxing.liang@imperial.ac.uk (X.L.); dong.ding11@imperial.ac.uk (D.D.); s.a.haque@imperial.ac.uk (S.A.H.)
* Correspondence: j.vanthor@imperial.ac.uk.

Time-resolved charge recombination analysis upon optical excitation of 600 nm thick film of $(FAPbI_3)_{0.97}(MAPbBr_3)_{0.03}$

a)

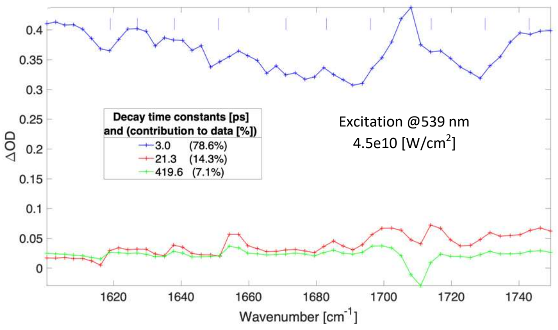

b)

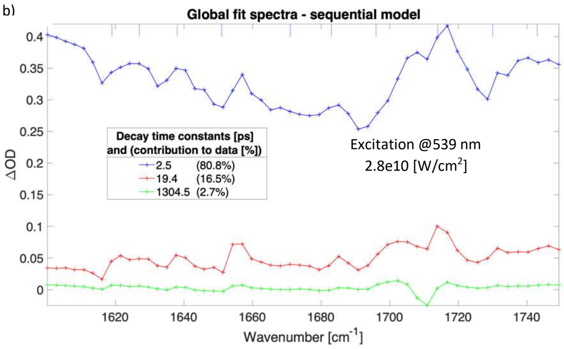

c)

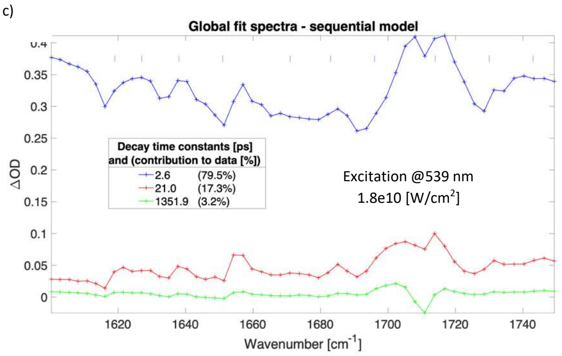

d)

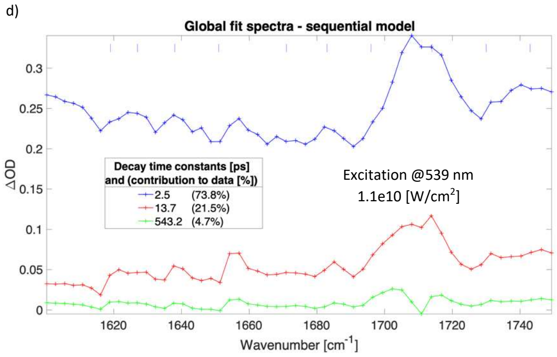

e)

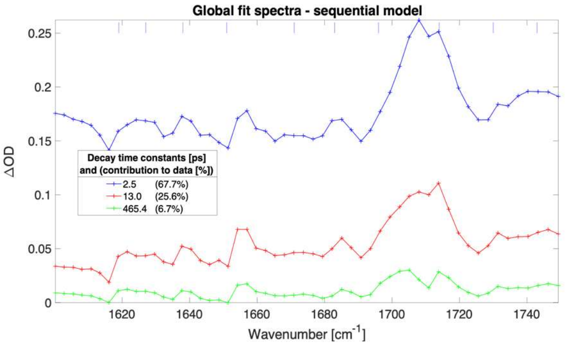

f)

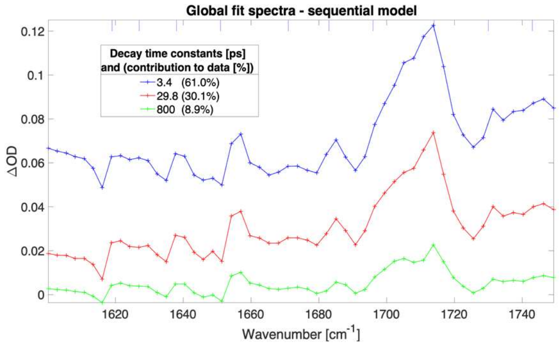

g)

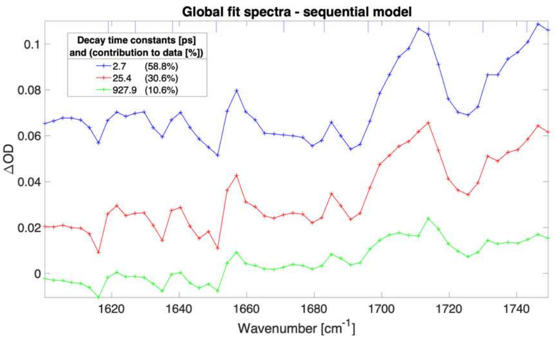

h)

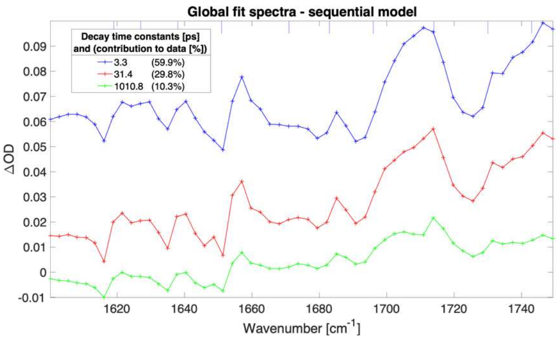

i)

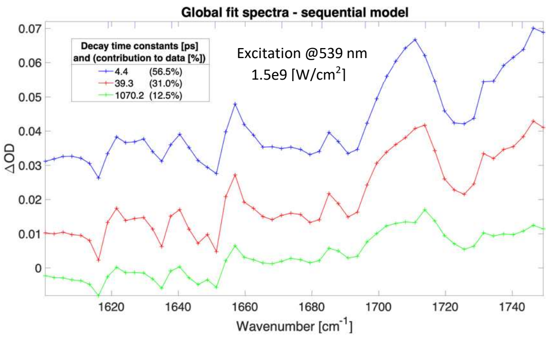

j)

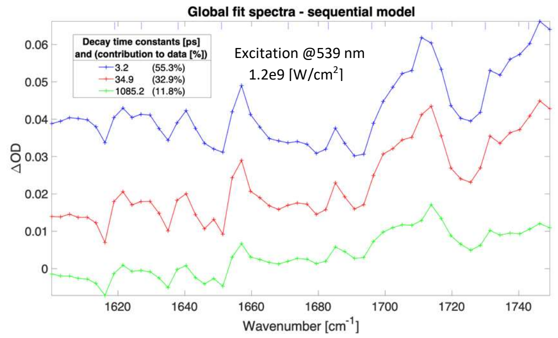

k)

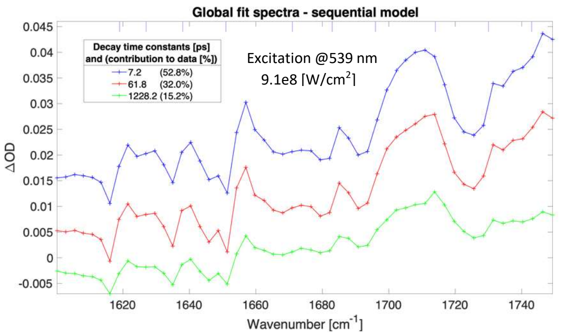

l)

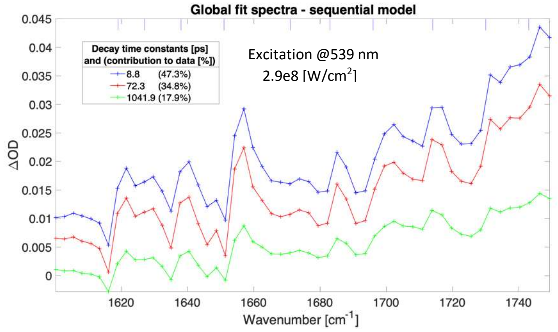

m)

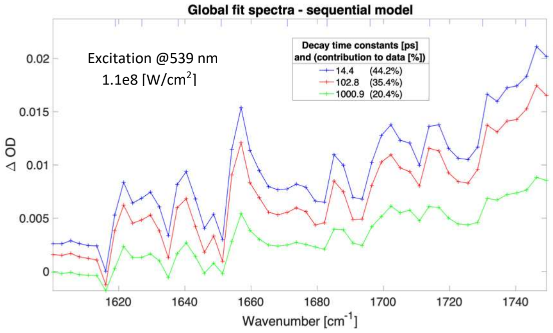

n)

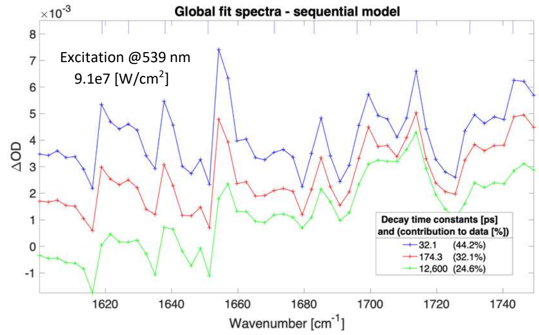

Figure S1. Results of three compartments sequential global fit to data for 600 nm thick film of $(FAPbI_3)_{0.97}(MAPbBr_3)_{0.03}$. The irradiance and SVD time constants; 4.5e10 W/cm^2, 3.0 ps, 21.3 ps, and 419.6 ps (a); 2.8e10 W/cm^2, 2.5 ps, 19.4 ps, and 1304.5 ps (b); 1.8e10 W/cm^2, 2.6 ps, 21.0 ps, and 1351.9 ps (c); 1.1e10 W/cm^2, 2.5 ps, 13.7 ps, and 543.2 ps (d); 7.1e9 W/cm^2, 2.5 ps, 13.0 ps, and 465.4 ps (e); 2.8e9 W/cm^2, 3.4 ps, 29.8 ps, and 800.0 ps (f); 2.3e9 W/cm^2, 2.7 ps, 25.4 ps, and 927.9 ps (g); 1.9e9 W/cm^2, 3.3 ps, 31.4 ps, and 1010.8 ps (h); 1.5e9 W/cm^2, 4.4 ps, 39.4 ps, and 1070.2 ps (i); 1.2e9 W/cm^2, 3.2 ps, 34.9 ps, and 1085.2 ps (j); 9.1e8 W/cm^2, 7.2 ps, 61.8 ps, and 1228.2 ps (k); 2.9e8 W/cm^2, 8.8 ps, 72.3 ps, and 1041.9 ps

(l); 1.1e8 W/cm^2, 14.4 ps, 102.8 ps, and 1000.9 ps (m); 9.1e7 W/cm^2, 32.1 ps, 174.3 ps, and 12,600.0 ps (n).

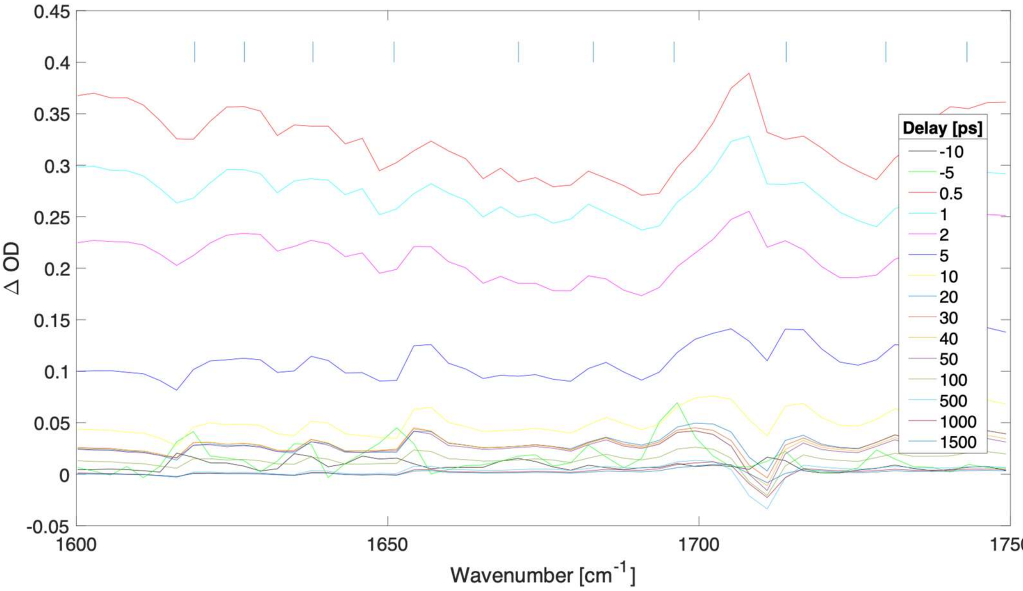

Figure S1.1. Difference transient absorption spectra at selected delays (ps) for a 600 nm thick film of $(FAPbI_3)_{0.97}(MAPbBr_3)_{0.03}$ with pump irradiance of 4.5e10 W/cm^2 (fluence of 11,204.5 uJ/cm^2).

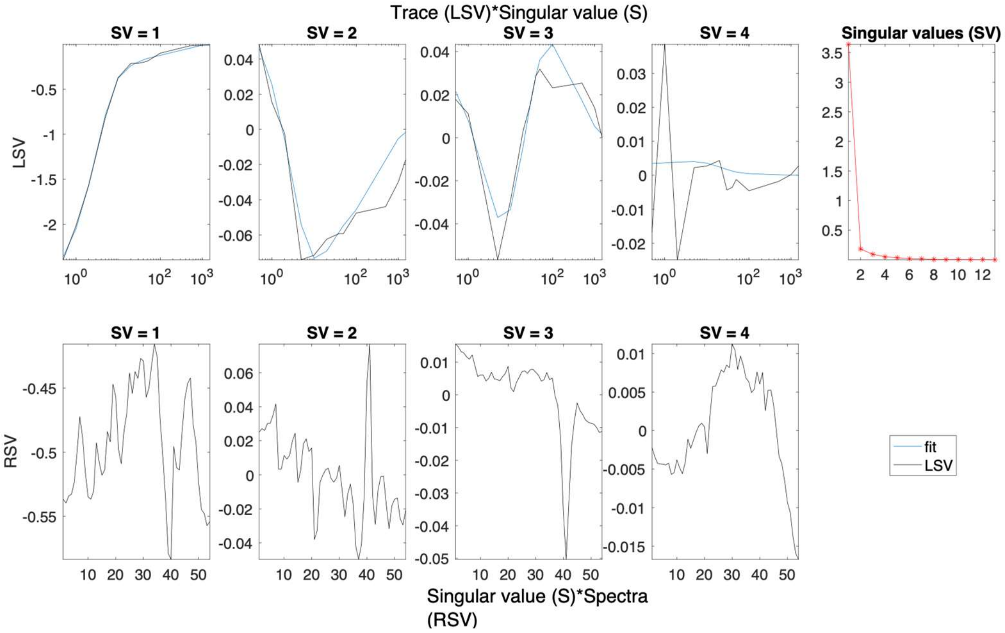

Figure S1.2. Left (LSV) and right (RSV) singular vectors with dominant singular values for the SVD analysis of spectra presented in Figure S1.1.

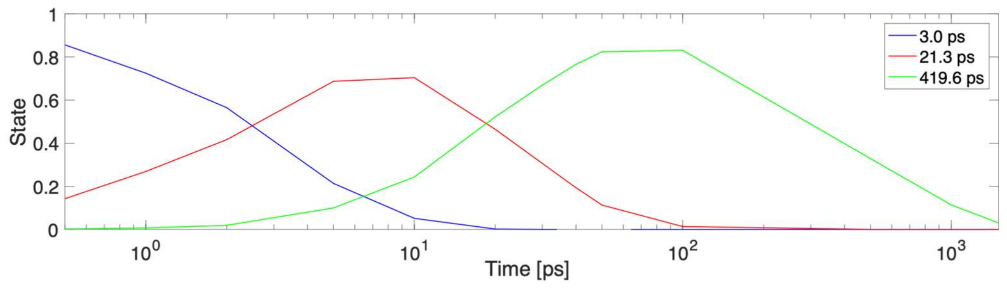

Figure S1.3. Concentration profiles for each time constant fitted to the data presented in Figure S1a.

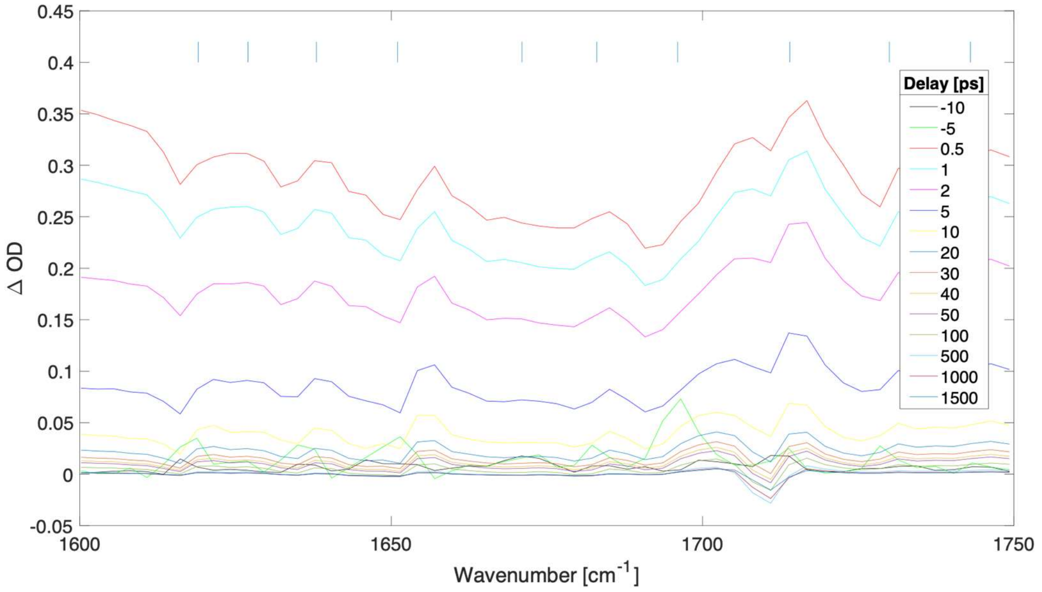

Figure S2.1. Difference transient absorption spectra at selected delays (ps) for a 600 nm thick film of $(FAPbI_3)_{0.97}(MAPbBr_3)_{0.03}$ with pump irradiance of 2.8e10 W/cm^2 (fluence of 7073.6 uJ/cm^2).

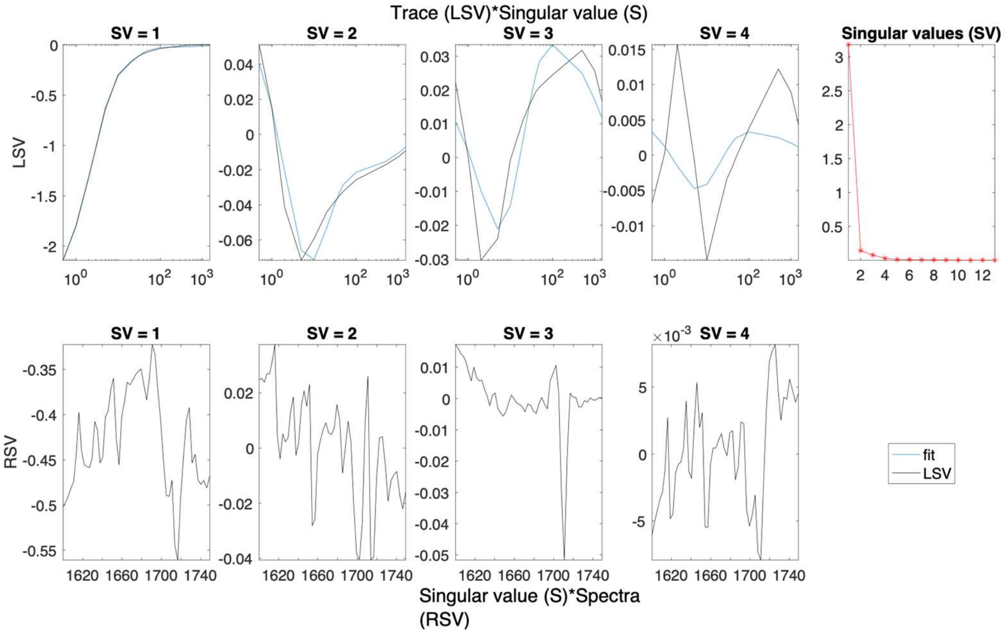

Figure S2.2. Left (LSV) and right (RSV) singular vectors with dominant singular values for the SVD analysis of spectra presented in Figure S2.1.

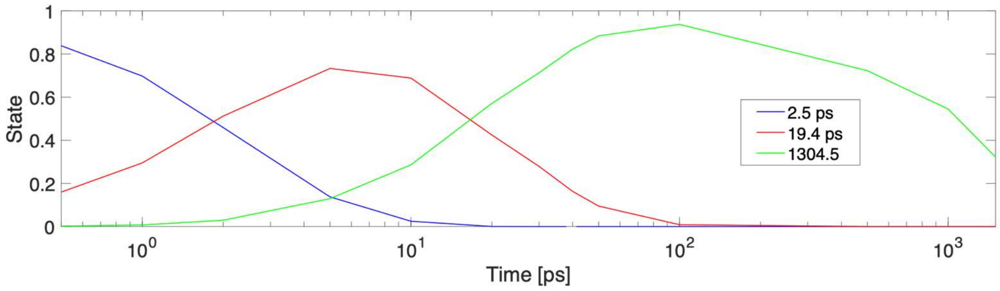

Figure S2.3. Concentration profiles for each time constant fitted to the data presented in Figure S1b.

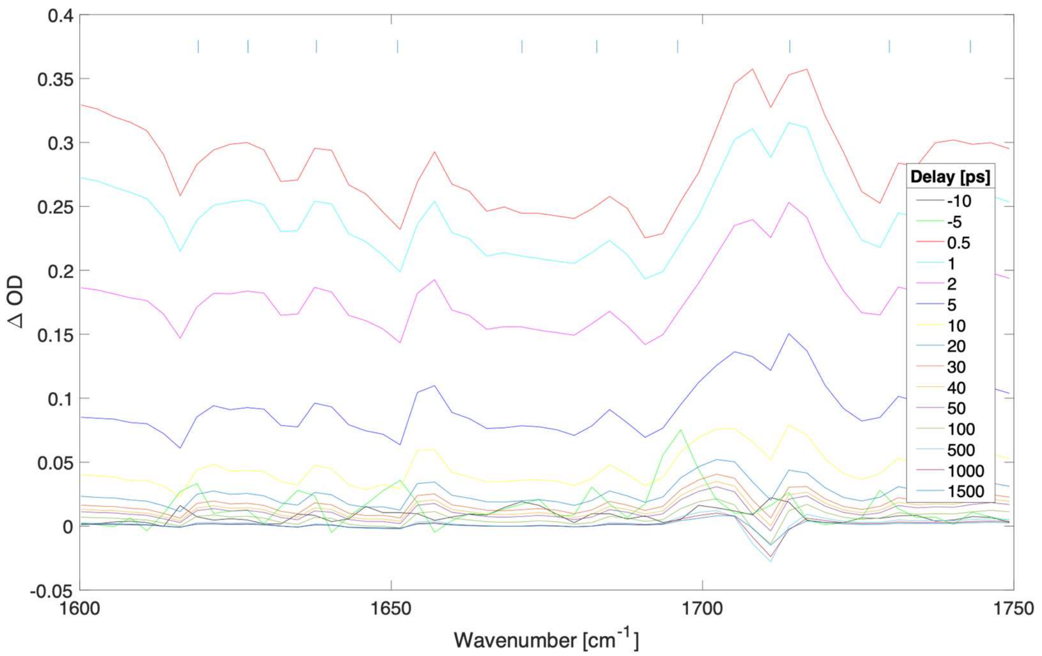

Figure S3.1. Difference transient absorption spectra at selected delays (ps) for a 600 nm thick film of $(FAPbI_3)_{0.97}(MAPbBr_3)_{0.03}$ with pump irradiance of 1.8e10 W/cm^2 (fluence of 4470.5 uJ/cm^2).

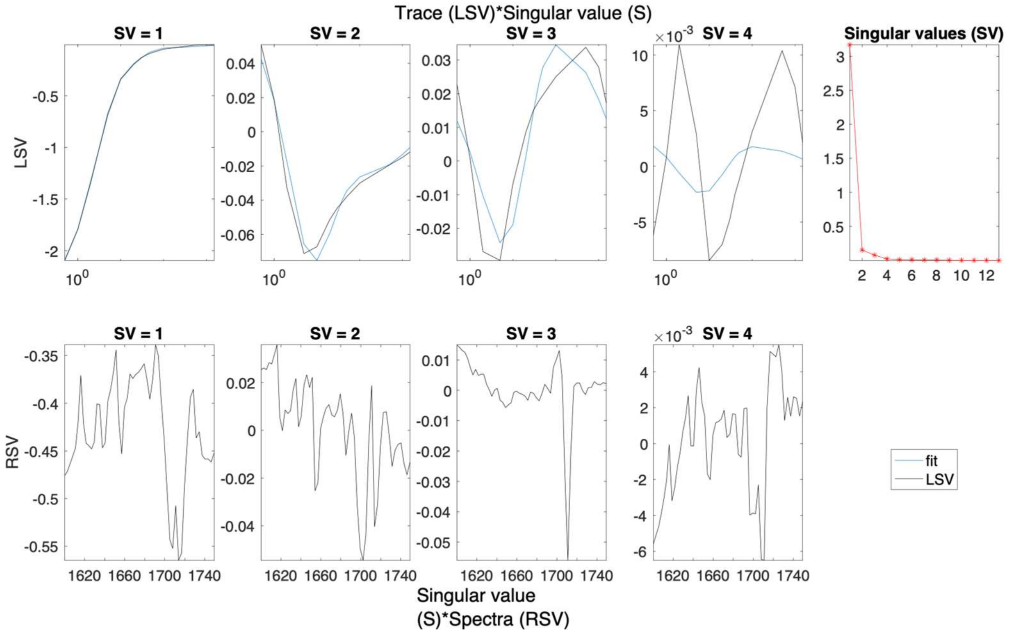

Figure S3.2. Left (LSV) and right (RSV) singular vectors with dominant singular values for the SVD analysis of spectra presented in Figure S3.1.

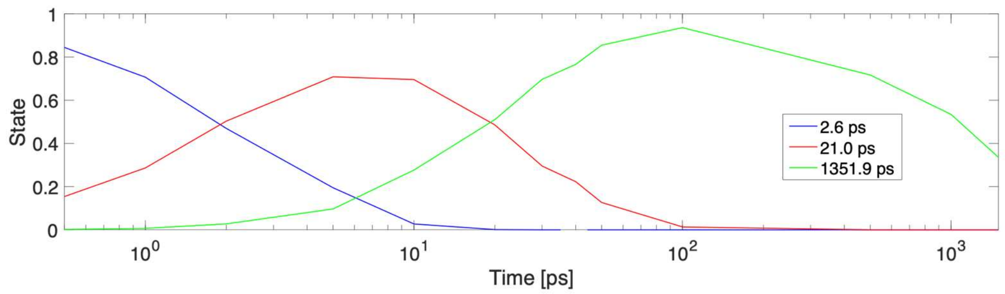

Figure S3.3. Concentration profiles for each time constant fitted to the data presented in Figure S1c.

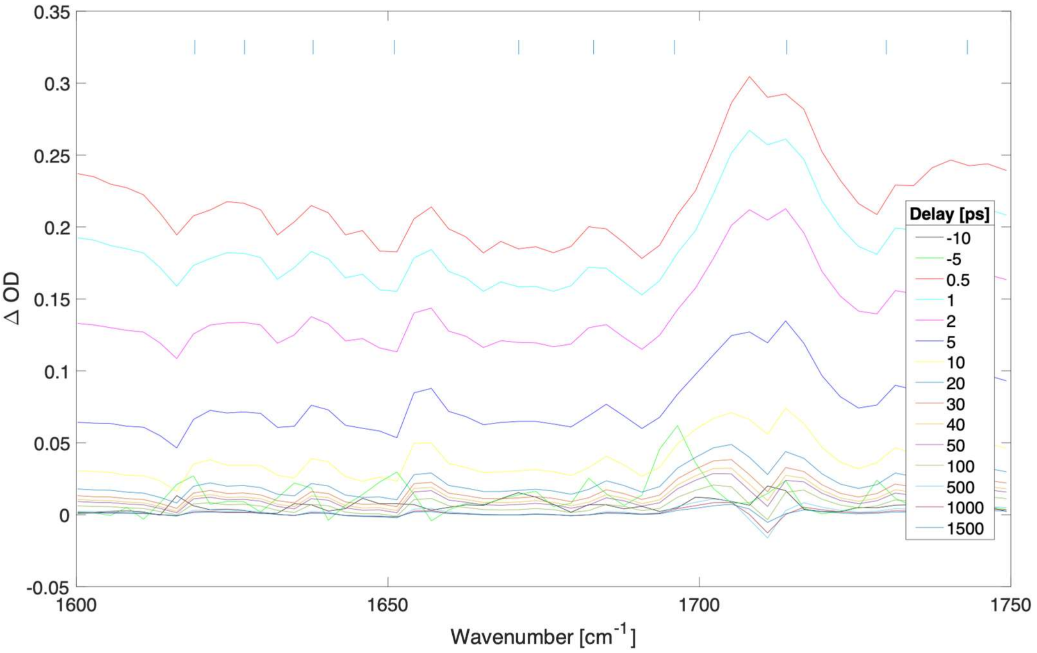

Figure S4.1. Difference transient absorption spectra at selected delays (ps) for a 600 nm thick film of $(FAPbI_3)_{0.97}(MAPbBr_3)_{0.03}$ with pump irradiance of 1.1e10 W/cm^2 (fluence of 2829.4uJ/cm^2).

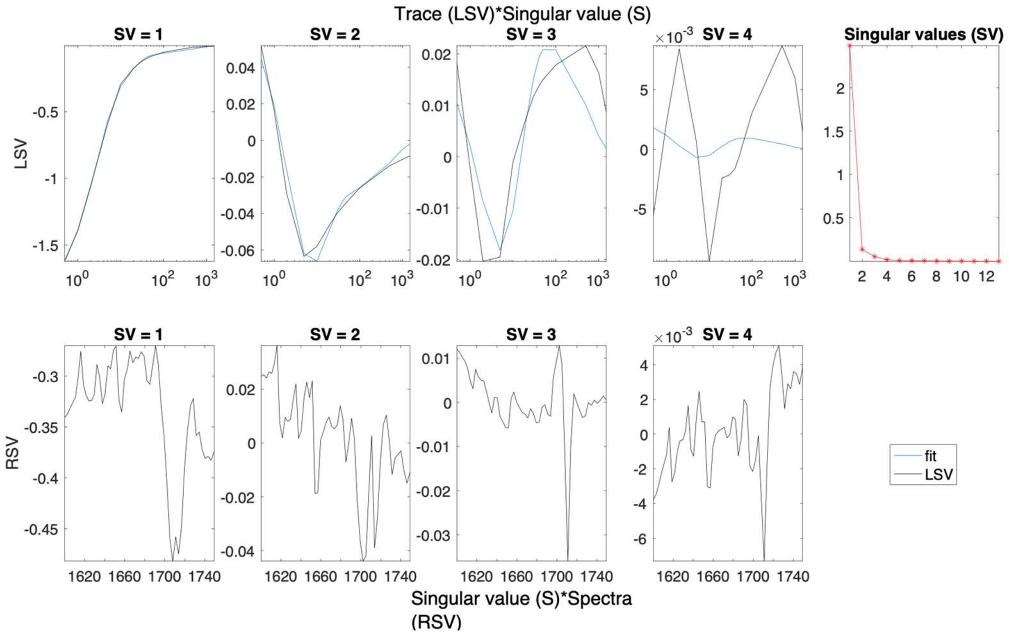

Figure S4.2. Left (LSV) and right (RSV) singular vectors with dominant singular values for the SVD analysis of spectra presented in Figure S4.1.

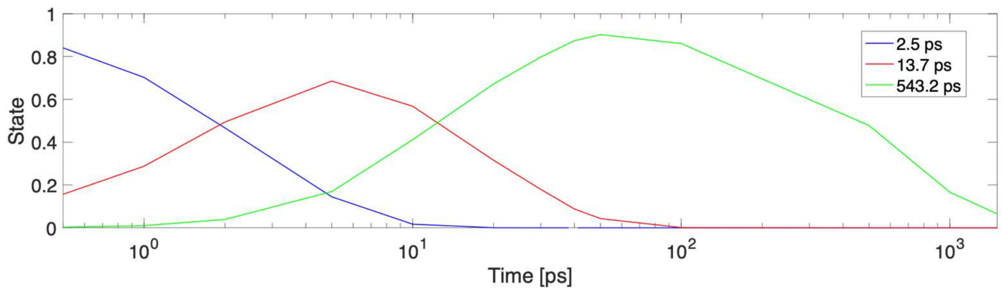

Figure S4.3. Concentration profiles for each time constant fitted to the data presented in Figure S1d.

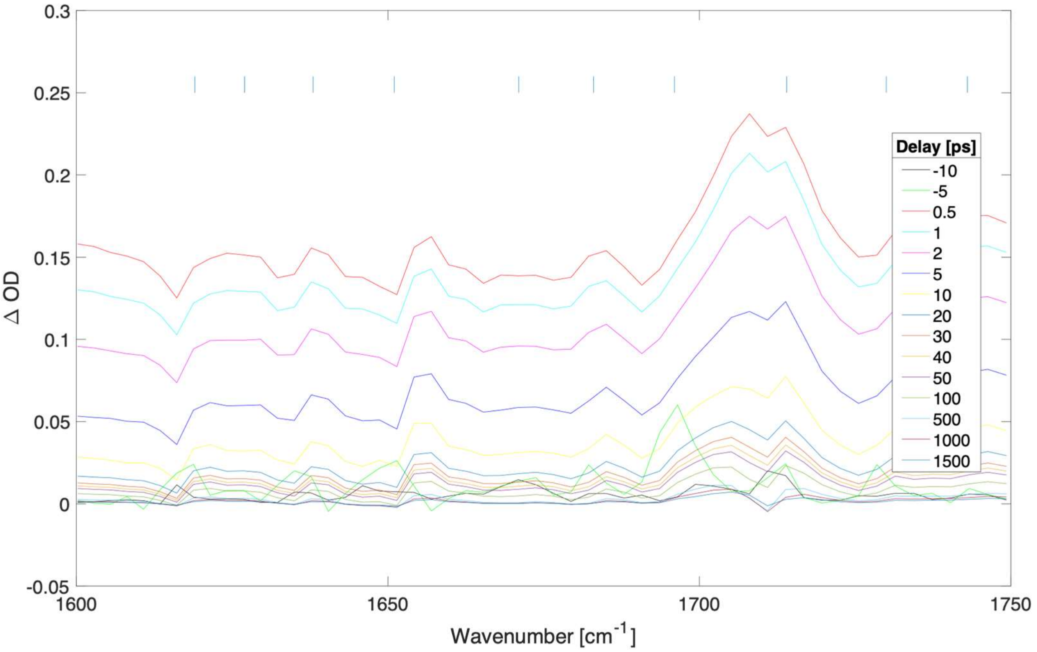

Figure S5.1. Difference transient absorption spectra at selected delays (ps) for a 600 nm thick film of $(FAPbI_3)_{0.97}(MAPbBr_3)_{0.03}$ with pump irradiance of 7.1e9 W/cm^2 (fluence of 1768.4 uJ/cm^2).

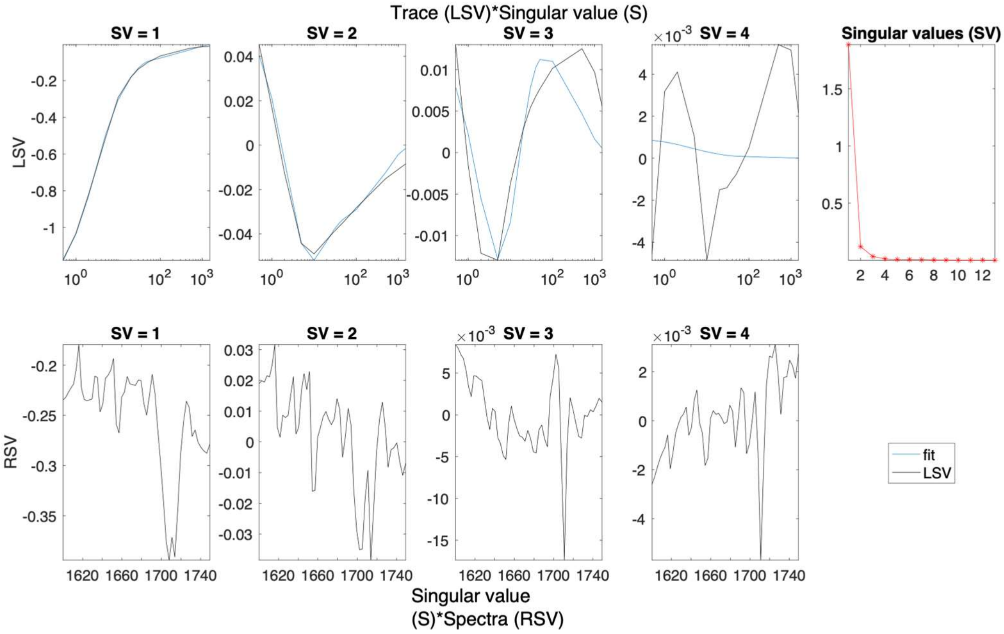

Figure S5.2. Left (LSV) and right (RSV) singular vectors with dominant singular values for the SVD analysis of spectra presented in Figure S5.1.

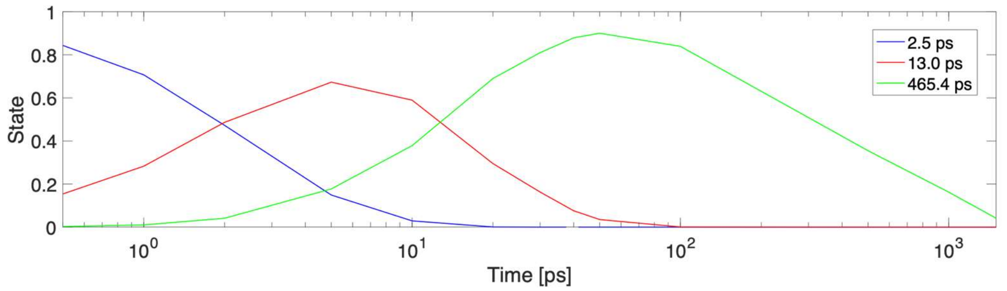

Figure S5.3. Concentration profiles for each time constant fitted to the data presented in Figure S1e.

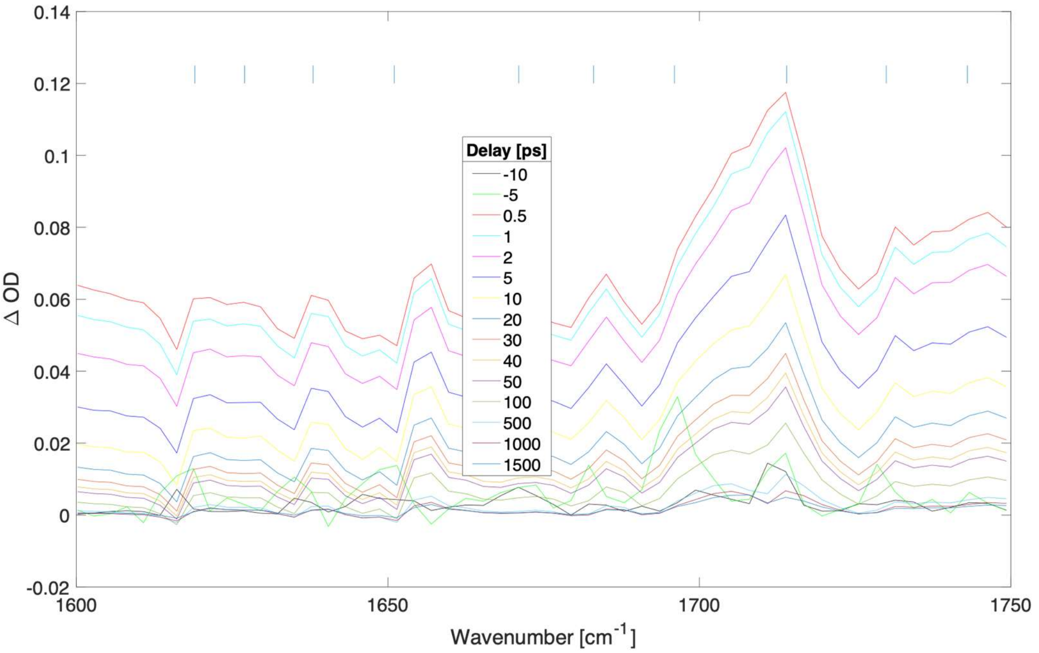

Figure S6.1. Difference transient absorption spectra at selected delays (ps) for a 600 nm thick film of $(FAPbI_3)_{0.97}(MAPbBr_3)_{0.03}$ with pump irradiance of 2.8e9 W/cm^2 (fluence of 707.4 uJ/cm^2).

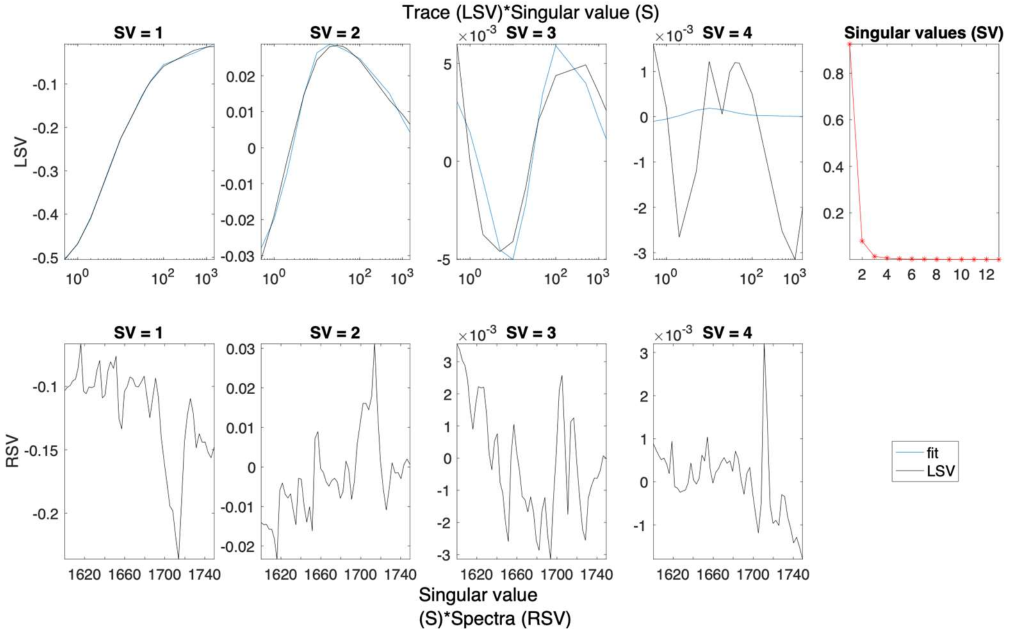

Figure S6.2. Left (LSV) and right (RSV) singular vectors with dominant singular values for the SVD analysis of spectra presented in Figure S6.1.

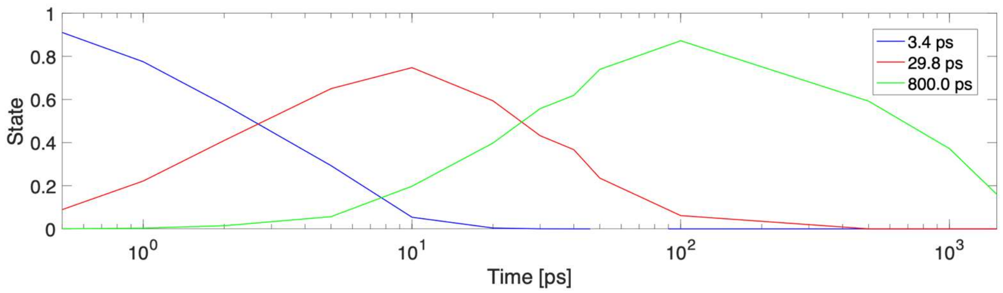

Figure S6.3. Concentration profiles for each time constant fitted to the data presented in Figure S1f.

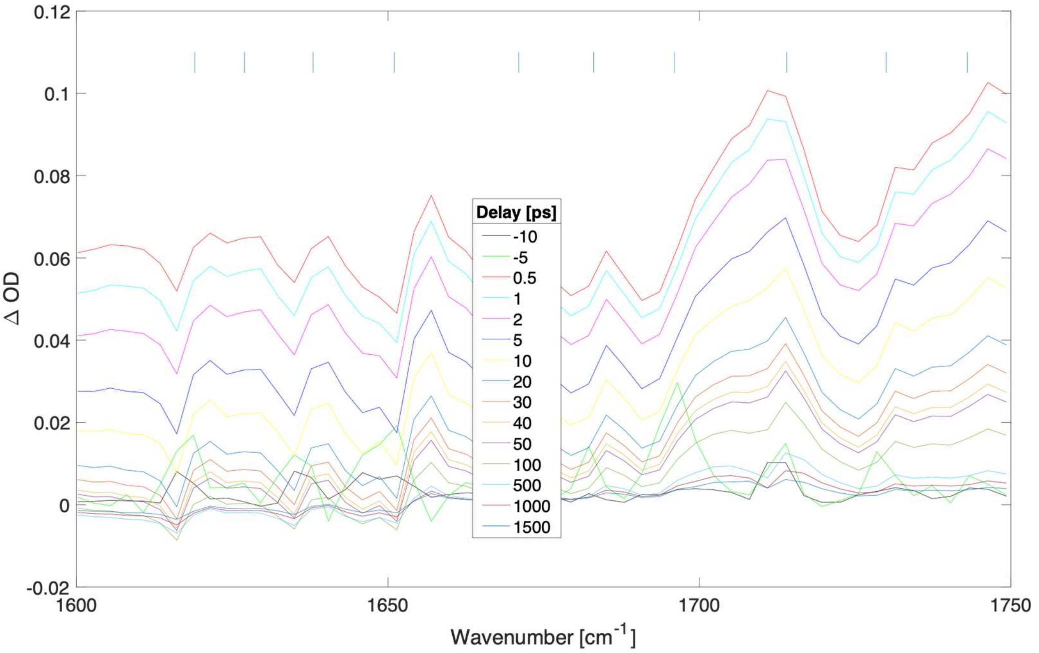

Figure S7.1. Difference transient absorption spectra at selected delays (ps) for a 600 nm thick film of $(FAPbI_3)_{0.97}(MAPbBr_3)_{0.03}$ with pump irradiance of 2.3e9 W/cm^2 (fluence of 580.0 uJ/cm^2).

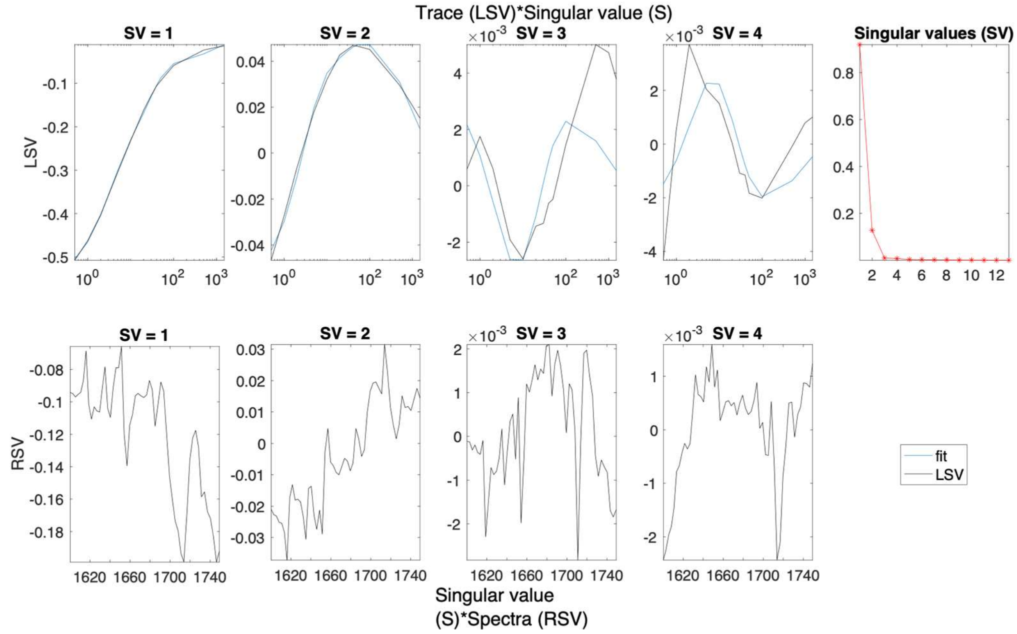

Figure S7.2. Left (LSV) and right (RSV) singular vectors with dominant singular values for the SVD analysis of spectra presented in Figure S7.1.).

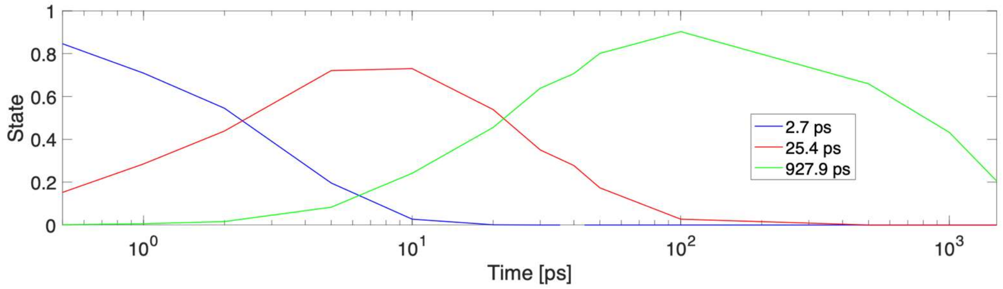

Figure S7.3. Concentration profiles for each time constant fitted to the data presented in Figure S1g.

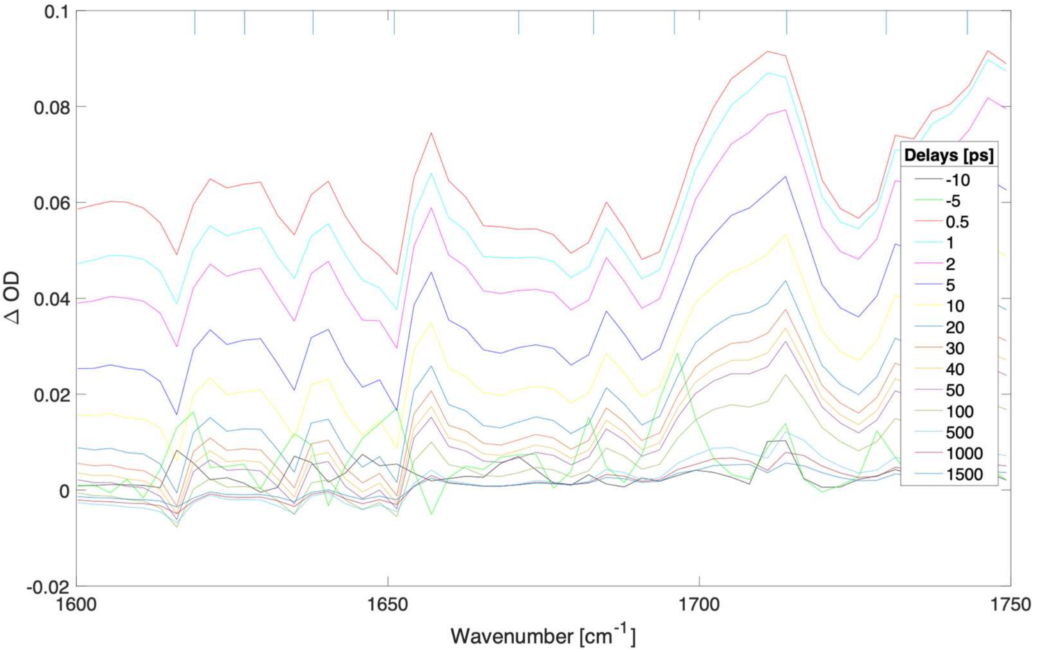

Figure S8.1. Difference transient absorption spectra at selected delays (ps) for a 600 nm thick film of $(FAPbI_3)_{0.97}(MAPbBr_3)_{0.03}$ with pump irradiance of 1.9e9 W/cm^2 (fluence of 464.0 uJ/cm^2).

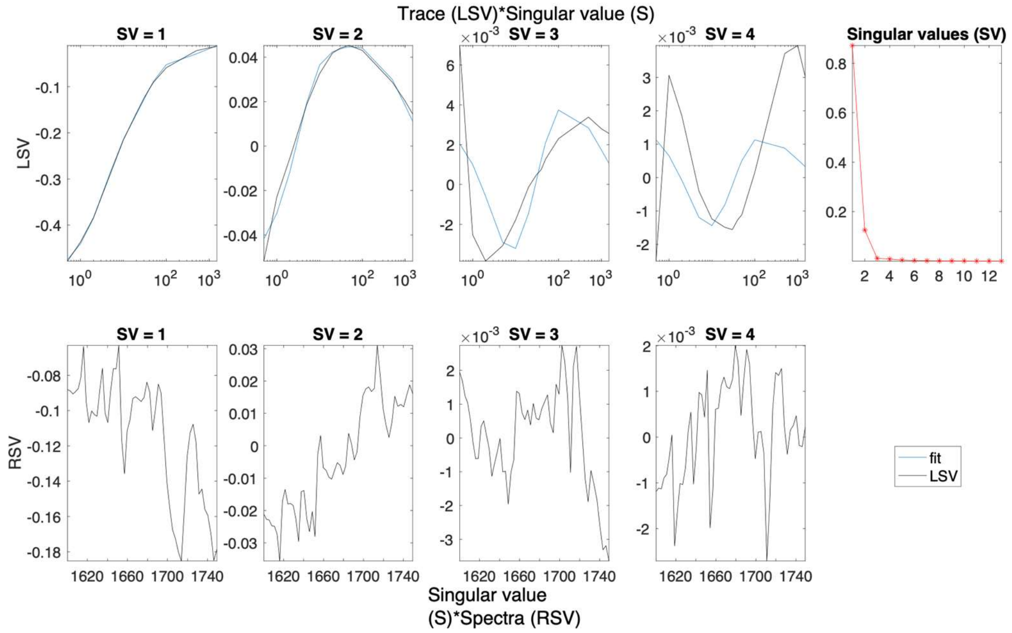

Figure S8.2. Left (LSV) and right (RSV) singular vectors with dominant singular values for the SVD analysis of spectra presented in Figure S8.1.

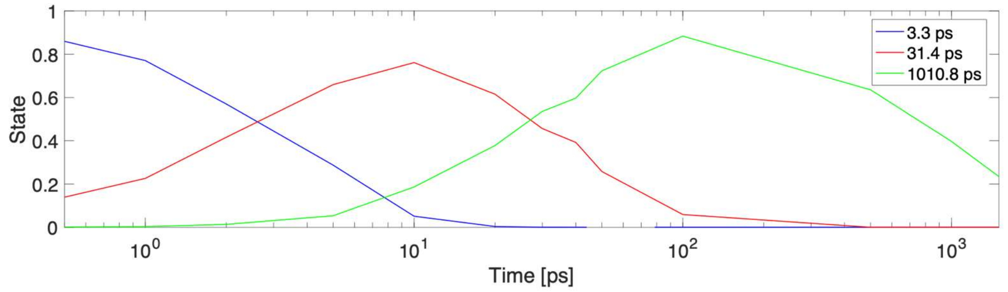

Figure S8.3. Concentration profiles for each time constant fitted to the data presented in Figure S1h.

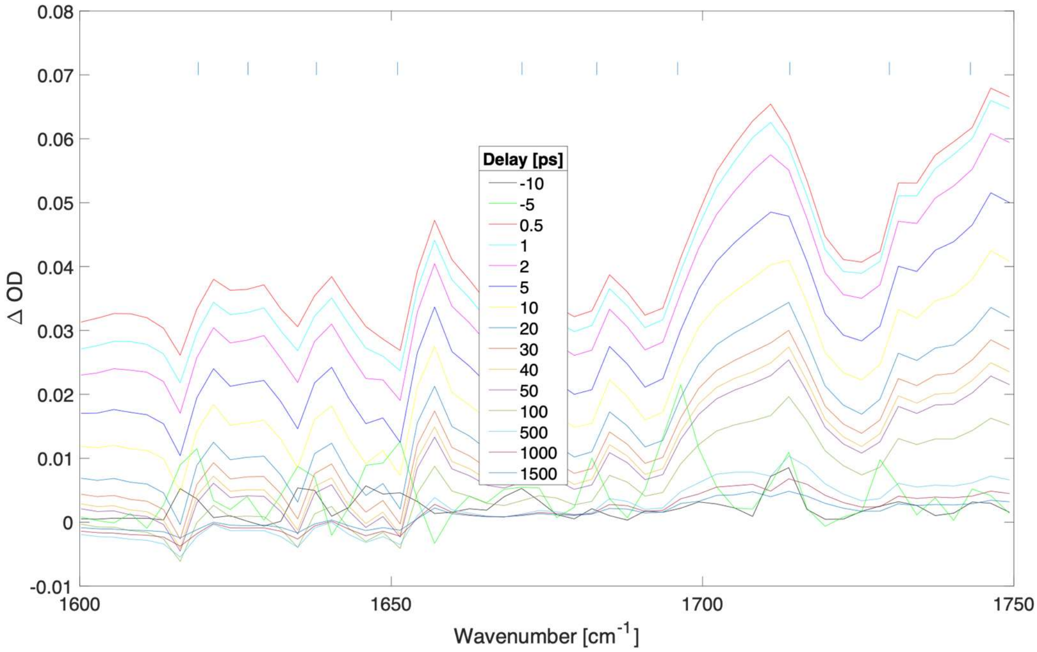

Figure S9.1. Difference transient absorption spectra at selected delays (ps) for a 600 nm thick film of $(FAPbI_3)_{0.97}(MAPbBr_3)_{0.03}$ with pump irradiance of 1.5e9 W/cm^2 (fluence of 367.8 uJ/cm^2).

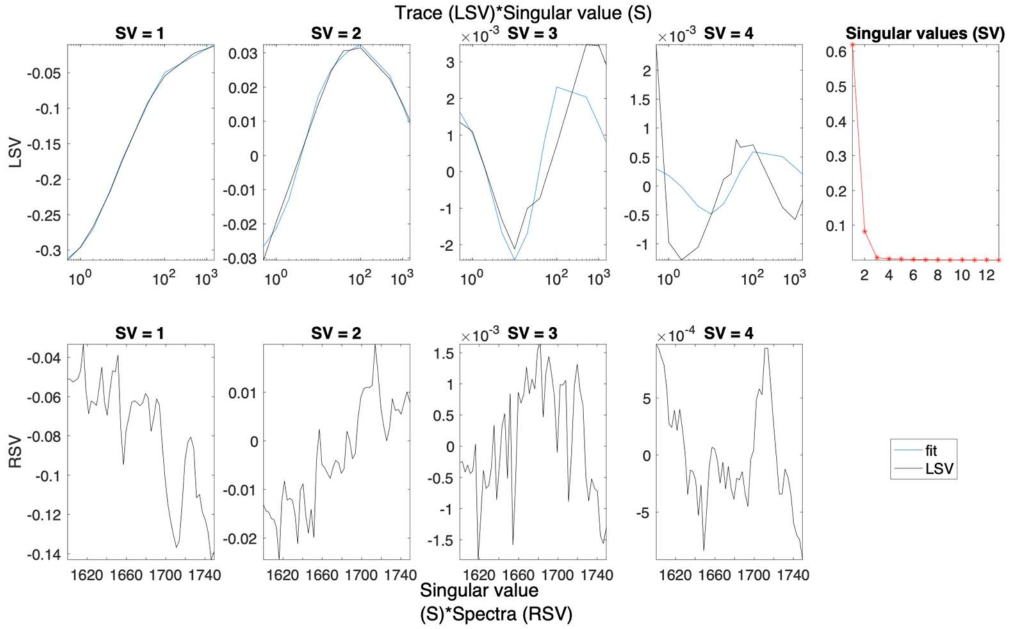

Figure S9.2. Left (LSV) and right (RSV) singular vectors with dominant singular values for the SVD analysis of spectra presented in Figure S9.1.

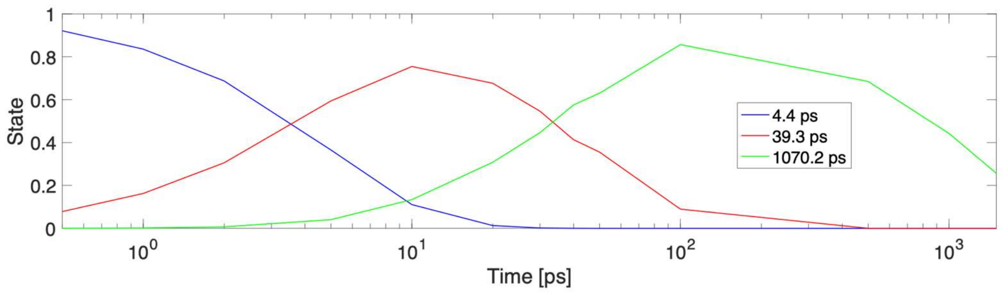

Figure S9.3. Concentration profiles for each time constant fitted to the data presented in Figure S1i.

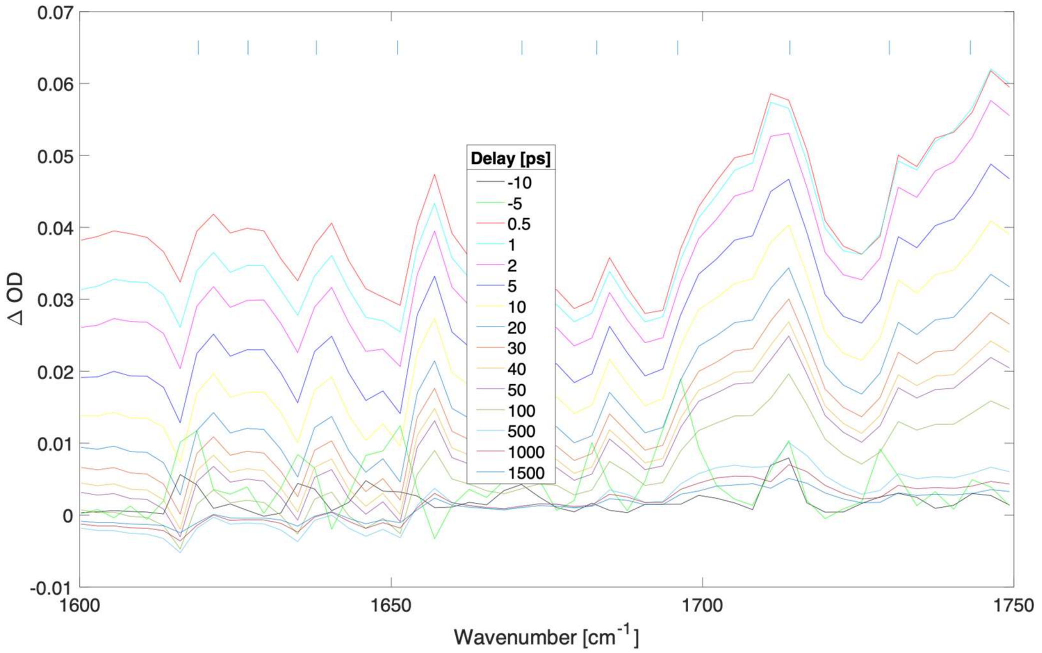

Figure S10.1. Difference transient absorption spectra at selected delays (ps) for a 600 nm thick film of $(FAPbI_3)_{0.97}(MAPbBr_3)_{0.03}$ with pump irradiance of 1.2e9 W/cm^2 (fluence of 294.2 uJ/cm^2).

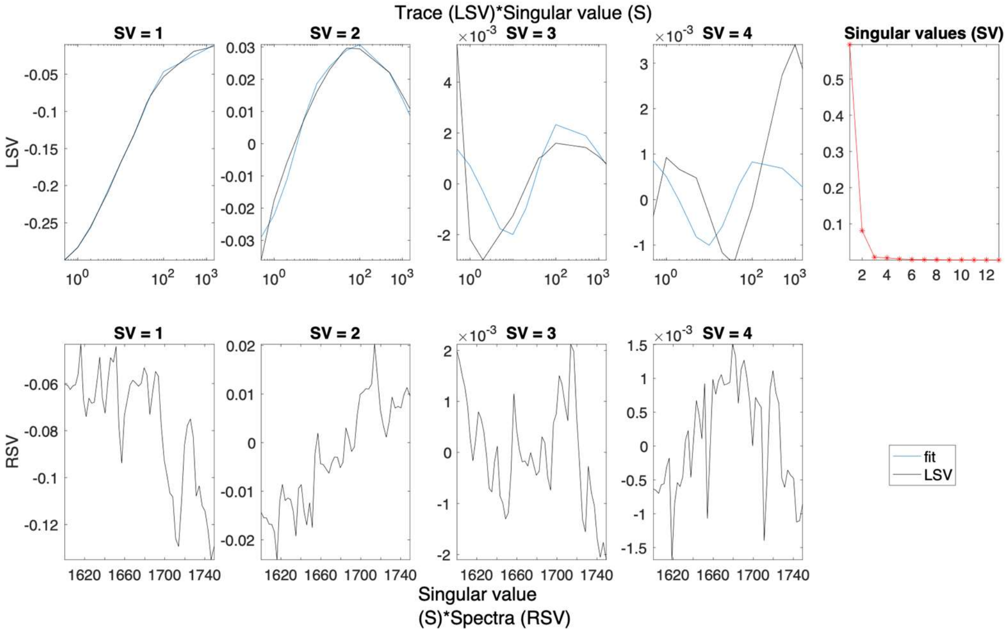

Figure S10.2. Left (LSV) and right (RSV) singular vectors with dominant singular values for the SVD analysis of spectra presented in Figure S10.1.

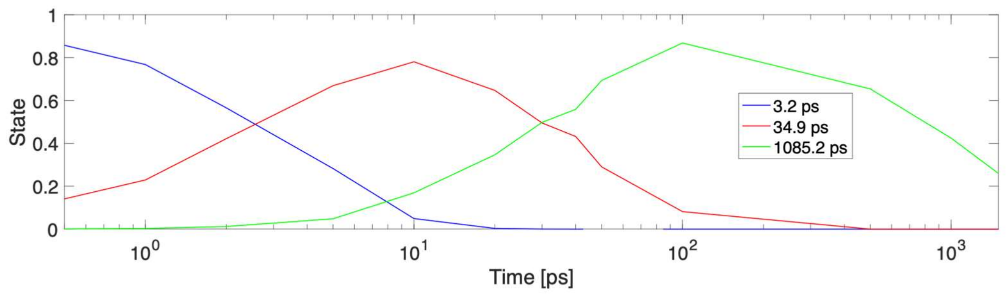

Figure S10.3. Concentration profiles for each time constant fitted to the data presented in Figure S1j.

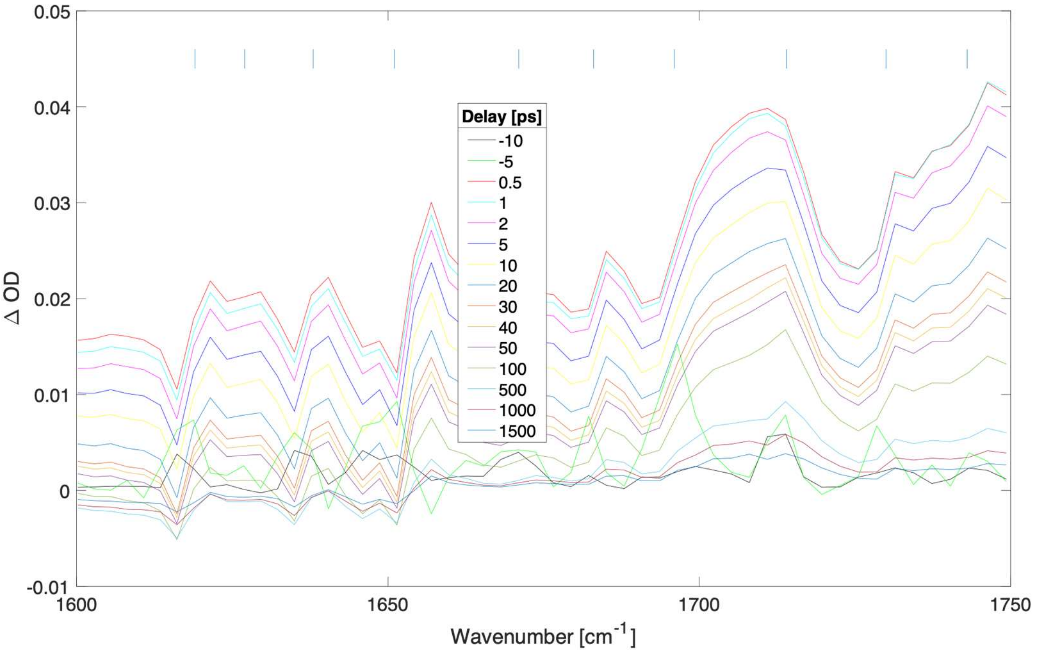

Figure S11.1. Difference transient absorption spectra at selected delays (ps) for a 600 nm thick film of $(FAPbI_3)_{0.97}(MAPbBr_3)_{0.03}$ with pump irradiance of 9.1e8 W/cm^2 (fluence of 226.4 uJ/cm^2).

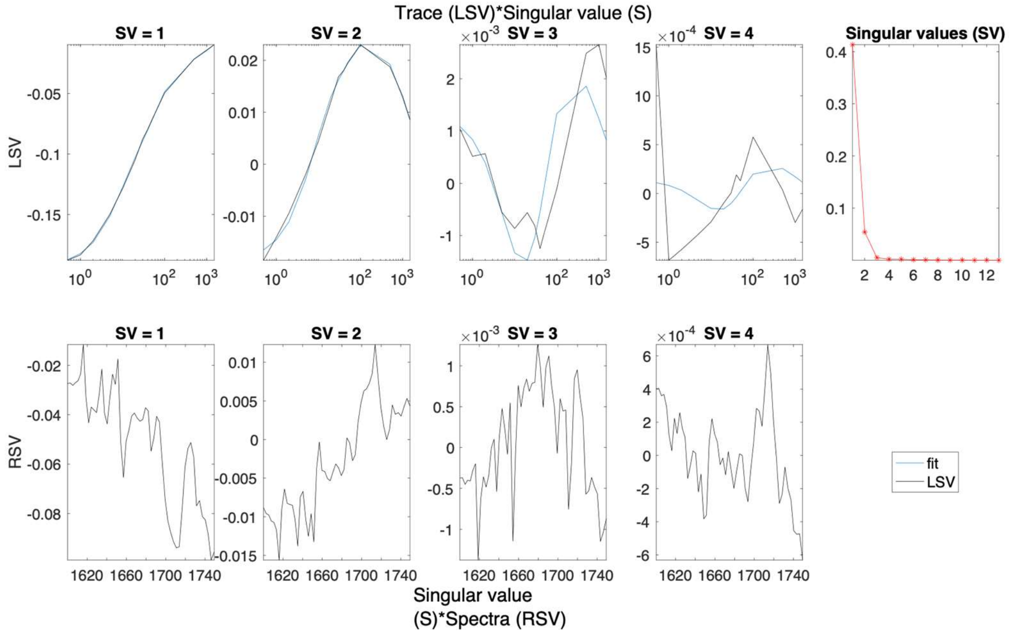

Figure S11.2. Left (LSV) and right (RSV) singular vectors with dominant singular values for the SVD analysis of spectra presented in Figure S11.1.

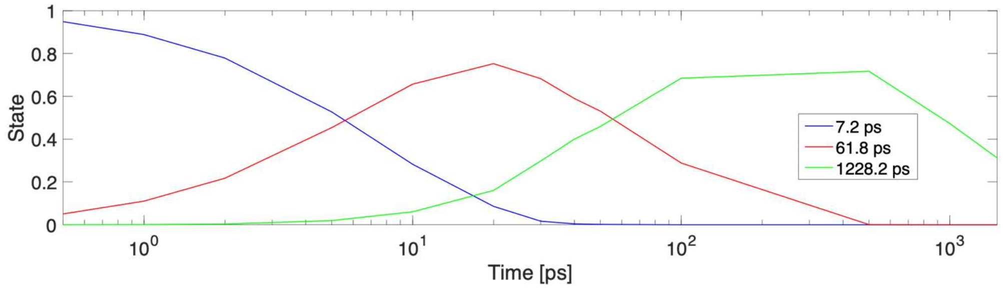

Figure S11.3. Concentration profiles for each time constant fitted to the data presented in Figure S1k.

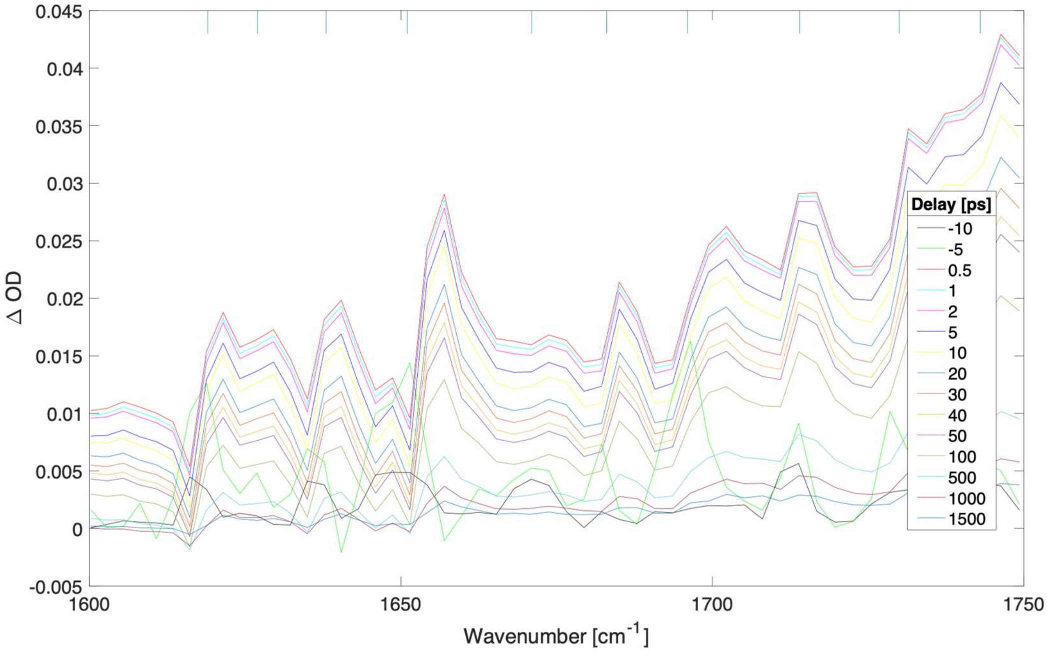

Figure S12.1. Difference transient absorption spectra at selected delays (ps) for a 600 nm thick film of $(FAPbI_3)_{0.97}(MAPbBr_3)_{0.03}$ with pump irradiance of 2.9e8 W/cm^2 (fluence of 73.6 uJ/cm^2).

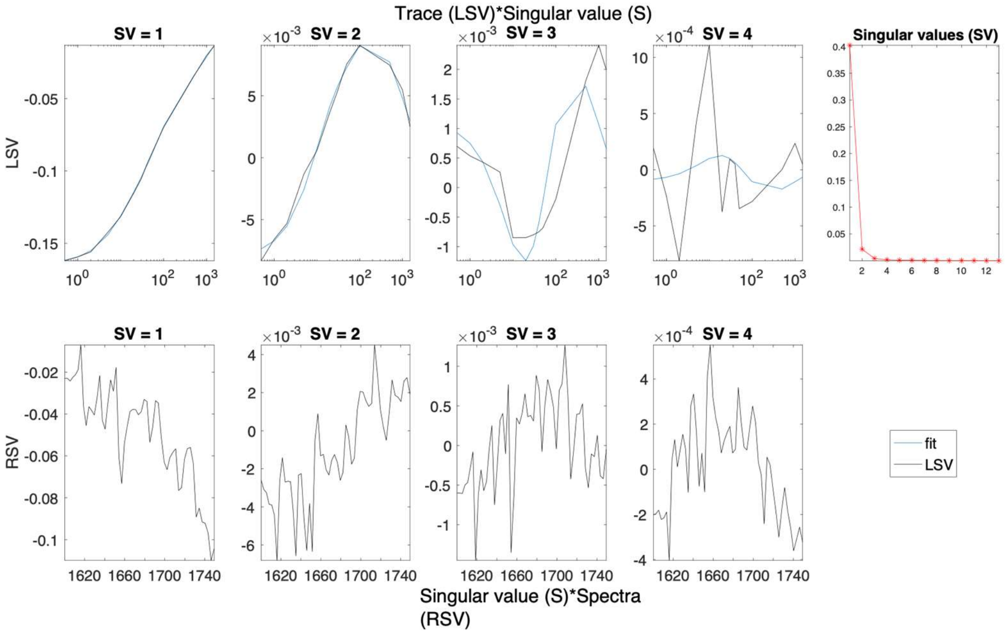

Figure S12.2. Left (LSV) and right (RSV) singular vectors with dominant singular values for the SVD analysis of spectra presented in Figure S12.1.

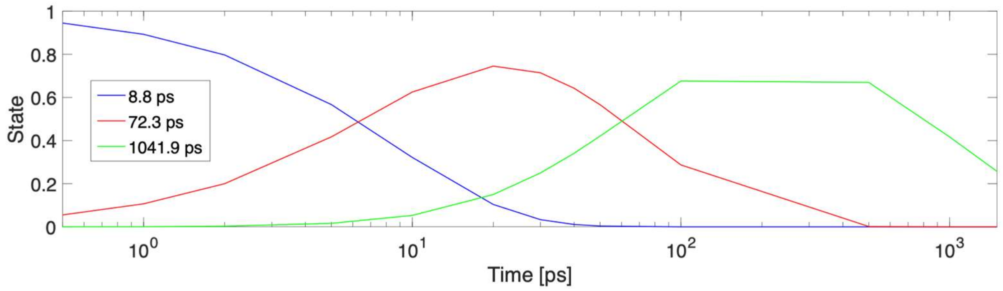

Figure S12.3. Concentration profiles for each time constant fitted to the data presented in Figure S1l.

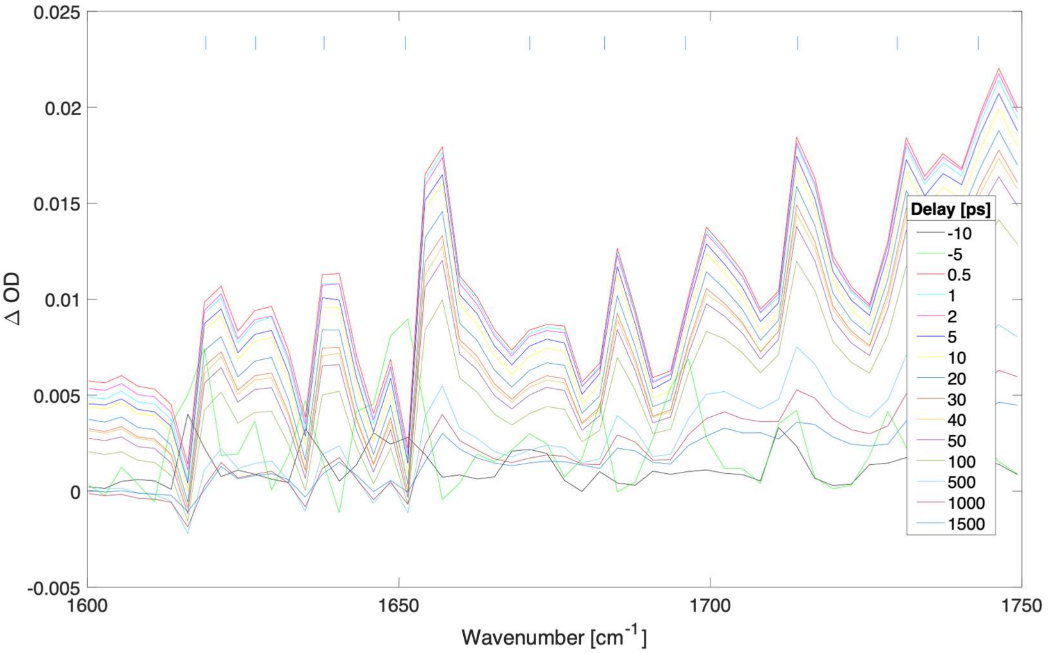

Figure S13.1. Difference transient absorption spectra at selected delays (ps) for a 600 nm thick film of $(FAPbI_3)_{0.97}(MAPbBr_3)_{0.03}$ with pump irradiance of 1.1e8 W/cm^2 (fluence of 28.3 uJ/cm^2).

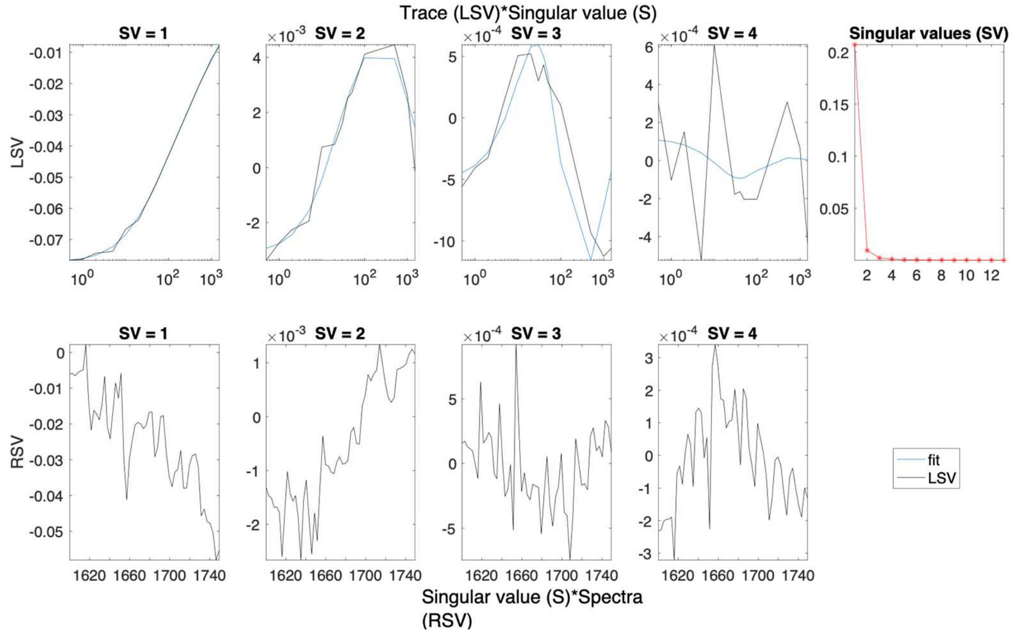

Figure S13.2. Left (LSV) and right (RSV) singular vectors with dominant singular values for the SVD analysis of spectra presented in Figure S13.1.

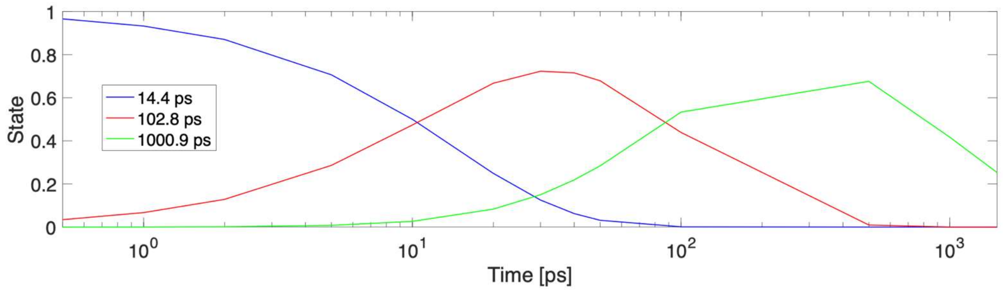

Figure S13.3. Concentration profiles for each time constant fitted to the data presented in Figure S1m.

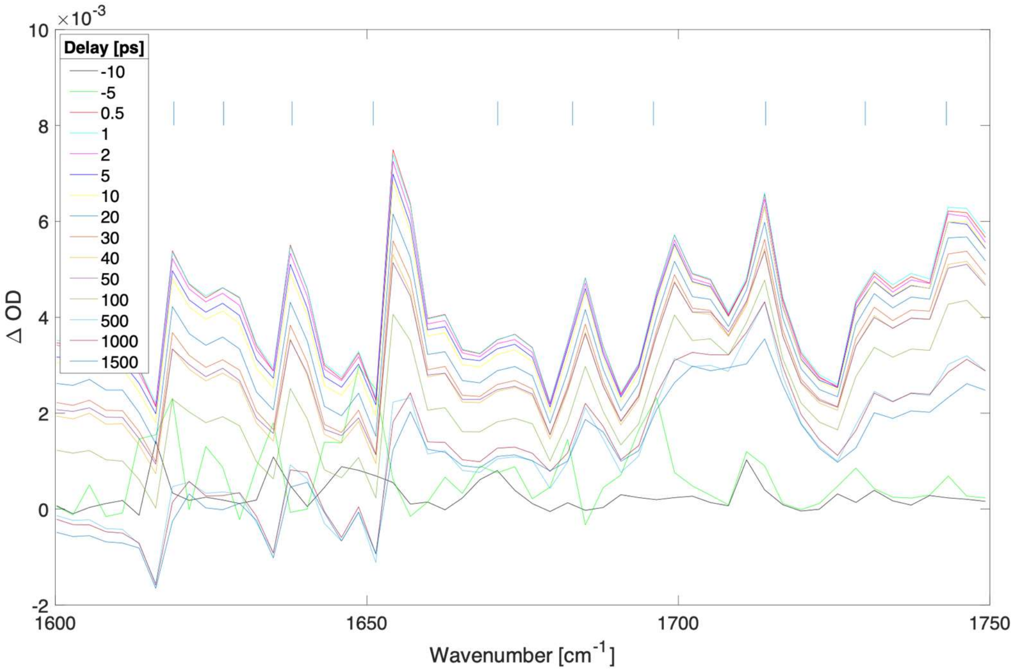

Figure S14.1. Difference transient absorption spectra at selected delays (ps) for a 600 nm thick film of $(FAPbI_3)_{0.97}(MAPbBr_3)_{0.03}$ with pump irradiance of 9.1e7 W/cm^2 (fluence of 22.6 uJ/cm^2).

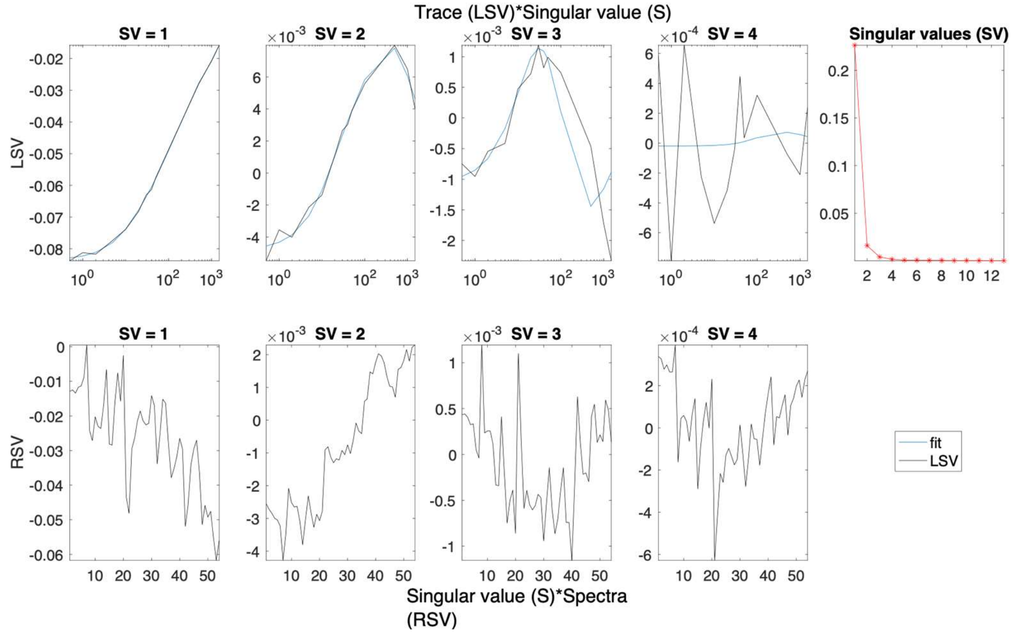

Figure S14.2. Left (LSV) and right (RSV) singular vectors with dominant singular values for the SVD analysis of spectra presented in Figure S14.1.

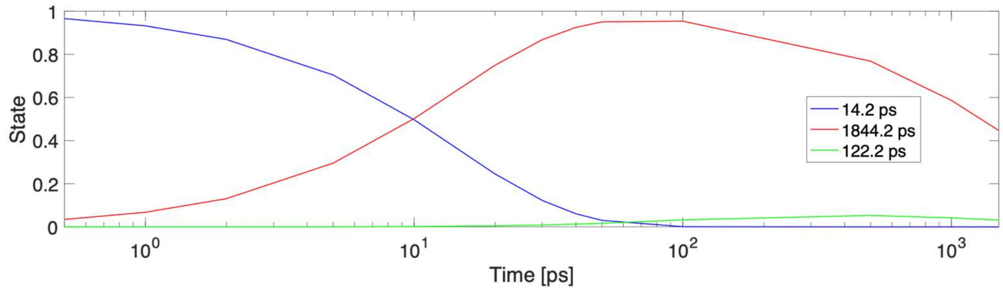

Figure S14.3. Concentration profiles for each time constant fitted to the data presented in Figure S1n.

Time-resolved charge recombination analysis upon optical excitation of 300 nm thick film of $MAPbI_3$

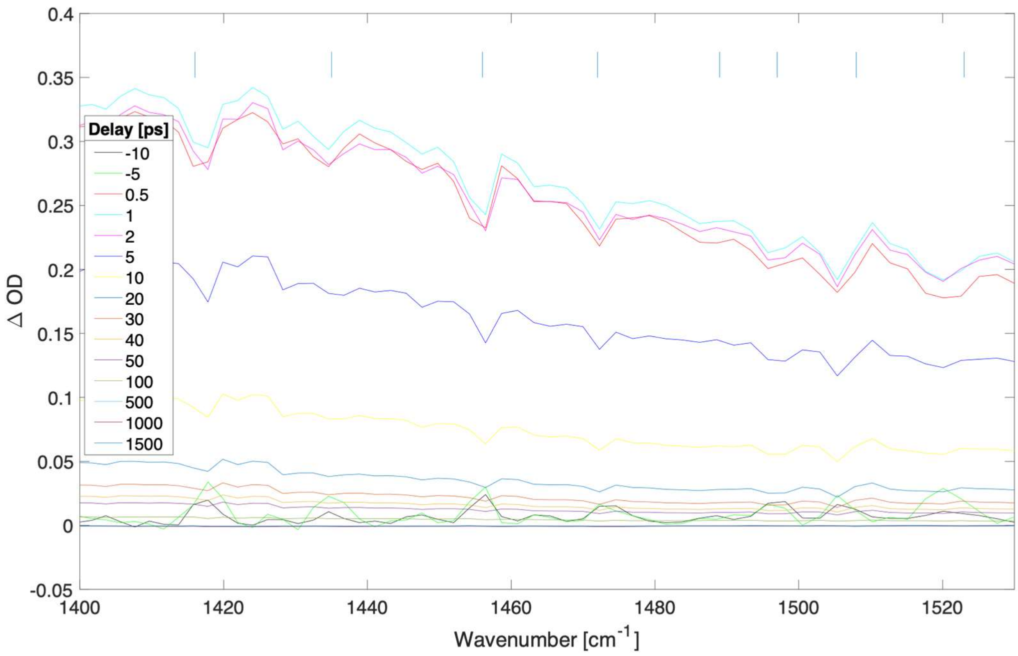

Figure S15.1. Difference transient absorption spectra at selected delays (ps) for a 300 nm thick film of $MAPbI_3$ with pump irradiance of 7.11e10 W/cm^2 (fluence of 17,768.8 uJ/cm^2) at 539 nm.

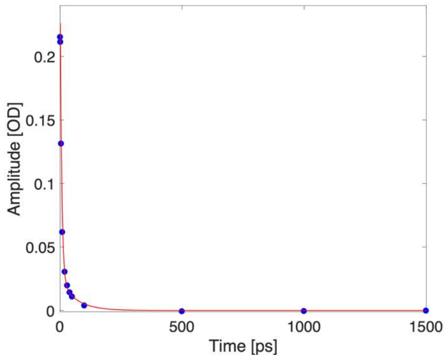

Figure S15.2. Kinetic trace taken at 1470 cm^{-1} (blue dots depict experimental data for selected delays, red curve represents a biexponential fit).

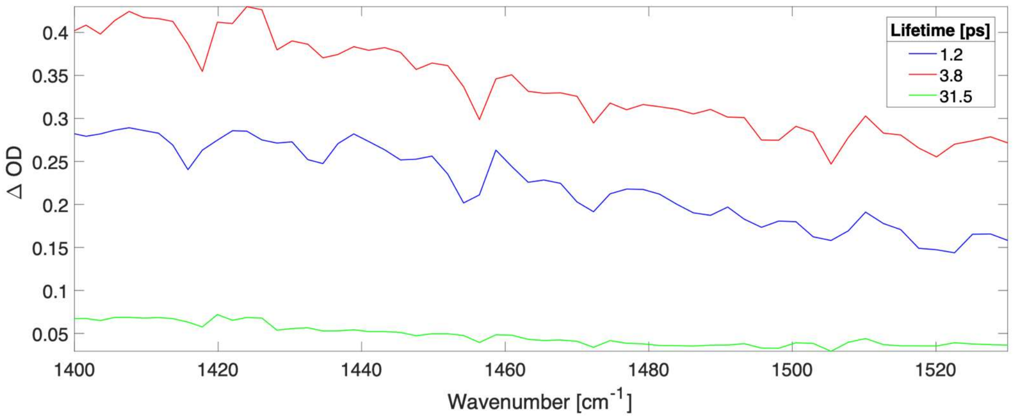

Figure S15.3. Time independent spectra of sequential three compartments global fit to spectra shown in Figure S15.1. The time constants (and contribution to data) were: 3.8 ps (55.7%), 1.2 ps (35.2%), and 31.5 ps (9.1%).

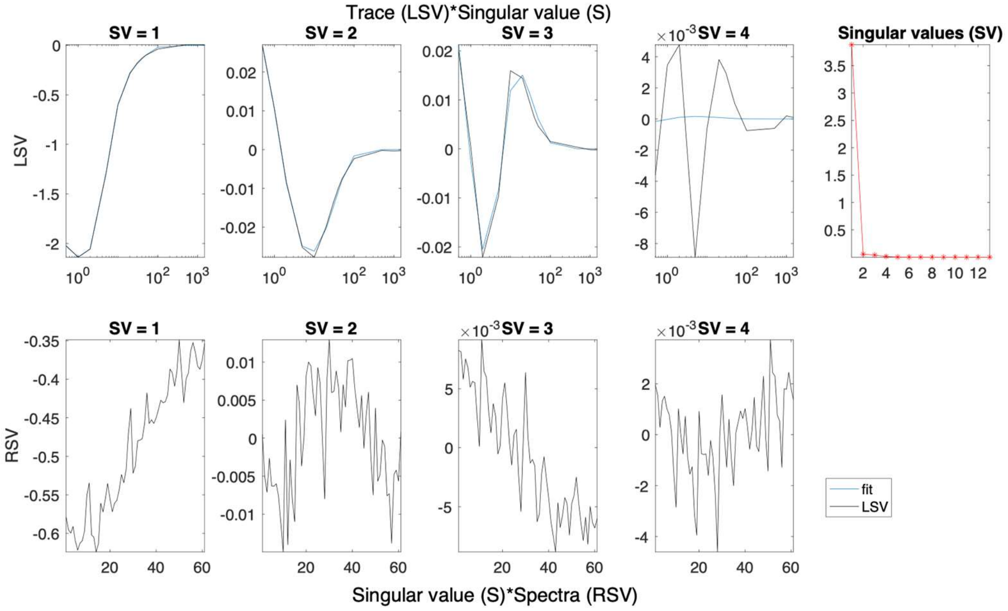

Figure S15.4. Left (LSV) and right (RSV) singular vectors with dominant singular values for the SVD analysis of spectra presented in Figure S15.1.

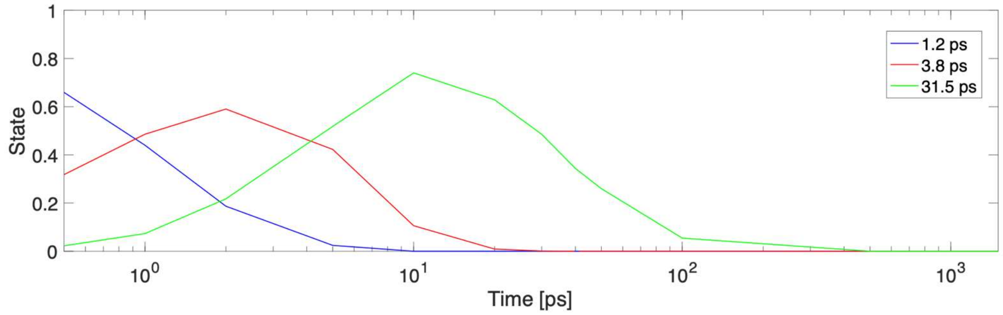

Figure S15.5. Concentration profiles for each time constant fitted to the data in Figure S15.3.

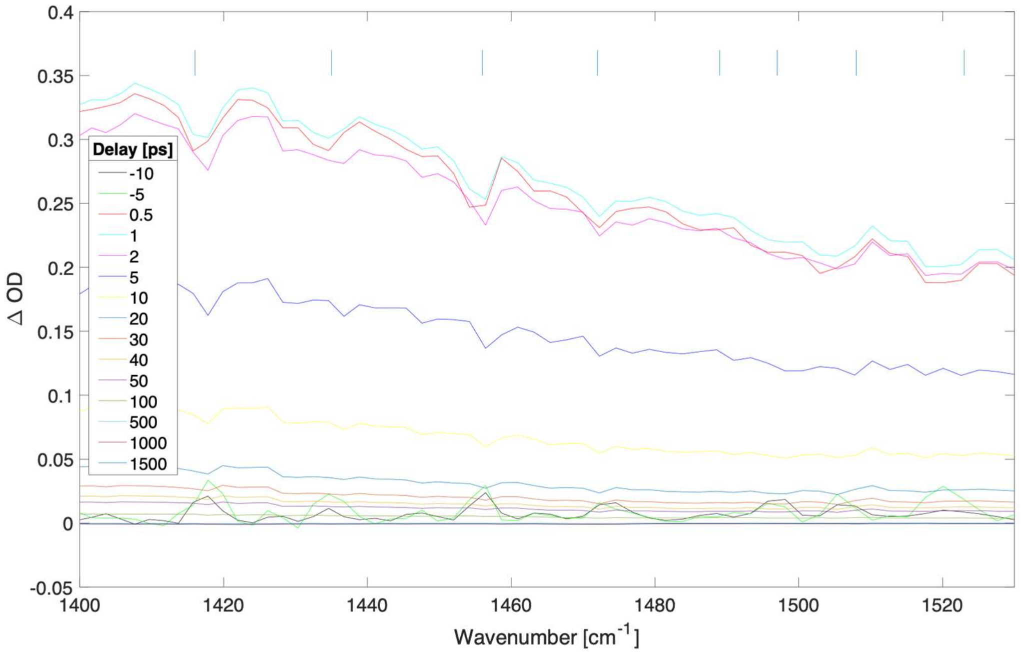

Figure S16.1. Difference transient absorption spectra at selected delays (ps) for a 300 nm thick film of $MAPbI_3$ with pump irradiance of 4.48e10 W/cm^2 (fluence of 11,204.5 uJ/cm^2) at 539 nm.

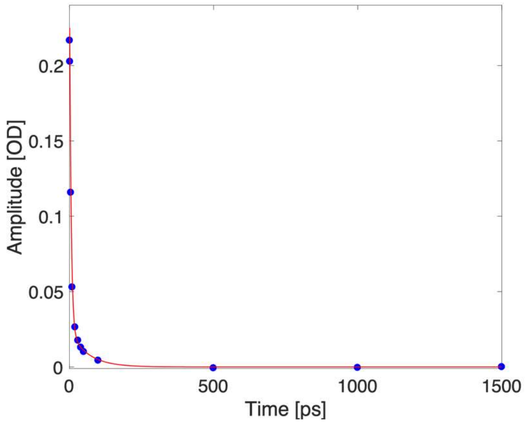

Figure S16.2. Kinetic trace taken at 1470 cm^{-1} (blue dots depict experimental data for selected delays, red curve represents a biexponential fit).

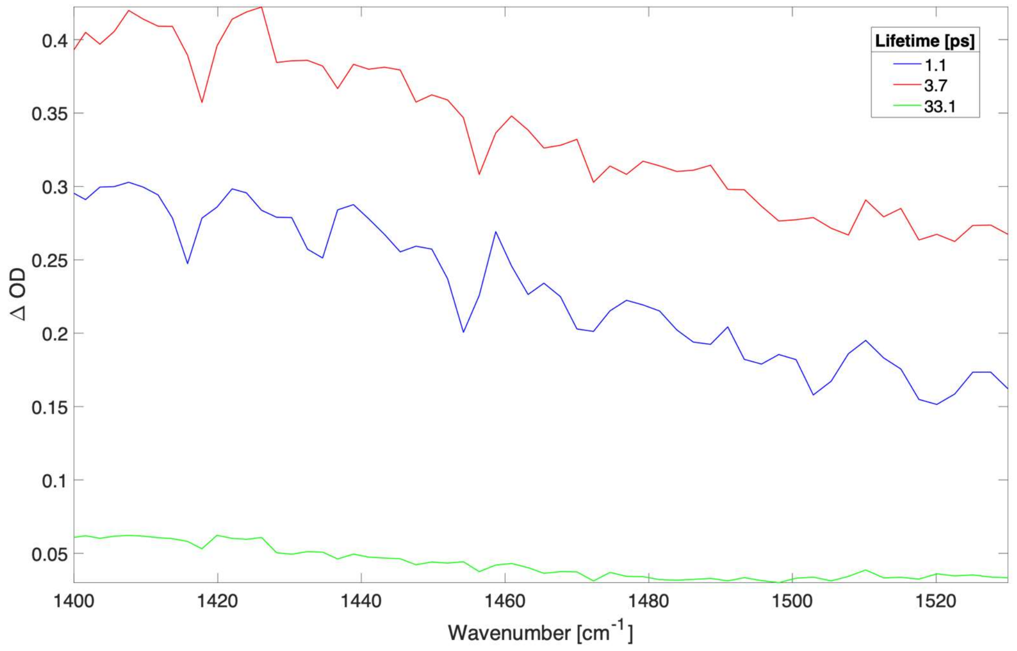

Figure S16.3. Time independent spectra of sequential three compartments global fit to spectra shown in Figure S16.1. The time constants (and contribution to data) were: 3.7 ps (54.6%), 1.1 ps (37.3%), and 33.1 ps (8%).

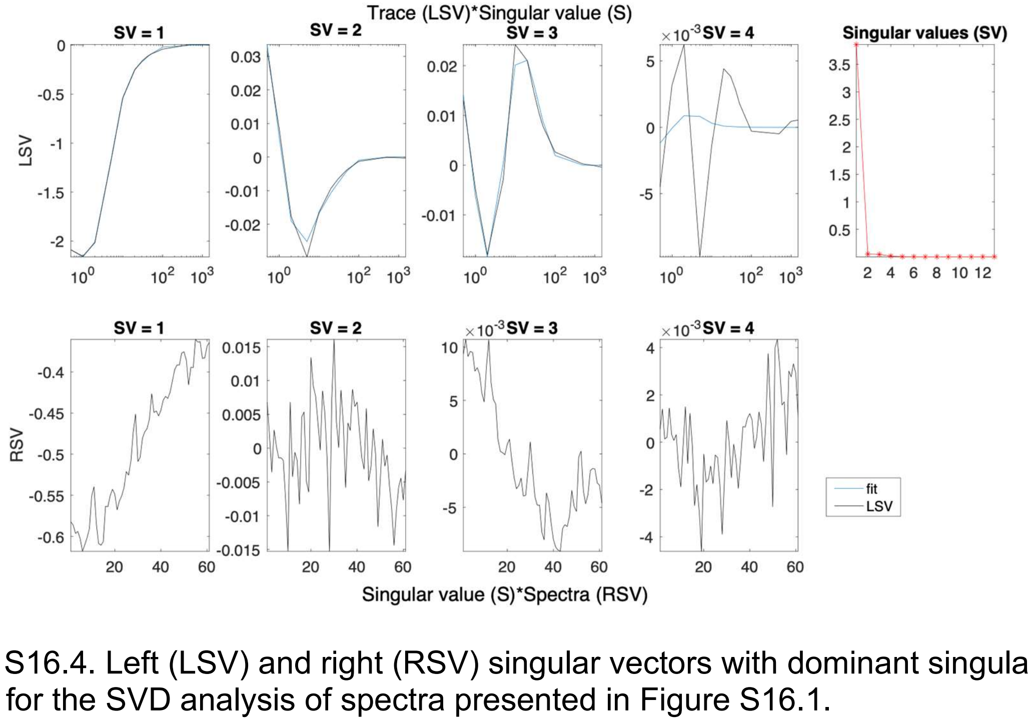

Figure S16.4. Left (LSV) and right (RSV) singular vectors with dominant singular values for the SVD analysis of spectra presented in Figure S16.1.

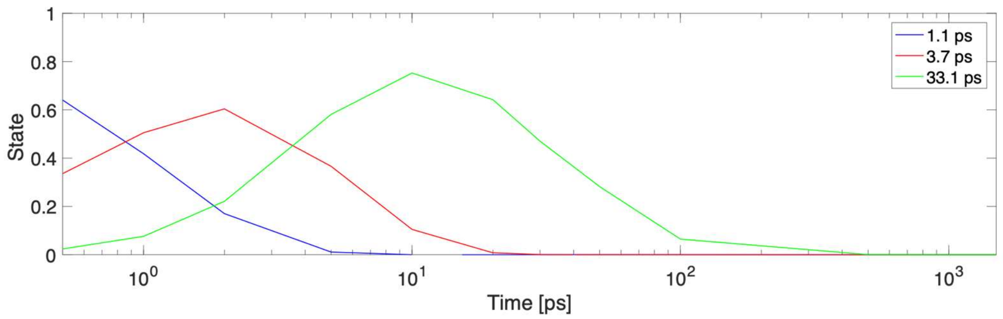

Figure S16.5. Concentration profiles for each time constant fitted to the data in Figure S16.3.

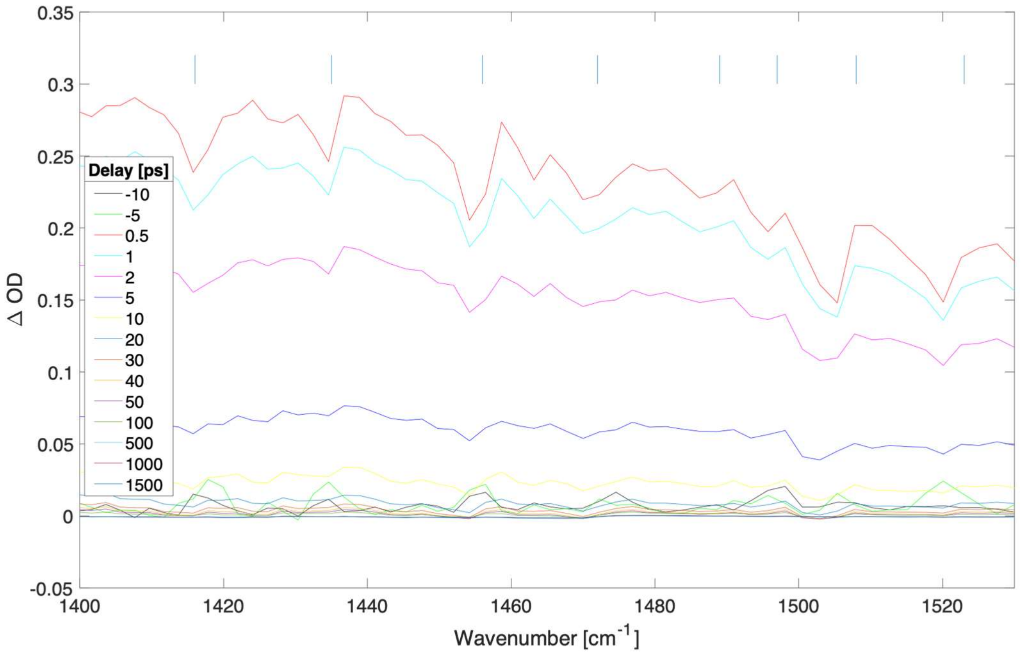

Figure S17.1. Difference transient absorption spectra at selected delays (ps) for a 300 nm thick film of $MAPbI_3$ with pump irradiance of 2.83e10 W/cm^2 (fluence of 7,073.6 uJ/cm^2) at 539 nm.

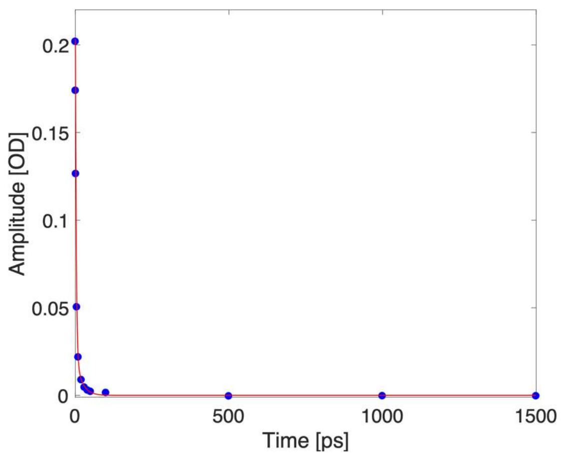

Figure S17.2. Kinetic trace taken at 1470 cm^{-1} (blue dots depict experimental data for selected delays, red curve represents a biexponential fit).

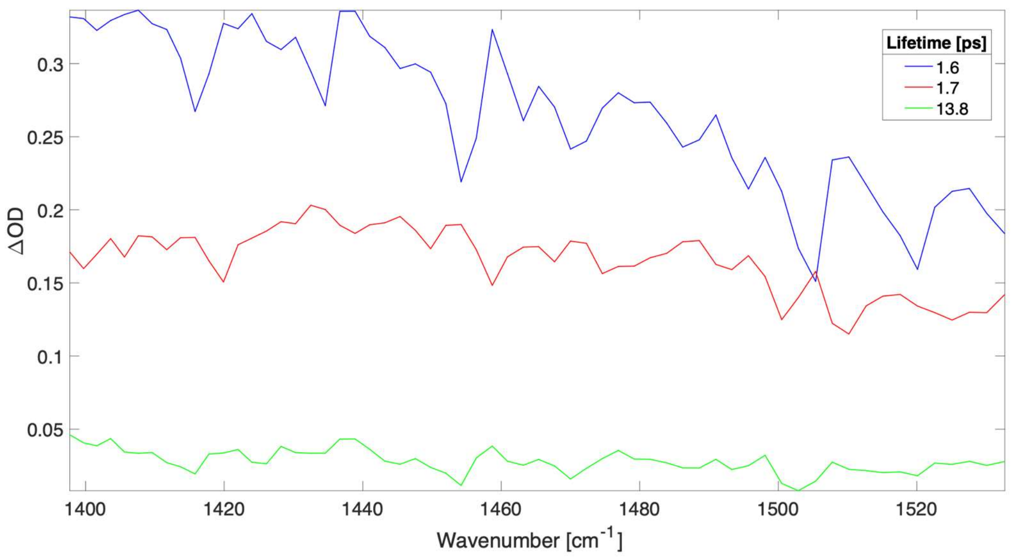

Figure S17.3. Time independent spectra of sequential three compartments global fit to spectra shown in Figure S17.1. The time constants (and contribution to data) were: 1.6 ps (54.3%), 1.7 ps (38.1%), and 13.8 ps (7.6%).

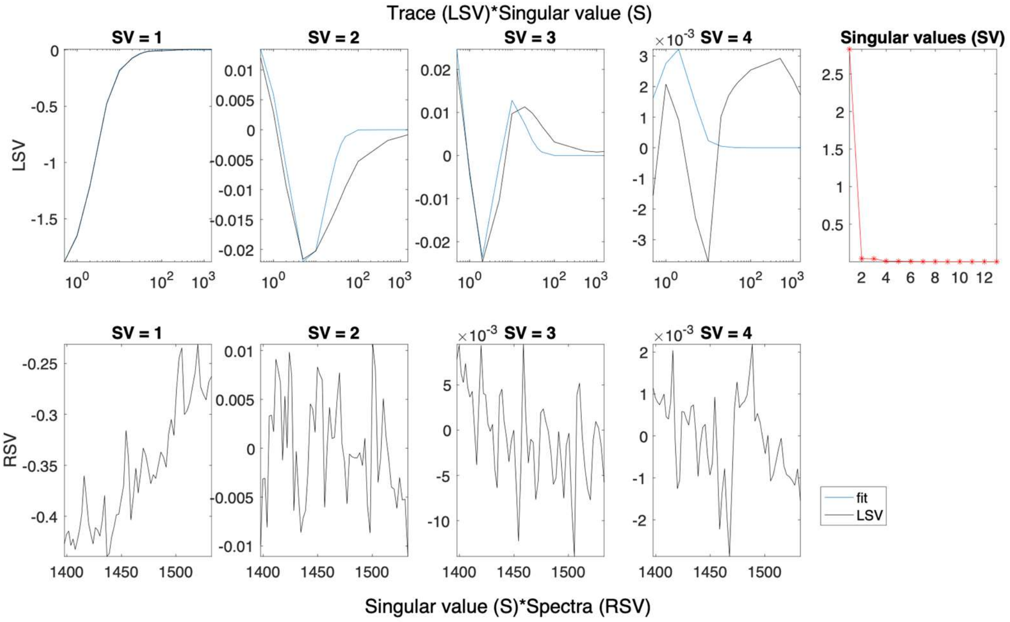

Figure S17.4. Left (LSV) and right (RSV) singular vectors with dominant singular values for the SVD analysis of spectra presented in Figure S17.1.

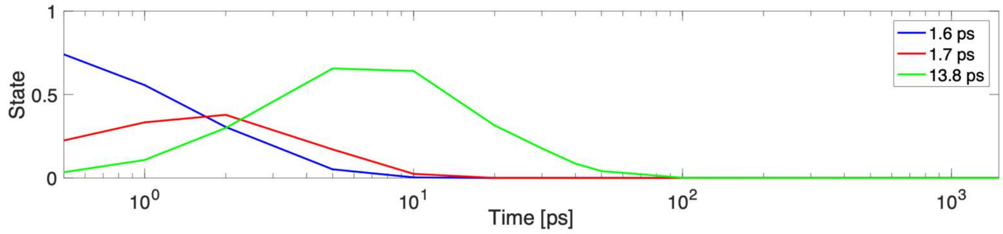

Figure S17.5. Concentration profiles for each time constant fitted to the data in Figure S17.3.

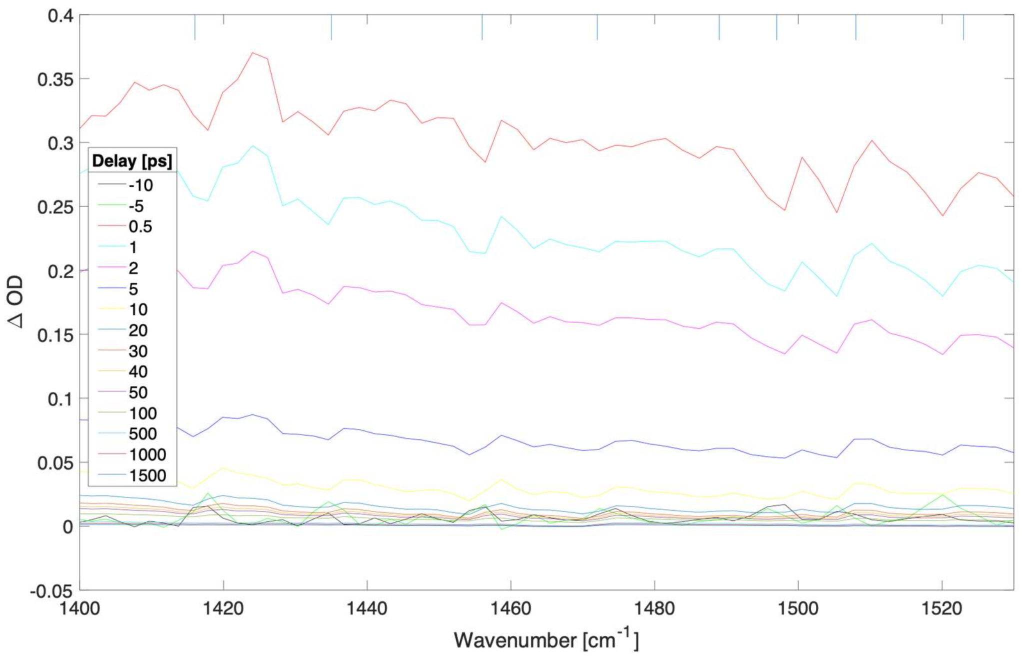

Figure S18.1. Difference transient absorption spectra at selected delays (ps) for a 300 nm thick film of $MAPbI_3$ with pump irradiance of 1.78.9e10 W/cm^2 (fluence of 4,470.5 uJ/cm^2) at 539 nm.

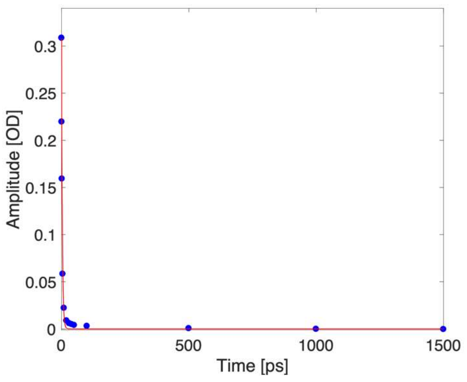

Figure S18.2. Kinetic trace taken at 1470 cm^{-1} (blue dots depict experimental data for selected delays, red curve represents a biexponential fit).

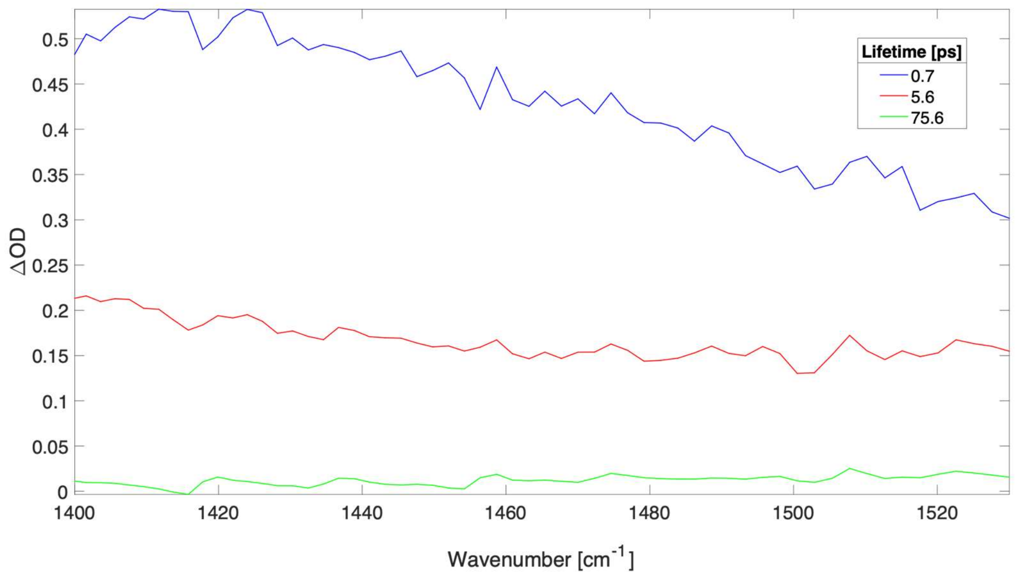

Figure S18.3. Time independent spectra of sequential three compartments global fit to spectra shown in Figure S18.1. The time constants were: 2.8 ps, 8.4 ps, and 321.3 ps. 16.3705 6.59951 77.03

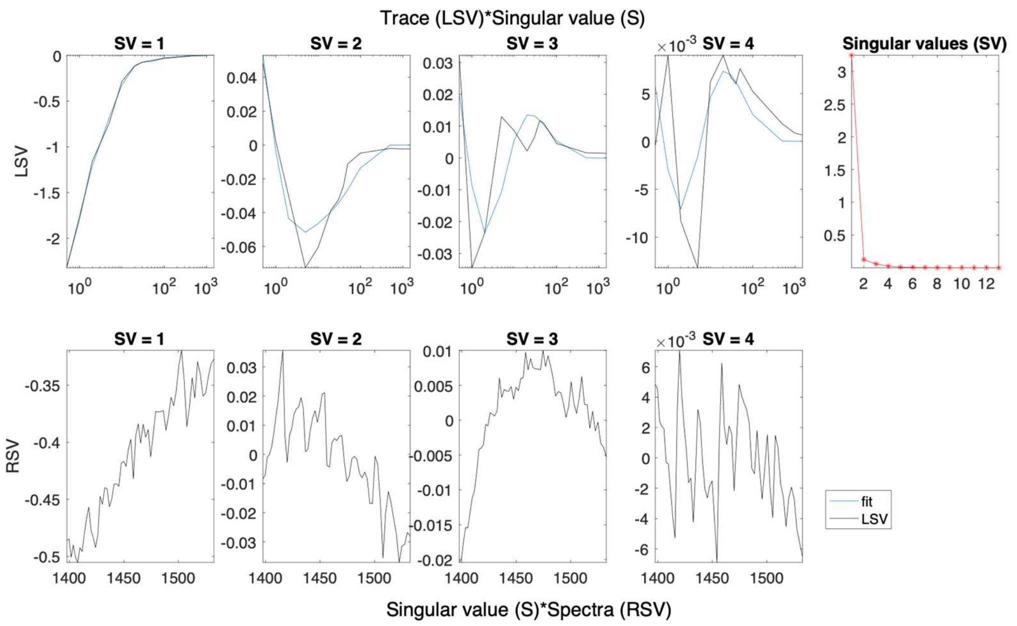

Figure S18.4. Left (LSV) and right (RSV) singular vectors with dominant singular values for the SVD analysis of spectra presented in Figure S18.1.

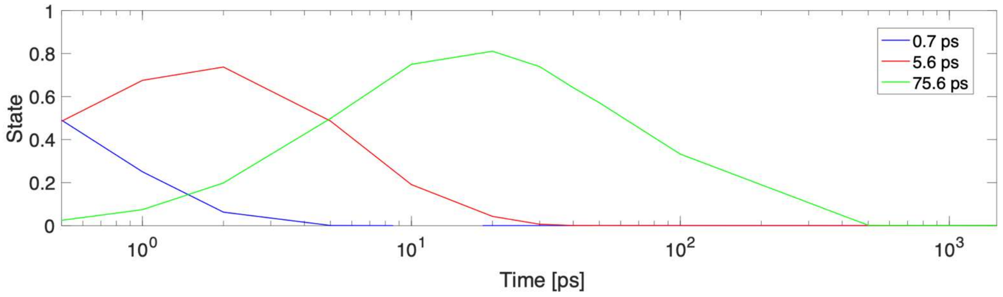

Figure S18.5. Concentration profiles for each time constant fitted to the data in Figure S18.3.

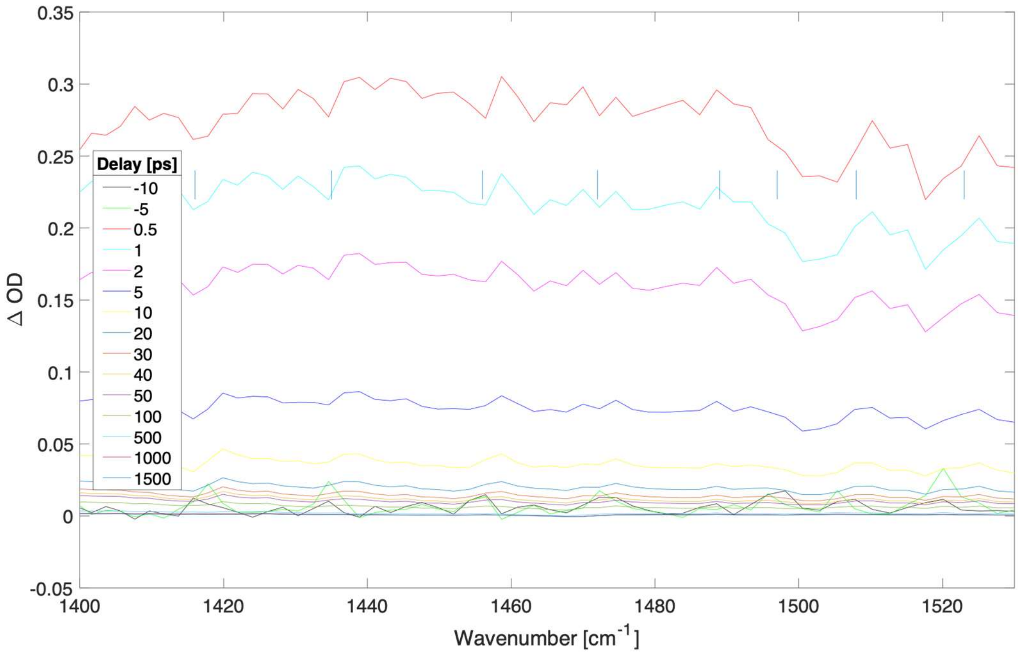

Figure S19.1. Difference transient absorption spectra at selected delays (ps) for a 300 nm thick film of $MAPbI_3$ with pump irradiance of 1.13e10 W/cm^2 (fluence of 2,829.4uJ/cm^2) at 539 nm.

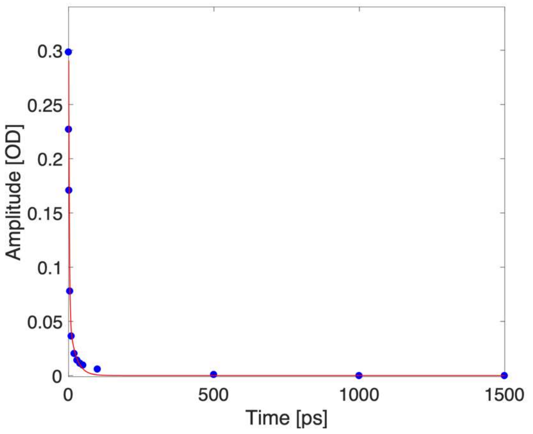

Figure S19.2. Kinetic trace taken at 1470 cm^{-1} (blue dots depict experimental data for selected delays, red curve represents a biexponential fit).

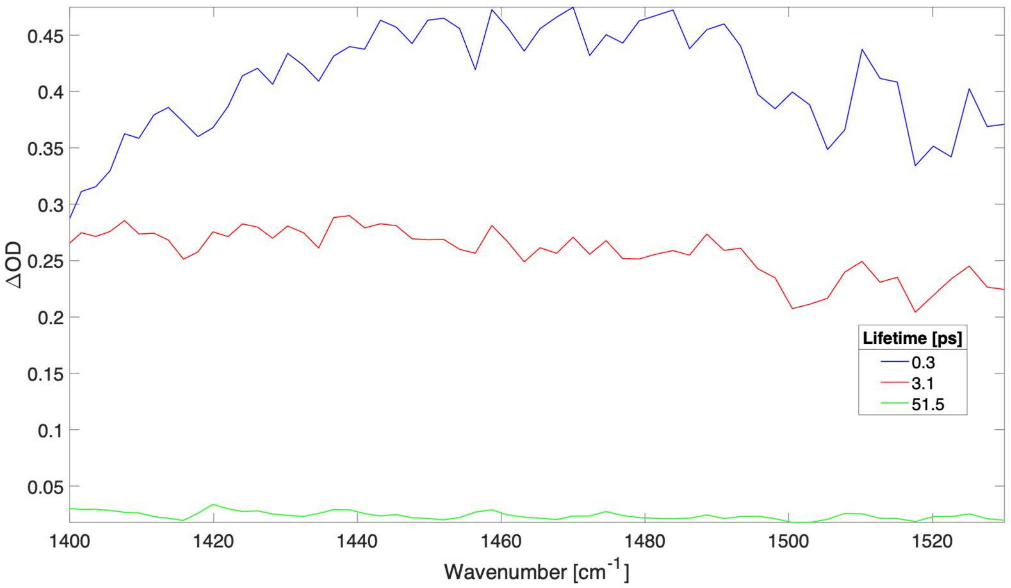

Figure S19.3. Time independent spectra of sequential three compartments global fit to spectra shown in Figure S19.1. The time constants (and contribution to data) were: 3.8 ps (64.3%), 1.2 ps (32.0%), and 31.5 ps (3.7%).

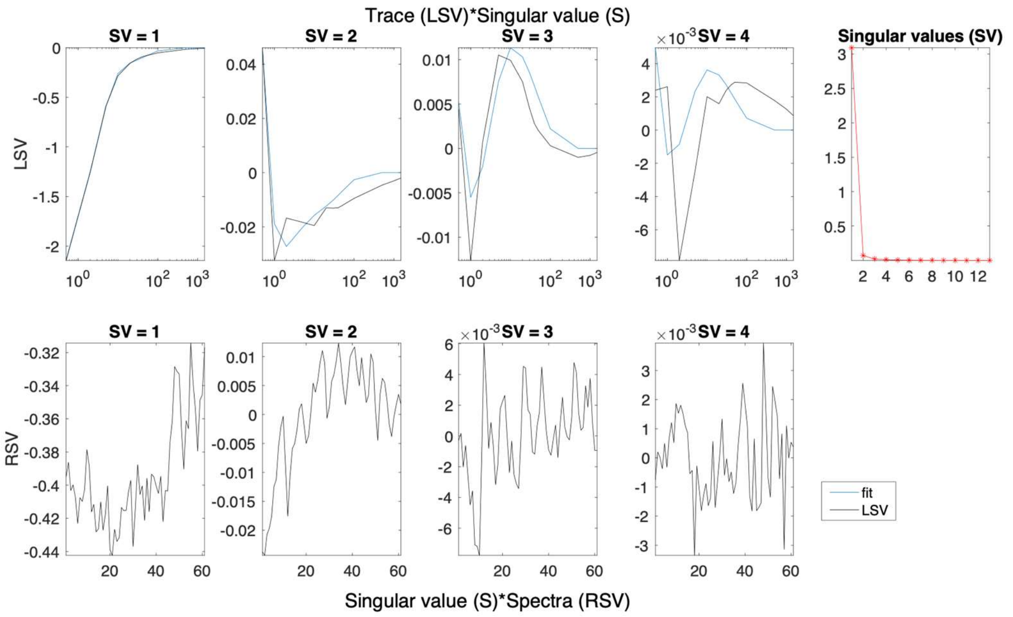

Figure S19.4. Left (LSV) and right (RSV) singular vectors with dominant singular values for the SVD analysis of spectra presented in Figure S19.1.

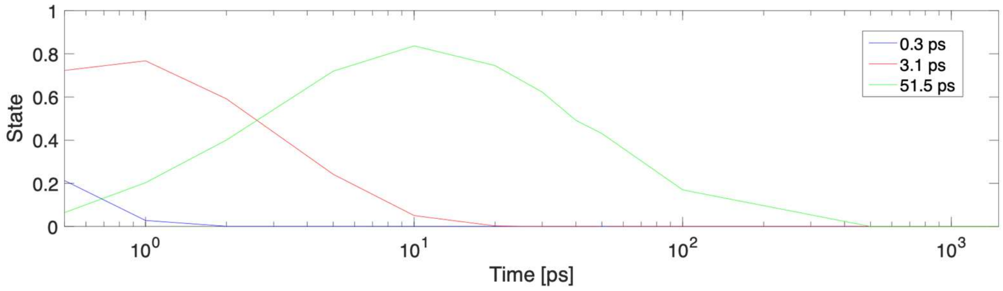

Figure S19.5. Concentration profiles for each time constant fitted to the data in Figure S19.3.

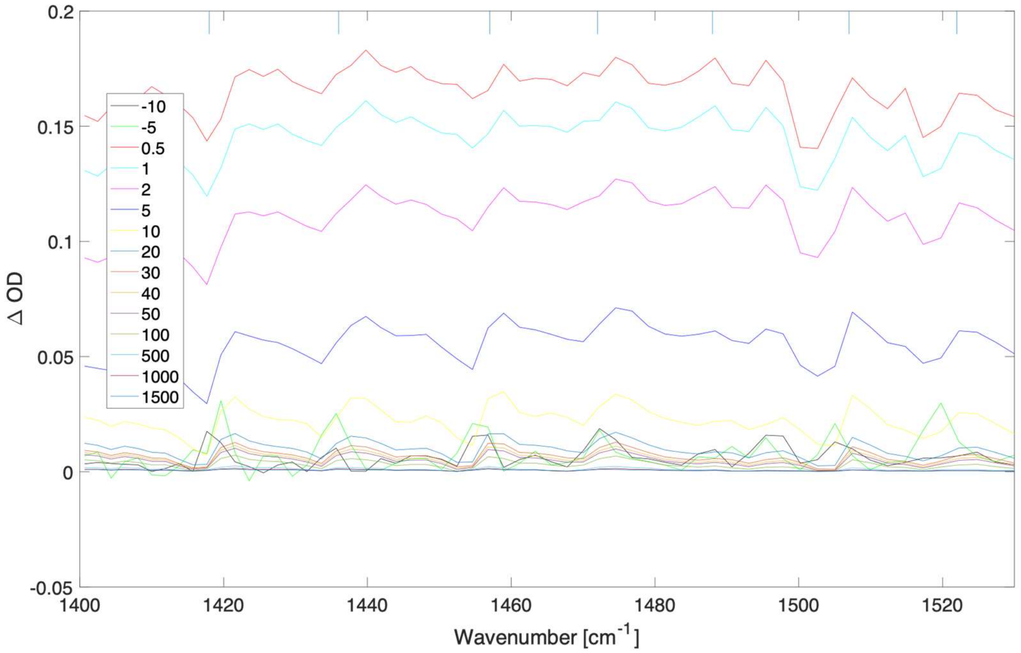

Figure S20.1. Difference transient absorption spectra at selected delays (ps) for a 300 nm thick film of $MAPbI_3$ with pump irradiance of 7.10e9 W/cm^2 (fluence of 1,774.0 uJ/cm^2) at 539 nm.

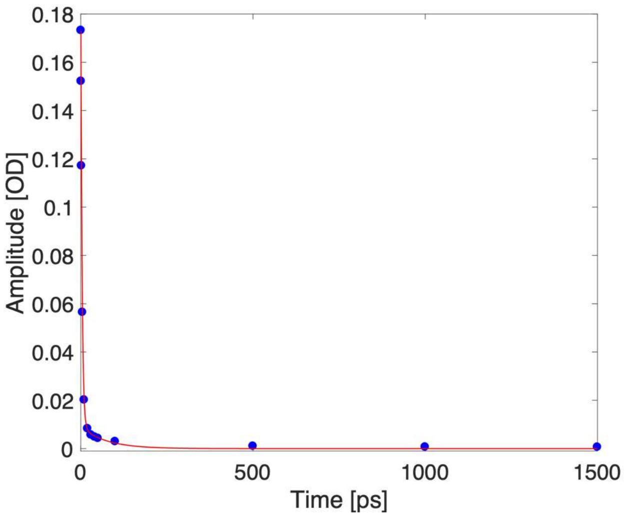

Figure S20.2. Kinetic trace taken at 1470 cm^{-1} (blue dots depict experimental data for selected delays, red curve represents a biexponential fit).

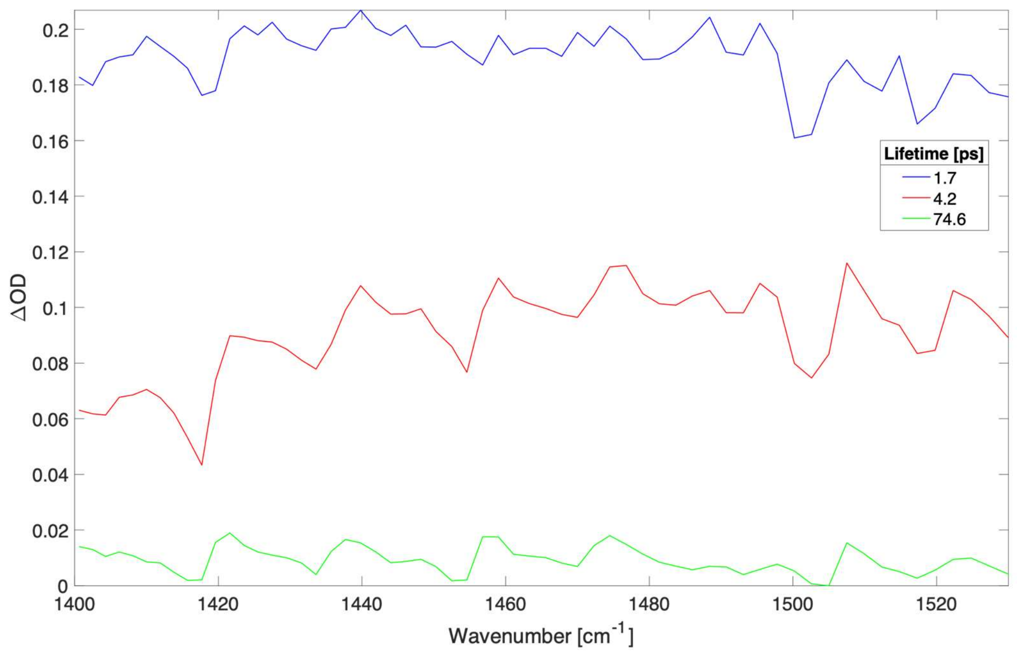

Figure S20.3. Time independent spectra of sequential three compartments global fit to spectra shown in Figure S20.1. The time constants (and contribution to data) were: 3.8 ps (61.4%), 1.2 ps (33.8%), and 31.5 ps (4.8%).

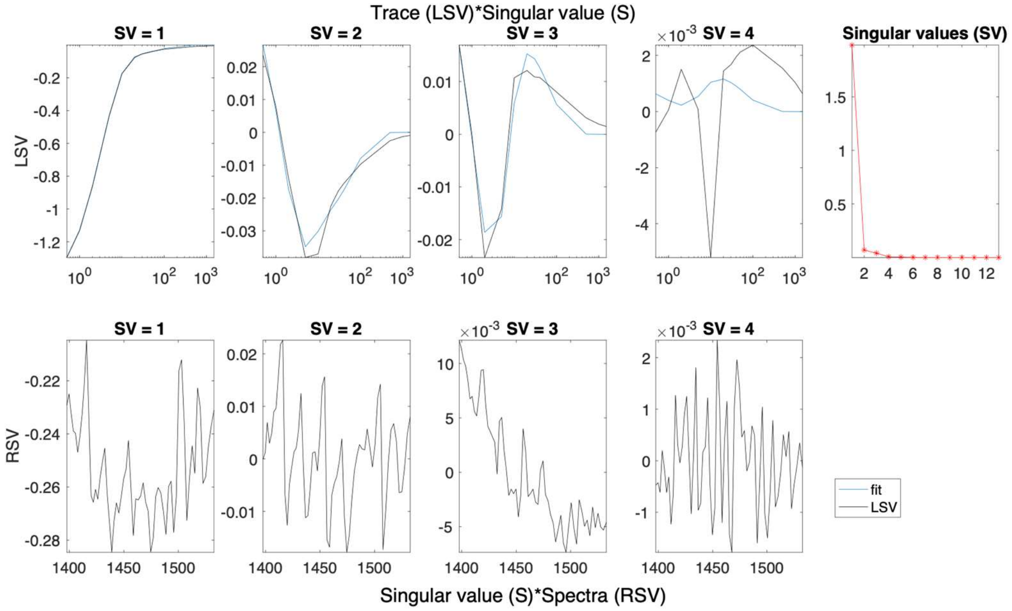

Figure S20.4. Left (LSV) and right (RSV) singular vectors with dominant singular values for the SVD analysis of spectra presented in Figure S20.1.

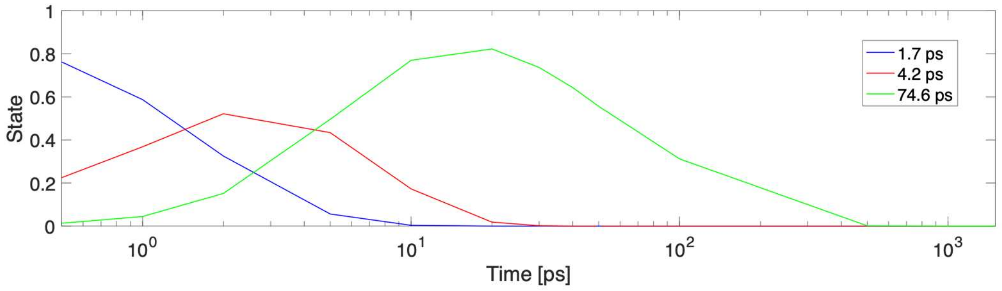

Figure S20.5. Concentration profiles for each time constant fitted to the data in Figure S20.2.

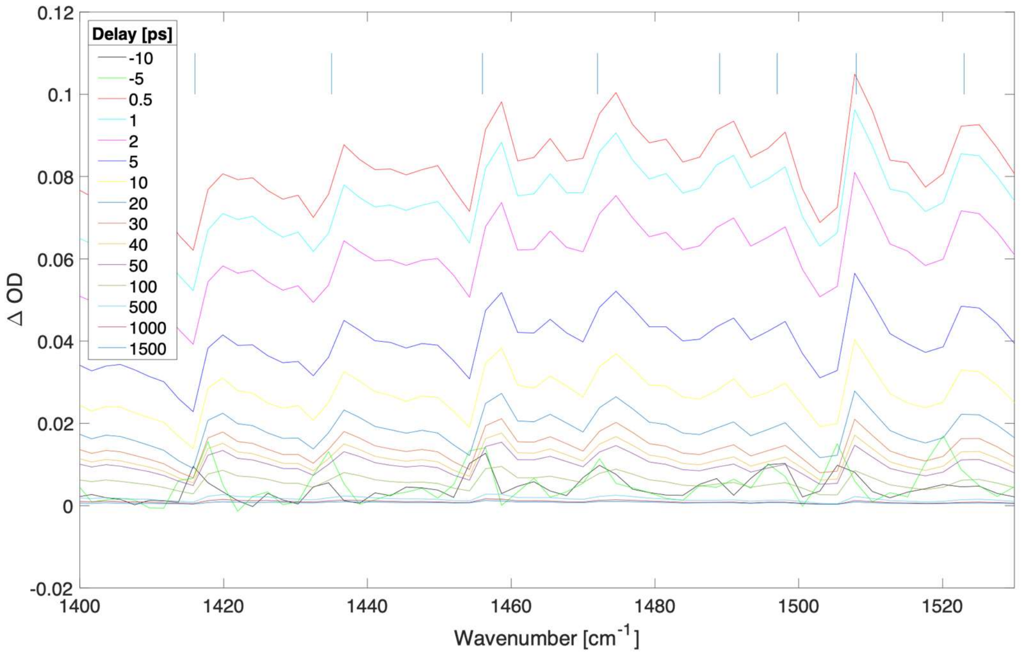

Figure S21.1. Difference transient absorption spectra at selected delays (ps) for a 300 nm thick film of $MAPbI_3$ with pump irradiance of 2.83e9 W/cm^2 (fluence of 707.4 uJ/cm^2) at 539 nm.

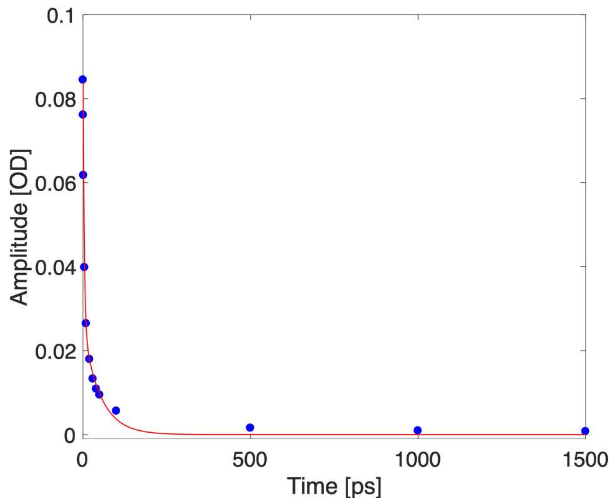

Figure S21.2. Kinetic trace taken at 1470 cm^{-1} (blue dots depict experimental data for selected delays, red curve represents a biexponential fit).

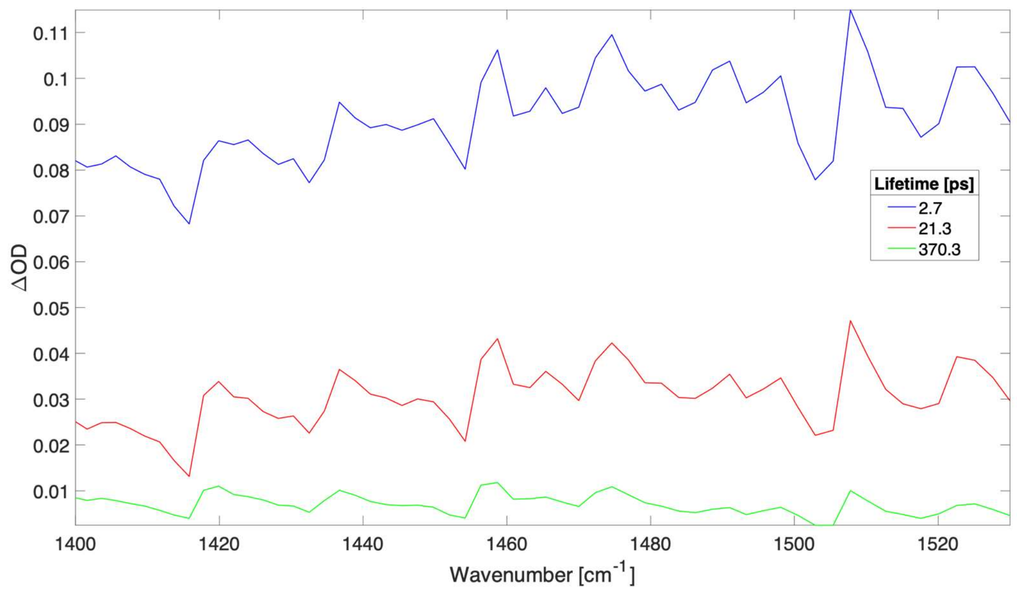

Figure S21.3. Time independent spectra of sequential three compartments global fit to spectra shown in Figure S21.1. The time constants (and contribution to data) were: 3.8 ps (67.0%), 1.2 ps (26.1%), and 31.5 ps (6.9%).

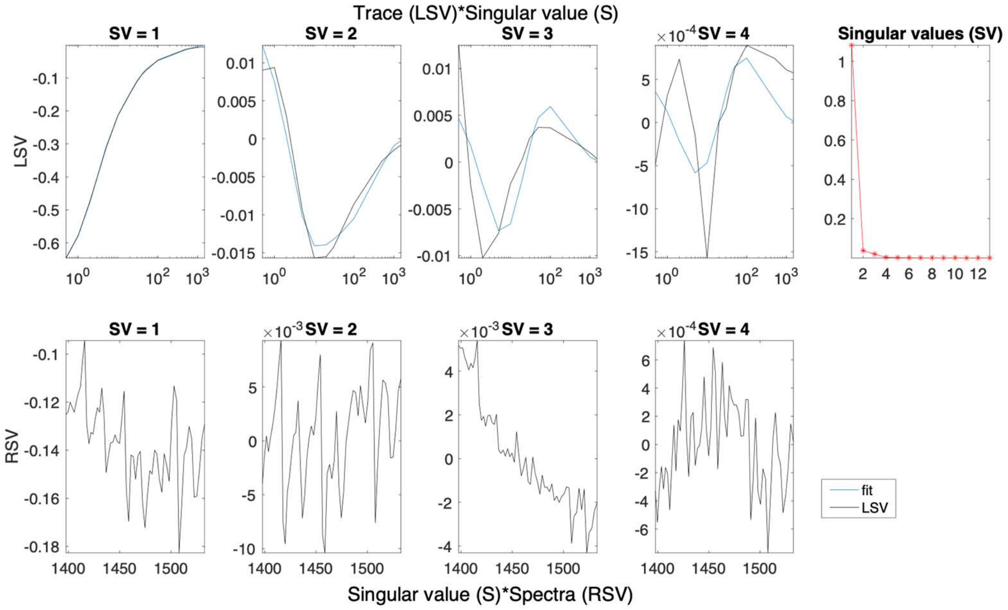

Figure S21.4. Left (LSV) and right (RSV) singular vectors with dominant singular values for the SVD analysis of spectra presented in Figure S21.1.

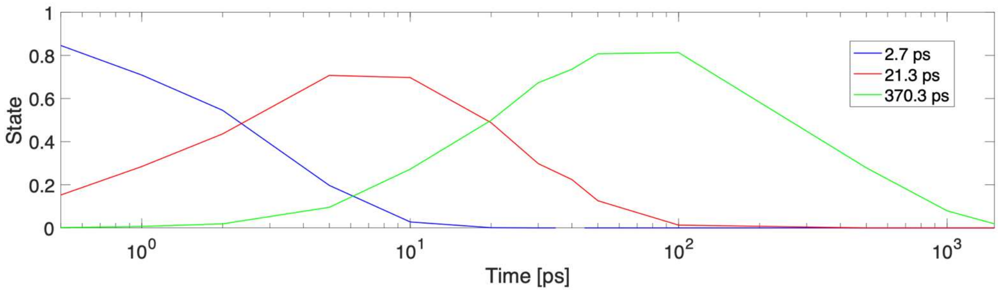

Figure S21.5. Concentration profiles for each time constant fitted to the data in Figure S21.2.

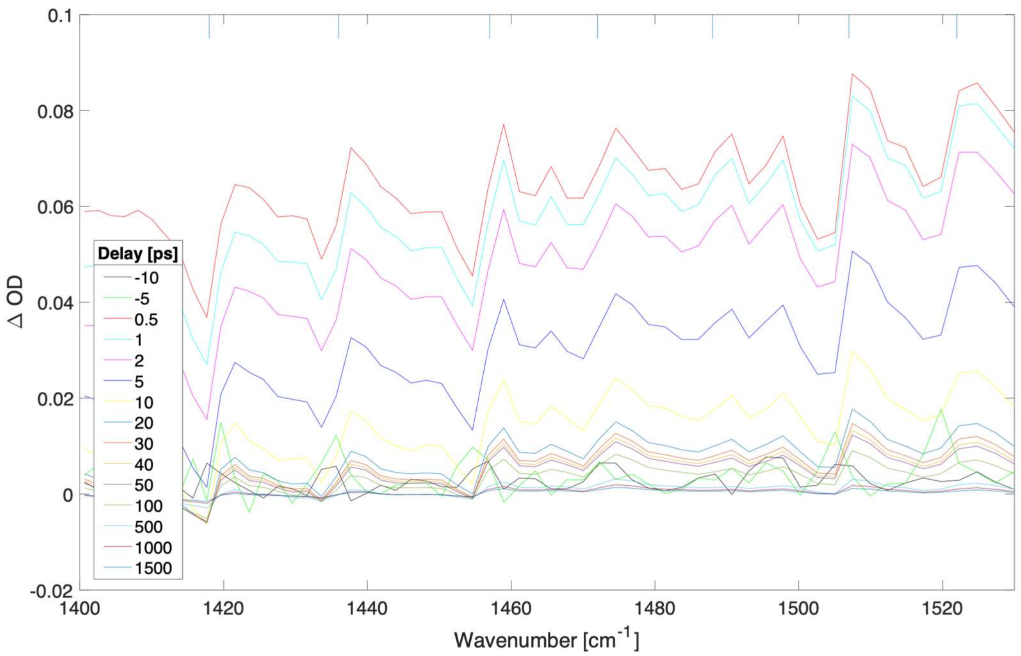

Figure S22.1. Difference transient absorption spectra at selected delays (ps) for a 300 nm thick film of $MAPbI_3$ with pump irradiance of 2.32 e9 W/cm^2 (fluence of 580.0 uJ/cm^2) at 539 nm.

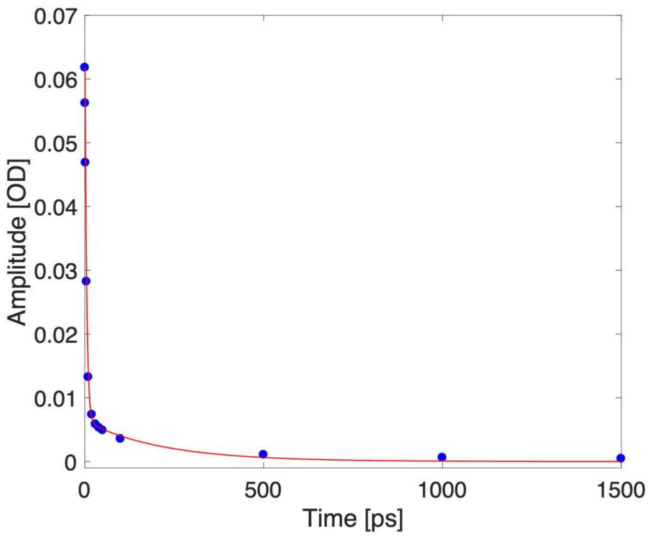

Figure S22.2. Kinetic trace taken at 1470 cm^{-1} (blue dots depict experimental data for selected delays, red curve represents a biexponential fit).

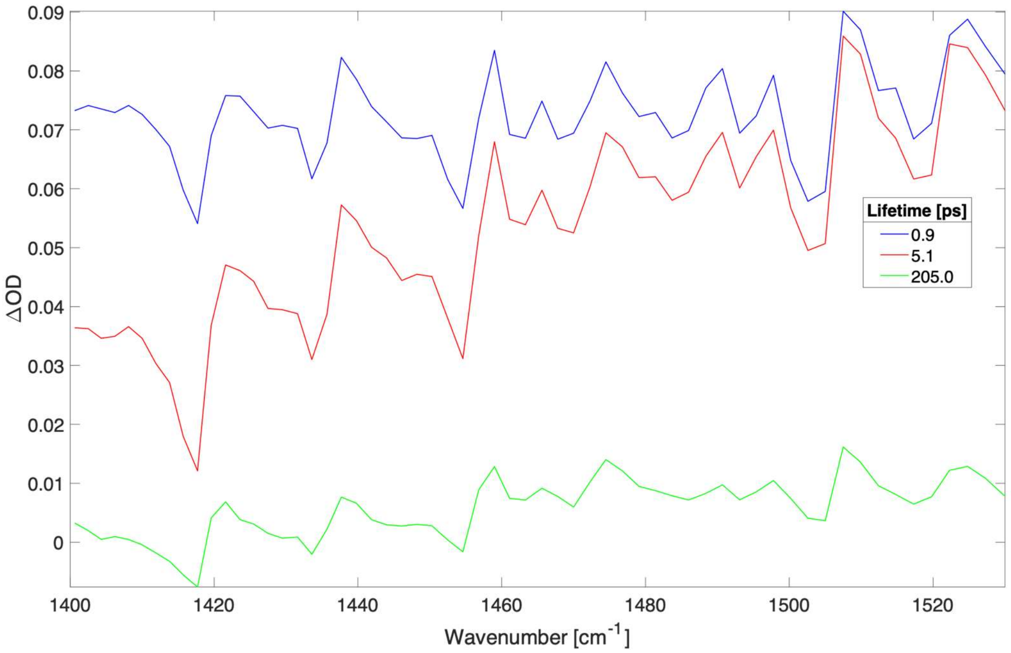

Figure S22.3. Time independent spectra of sequential three compartments global fit to spectra shown in Figure S22.1. The time constants (and contribution to data) were: 3.8 ps (53.6%), 1.2 ps (39.6%), and 31.5 ps (6.8%).

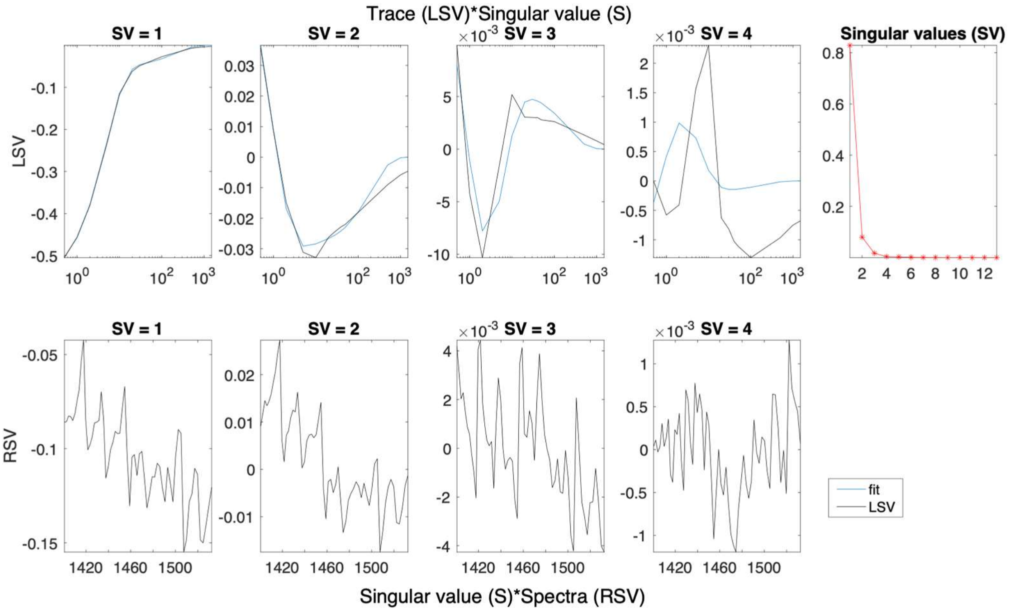

Figure S22.4. Left (LSV) and right (RSV) singular vectors with dominant singular values for the SVD analysis of spectra presented in Figure S22.1.

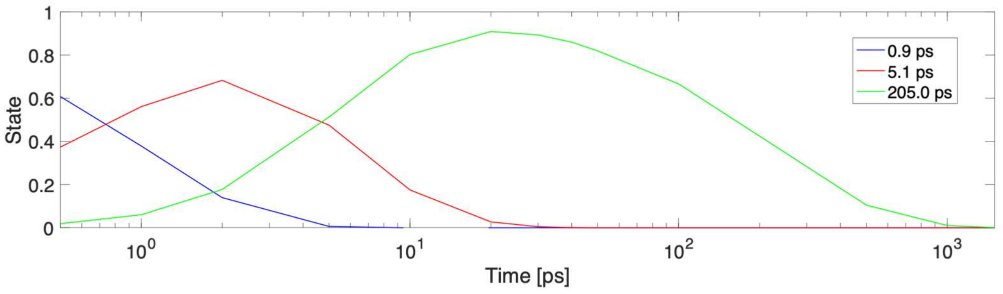

Figure S22.5. Concentration profiles for each time constant fitted to the data in Figure S22.3.

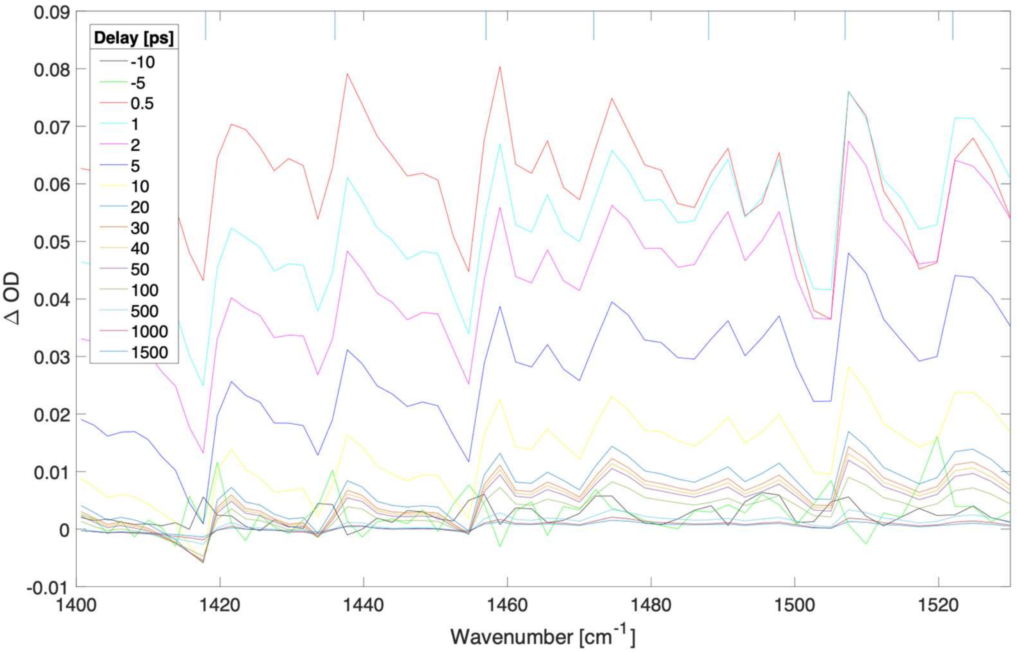

Figure S23.1. Difference transient absorption spectra at selected delays (ps) for a 300 nm thick film of $MAPbI_3$ with pump irradiance of 1.86e9 W/cm^2 (fluence of 464.0 uJ/cm^2) at 539 nm.

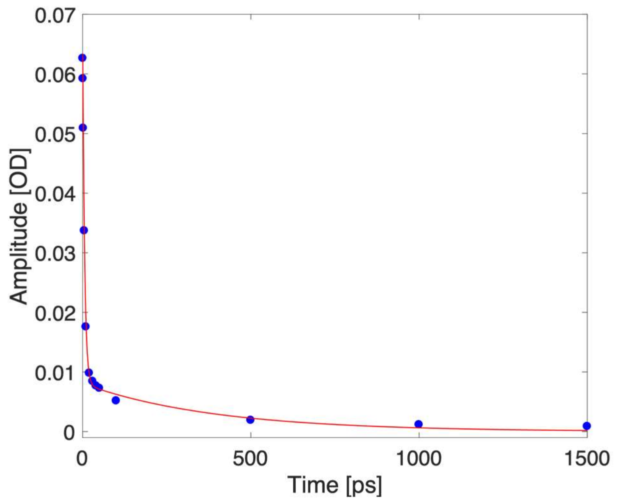

Figure S23.2. Kinetic trace taken at 1470 cm^{-1} (blue dots depict experimental data for selected delays, red curve represents a biexponential fit).

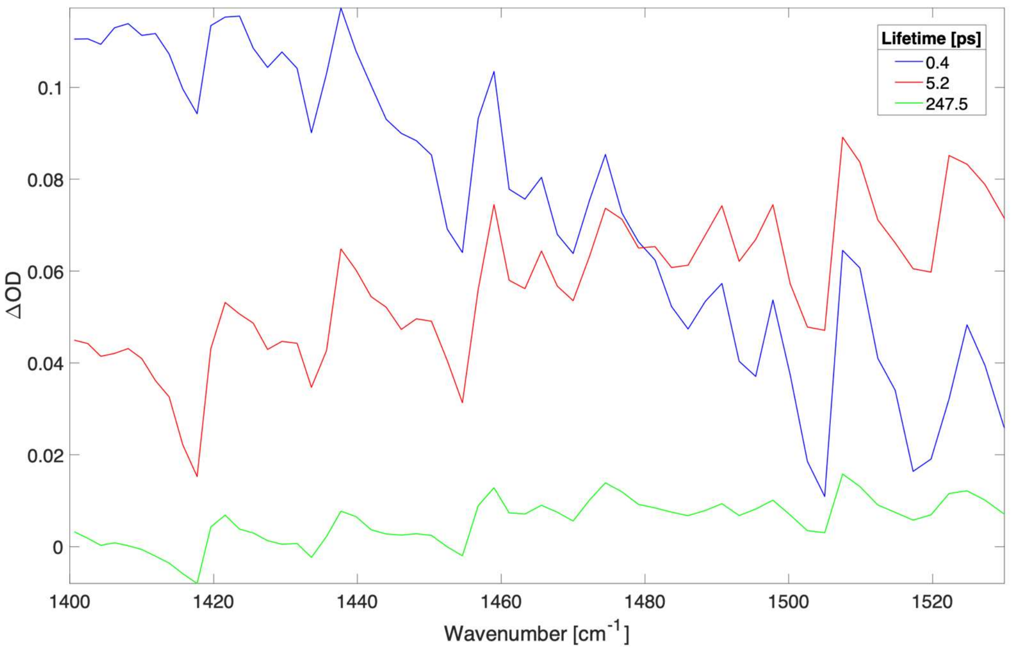

Figure S23.3. Time independent spectra of sequential three compartments global fit to spectra shown in Figure S23.1. . The time constants (and contribution to data) were: 0.4 ps (61.2%), 5.2 ps (33.1%), and 247.5 ps (5.7%).

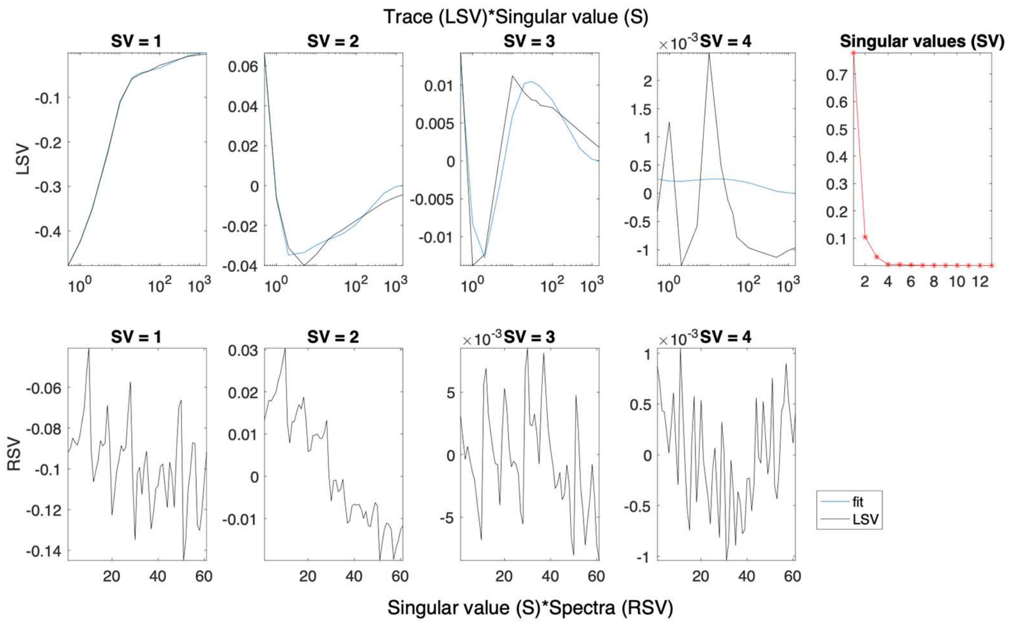

Figure S23.4. Left (LSV) and right (RSV) singular vectors with dominant singular values for the SVD analysis of spectra presented in Figure S23.1.

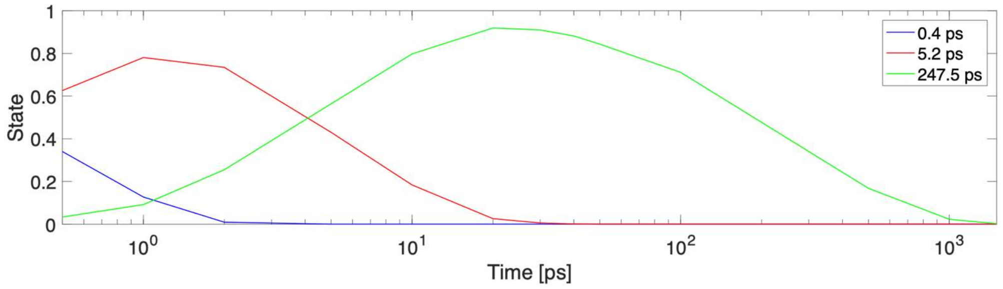

Figure S23.5. Concentration profiles for each time constant fitted to the data in Figure S23.3.

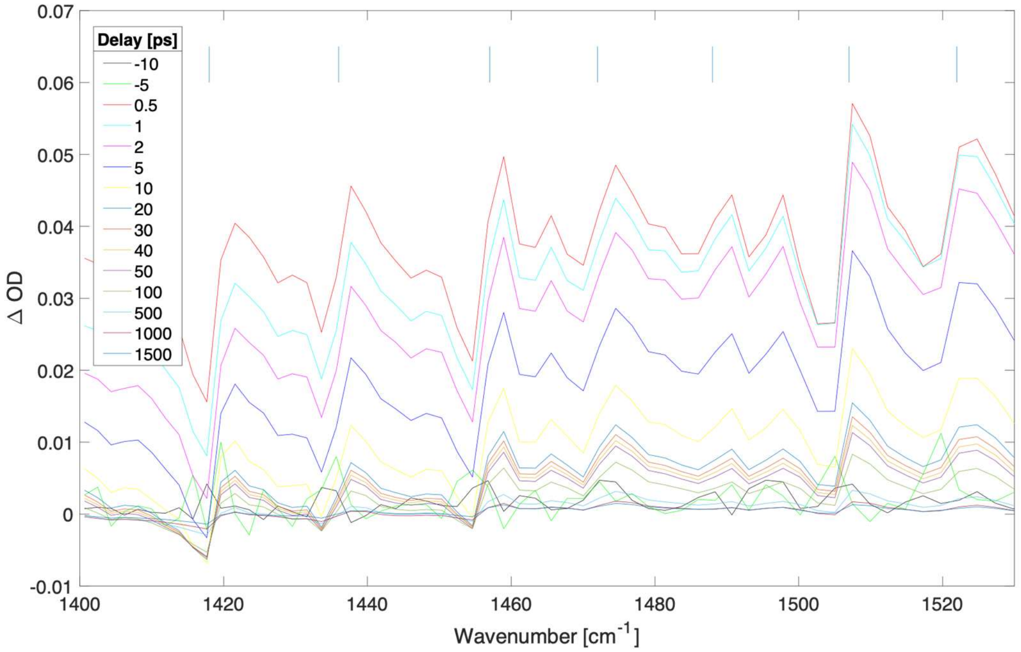

Figure S24.1. Difference transient absorption spectra at selected delays (ps) for a 300 nm thick film of $MAPbI_3$ with pump irradiance of 1.47e9 W/cm^2 (fluence of 367.8 uJ/cm^2) at 539 nm.

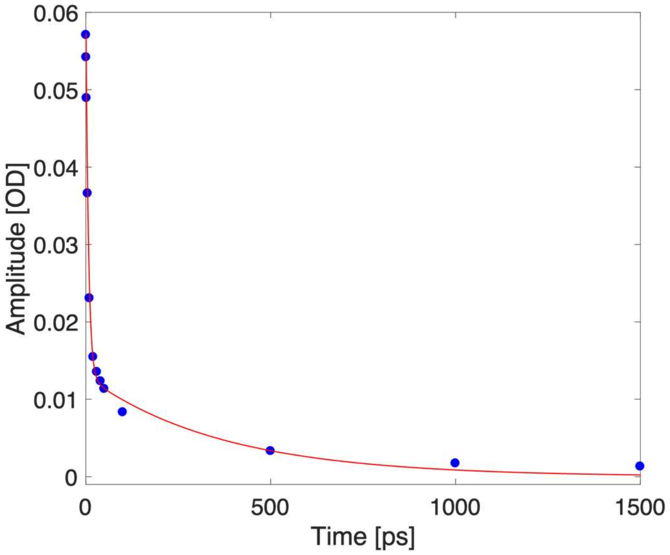

Figure S24.2. Kinetic trace taken at 1470 cm^{-1} (blue dots depict experimental data for selected delays, red curve represents a biexponential fit).

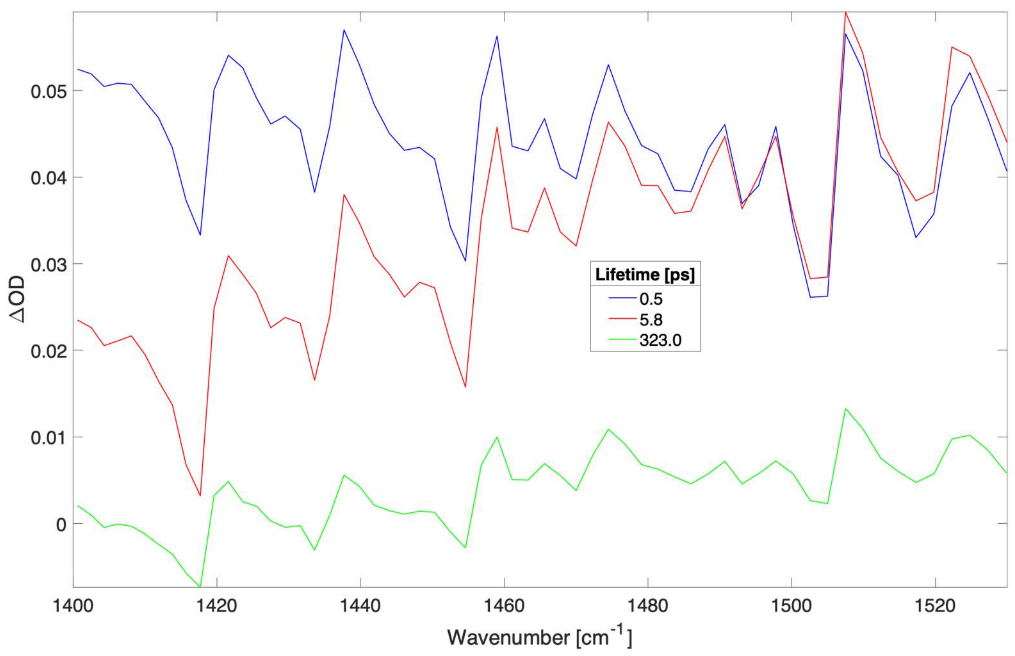

Figure S24.3. Time independent spectra of sequential three compartments global fit to spectra shown in Figure S24.1. The time constants (and contribution to data) were: 0.5 ps (56.9%), 5.8 ps (35.3%), and 323.0 ps (7.8%).

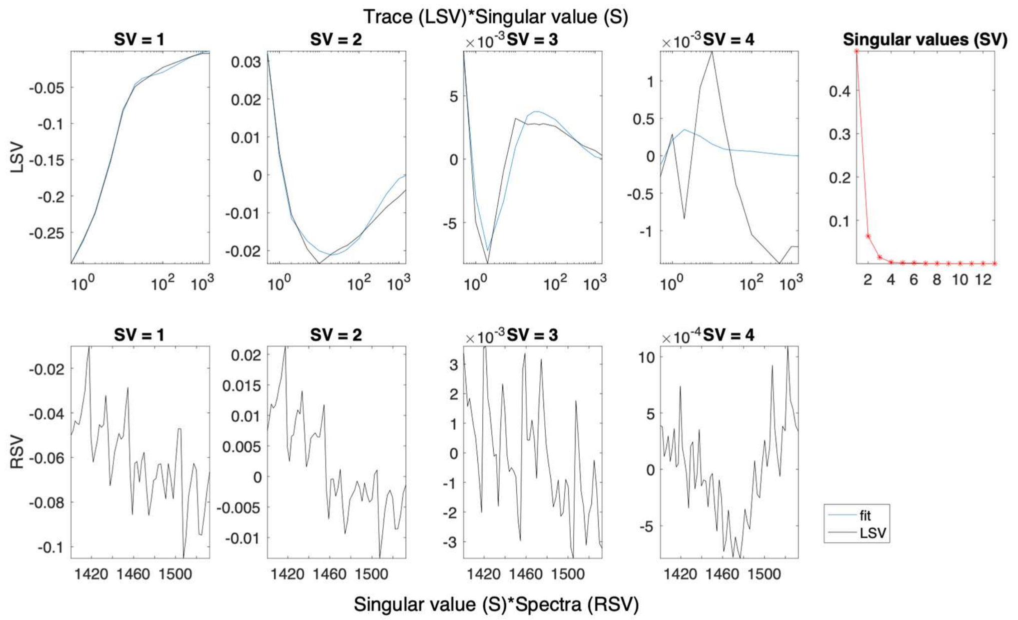

Figure S24.4. Left (LSV) and right (RSV) singular vectors with dominant singular values for the SVD analysis of spectra presented in Figure S24.1.

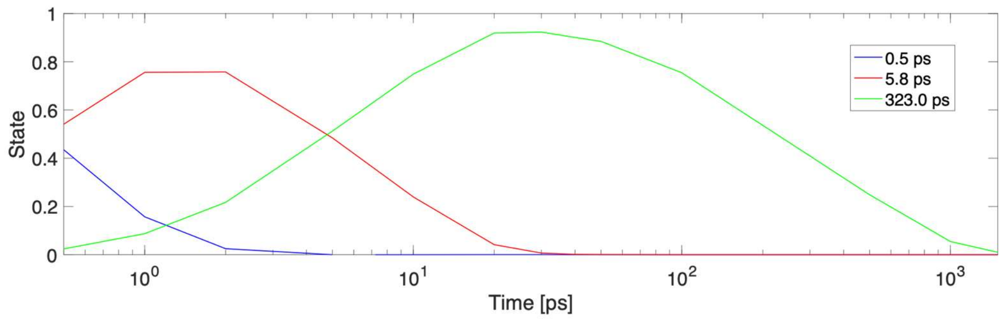

Figure S24.5. Concentration profiles for each time constant fitted to the data in Figure S24.3.

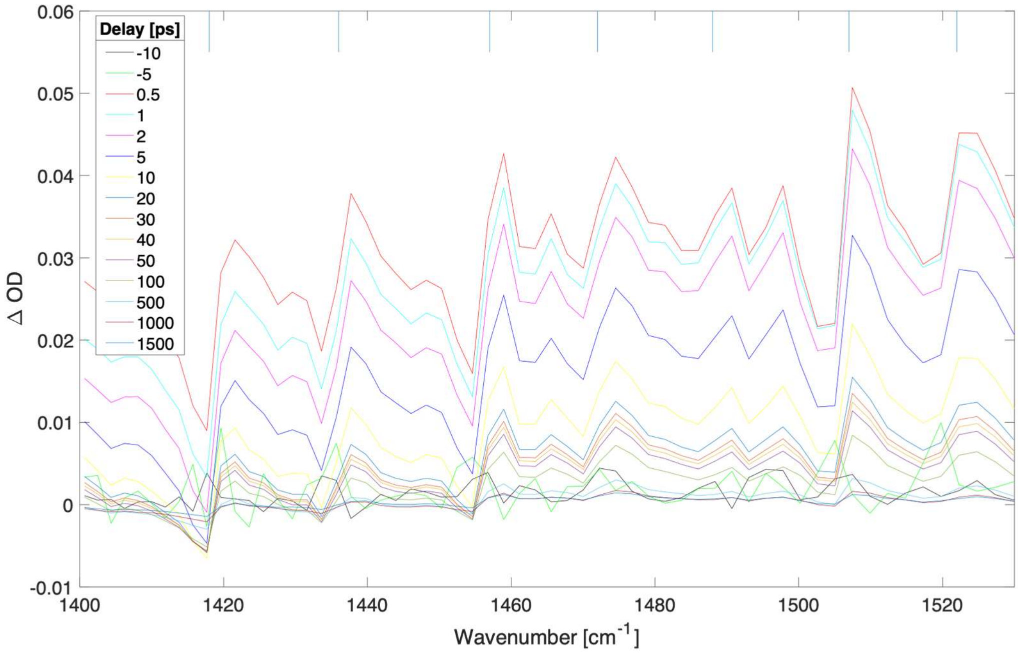

Figure S25.1. Difference transient absorption spectra at selected delays (ps) for a 300 nm thick film of $MAPbI_3$ with pump irradiance of 1.18e9 W/cm^2 (fluence of 294.2 uJ/cm^2) at 539 nm.

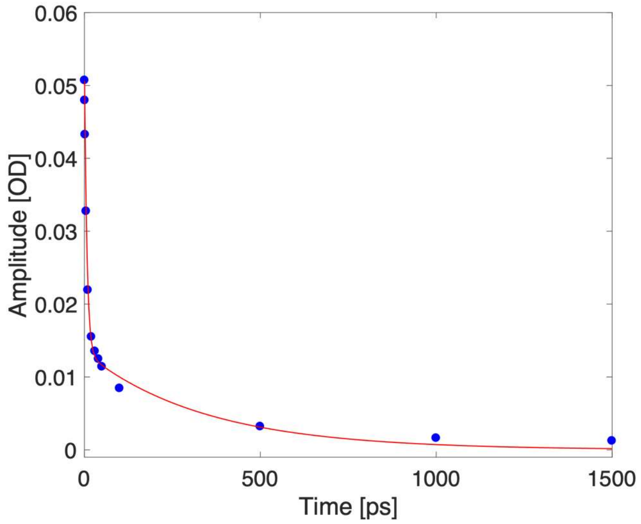

Figure S25.2. Kinetic trace taken at 1470 cm^{-1} (blue dots depict experimental data for selected delays, red curve represents a biexponential fit).

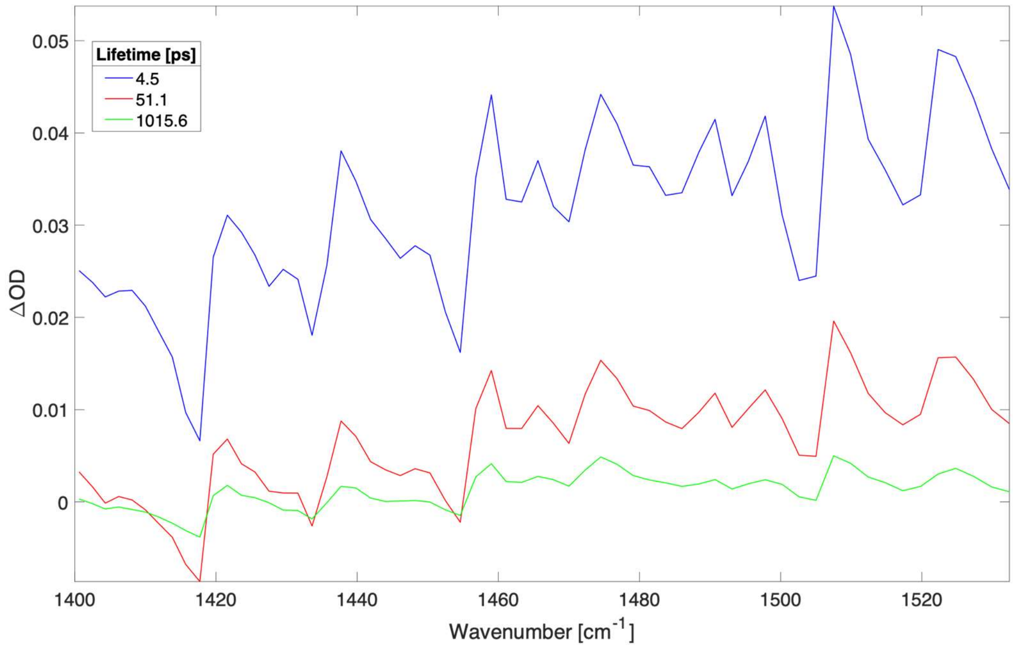

Figure S25.3. Time independent spectra of sequential three compartments global fit to spectra shown in Figure S25.1. . The time constants (and contribution to data) were: 4.5 ps (70.1%), 51.1 ps (22.7%), and 1015.6 ps (7.2%).

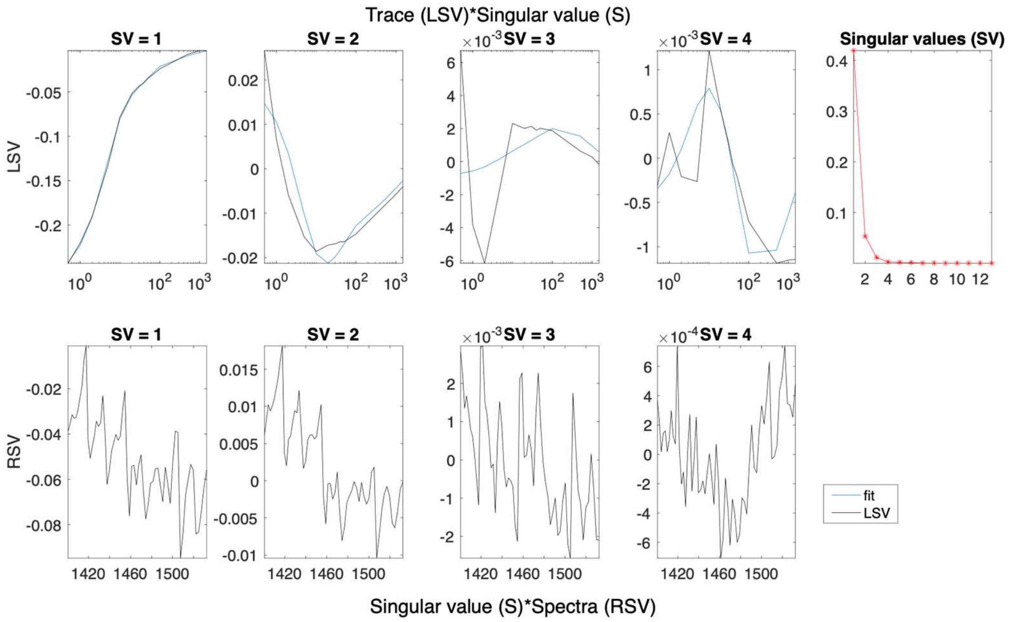

Figure S25.4. Left (LSV) and right (RSV) singular vectors with dominant singular values for the SVD analysis of spectra presented in Figure S25.1.

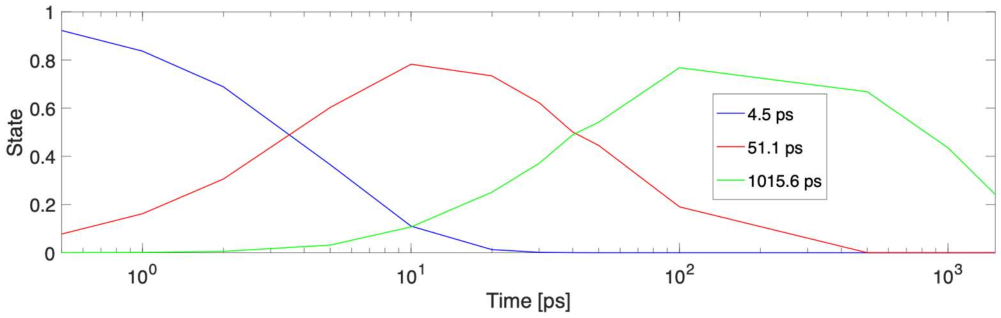

Figure S25.5. Concentration profiles for each time constant fitted to the data in Figure S25.3.

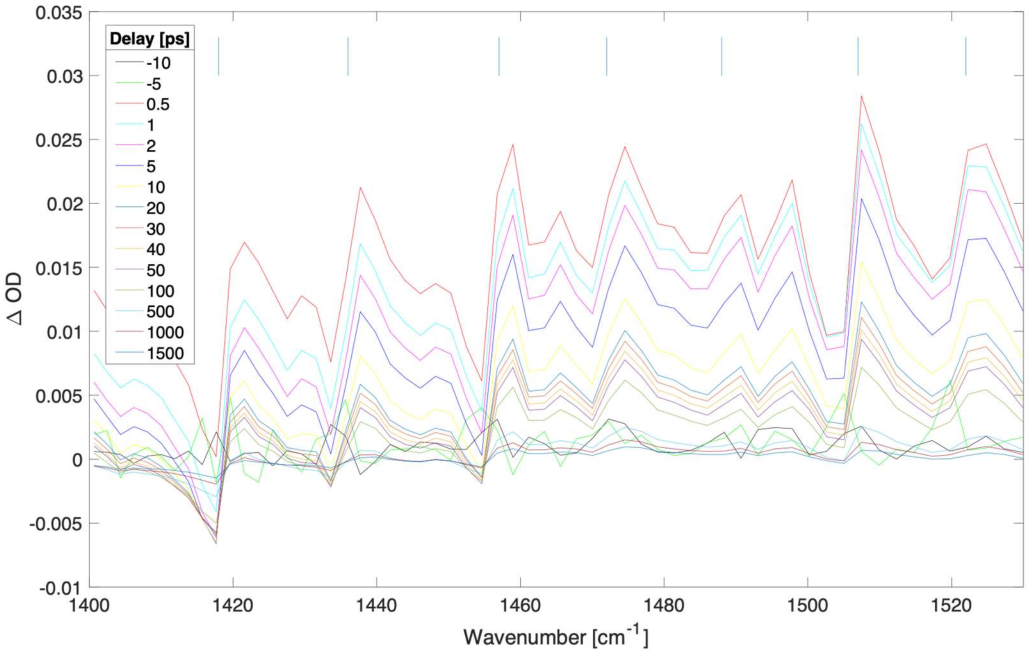

Figure S26.1. Difference transient absorption spectra at selected delays (ps) for a 300 nm thick film of $MAPbI_3$ with pump irradiance of 9.05e8 W/cm^2 (fluence of 226.4 uJ/cm^2) at 539 nm.

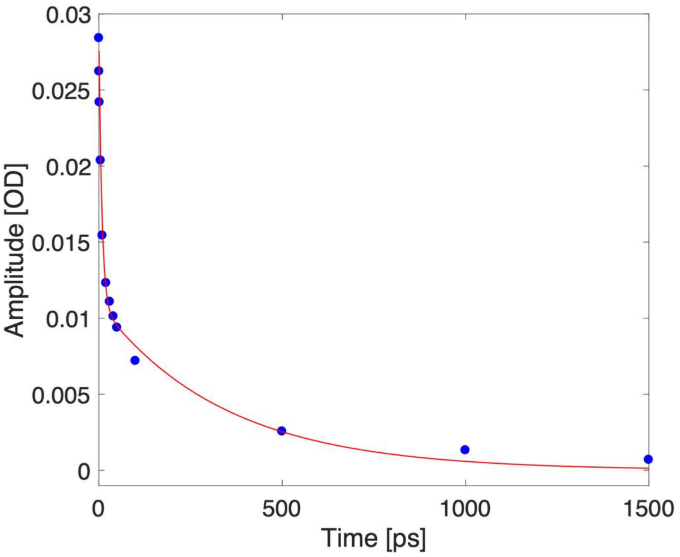

Figure S26.2. Kinetic trace taken at 1470 cm^{-1} (blue dots depict experimental data for selected delays, red curve represents a biexponential fit).

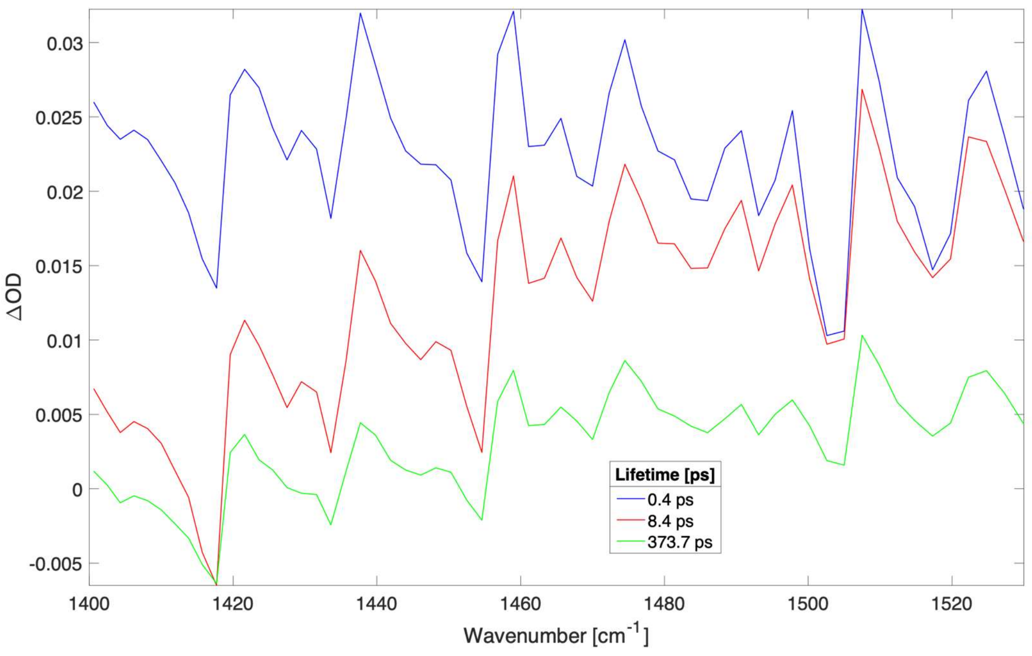

Figure S26.3. Time independent spectra of sequential three compartments global fit to spectra shown in Figure S26.1. The time constants (and contribution to data) were: 0.4 ps (61.9%), 8.4 ps (27.0%), and 373.7 ps (11.1%).

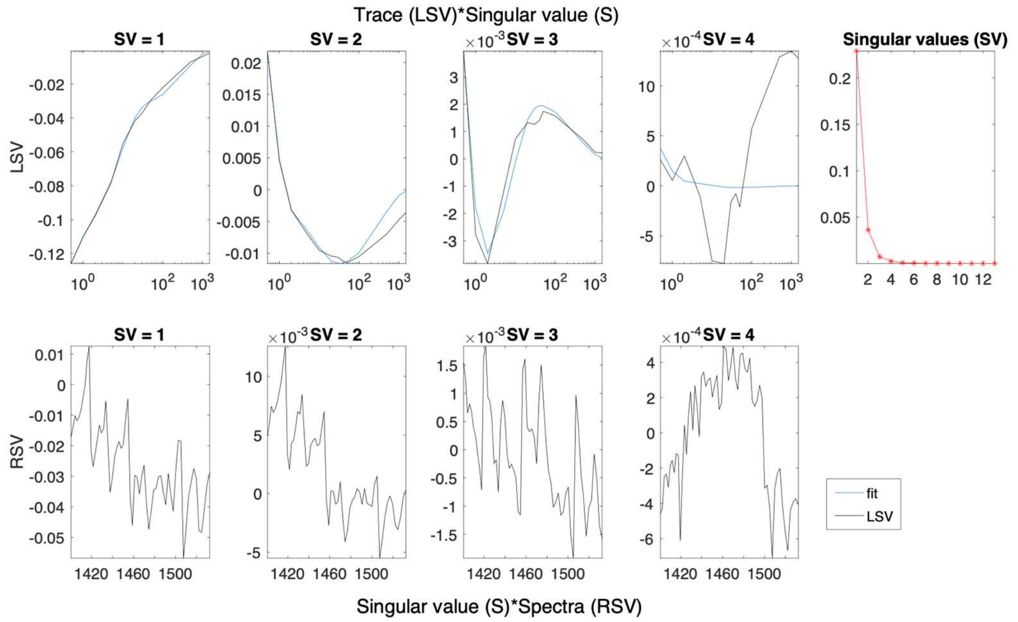

Figure S26.4. Left (LSV) and right (RSV) singular vectors with dominant singular values for the SVD analysis of spectra presented in Figure S26.1.

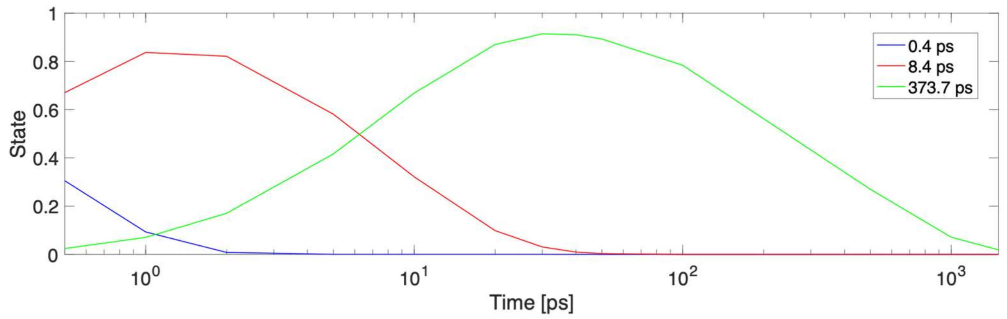

Figure S26.5. Concentration profiles for each time constant fitted to the data in Figure S26.3.

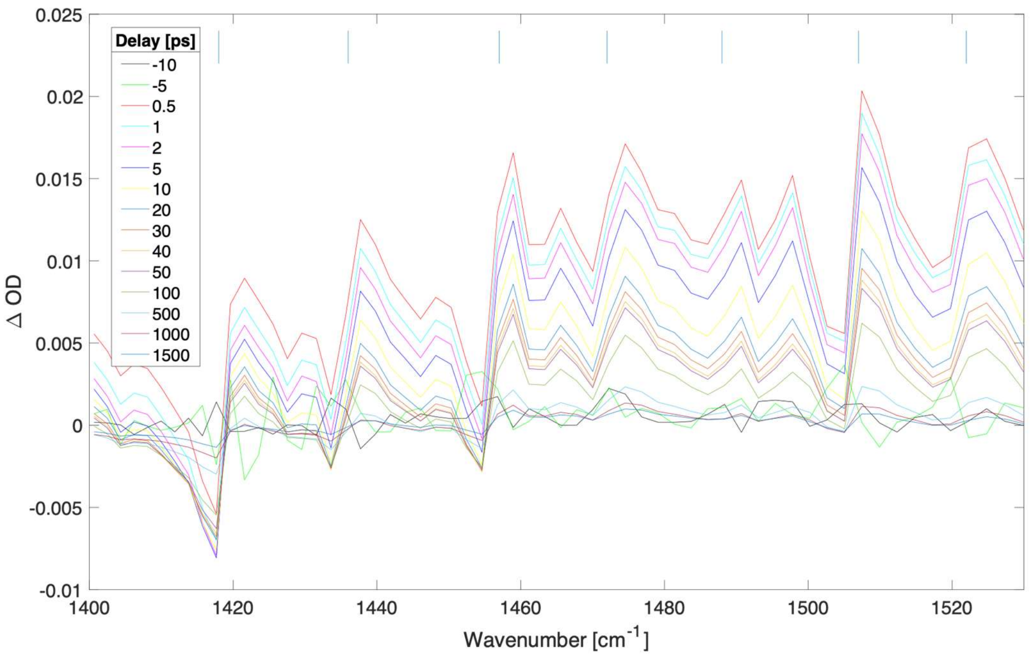

Figure S27.1. Difference transient absorption spectra at selected delays (ps) for a 300 nm thick film of $MAPbI_3$ with pump irradiance of 2.94e8 W/cm^2 (fluence of 73.6 uJ/cm^2) at 539 nm.

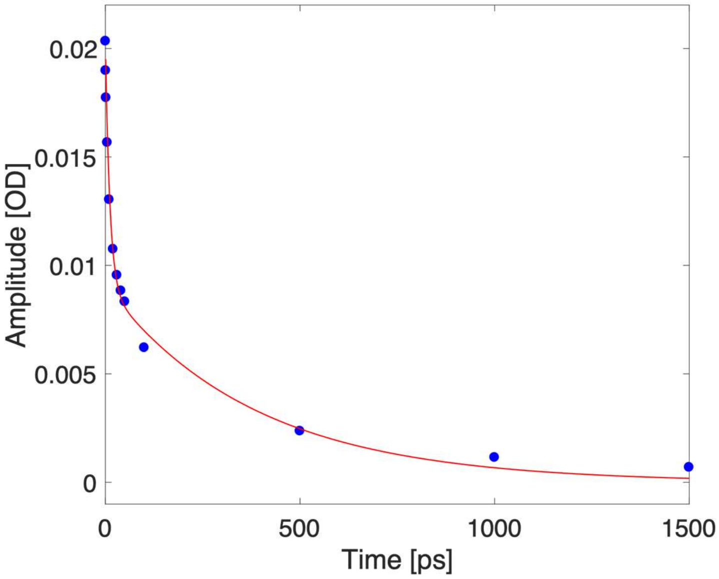

Figure S27.2. Kinetic trace taken at 1470 cm^{-1} (blue dots depict experimental data for selected delays, red curve represents a biexponential fit).

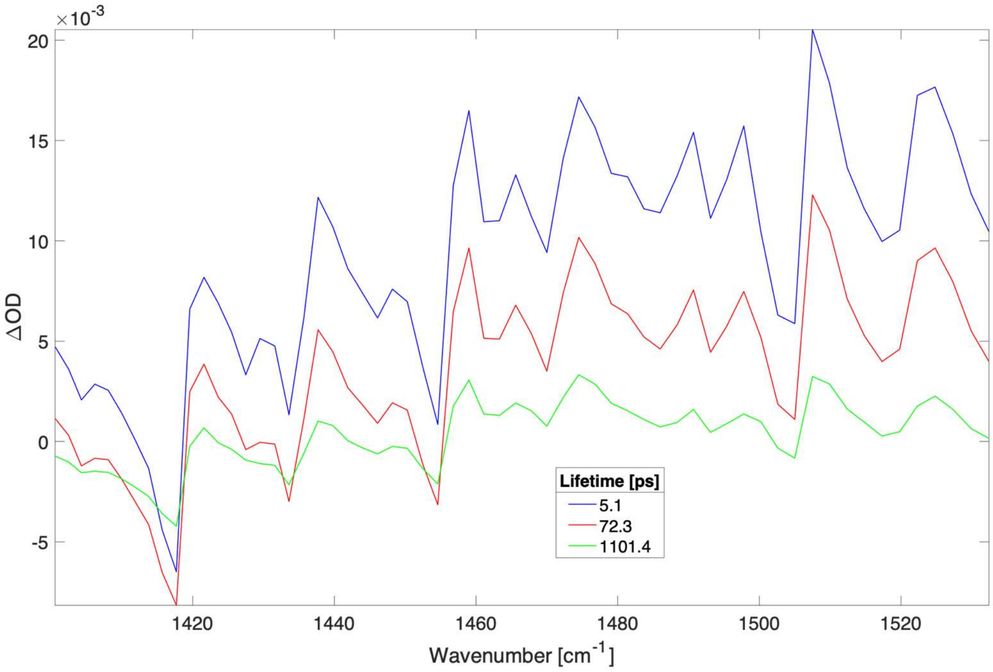

Figure S27.3. Time independent spectra of sequential three compartments global fit to spectra shown in Figure S27.1. The time constants (and contribution to data) were: 5.1 ps (57.0%), 72.3 ps (30.8%), and 1101.4 ps (12.2%).

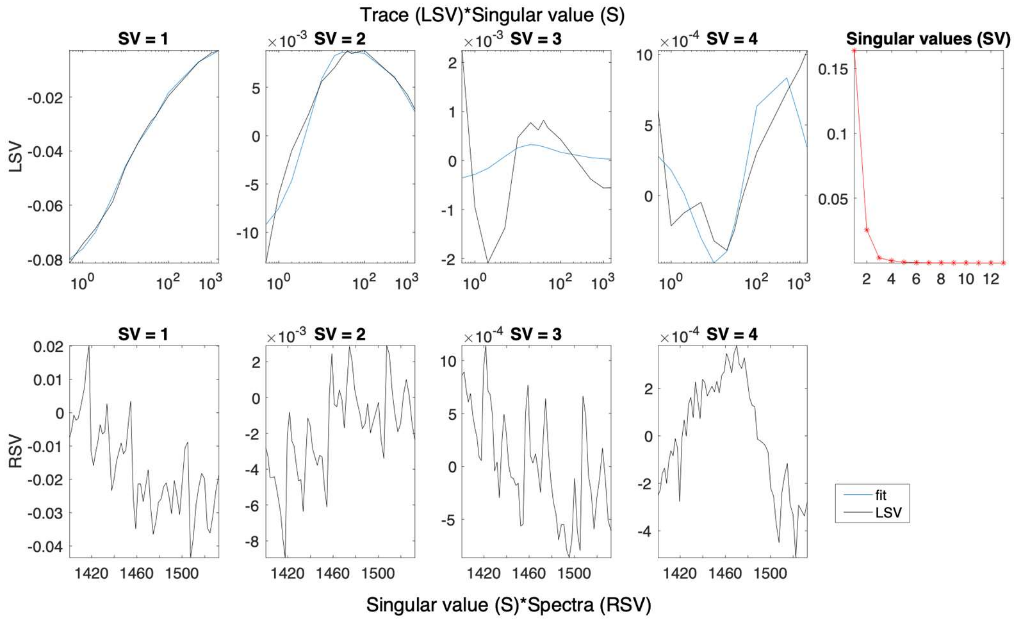

Figure S27.4. Left (LSV) and right (RSV) singular vectors with dominant singular values for the SVD analysis of spectra presented in Figure S27.1.

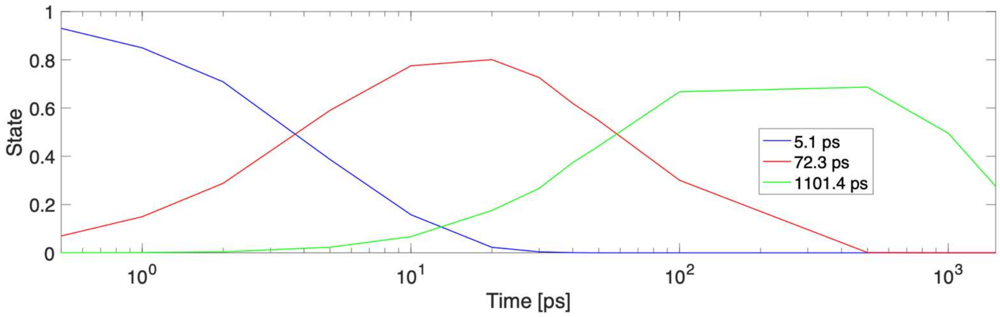

Figure S27.5. Concentration profiles for each time constant fitted to the data in Figure S27.3

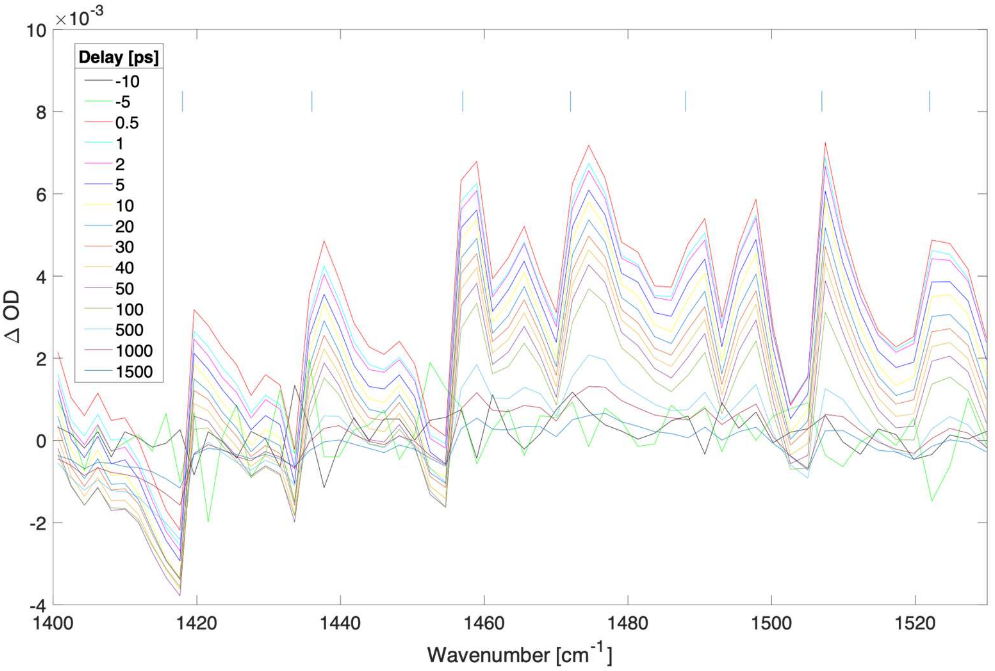

Figure S28.1. Difference transient absorption spectra at selected delays (ps) for a 300 nm thick film of $MAPbI_3$ with pump irradiance of 1.47e8 W/cm^2 (fluence of 36.8 uJ/cm^2) at 539 nm.

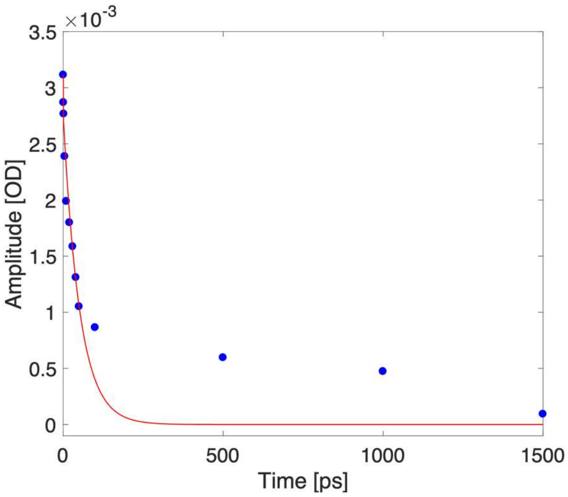

Figure S28.2. Kinetic trace taken at 1470 cm^{-1} (blue dots depict experimental data for selected delays, red curve represents a biexponential fit).

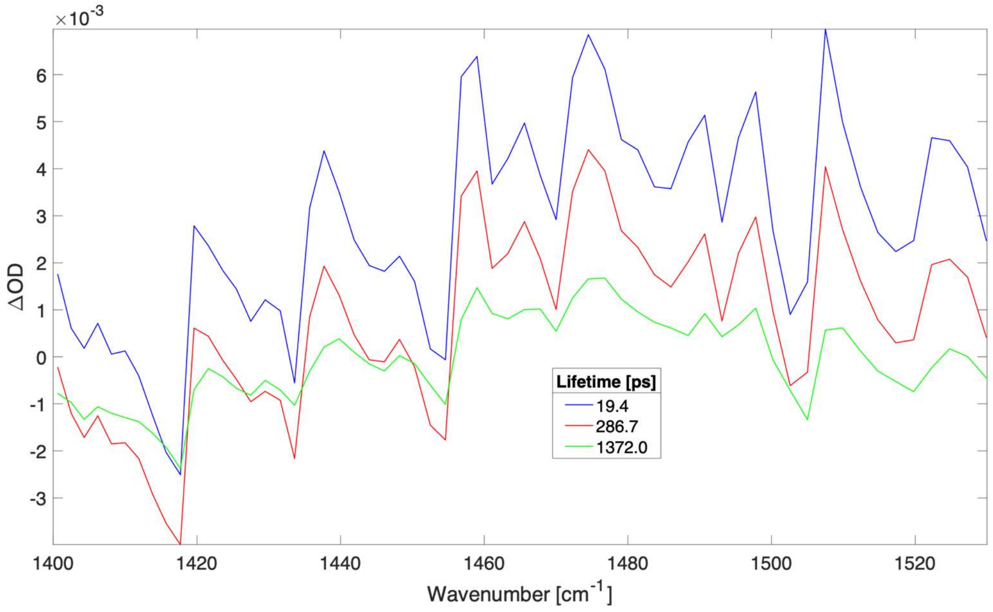

Figure S28.3. Time independent spectra of sequential three compartments global fit to spectra shown in Figure S28.1. The time constants (and contribution to data) were: 19.4 ps (48.1%), 286.7 ps (32.8%), and 1372.0 ps (19.1%).

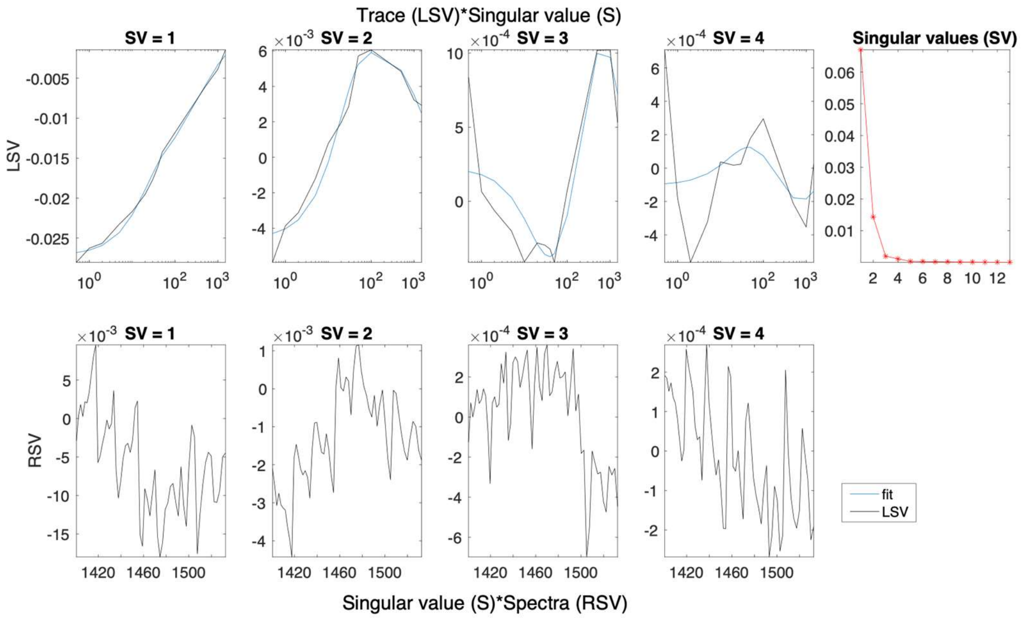

Figure S28.4. Left (LSV) and right (RSV) singular vectors with dominant singular values for the SVD analysis of spectra presented in Figure S28.1.

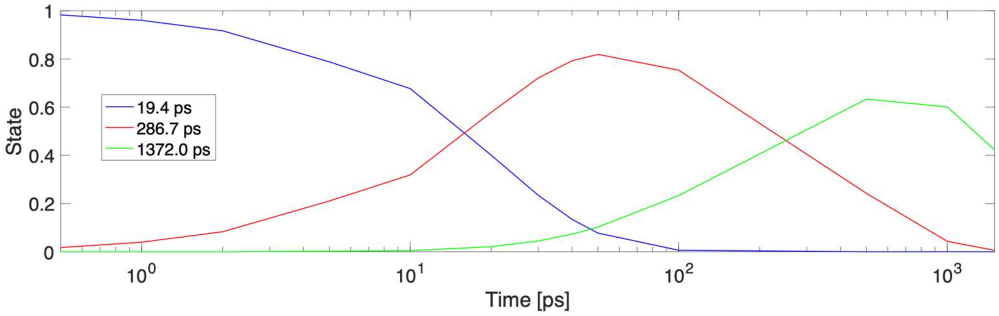

Figure S28.5. Concentration profiles for each time constant fitted to the data in Figure S28.3.

Time-resolved charge recombination analysis upon optical excitation of 500 nm thick film of $MAPbBr_3$

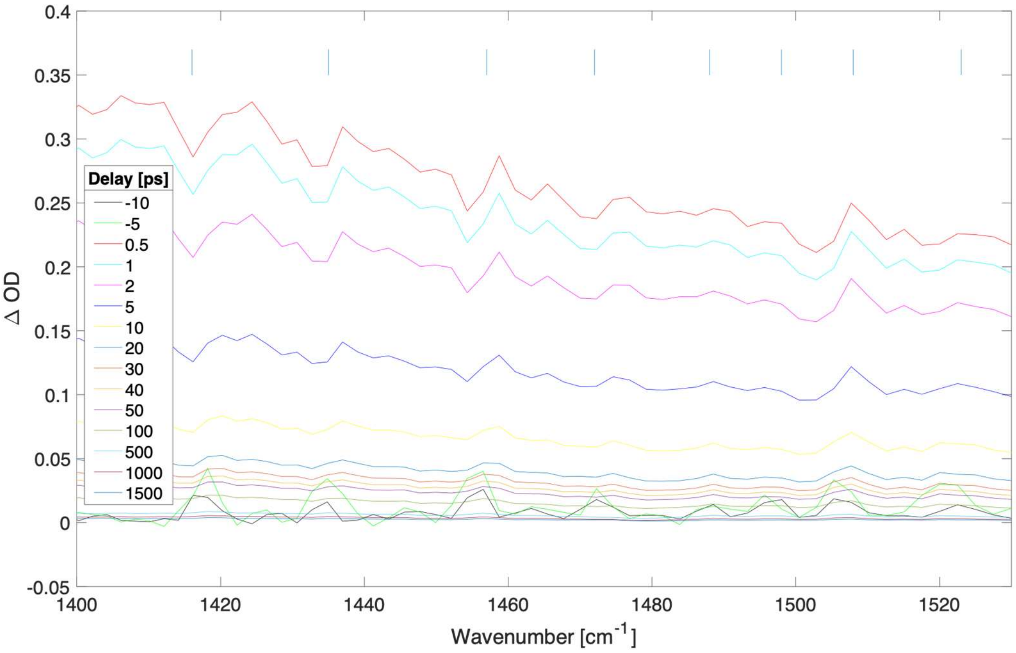

Figure S29.1. Difference transient absorption spectra at selected delays (ps) for a 500 nm thick film of $MAPbBr_3$ with pump irradiance of 7.11e10 W/cm^2 (fluence of 17,768.8 uJ/cm^2) at 539 nm.

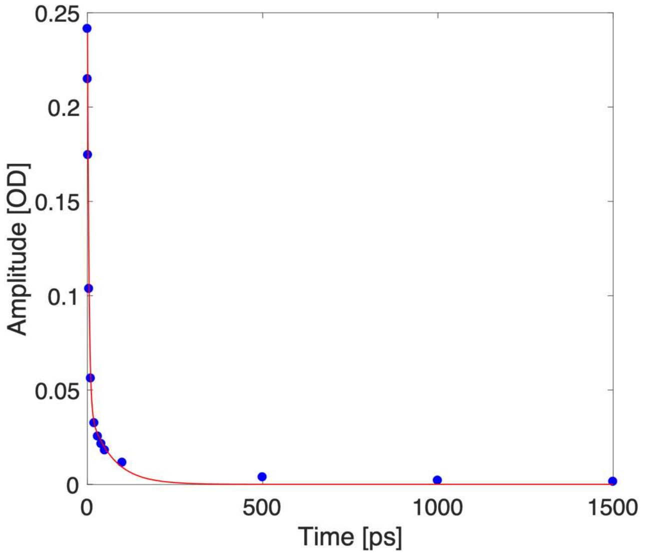

Figure S29.2. Kinetic trace taken at 1480 cm^{-1} (blue dots depict experimental data for selected delays, red curve represents a biexponential fit).

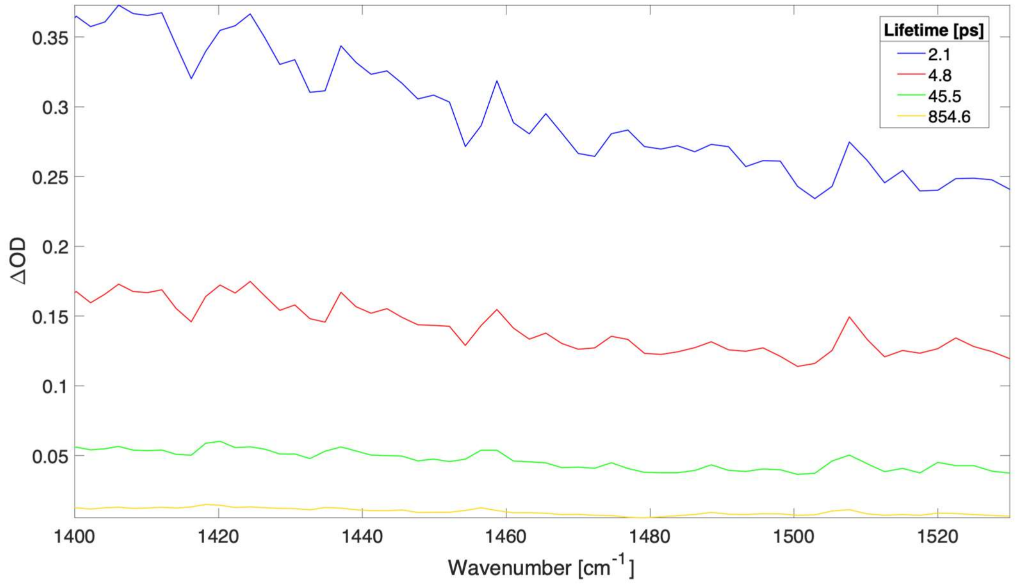

Figure S29.3. Time independent spectra of sequential three compartments global fit to spectra shown in Figure S29.1. The time constants (and contribution to data) were: 2.1 ps (57.9%), 4.8 ps (30.3%), 45.5 ps (9.3%), and 854.6 ps (2.5%).

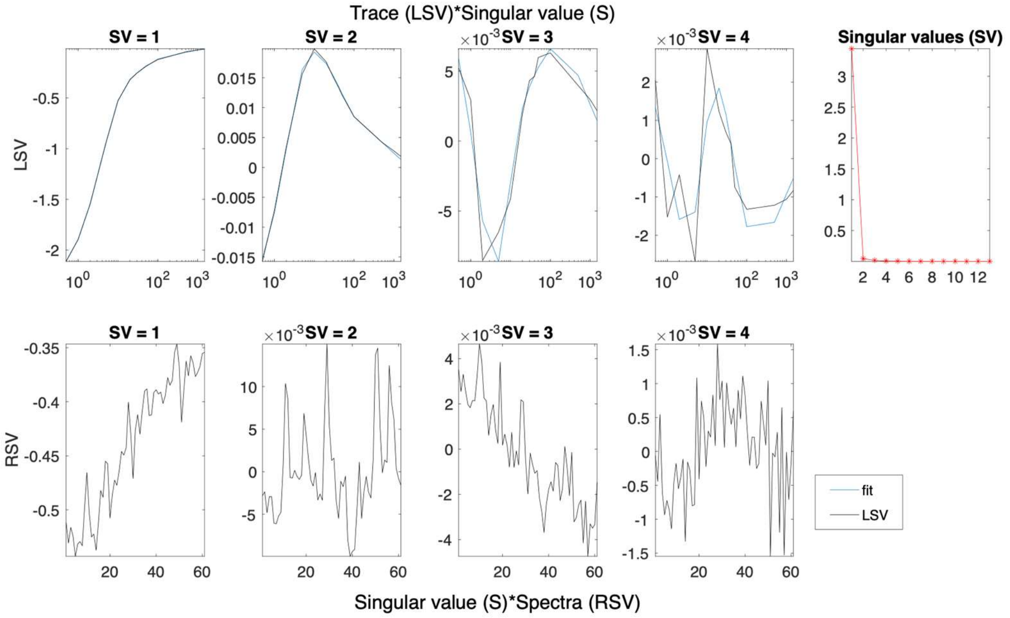

Figure S29.4. Left (LSV) and right (RSV) singular vectors with dominant singular values for the SVD analysis of spectra presented in Figure S29.1.

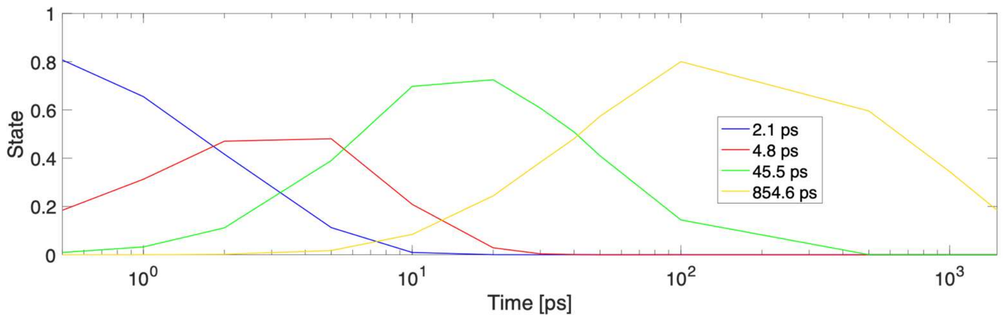

Figure S29.5. Concentration profiles for each time constant fitted to the data in Figure S29.3.

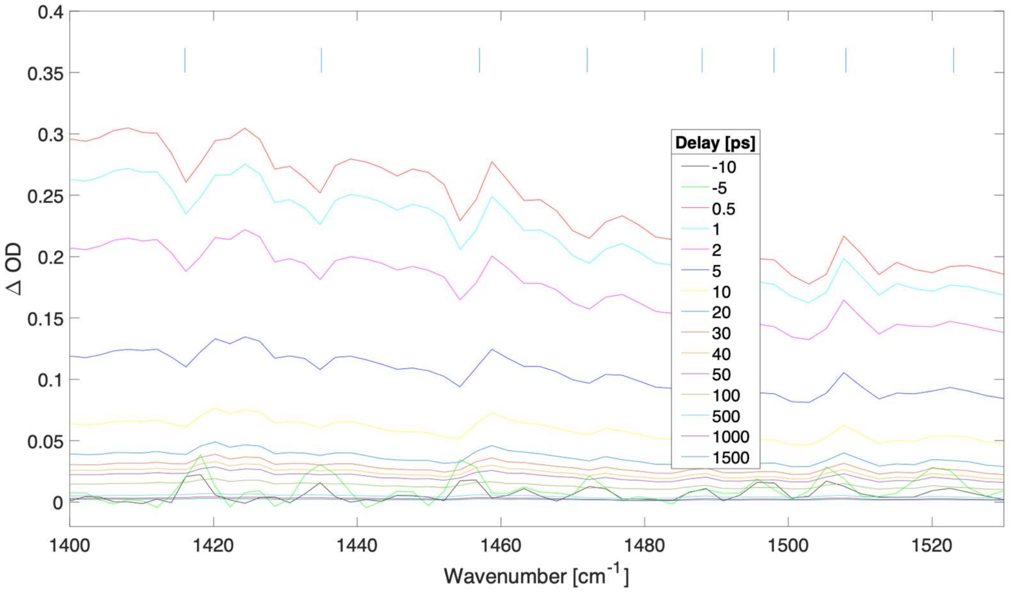

Figure S30.1. Difference transient absorption spectra at selected delays (ps) for a 500 nm thick film of $MAPbBr_3$ with pump irradiance of 4.48e10 W/cm^2 (fluence of 11,204.5 uJ/cm^2) at 539 nm.

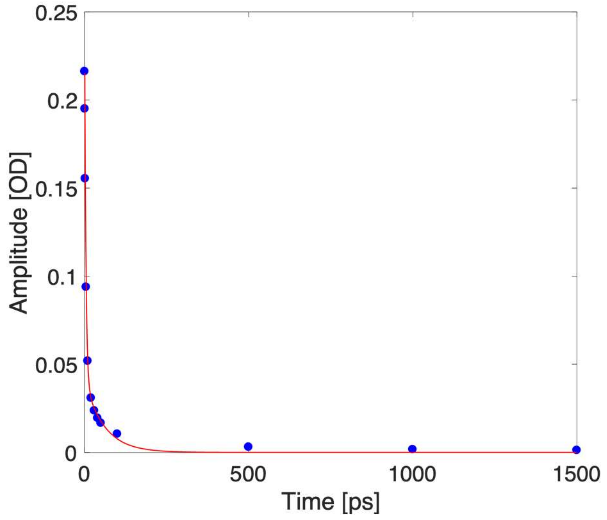

Figure S30.2. Kinetic trace taken at 1480 cm^{-1} (blue dots depict experimental data for selected delays, red curve represents a biexponential fit).

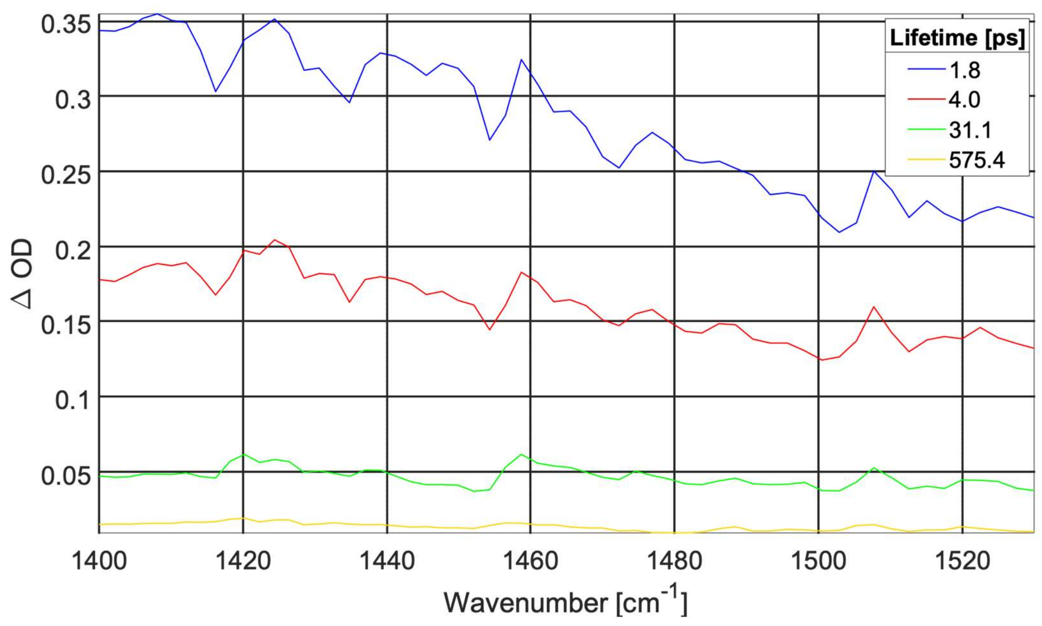

Figure S30.3. Time independent spectra of sequential three compartments global fit to spectra shown in Figure S30.1. The time constants (and contribution to data) were: 1.8 ps (73.1%), 4.0 ps (13.6%), 31.1 ps (9.5%) and 575.4 ps (3.8%).

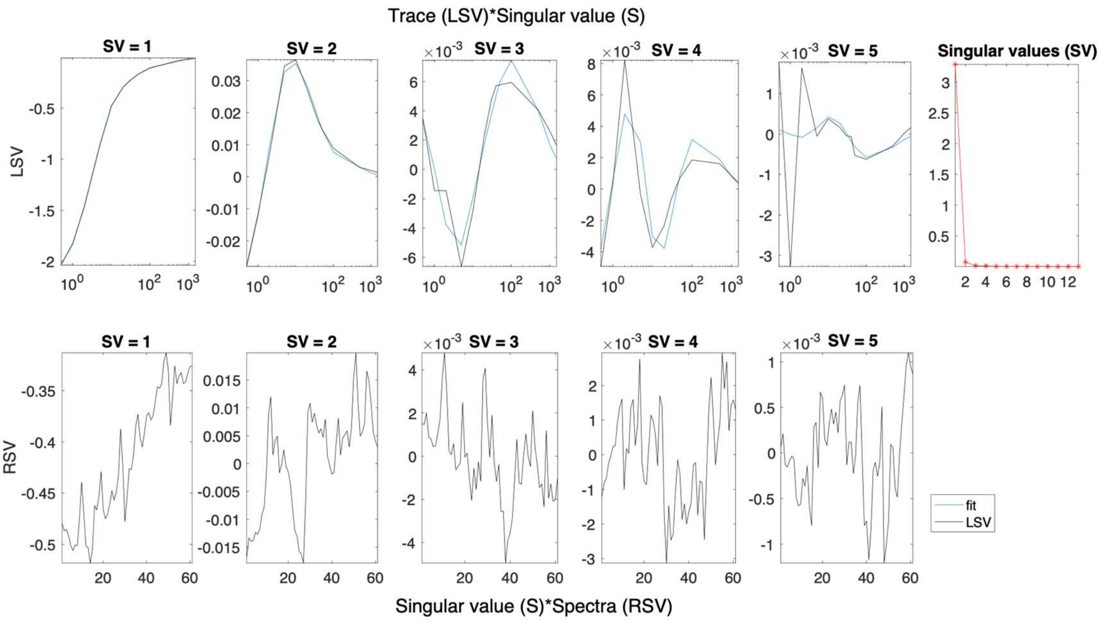

Figure S30.4. Left (LSV) and right (RSV) singular vectors with dominant singular values for the SVD analysis of spectra presented in Figure S30.1.

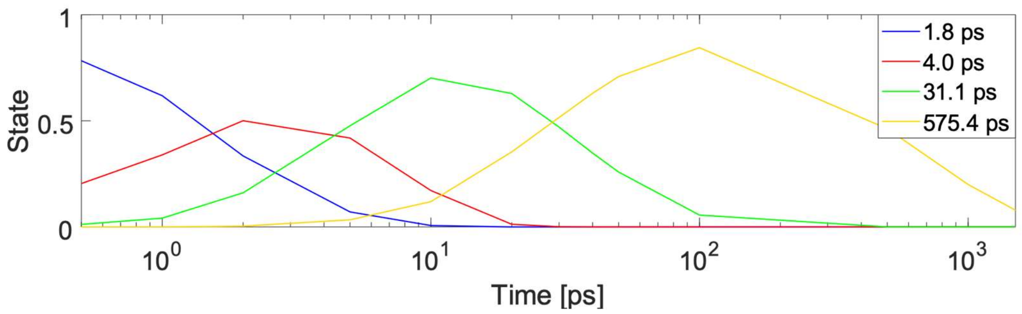

Figure S30.5. Concentration profiles for each time constant fitted to the data in Figure S30.3.

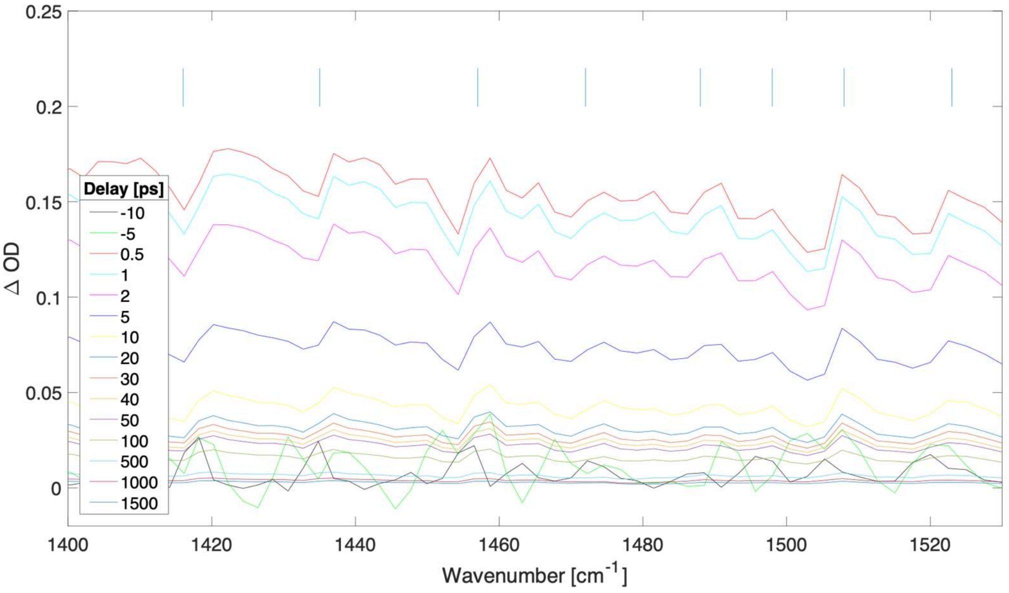

Figure S31.1. Difference transient absorption spectra at selected delays (ps) for a 500 nm thick film of $MAPbBr_3$ with pump irradiance of 2.83e10 W/cm^2 (fluence of 7,073.6 uJ/cm^2) at 539 nm.

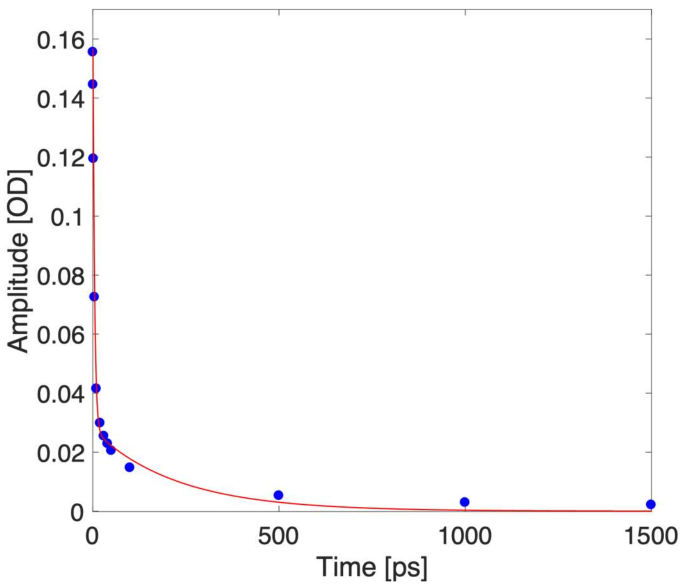

Figure S31.2. Kinetic trace taken at 1480 cm^{-1} (blue dots depict experimental data for selected delays, red curve represents a biexponential fit).

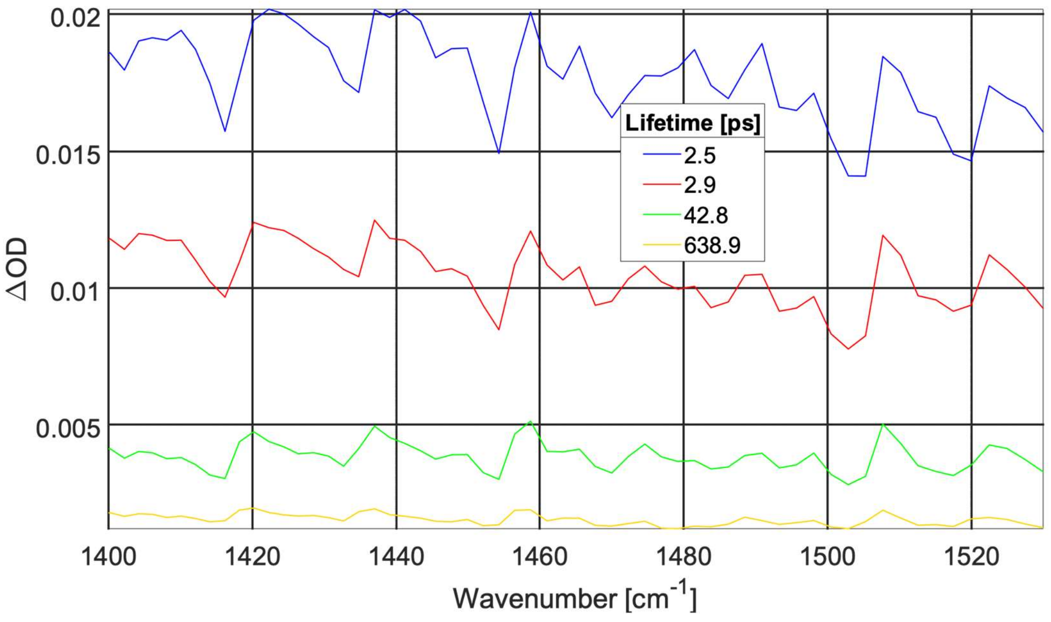

Figure S31.3. Time independent spectra of sequential three compartments global fit to spectra shown in Figure S31.1. The time constants (and contribution to data) were: 2.5 ps (50.7%), 2.9 ps (32.3%), 42.8 ps (11.9%), and 638.9 ps (5.1%).

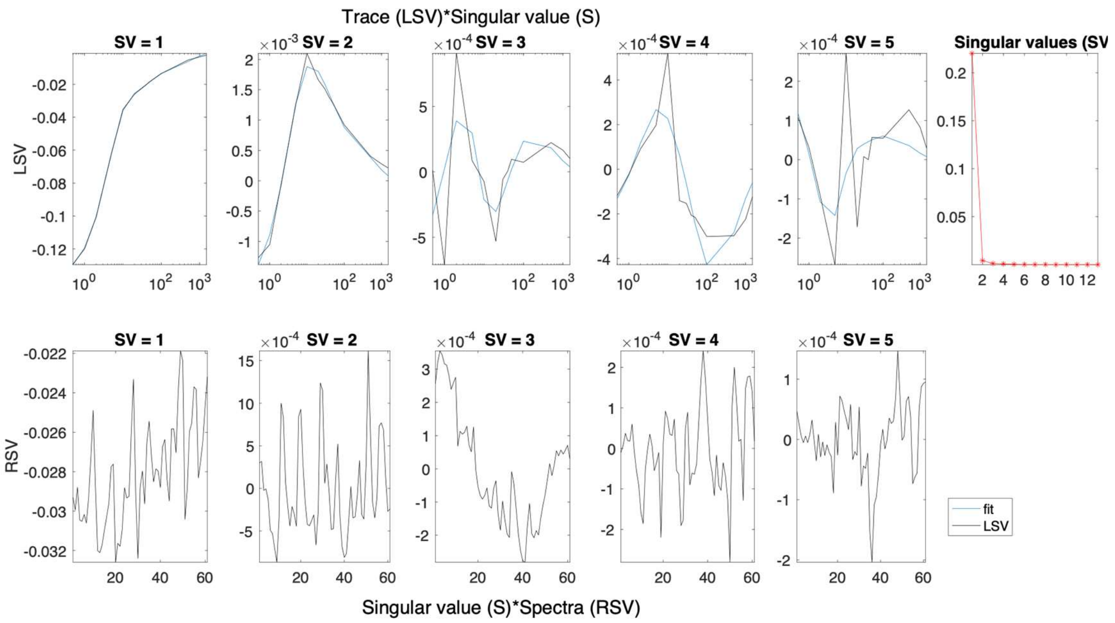

Figure S31.4. Left (LSV) and right (RSV) singular vectors with dominant singular values for the SVD analysis of spectra presented in Figure S31.1.

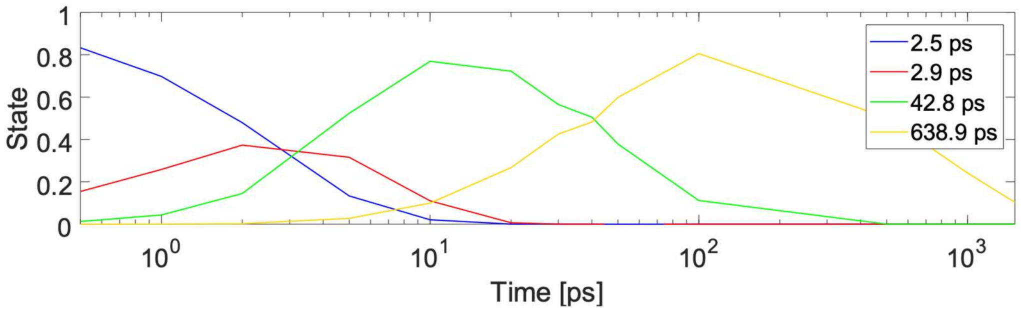

Figure S31.5. Concentration profiles for each time constant fitted to the data in Figure S31.3.

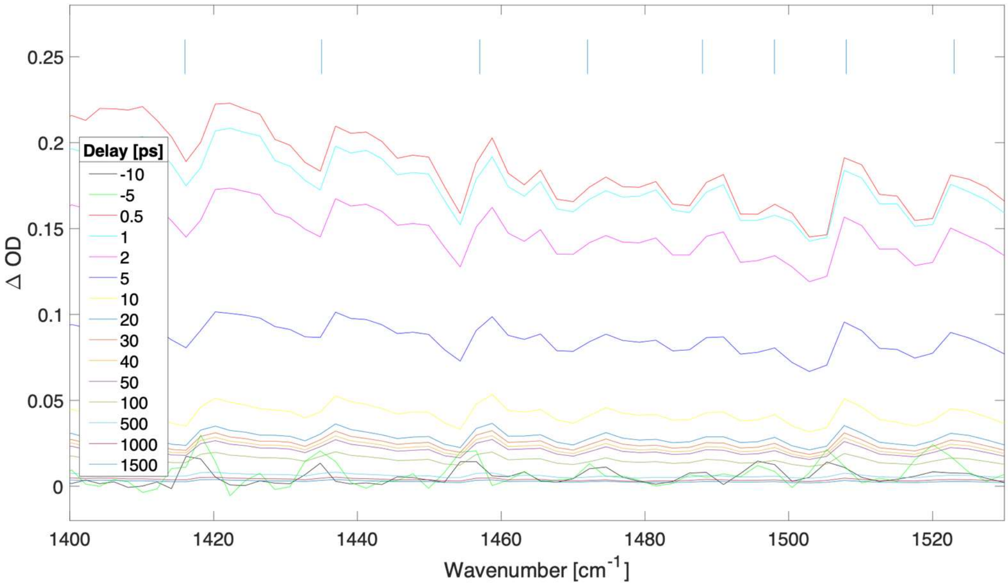

Figure S32.1. Difference transient absorption spectra at selected delays (ps) for a 500 nm thick film of $MAPbBr_3$ with pump irradiance of 1.78.9e10 W/cm^2 (fluence of 4,470.5 uJ/cm^2) at 539 nm.

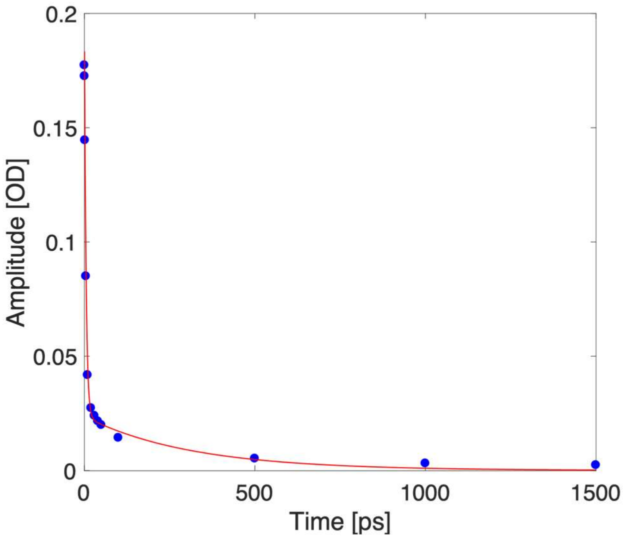

Figure S32.2. Kinetic trace taken at 1480 cm^{-1} (blue dots depict experimental data for selected delays, red curve represents a biexponential fit).

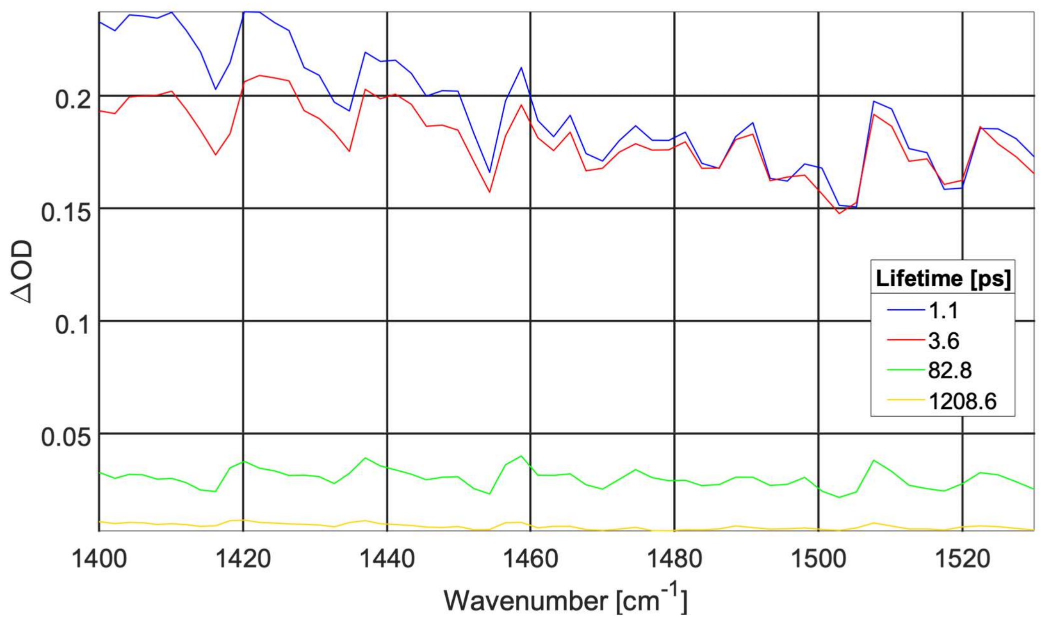

Figure S32.3. Time independent spectra of sequential three compartments global fit to spectra shown in Figure S32.1. The time constants (and contribution to data) were: 1.1 ps (46.2%), 3.6 ps (43.8%), 82.8 ps (7.7%), and 1208.6 ps (2.3%).

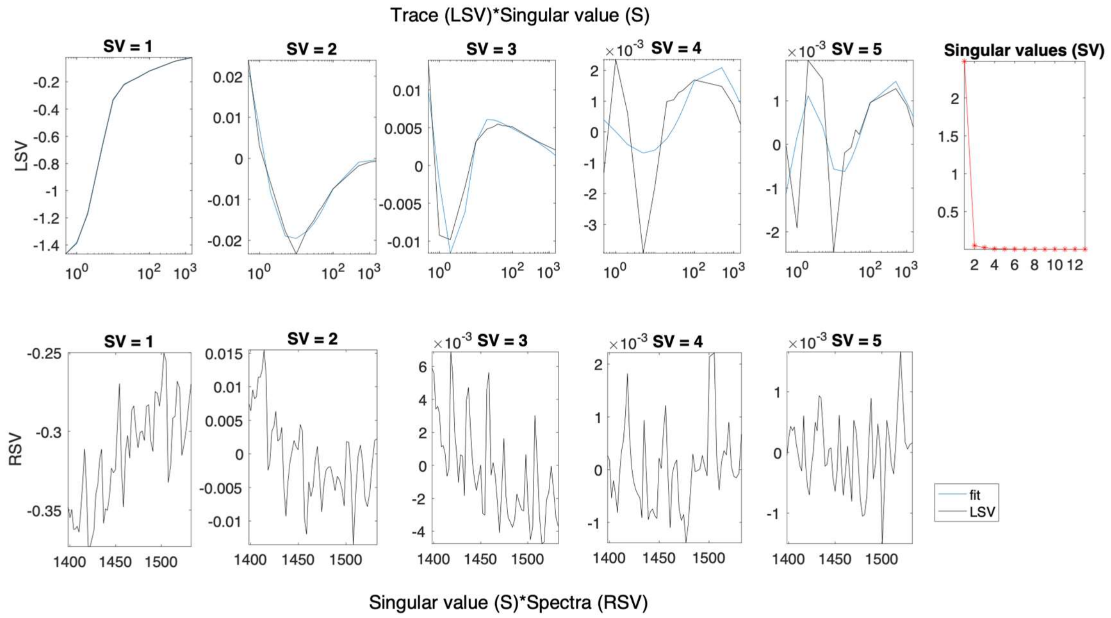

Figure S32.4. Left (LSV) and right (RSV) singular vectors with dominant singular values for the SVD analysis of spectra presented in Figure S32.1.

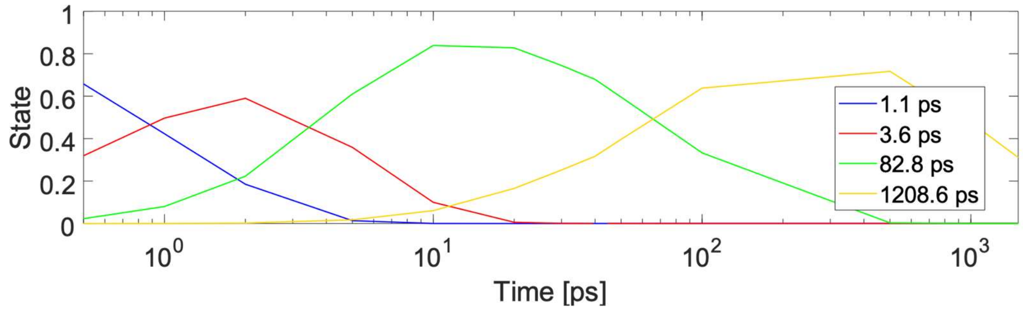

Figure S32.5. Concentration profiles for each time constant fitted to the data in Figure S32.3.

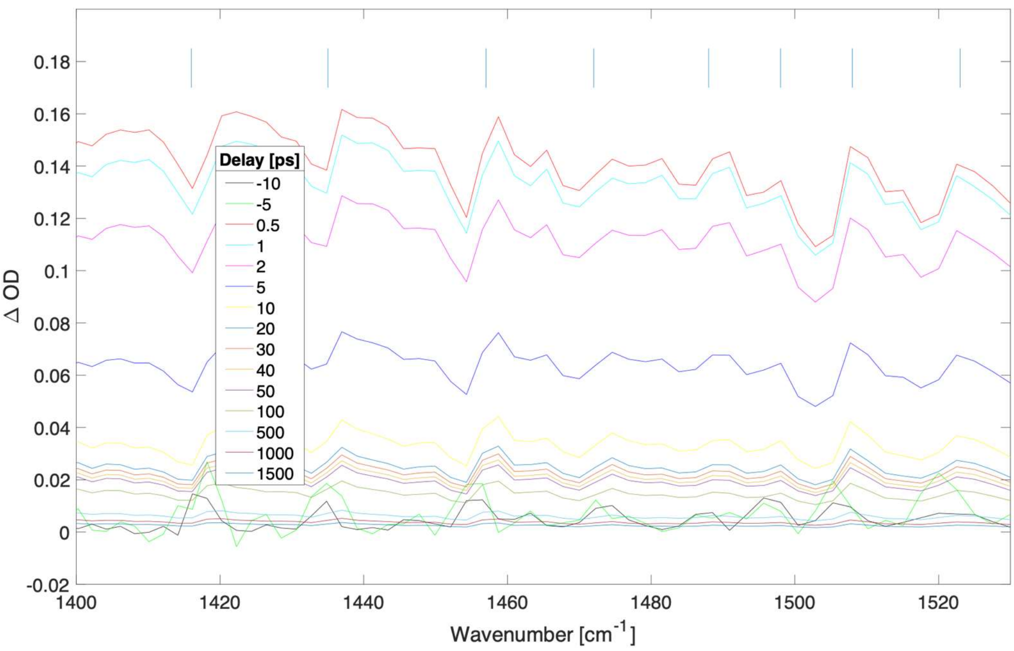

Figure S33.1. Difference transient absorption spectra at selected delays (ps) for a 500 nm thick film of $MAPbBr_3$ with pump irradiance of 1.13e10 W/cm^2 (fluence of 2,829.4uJ/cm^2) at 539 nm.

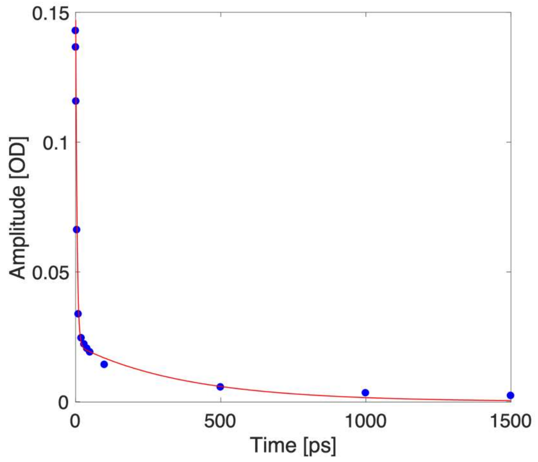

Figure S33.2. Kinetic trace taken at 1480 cm^{-1} (blue dots depict experimental data for selected delays, red curve represents a biexponential fit).

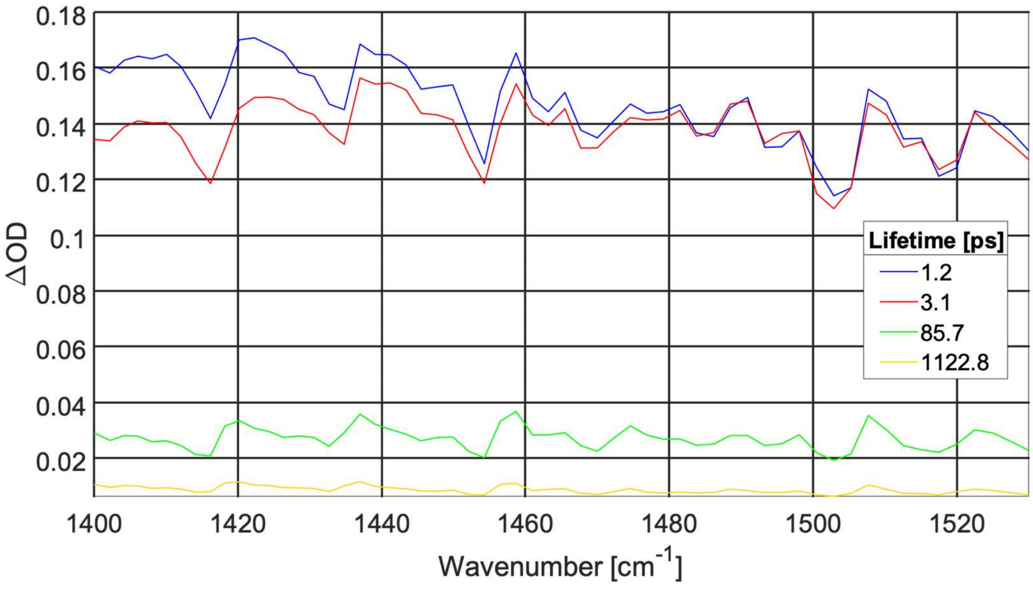

Figure S33.3. Time independent spectra of sequential three compartments global fit to spectra shown in Figure S33.1. The time constants (and contribution to data) were: 1.2 ps (45.2%), 3.1 ps (42.9%), 85.7 ps (8.9%), and 1122.8 ps (3.0%).

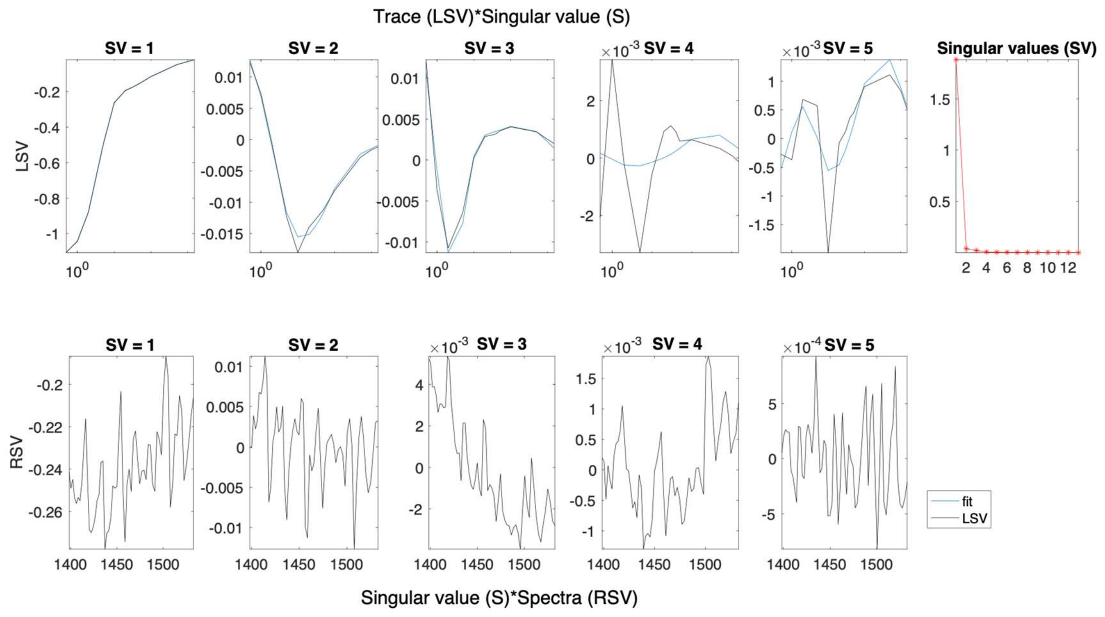

Figure S33.4. Left (LSV) and right (RSV) singular vectors with dominant singular values for the SVD analysis of spectra presented in Figure S33.1.

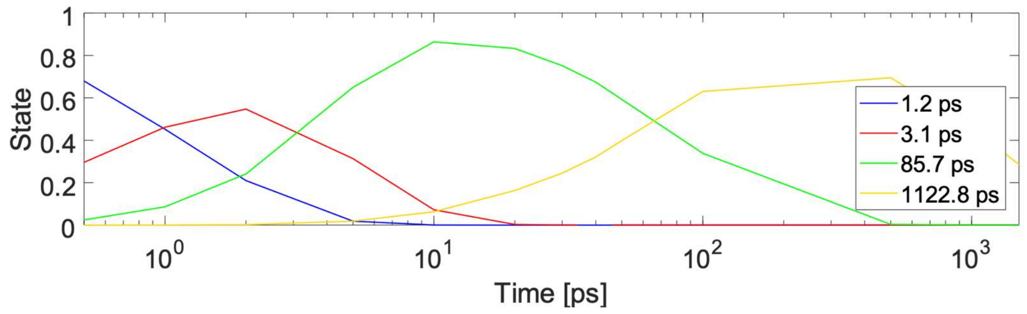

Figure S33.5. Concentration profiles for each time constant fitted to the data in Figure S33.3.

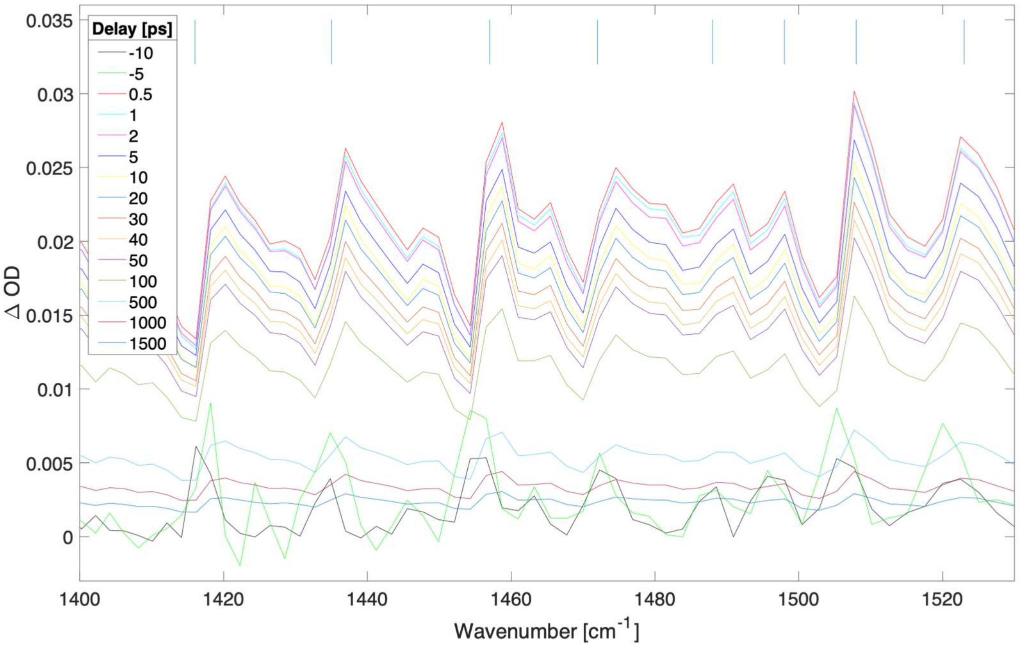

Figure S34.1. Difference transient absorption spectra at selected delays (ps) for a 500 nm thick film of $MAPbBr_3$ with pump irradiance of 2.83e9 W/cm^2 (fluence of 707.4 uJ/cm^2) at 539 nm.

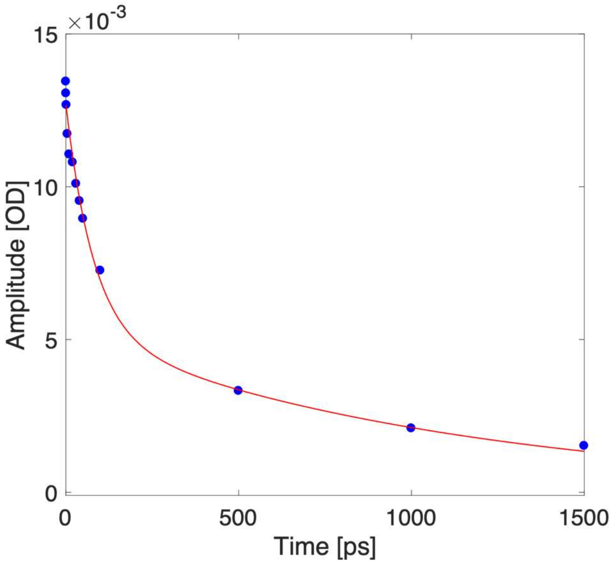

Figure S34.2. Kinetic trace taken at 1480 cm^{-1} (blue dots depict experimental data for selected delays, red curve represents a biexponential fit).

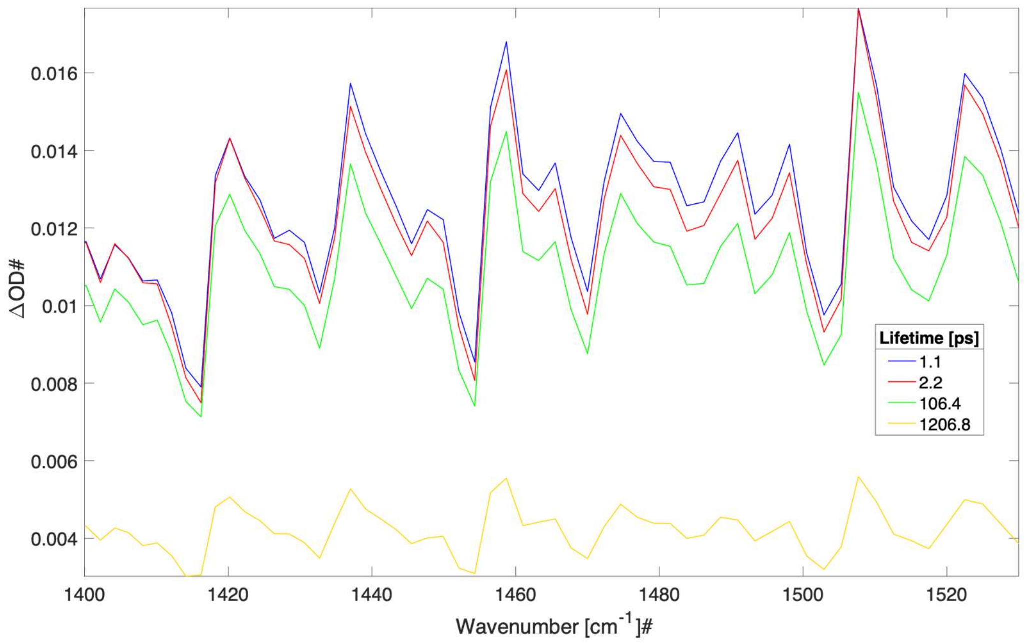

Figure S34.3. Time independent spectra of sequential three compartments global fit to spectra shown in Figure S34.1. The time constants (and contribution to data) were: 1.1 ps (31.7%), 2.2 ps (30.2%), 106.4 ps (27.0%), and 1206.8 ps (11.1%).

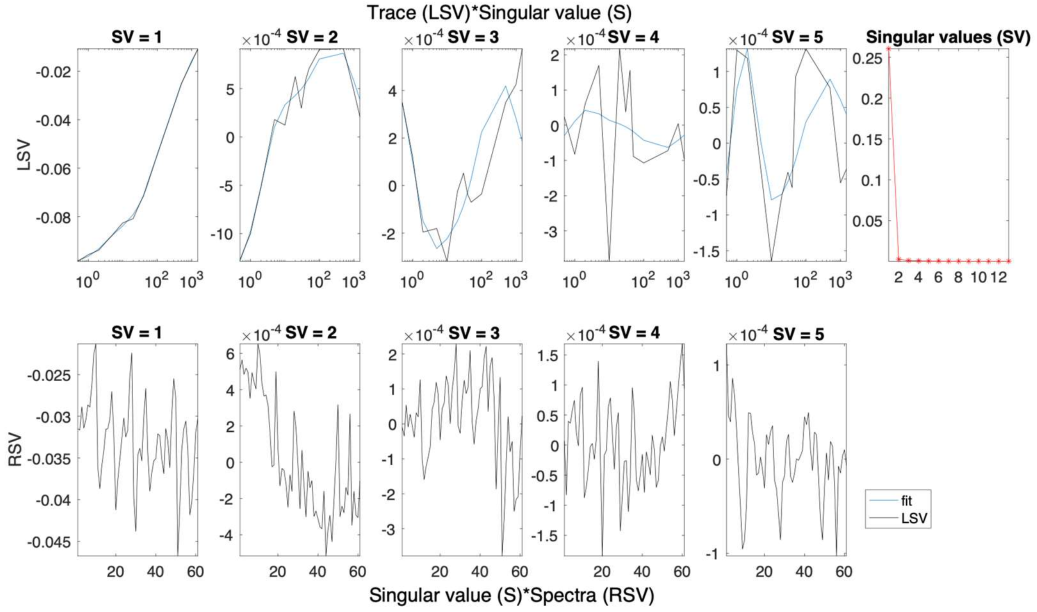

Figure S34.4. Left (LSV) and right (RSV) singular vectors with dominant singular values for the SVD analysis of spectra presented in Figure S34.1

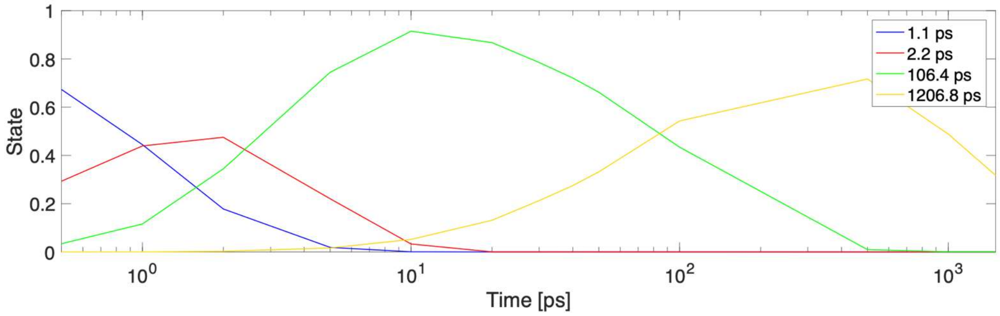

Figure S34.5. Concentration profiles for each time constant fitted to the data in Figure S34.3.

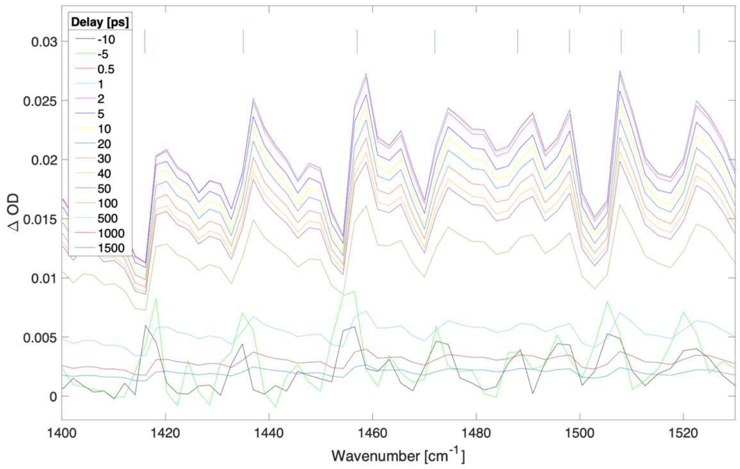

Figure S35.1. Difference transient absorption spectra at selected delays (ps) for a 500 nm thick film of $MAPbBr_3$ with pump irradiance of 2.32 e9 W/cm^2 (fluence of 580.0 uJ/cm^2) at 539 nm.

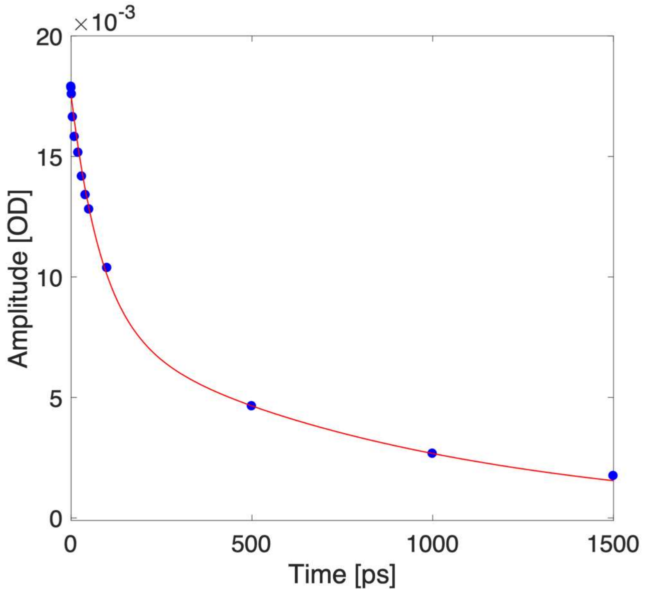

Figure S35.2. Kinetic trace taken at 1480 cm^{-1} (blue dots depict experimental data for selected delays, red curve represents a biexponential fit).

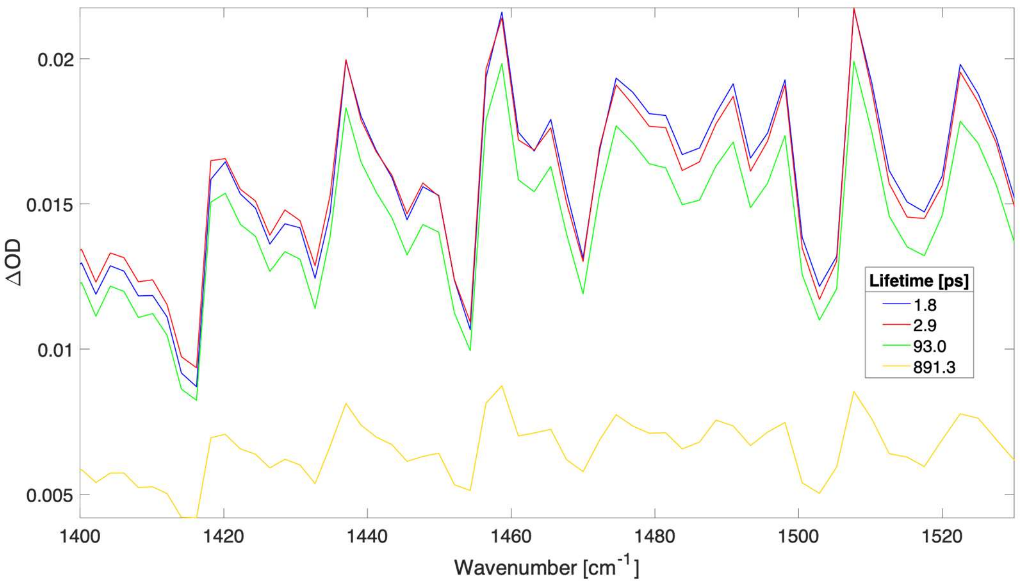

Figure S35.3. Time independent spectra of sequential three compartments global fit to spectra shown in Figure S35.1. The time constants (and contribution to data) were: 1.8 ps (30.0%), 2.9 ps (29.8%), 93.0 ps (27.1%), and 891.3 ps (13.1%).

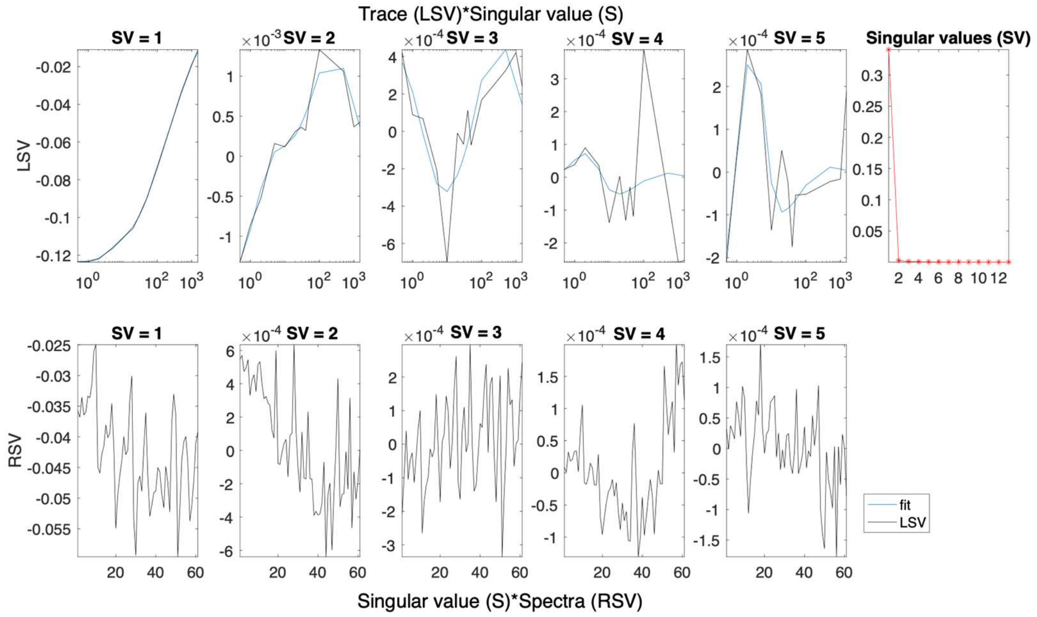

Figure S35.4. Left (LSV) and right (RSV) singular vectors with dominant singular values for the SVD analysis of spectra presented in Figure S35.1.

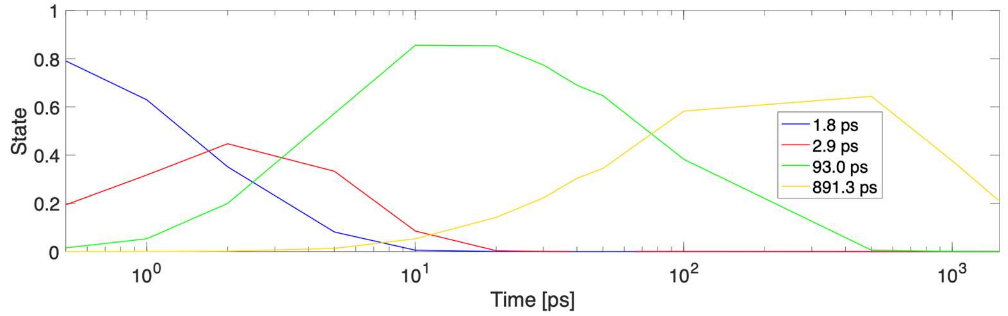

Figure S35.5. Concentration profiles for each time constant fitted to the data in Figure S35.3.

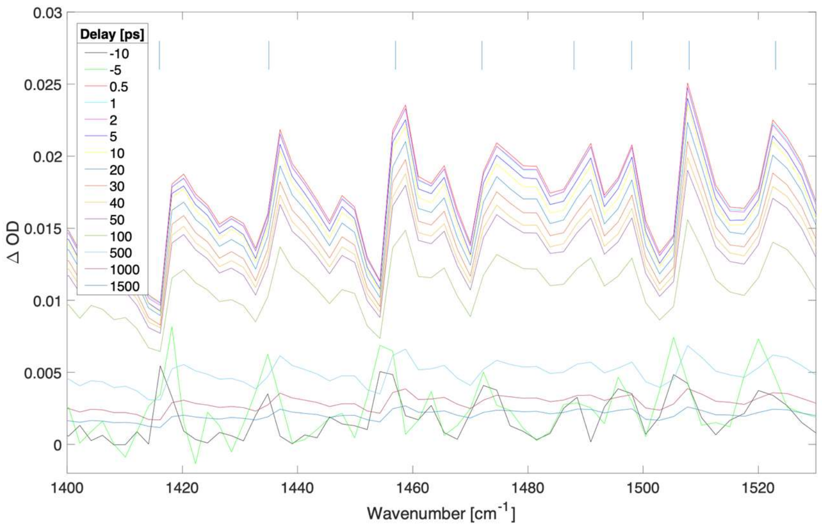

Figure S36.1. Difference transient absorption spectra at selected delays (ps) for a 500 nm thick film of $MAPbBr_3$ with pump irradiance of 1.86e9 W/cm^2 (fluence of 464.0 uJ/cm^2) at 539 nm.

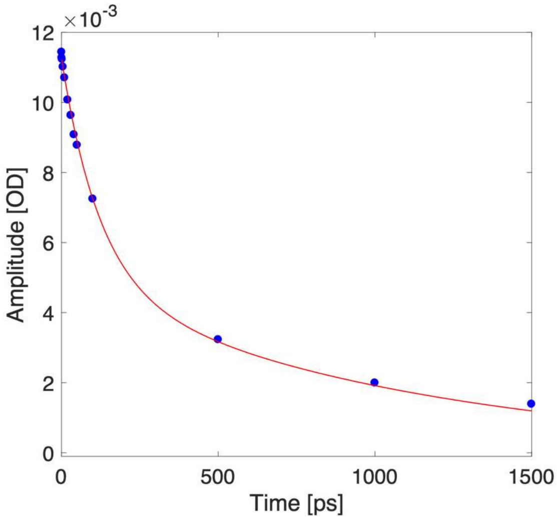

Figure S36.2. Kinetic trace taken at 1480 cm^{-1}. (blue dots depict experimental data for selected delays, red curve represents a biexponential fit)

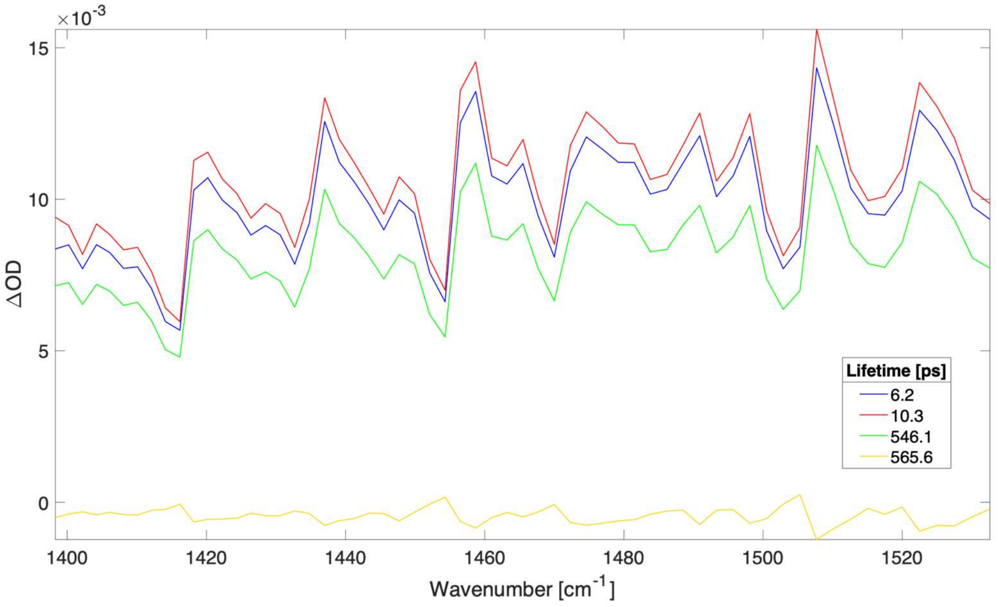

Figure S36.3. Time independent spectra of sequential three compartments global fit to spectra shown in Figure S36.1. The time constants (and contribution to data) were: 6.2 ps (34.0%), 10.3 ps (36.4%), 546.1 ps (28.1%), and 565.6 ps (1.5%).

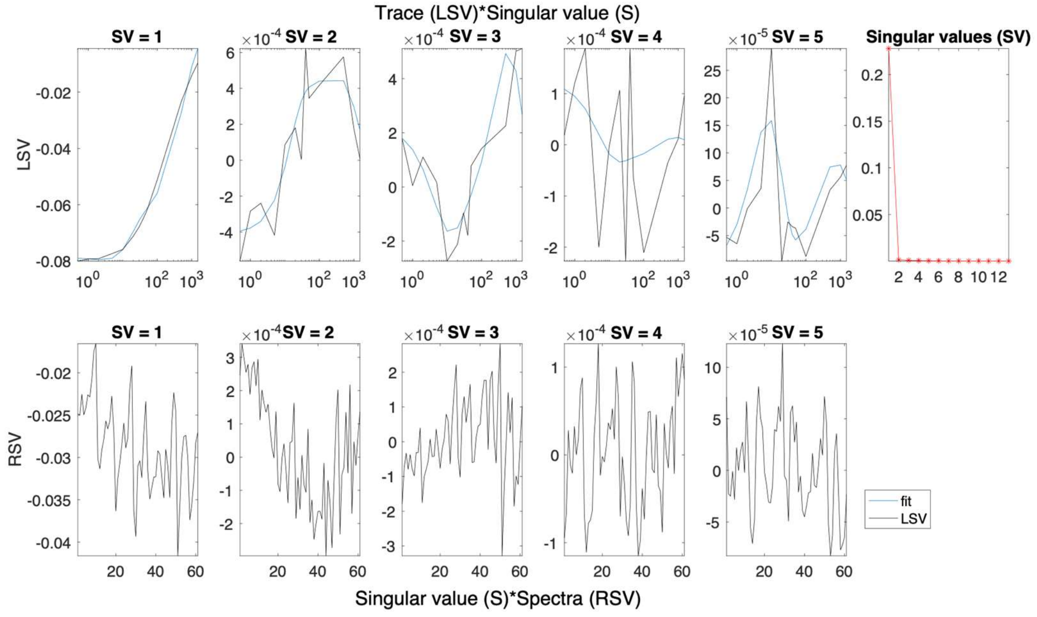

Figure S36.4. Left (LSV) and right (RSV) singular vectors with dominant singular values for the SVD analysis of spectra presented in Figure S36.1.

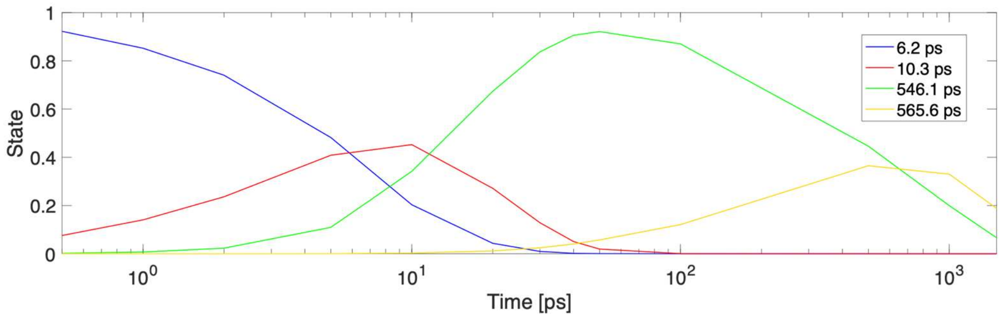

Figure S36.5. Concentration profiles for each time constant fitted to the data in Figure S36.3.

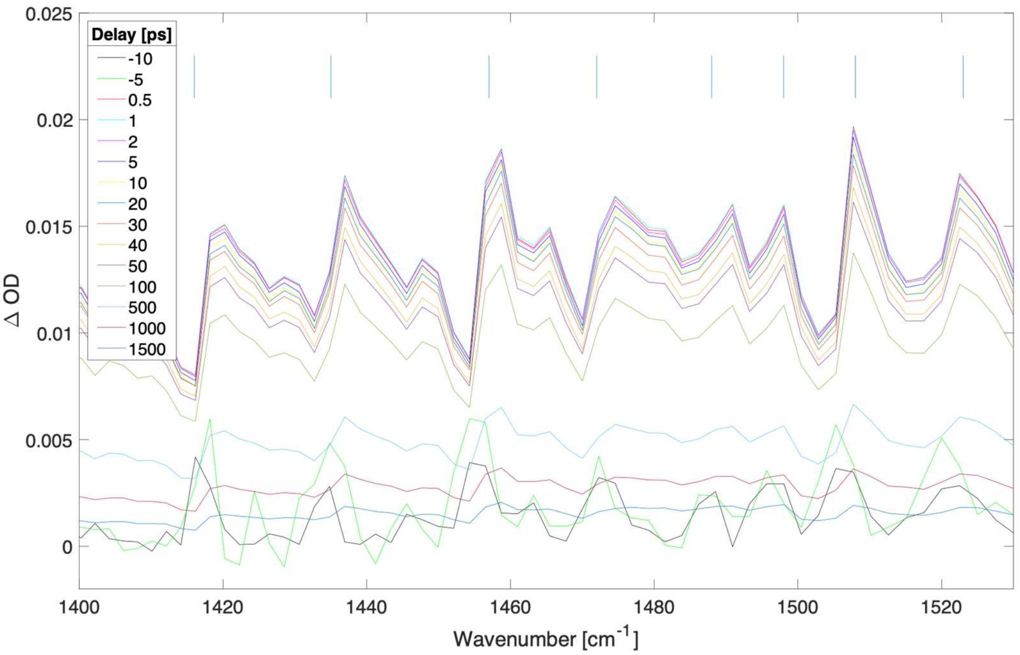

Figure S37.1. Difference transient absorption spectra at selected delays (ps) for a 500 nm thick film of $MAPbBr_3$ with pump irradiance of 1.47e9 W/cm^2 (fluence of 367.8 uJ/cm^2) at 539 nm.

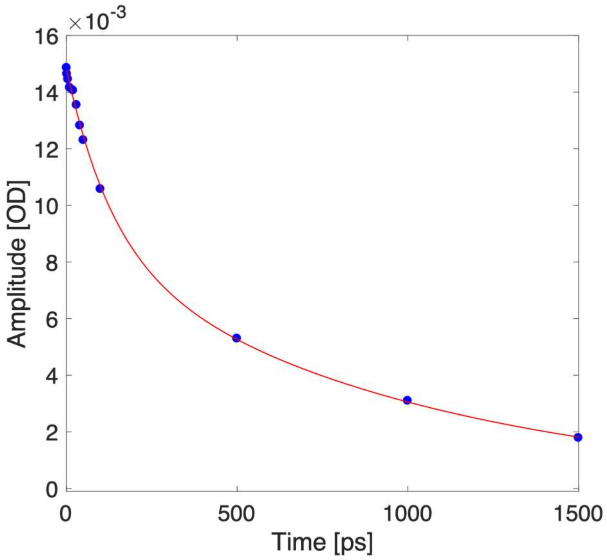

Figure S37.2. Kinetic trace taken at 1480 cm^{-1} (blue dots depict experimental data for selected delays, red curve represents a biexponential fit).

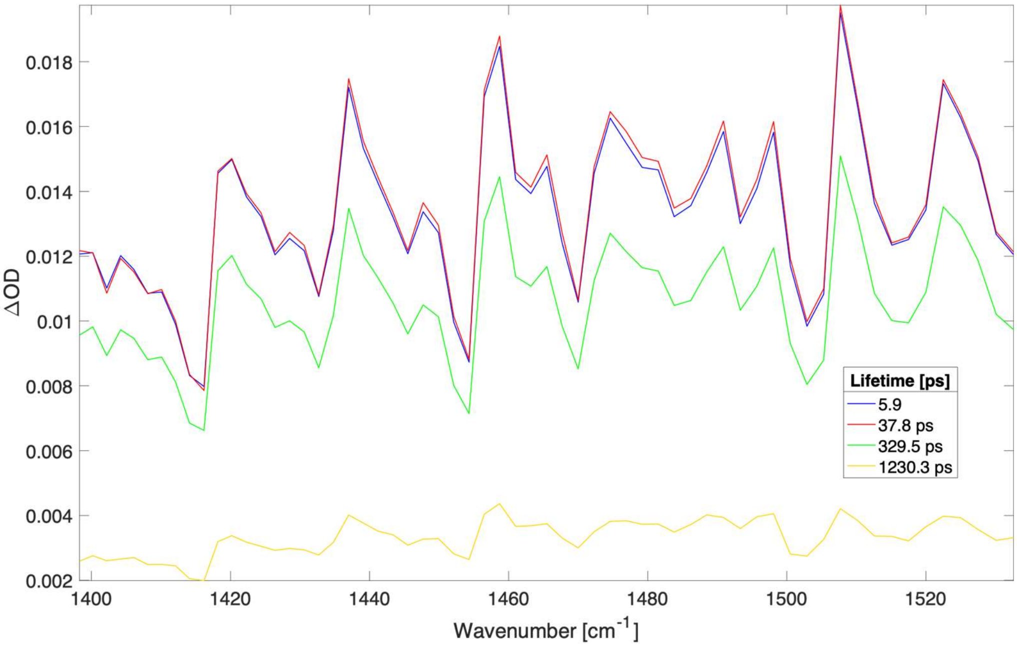

Figure S37.3. Time independent spectra of sequential three compartments global fit to spectra shown in Figure S37.1. The time constants (and contribution to data) were: 5.9 ps (32.0%), 37.8 ps (32.3%), 329.5 ps (26.2%), and 1230.3 ps (9.5%).

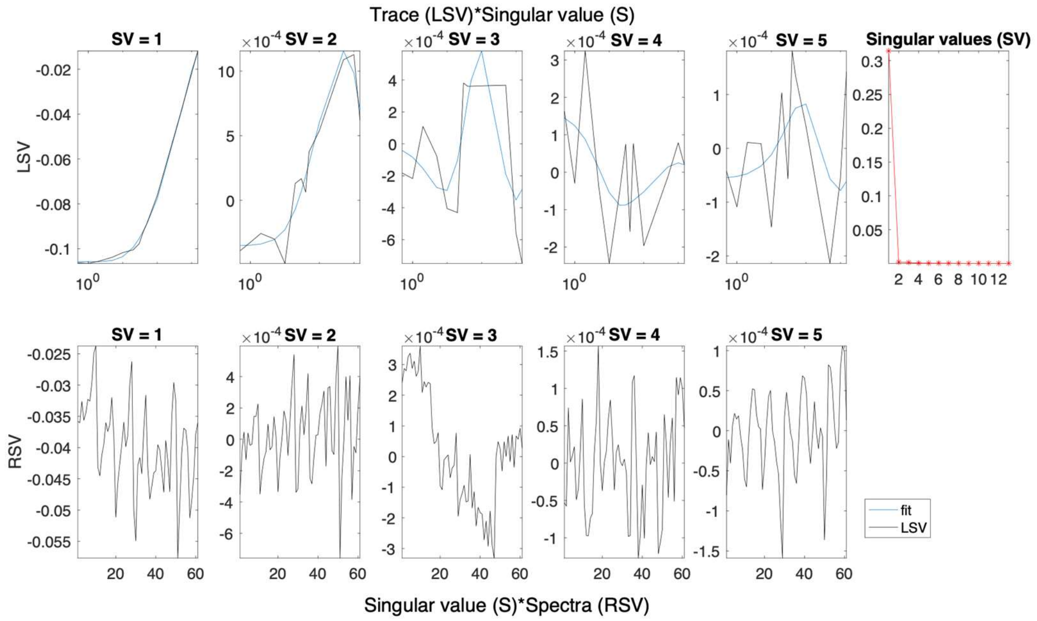

Figure S37.4. Left (LSV) and right (RSV) singular vectors with dominant singular values for the SVD analysis of spectra presented in Figure S37.1.

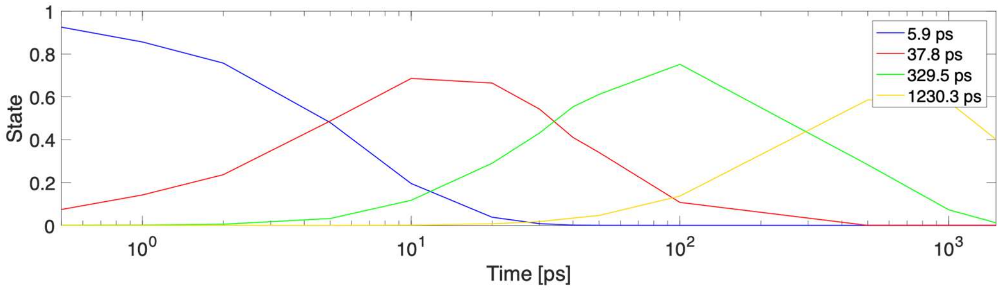

Figure S37.5. Concentration profiles for each time constant fitted to the data in Figure S37.3

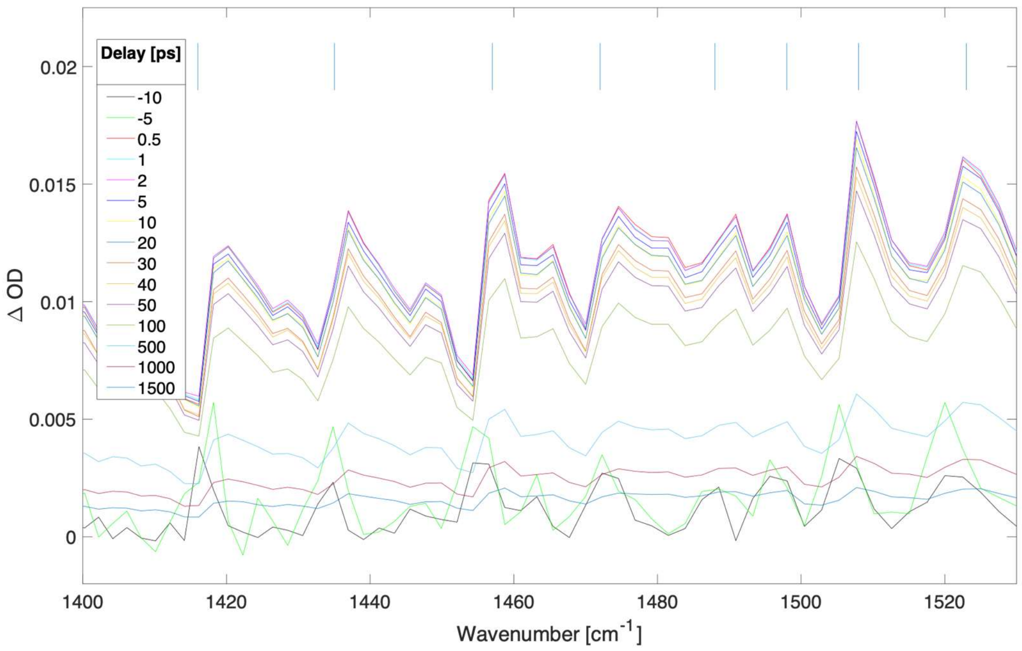

Figure S38.1. Difference transient absorption spectra at selected delays (ps) for a 500 nm thick film of $MAPbBr_3$ with pump irradiance of 1.18e9 W/cm^2 (fluence of 294.2 uJ/cm^2) at 539 nm.

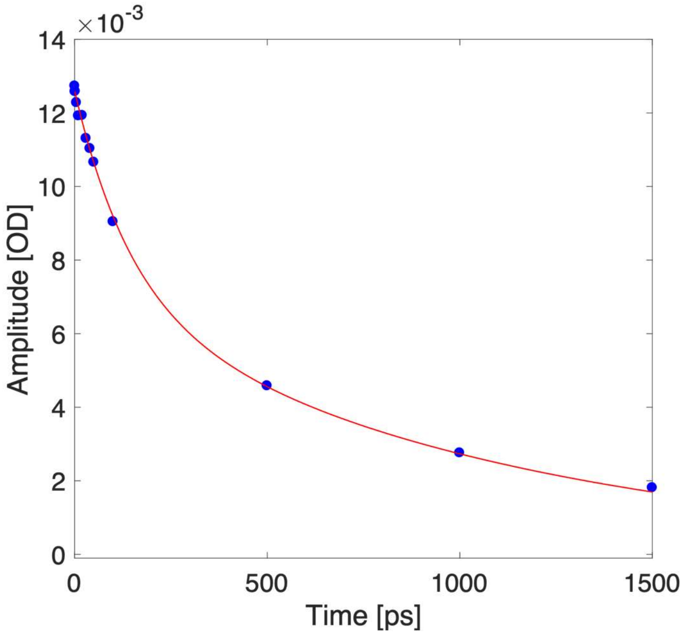

Figure S38.2. Kinetic trace taken at 1480 cm^{-1} (blue dots depict experimental data for selected delays, red curve represents a biexponential fit).

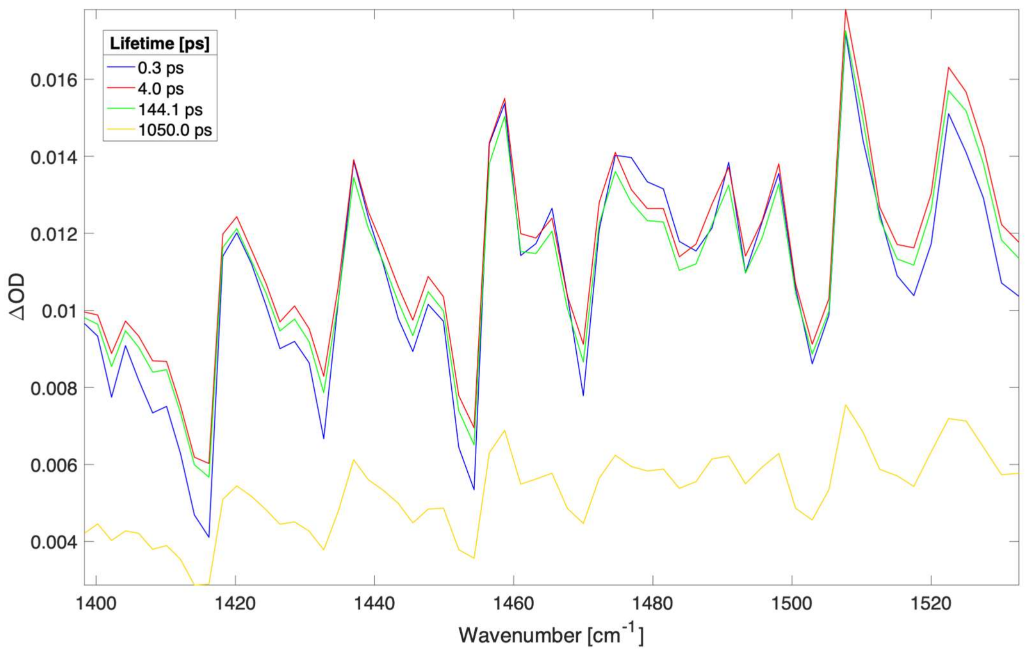

Figure S38.3. Time independent spectra of sequential three compartments global fit to spectra shown in Figure S38.1. The time constants (and contribution to data) were: 0.3 ps (27.6%), 4.0 ps (30.0%), 144.1 ps (28.4%), and 1050.0 ps (14.0%).

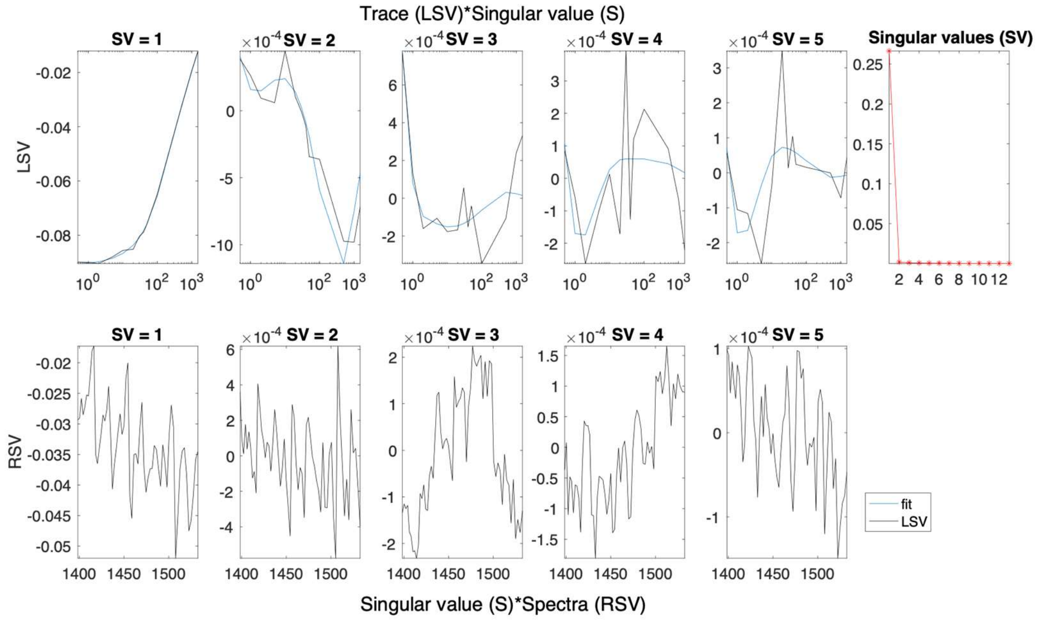

Figure S38.4. Left (LSV) and right (RSV) singular vectors with dominant singular values for the SVD analysis of spectra presented in Figure S38.1.

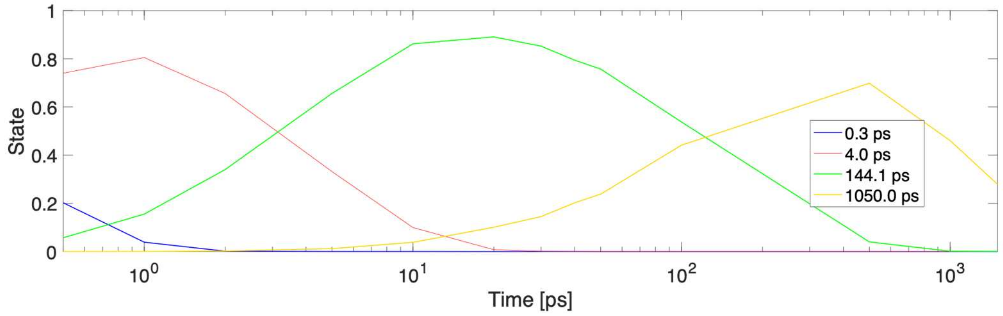

Figure S38.5. Concentration profiles for each time constant fitted to the data in Figure S38.3.

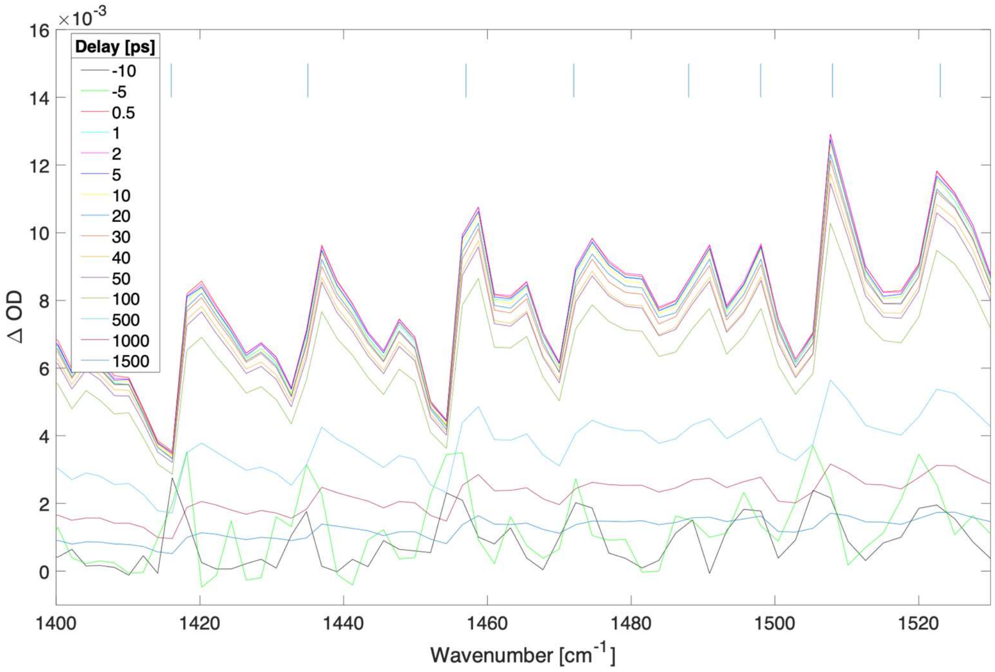

Figure S39.1. Difference transient absorption spectra at selected delays (ps) for a 500 nm thick film of $MAPbBr_3$ with pump irradiance of 9.05e8 W/cm^2 (fluence of 226.4 uJ/cm^2) at 539 nm.

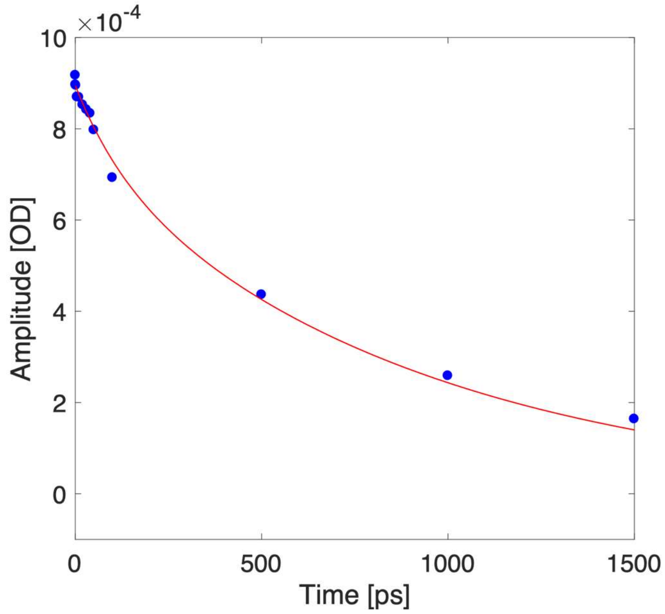

Figure S39.2. Kinetic trace taken at 1480 cm^{-1} (blue dots depict experimental data for selected delays, red curve represents a biexponential fit).

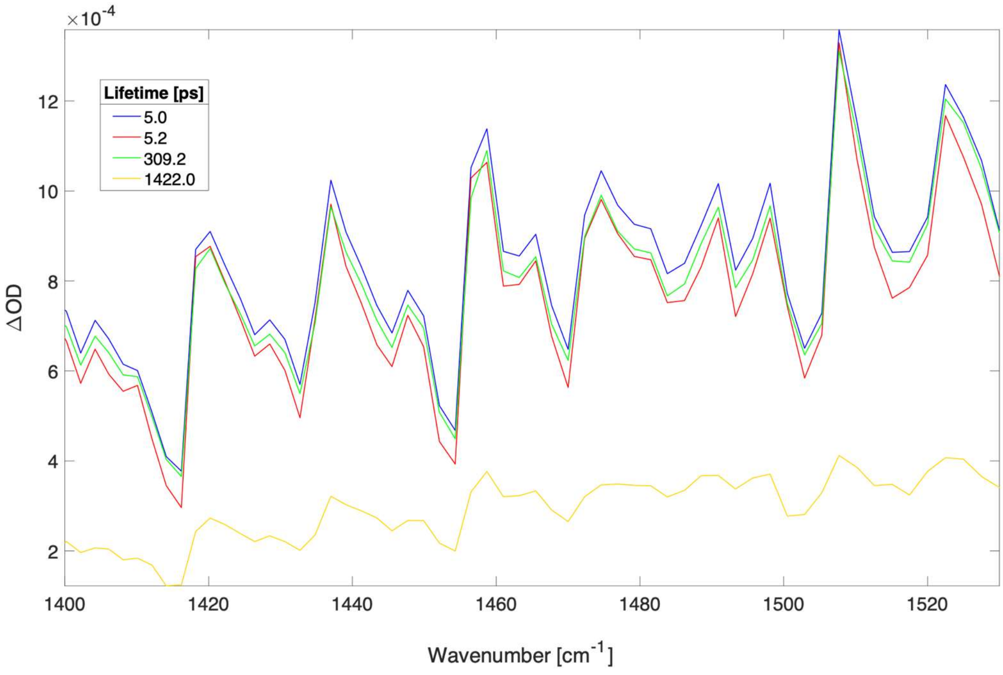

Figure S39.3. Time independent spectra of sequential three compartments global fit to spectra shown in Figure S39.1. The time constants (and contribution to data) were: 5.0 ps (29.8%), 5.2 ps (28.6%), 309.2 ps (28.7%), and 1422.0 ps (12.9%).

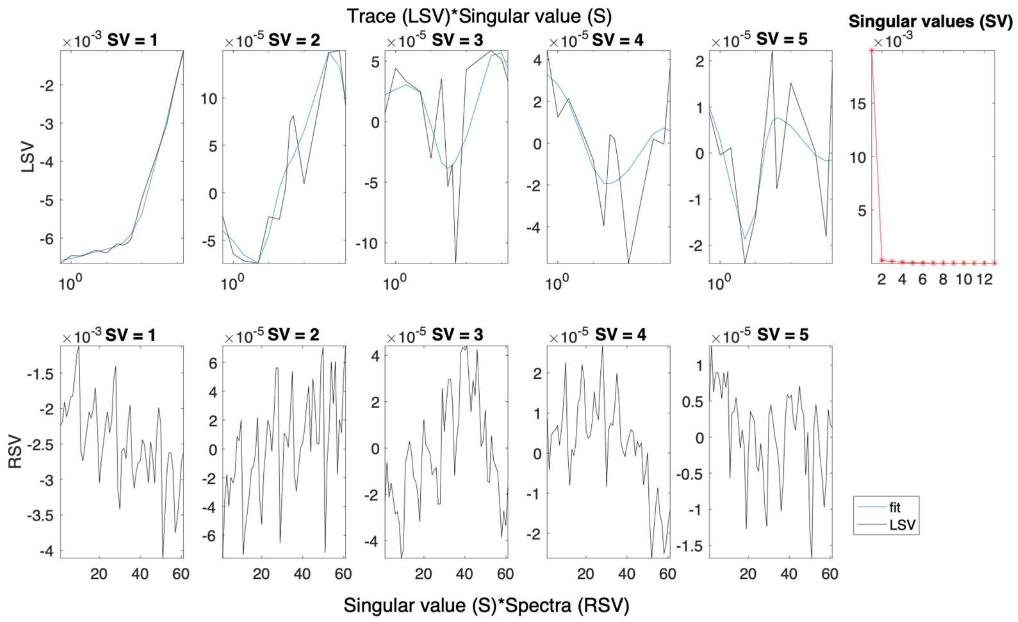

Figure S39.4. Left (LSV) and right (RSV) singular vectors with dominant singular values for the SVD analysis of spectra presented in Figure S39.1.

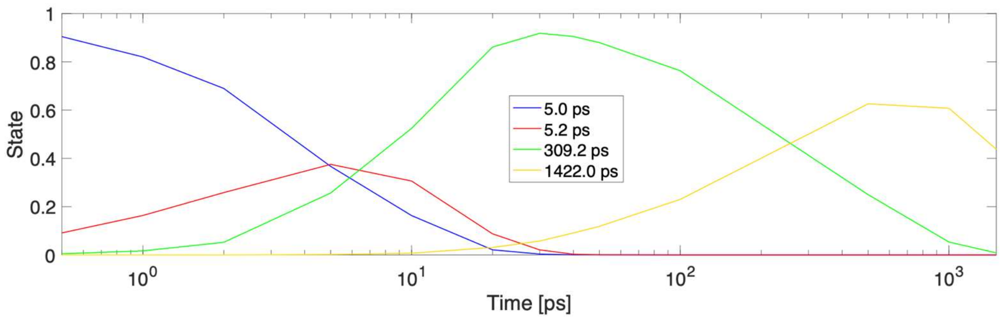

Figure S39.5. Concentration profiles for each time constant fitted to the data in Figure S39.3.

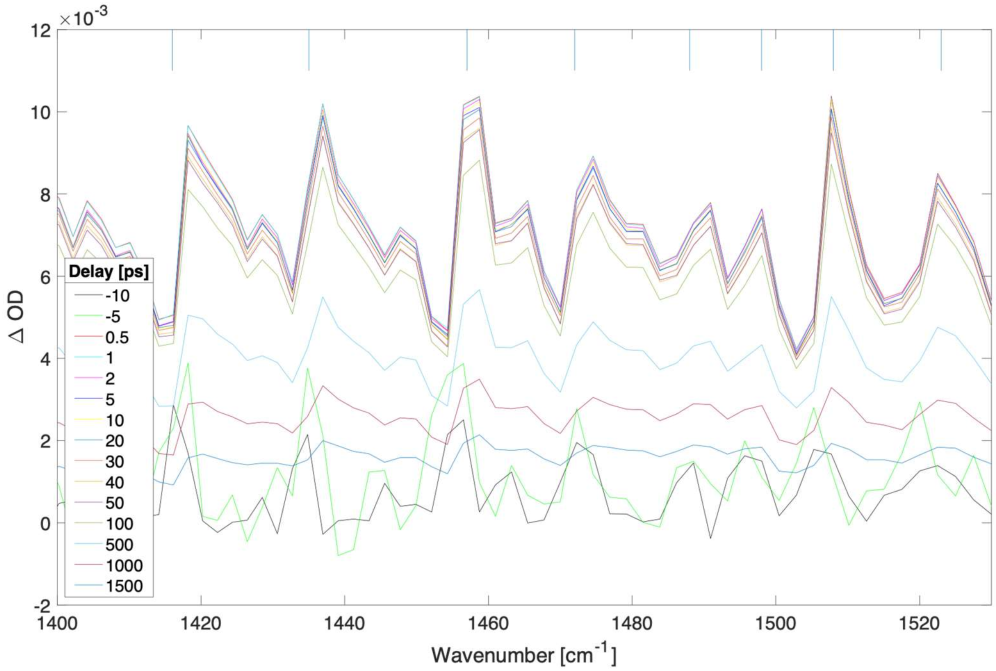

Figure S40.1. Difference transient absorption spectra at selected delays (ps) for a 500 nm thick film of $MAPbBr_3$with pump irradiance of 2.94e8 W/cm^2 (fluence of 73.6 uJ/cm^2) at 539 nm.

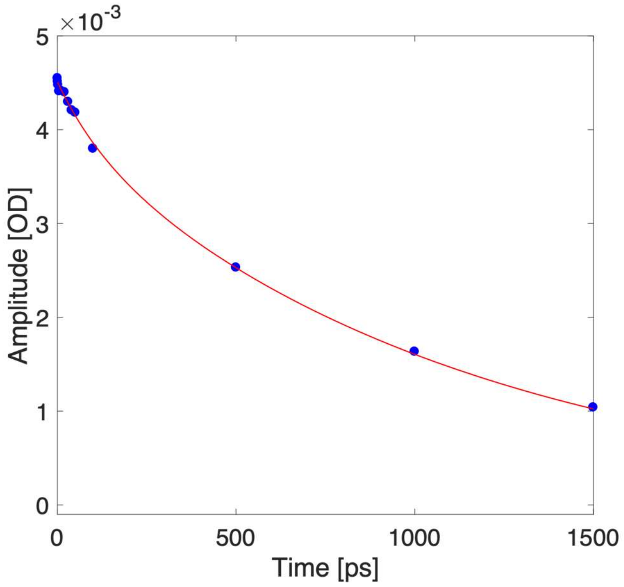

Figure S40.2. Kinetic trace taken at 1480 cm^{-1} (blue dots depict experimental data for selected delays, red curve represents a biexponential fit).

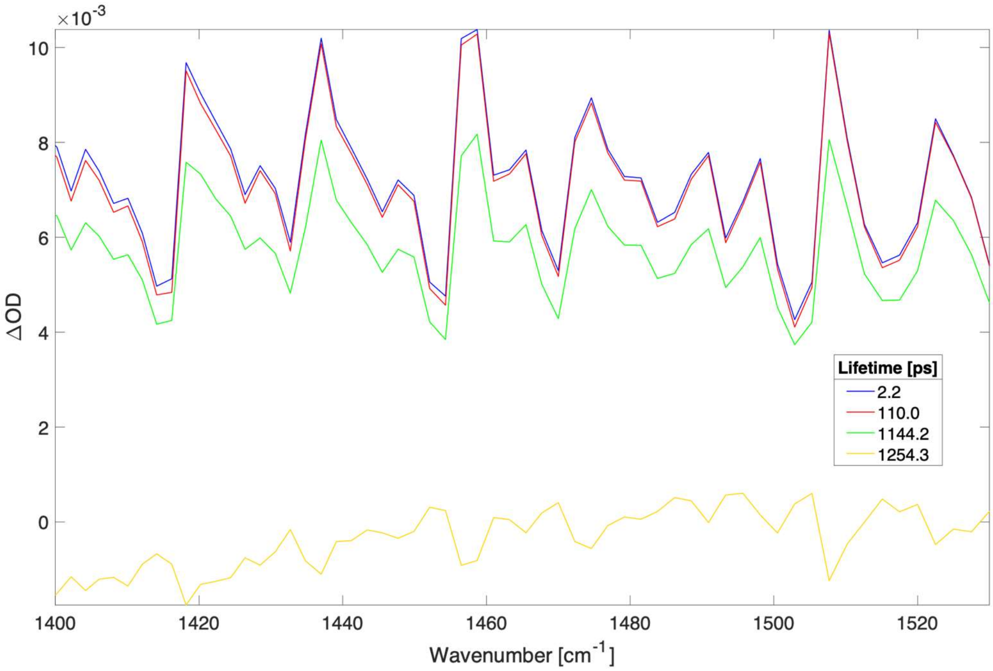

Figure S40.3. Time independent spectra of sequential three compartments global fit to spectra shown in Figure S40.1. The time constants (and contribution to data) were: 2.2 ps (32.5%), 110.0 ps (32.0%), 1144.2 ps (26.8%), and 1254.3 ps (8.7%).

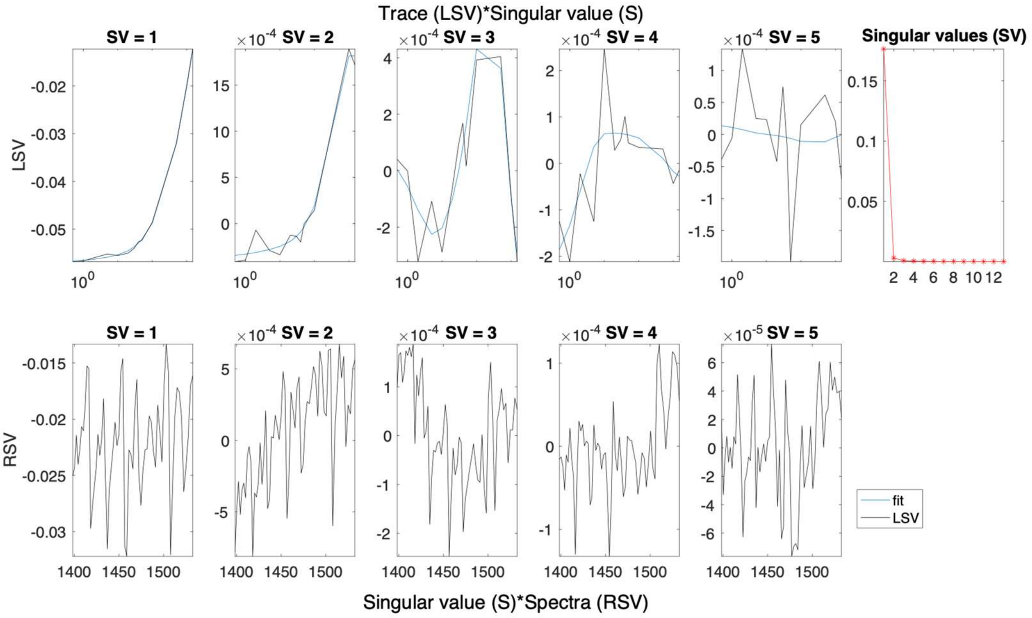

Figure S40.4. Left (LSV) and right (RSV) singular vectors with dominant singular values for the SVD analysis of spectra presented in Figure S40.1.

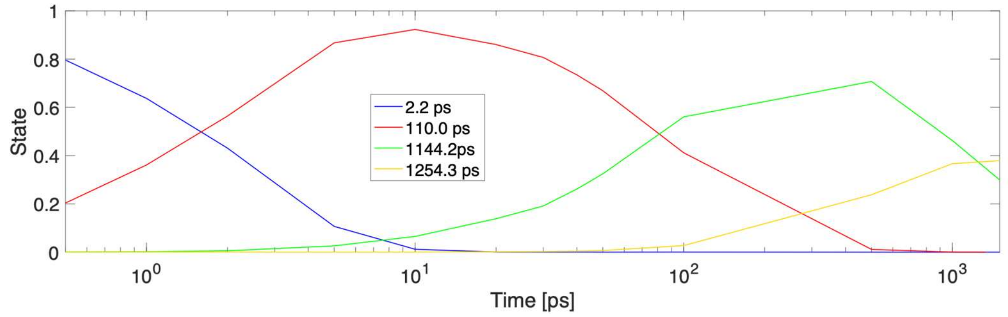

Figure S40.5. Concentration profiles for each time constant fitted to the data in Figure S40.3.

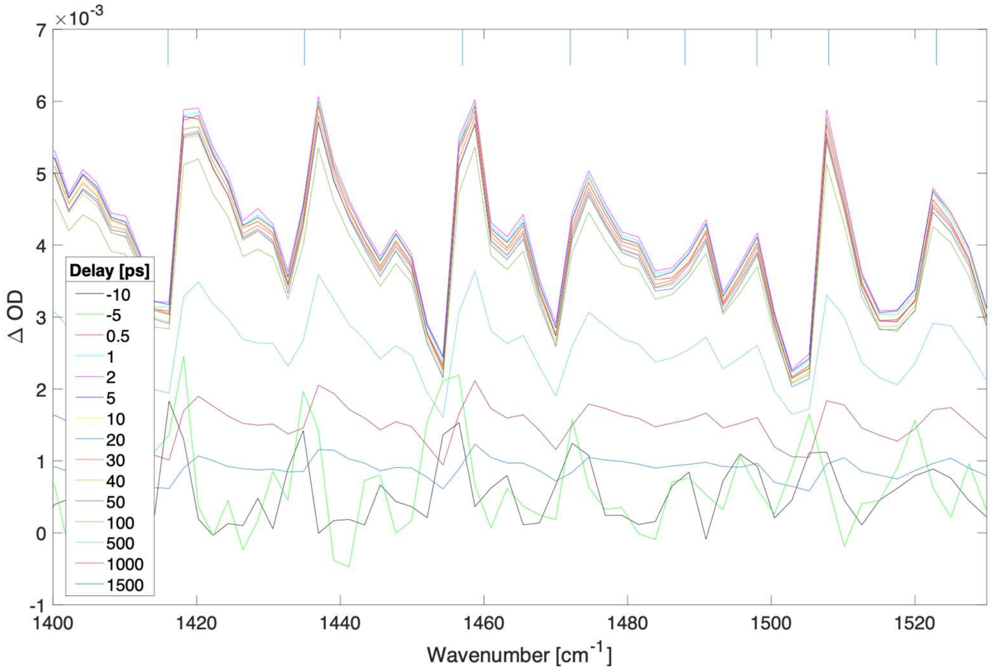

Figure S41.1. Difference transient absorption spectra at selected delays (ps) for a 500 nm thick film of $MAPbBr_3$ with pump irradiance of 1.47e8 W/cm^2 (fluence of 36.8 uJ/cm^2) at 539 nm.

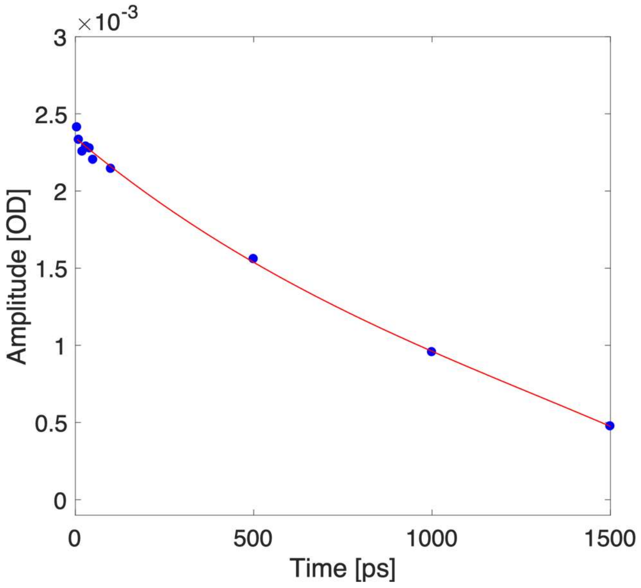

Figure S41.2. Kinetic trace taken at 1480 cm^{-1} (blue dots depict experimental data for selected delays, red curve represents a biexponential fit).

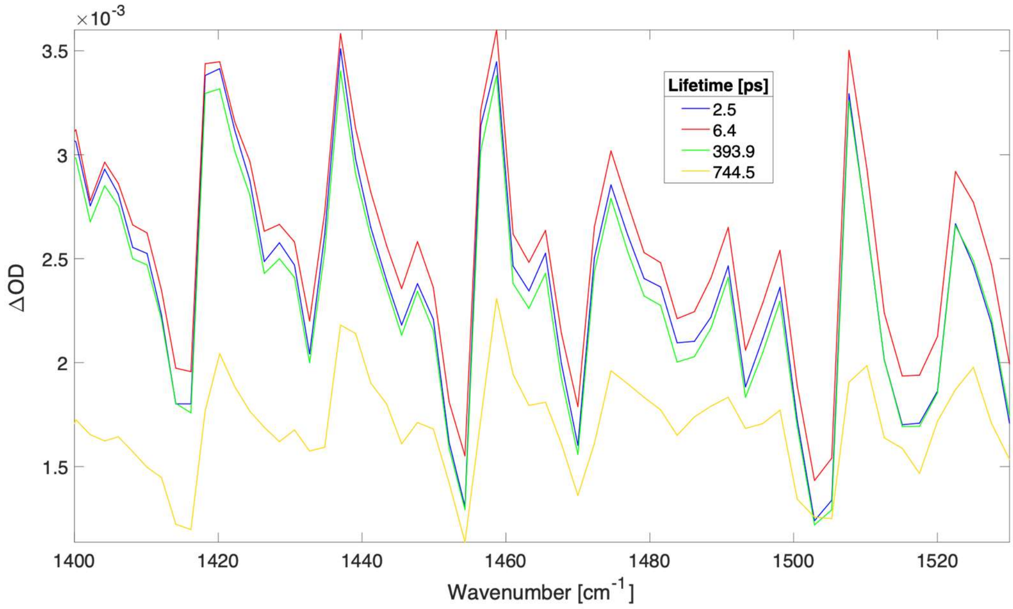

Figure S41.3. Time independent spectra of sequential three compartments global fit to spectra shown in Figure S41.1. The time constants (and contribution to data) were: 2.5 ps (26.0%), 6.4 ps (27.7%), 393.9 ps (25.2%), and 744.5 ps (21.1%).

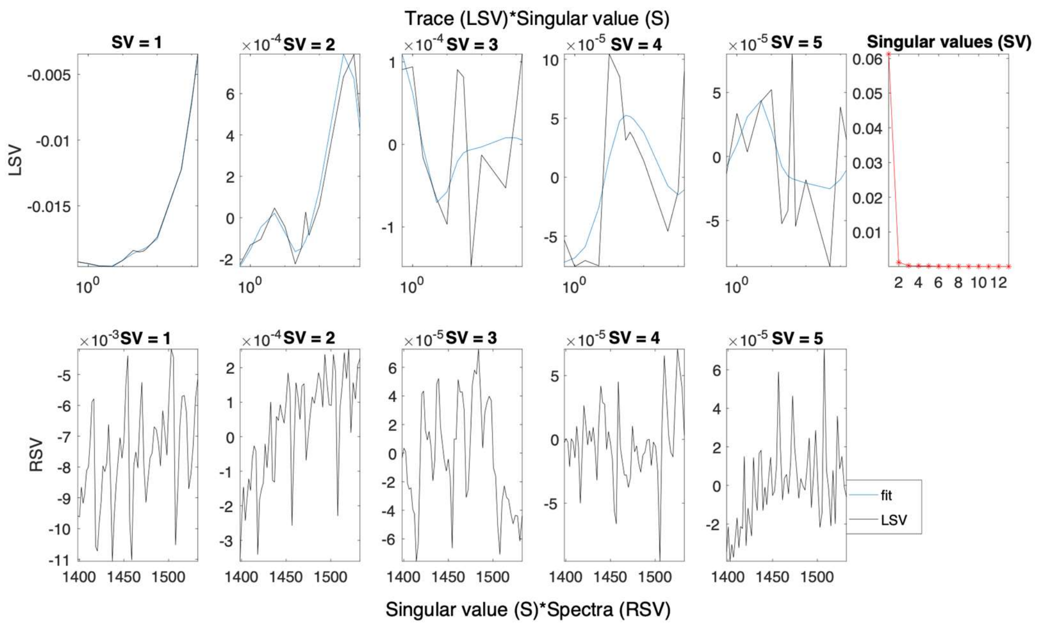

Figure S41.4. Left (LSV) and right (RSV) singular vectors with dominant singular values for the SVD analysis of spectra presented in Figure S41.1.

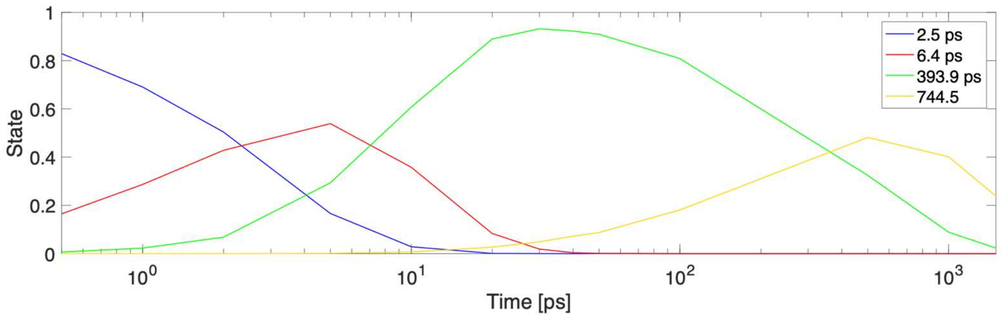

Figure S41.5. Concentration profiles for each time constant fitted to the data in Figure S41.3.

Time-resolved charge recombination analysis upon optical excitation of 400 nm thick film of $(FAPbI_3)_{0.97}(MAPbBr_3)_{0.03}$

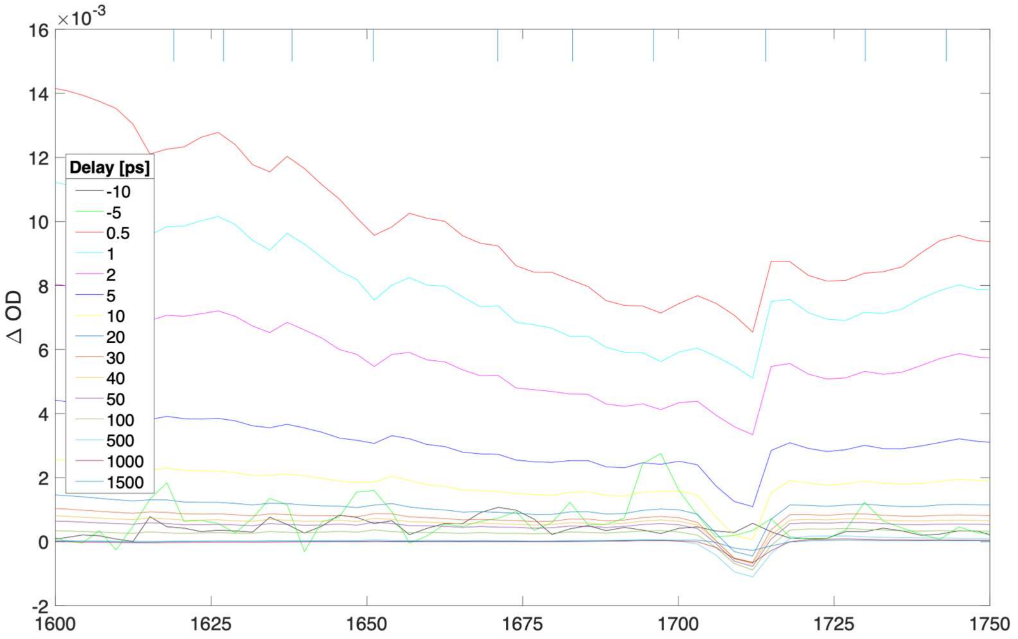

Figure S42.1. Difference transient absorption spectra at selected delays (ps) for a 400 nm thick film of $(FAPbI_3)_{0.97}(MAPbBr_3)_{0.03}$ with pump irradiance of 4.48e10 W/cm^2 (fluence of 11,204.5 uJ/cm^2) at 539 nm.

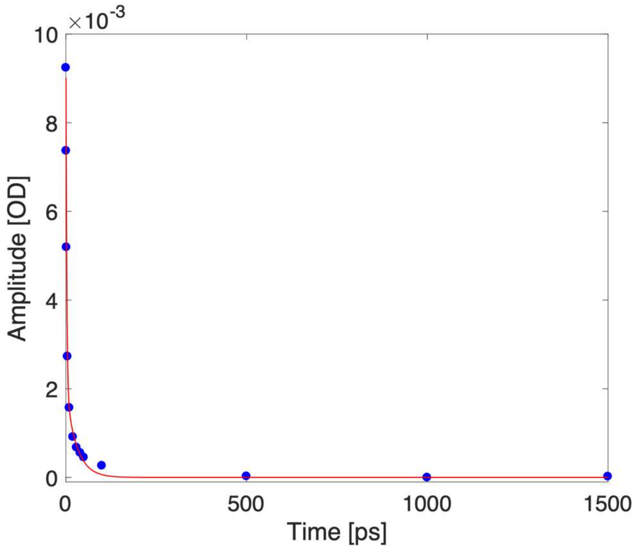

Figure S42.2. Kinetic trace taken at 1670 cm^{-1} (blue dots depict experimental data for selected delays, red curve represents a biexponential fit).

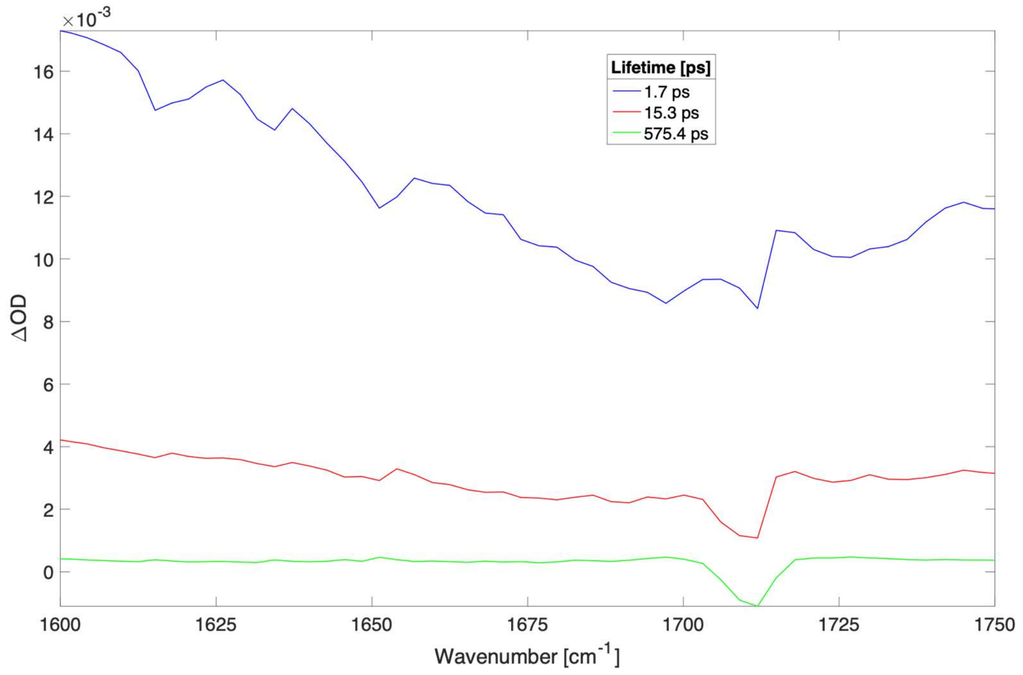

Figure S42.3. Time independent spectra of sequential three compartments global fit to spectra shown in Figure S42.1. The time constants (and contribution to data) were: 1.7 ps (76.5%), 15.3 ps (20.0%), and 575.4 ps (3.5%).

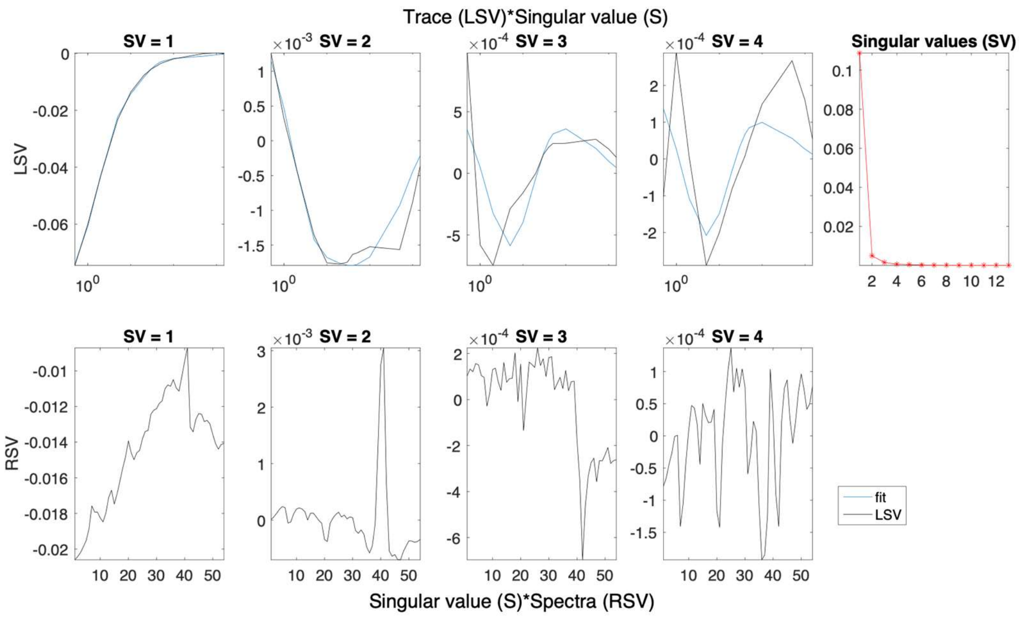

Figure S42.4. Left (LSV) and right (RSV) singular vectors with dominant singular values for the SVD analysis of spectra presented in Figure S42.1.

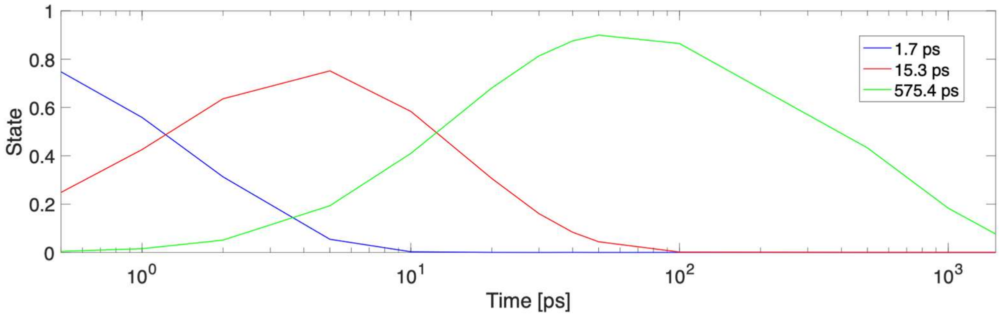

Figure S42.5. Concentration profiles for each time constant fitted to the data in Figure S42.3.

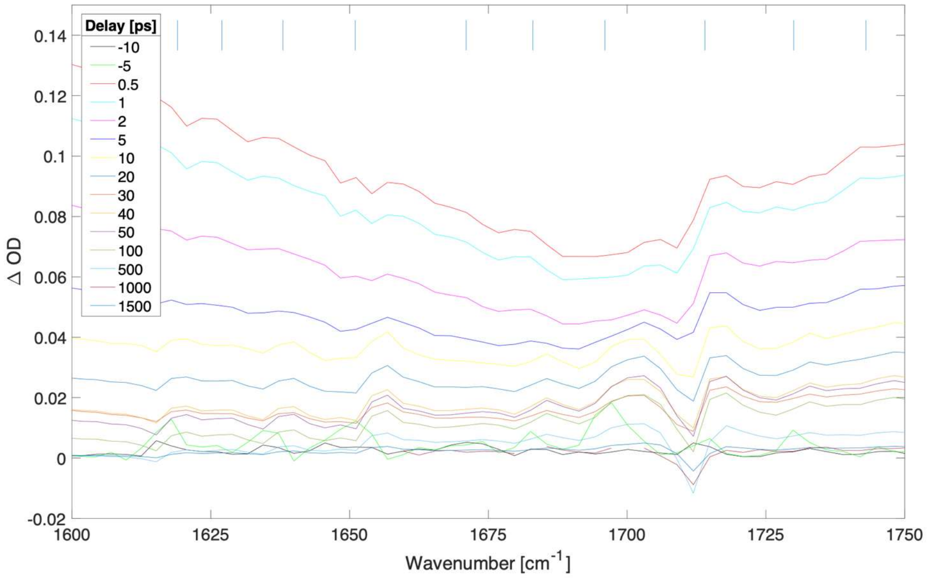

Figure S43.1. Difference transient absorption spectra at selected delays (ps) for a 400 nm thick film of $(FAPbI_3)_{0.97}(MAPbBr_3)_{0.03}$ with pump irradiance of 2.83e10 W/cm^2 (fluence of 7,073.6 uJ/cm^2) at 539 nm.

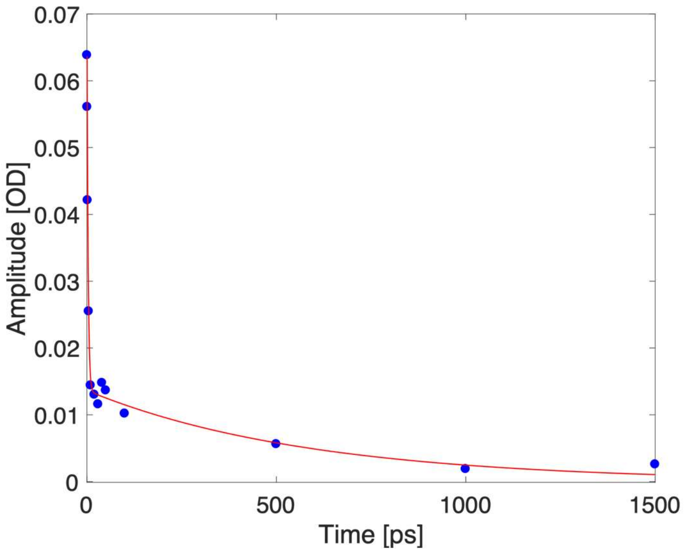

Figure S43.2. Kinetic trace taken at 1670 cm^{-1} (blue dots depict experimental data for selected delays, red curve represents a biexponential fit).

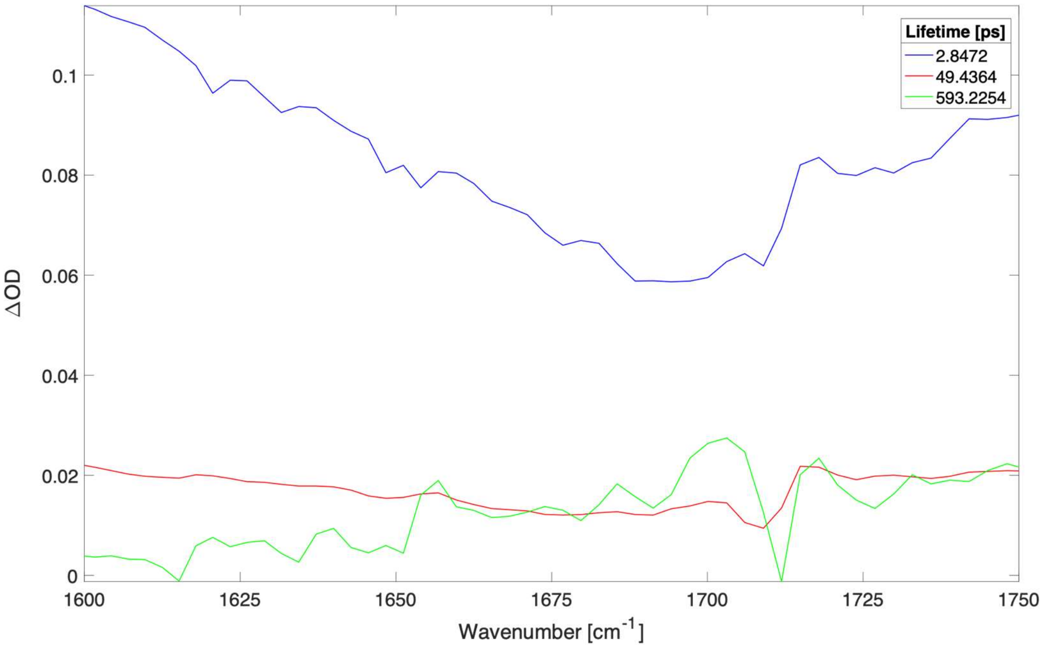

Figure S43.3. Time independent spectra of sequential three compartments global fit to spectra shown in Figure S43.1. The time constants (and contribution to data) were: 2.8 ps (68.5%), 49.4 ps (16.0%), and 593.2 ps (15.5%).

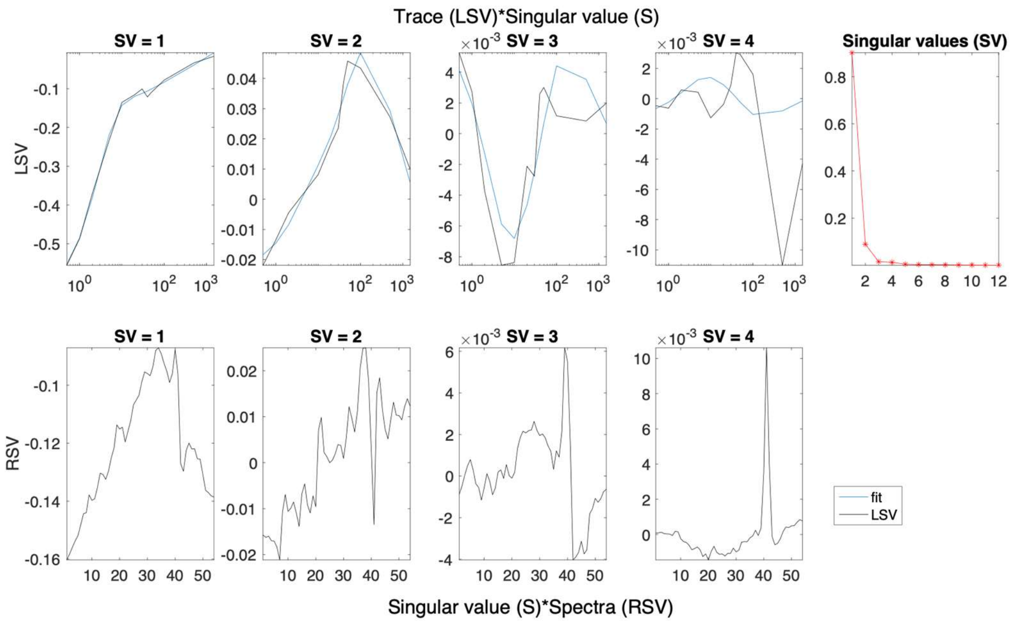

Figure S43.4. Left (LSV) and right (RSV) singular vectors with dominant singular values for the SVD analysis of spectra presented in Figure S43.1.

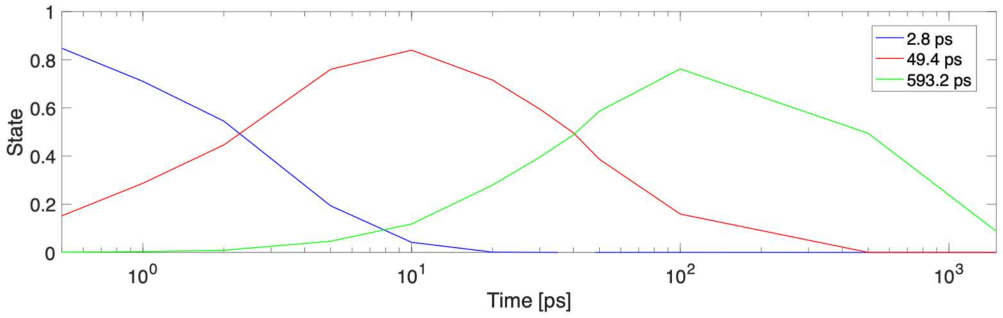

Figure S43.5. Concentration profiles for each time constant fitted to the data in Figure S43.3

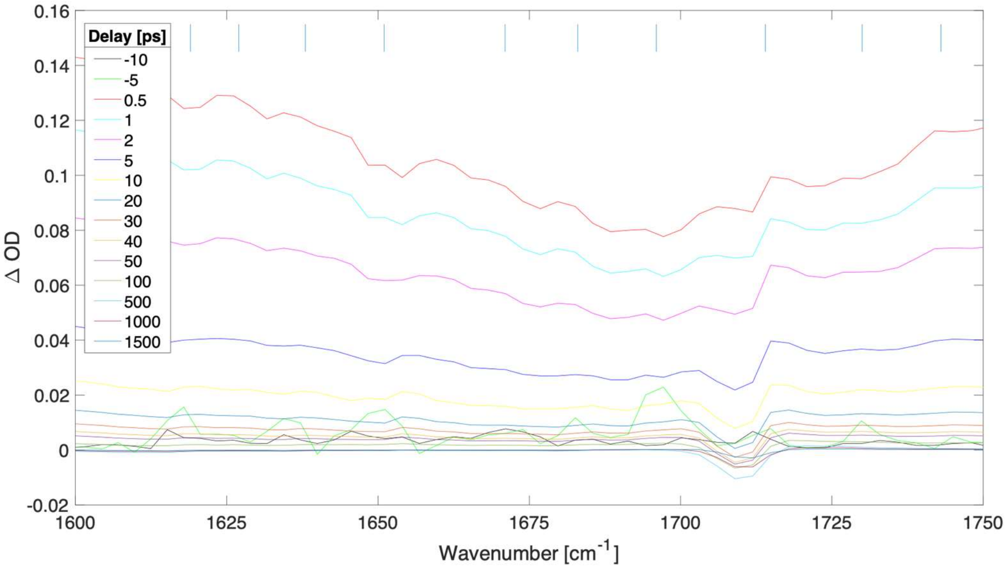

Figure S44.1. Difference transient absorption spectra at selected delays (ps) for a 400 nm thick film of $(FAPbI_3)_{0.97}(MAPbBr_3)_{0.03}$ with pump irradiance of 1.78.9e10 W/cm^2 (fluence of 4,470.5 uJ/cm^2) at 539 nm.

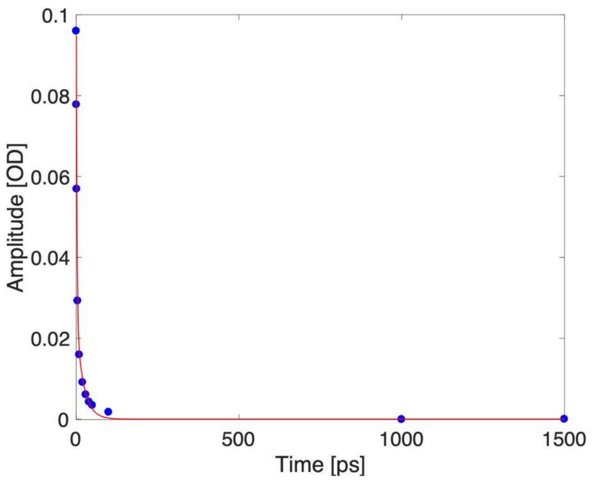

Figure S44.2. Kinetic trace taken at 1670 cm^{-1} (blue dots depict experimental data for selected delays, red curve represents a biexponential fit).

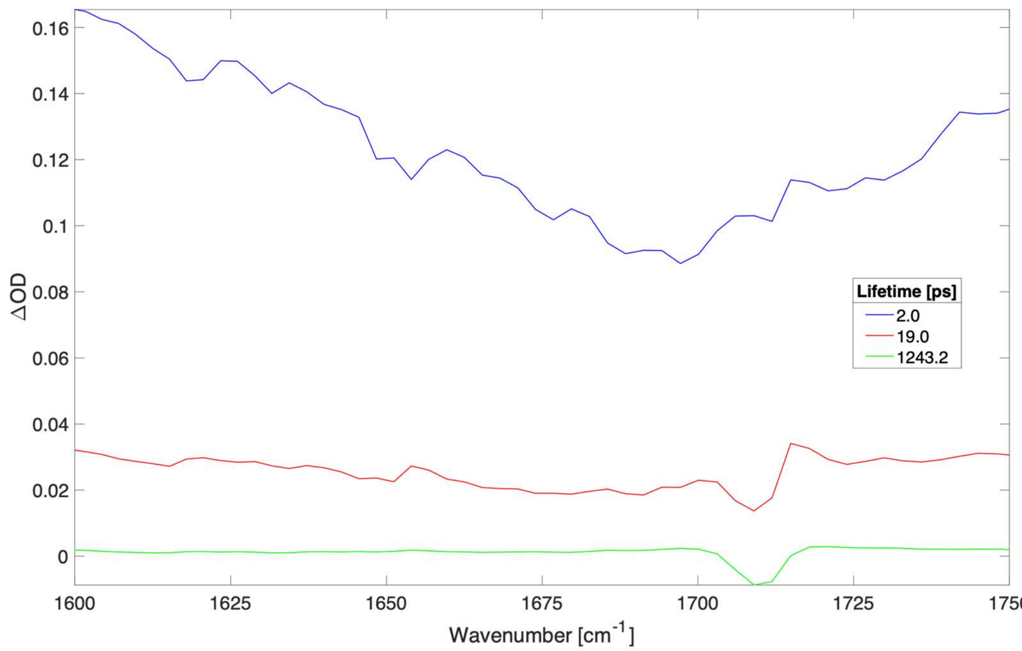

Figure S44.3. Time independent spectra of sequential three compartments global fit to spectra shown in Figure S44.1. The time constants (and contribution to data) were: 2.0 ps (79.1%), 19.0 ps (18.5%), and 1243.2 ps (2.4%).

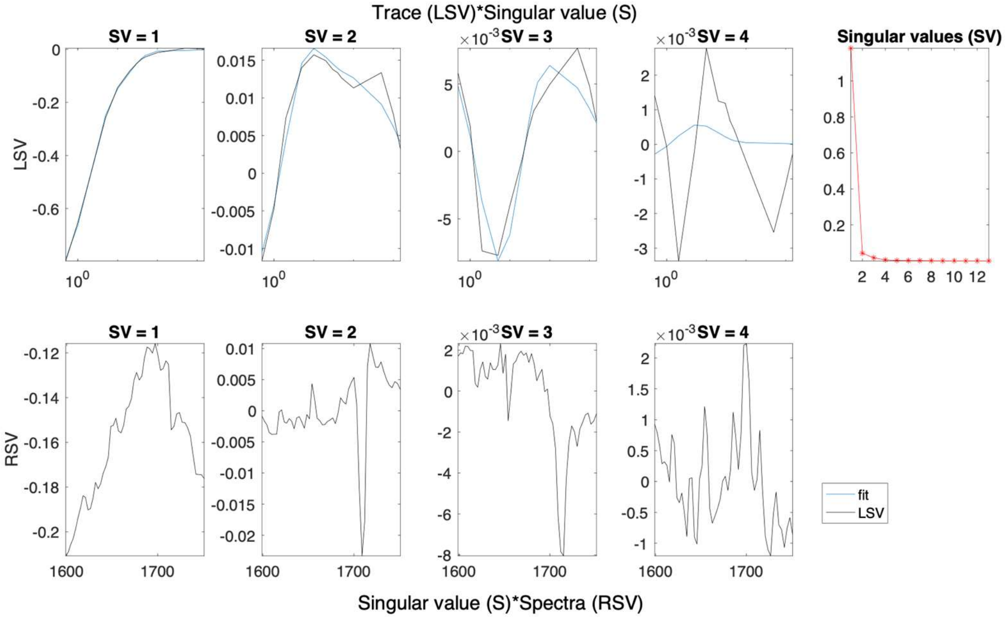

Figure S44.4. Left (LSV) and right (RSV) singular vectors with dominant singular values for the SVD analysis of spectra presented in Figure S44.1.

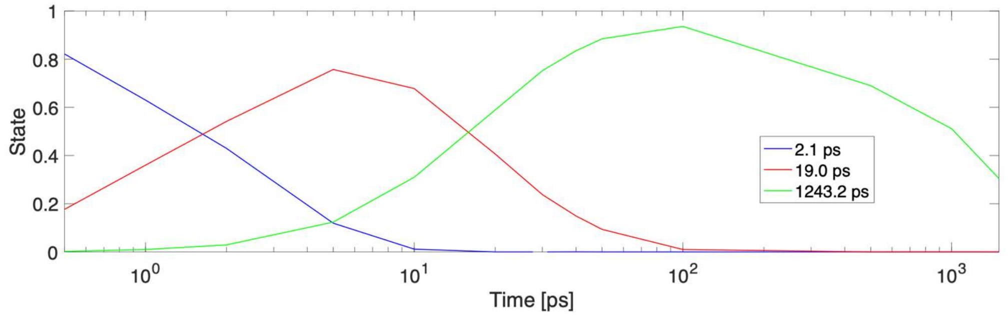

Figure S44.5. Concentration profiles for each time constant fitted to the data in Figure S44.3.

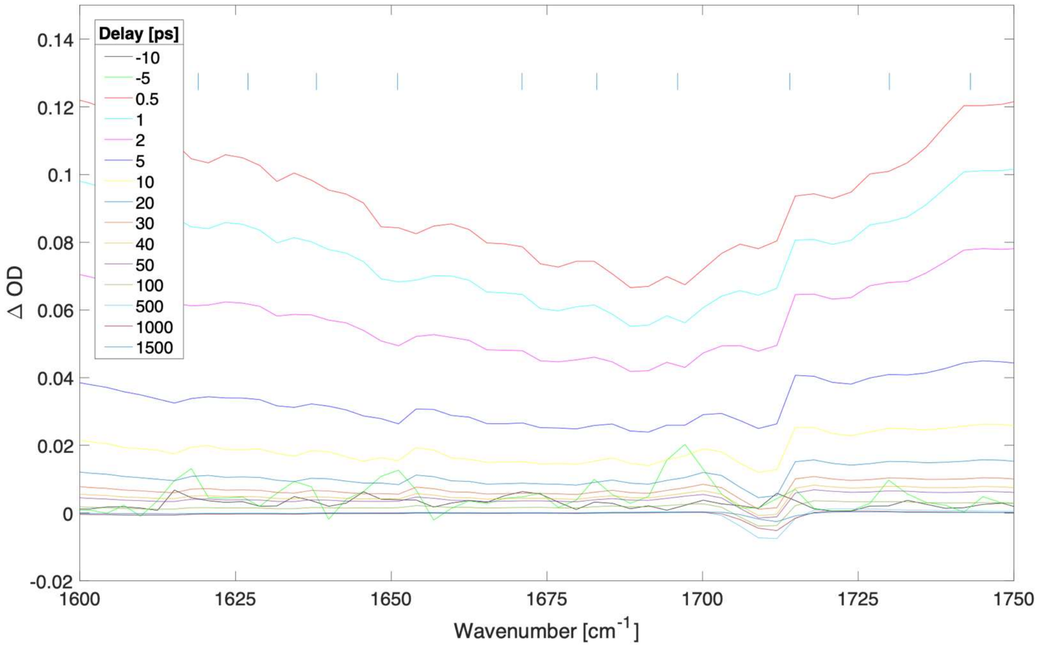

Figure S45.1. Difference transient absorption spectra at selected delays (ps) for a 400 nm thick film of $(FAPbI_3)_{0.97}(MAPbBr_3)_{0.03}$ with pump irradiance of 1.13e10 W/cm^2 (fluence of 2,829.4uJ/cm^2) at 539 nm.

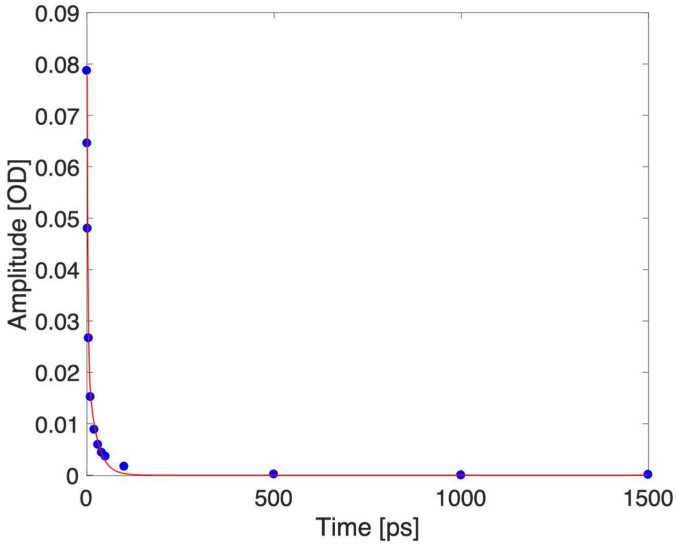

Figure S45.2. Kinetic trace taken at 1670 cm^{-1} (blue dots depict experimental data for selected delays, red curve represents a biexponential fit).

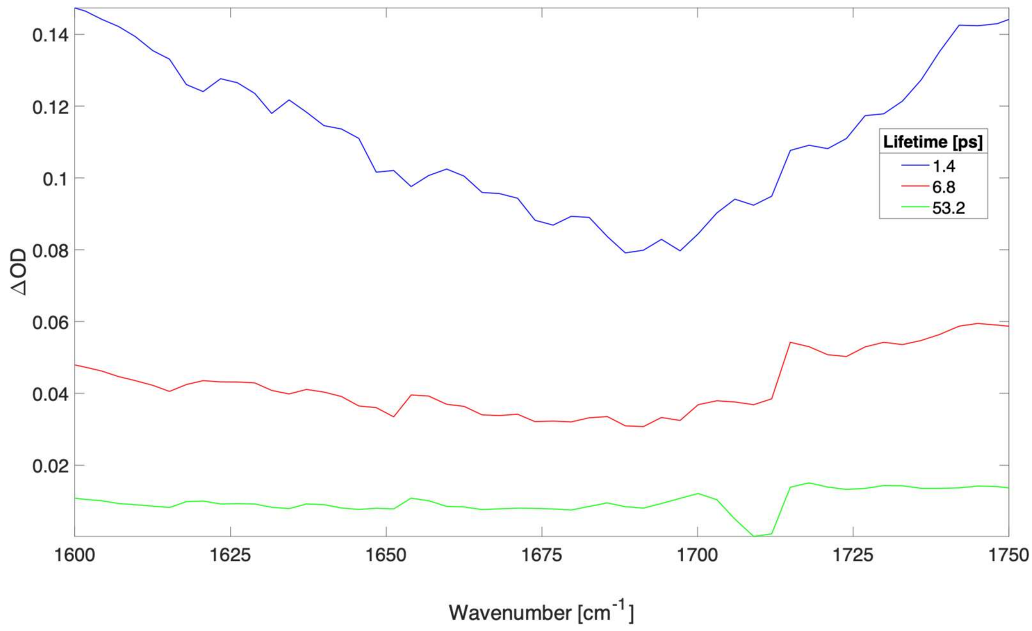

Figure S45.3. Time independent spectra of sequential three compartments global fit to spectra shown in Figure S45.1. The time constants (and contribution to data) were: 1.4 ps (65.7%), 6.8 ps (27.3%), and 53.2 (7.0%).

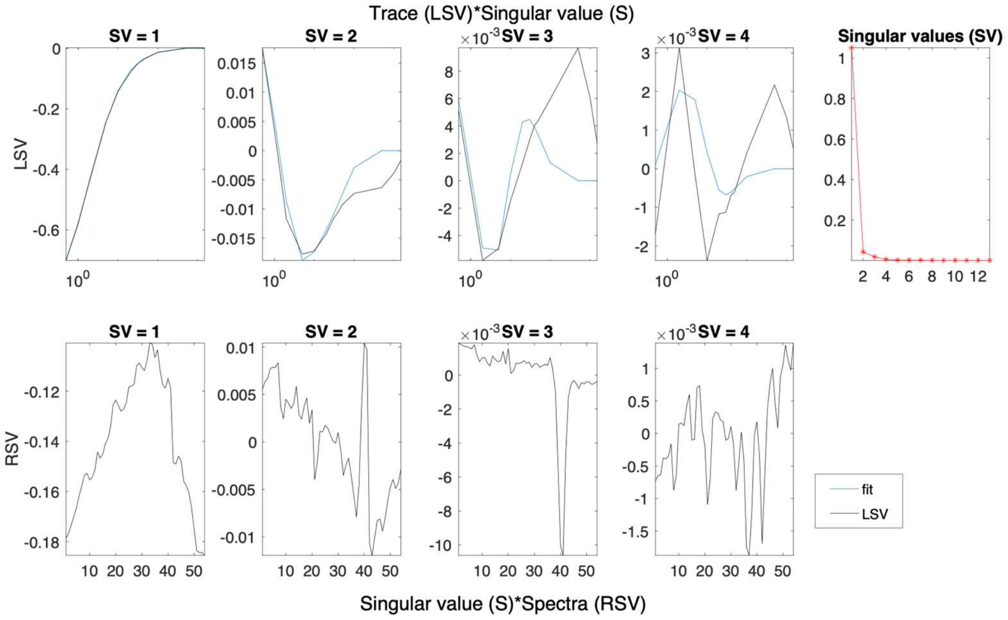

Figure S45.4. Left (LSV) and right (RSV) singular vectors with dominant singular values for the SVD analysis of spectra presented in Figure S45.1.

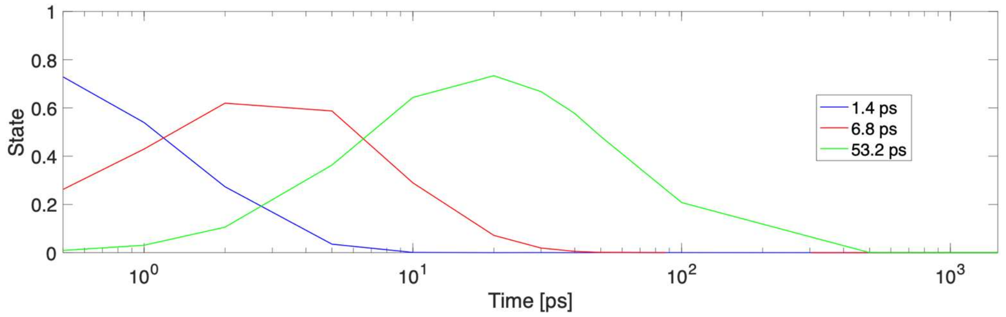

Figure S45.5. Concentration profiles for each time constant fitted to the data in Figure S45.3.

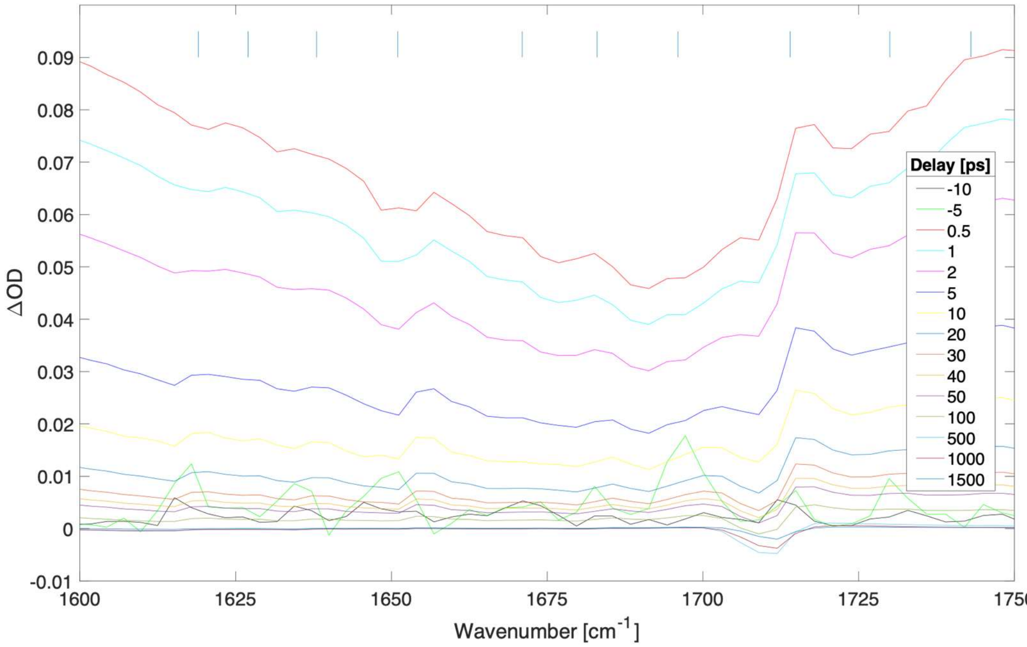

Figure S46.1. Difference transient absorption spectra at selected delays (ps) for a 400 nm thick film of $(FAPbI_3)_{0.97}(MAPbBr_3)_{0.03}$ with pump irradiance of 7.10e9 W/cm^2 (fluence of 1,774.0 uJ/cm^2) at 539 nm.

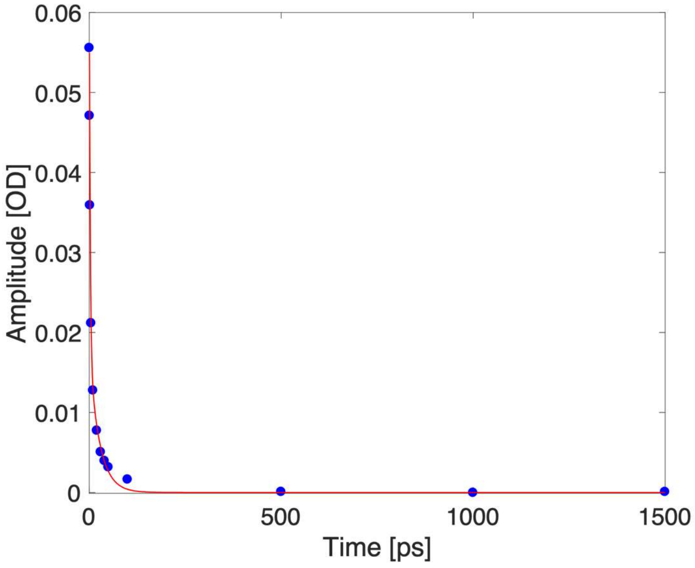

Figure S46.2. Kinetic trace taken at 1670 cm^{-1} (blue dots depict experimental data for selected delays, red curve represents a biexponential fit).

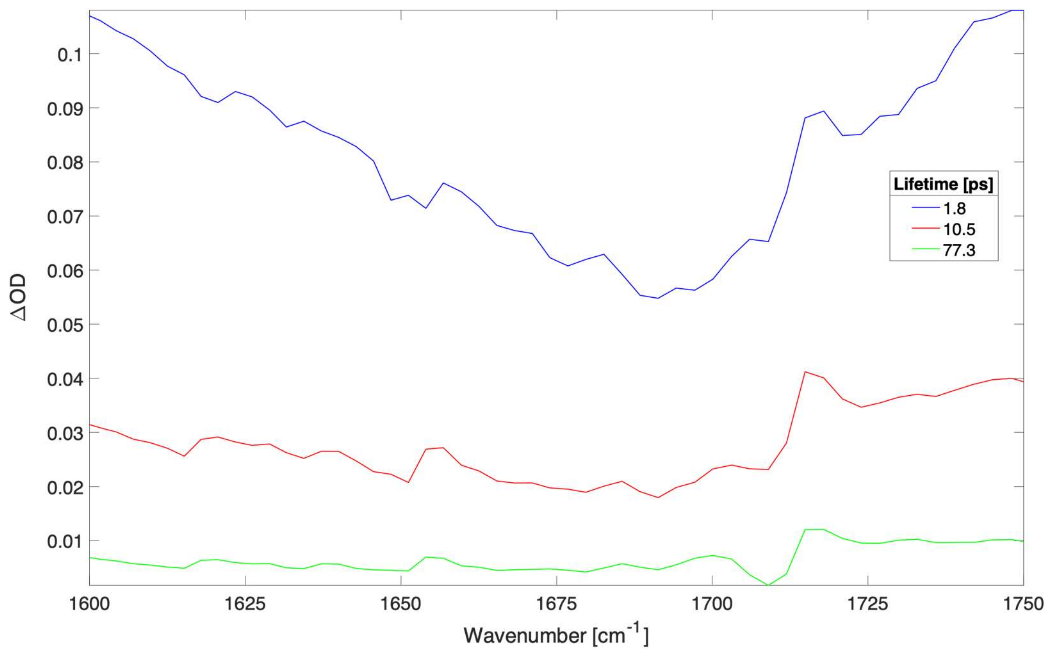

Figure S46.3. Time independent spectra of sequential three compartments global fit to spectra shown in Figure S46.1. The time constants (and contribution to data) were: 1.8 ps (68.0%), 10.5 ps (25.0%), and 77.3 ps (7.0%).

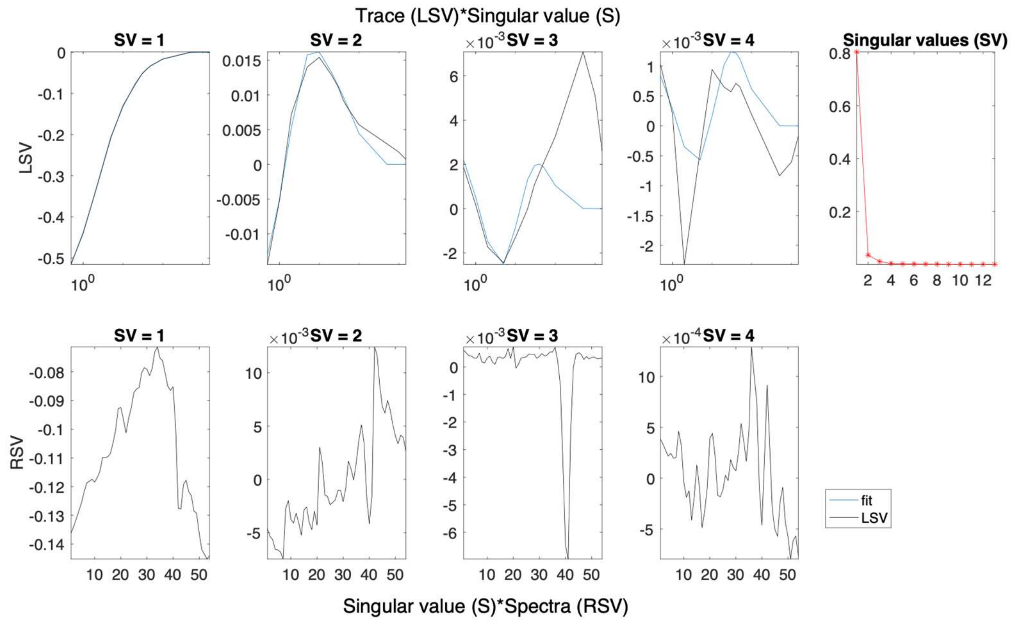

Figure S46.4. Left (LSV) and right (RSV) singular vectors with dominant singular values for the SVD analysis of spectra presented in Figure S46.1.

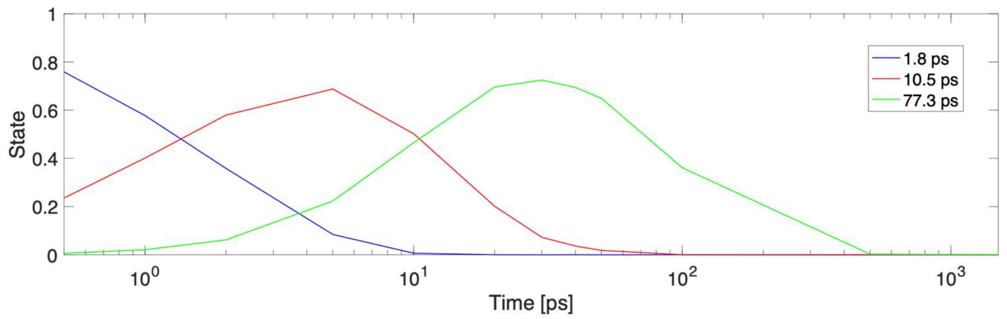

Figure S46.5. Concentration profiles for each time constant fitted to the data in Figure S46.3.

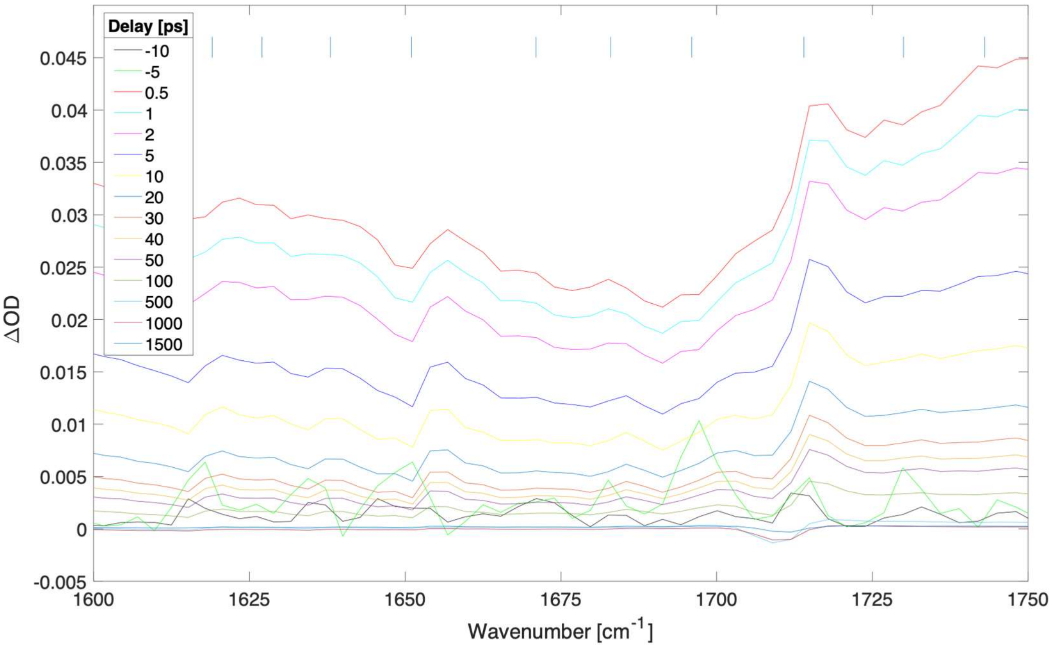

Figure S47.1. Difference transient absorption spectra at selected delays (ps) for a 400 nm thick film of $(FAPbI_3)_{0.97}(MAPbBr_3)_{0.03}$ with pump irradiance of 2.83e9 W/cm^2 (fluence of 707.4 uJ/cm^2) at 539 nm.

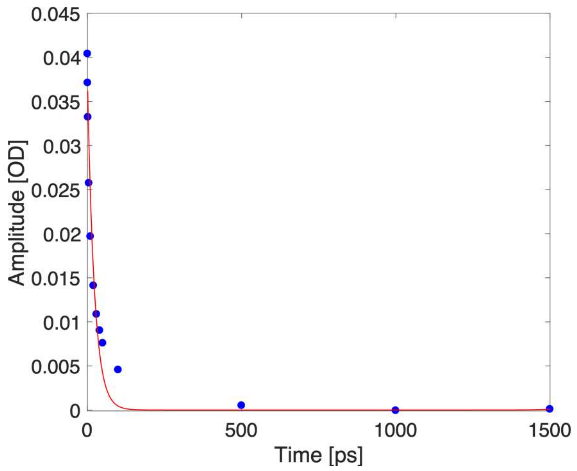

Figure S47.2. Kinetic trace taken at 1670 cm^{-1} (blue dots depict experimental data for selected delays, red curve represents a biexponential fit).

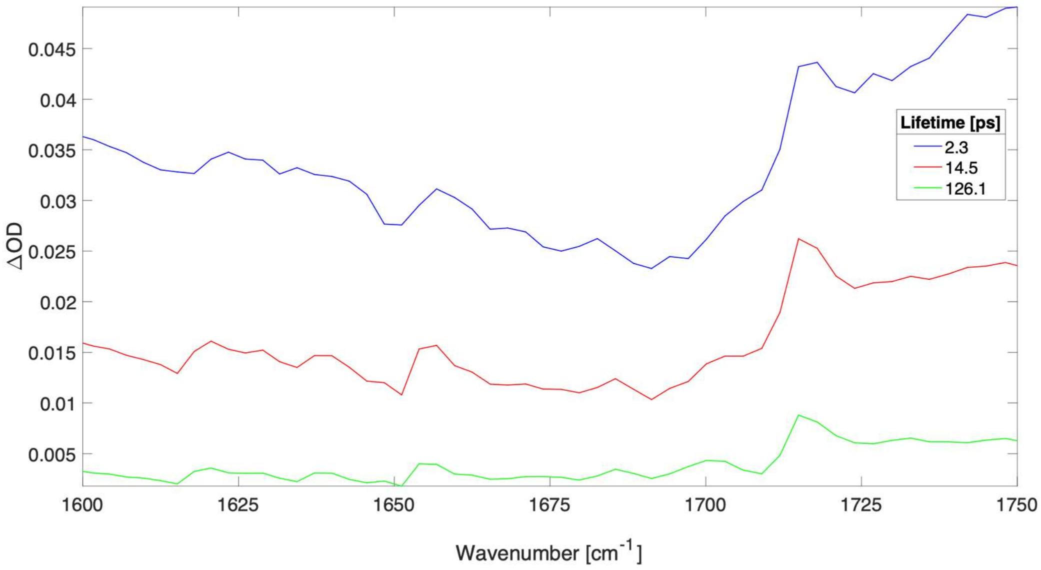

Figure S47.3. Time independent spectra of sequential three compartments global fit to spectra shown in Figure S47.1. The time constants (and contribution to data) were: 2.3 ps (64.7%), 14.5 ps (23.4%), and 126.1 ps (11.9%).

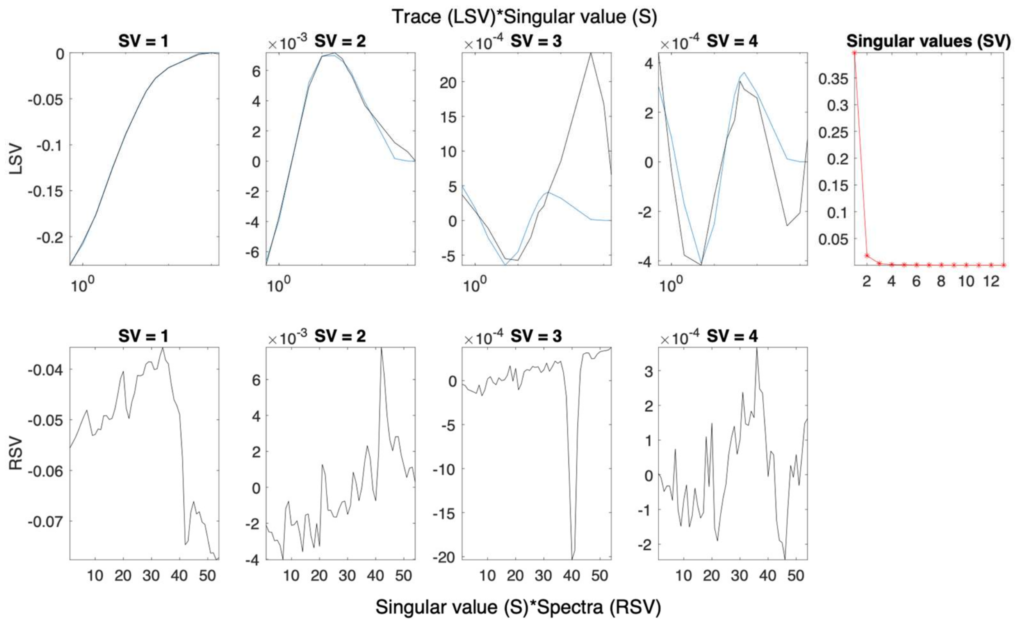

Figure S47.4. Left (LSV) and right (RSV) singular vectors with dominant singular values for the SVD analysis of spectra presented in Figure S47.1.

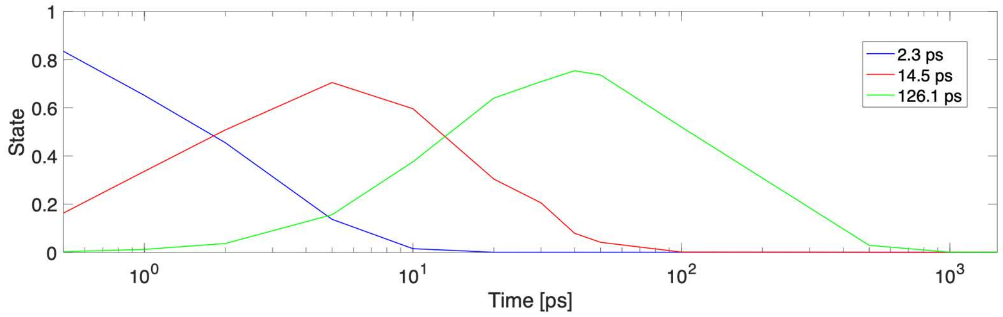

Figure S47.5. Concentration profiles for each time constant fitted to the data in Figure S47.3.

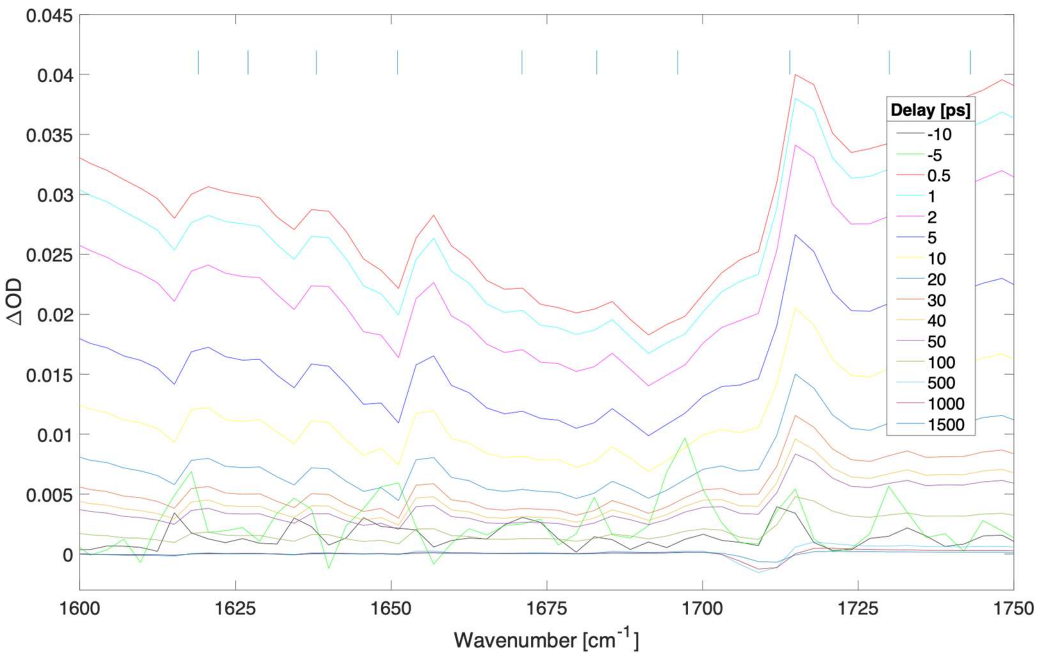

Figure S48.1. Difference transient absorption spectra at selected delays (ps) for a 400 nm thick film of $(FAPbI_3)_{0.97}(MAPbBr_3)_{0.03}$ with pump irradiance of 2.32 e9 W/cm^2 (fluence of 580.0 uJ/cm^2) at 539 nm.

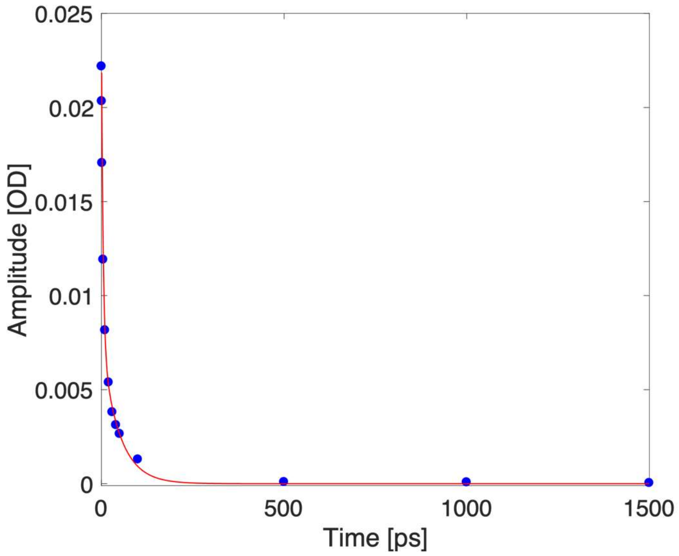

Figure S48.2. Kinetic trace taken at 1670 cm^{-1} (blue dots depict experimental data for selected delays, red curve represents a biexponential fit).

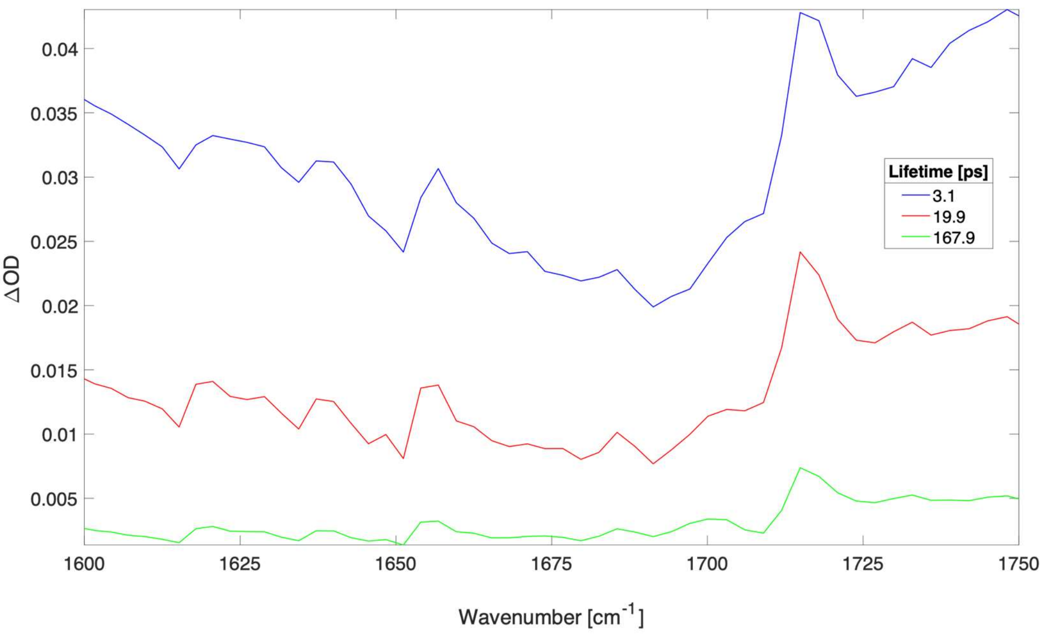

Figure S48.3. Time independent spectra of sequential three compartments global fit to spectra shown in Figure S48.1. The time constants (and contribution to data) were: 3.1 ps (62.0%), 19.9 ps (29.7%), and 167.9 ps (8.3%).

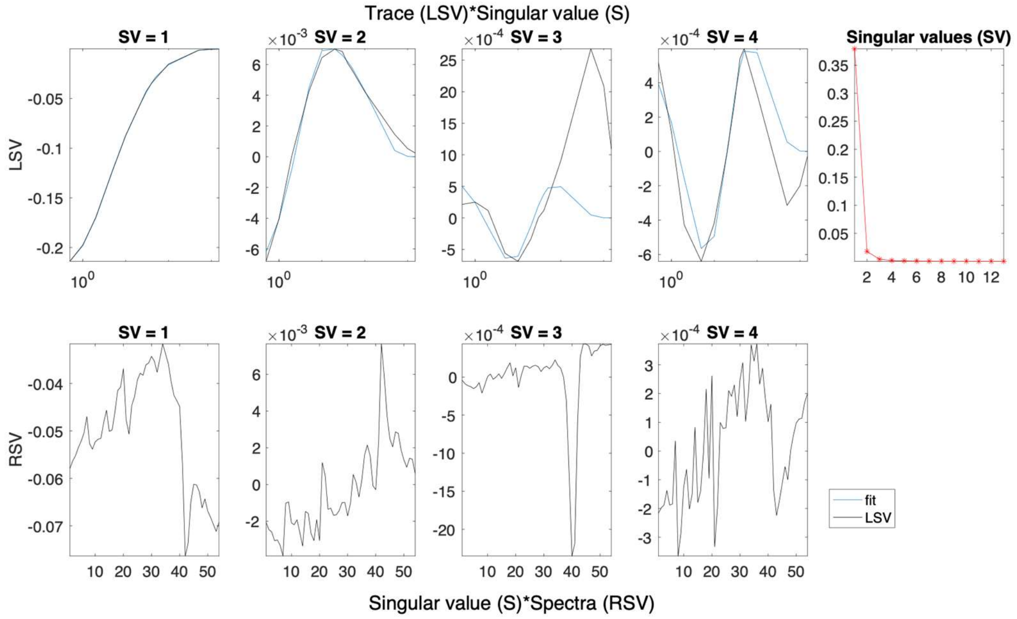

Figure S48.4. Left (LSV) and right (RSV) singular vectors with dominant singular values for the SVD analysis of spectra presented in Figure S48.1.

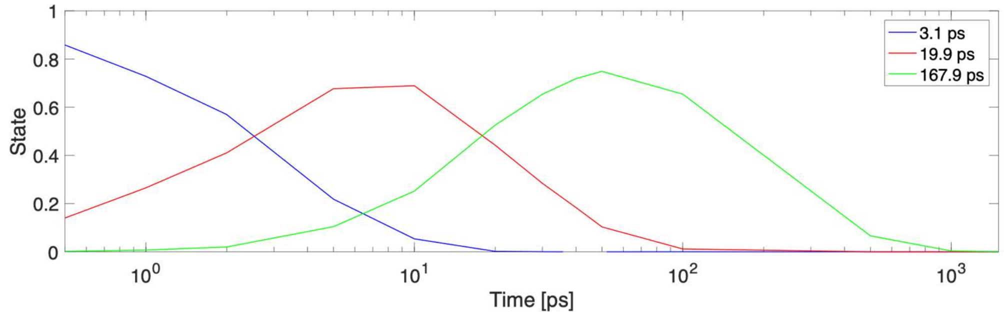

Figure S48.5. Concentration profiles for each time constant fitted to the data in Figure S48.3.

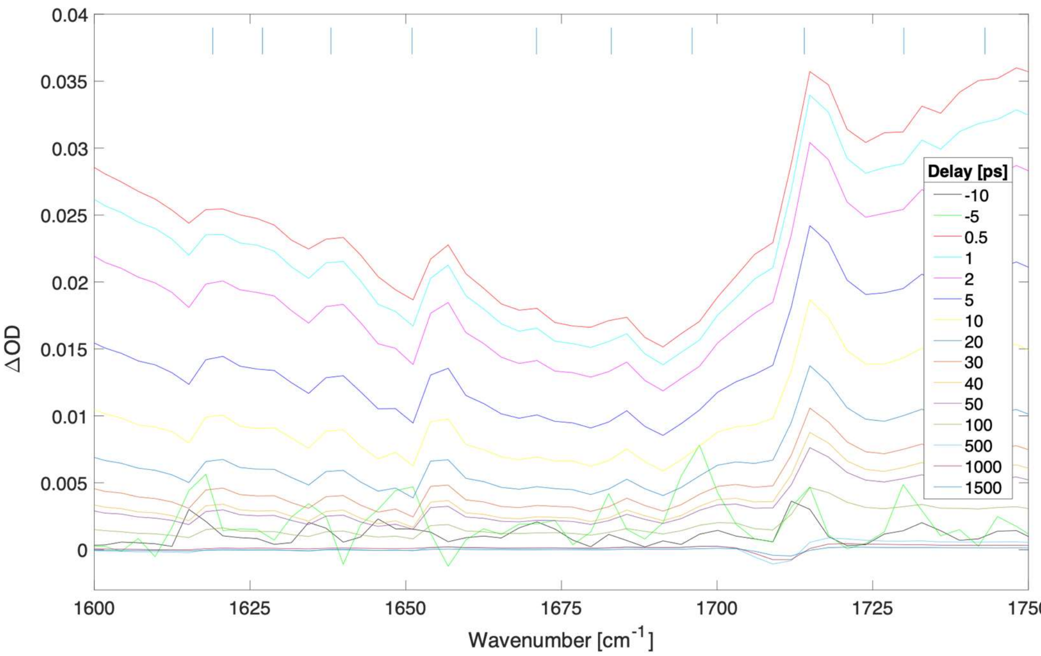

Figure S49.1. Difference transient absorption spectra at selected delays (ps) for a 400 nm thick film of $(FAPbI_3)_{0.97}(MAPbBr_3)_{0.03}$ with pump irradiance of 1.86e9 W/cm^2 (fluence of 464.0 uJ/cm^2) at 539 nm.

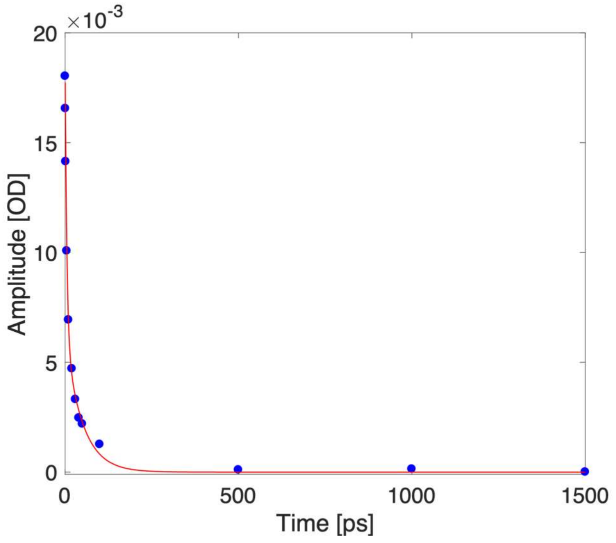

Figure S49.2. Kinetic trace taken at 1670 cm^{-1} (blue dots depict experimental data for selected delays, red curve represents a biexponential fit).

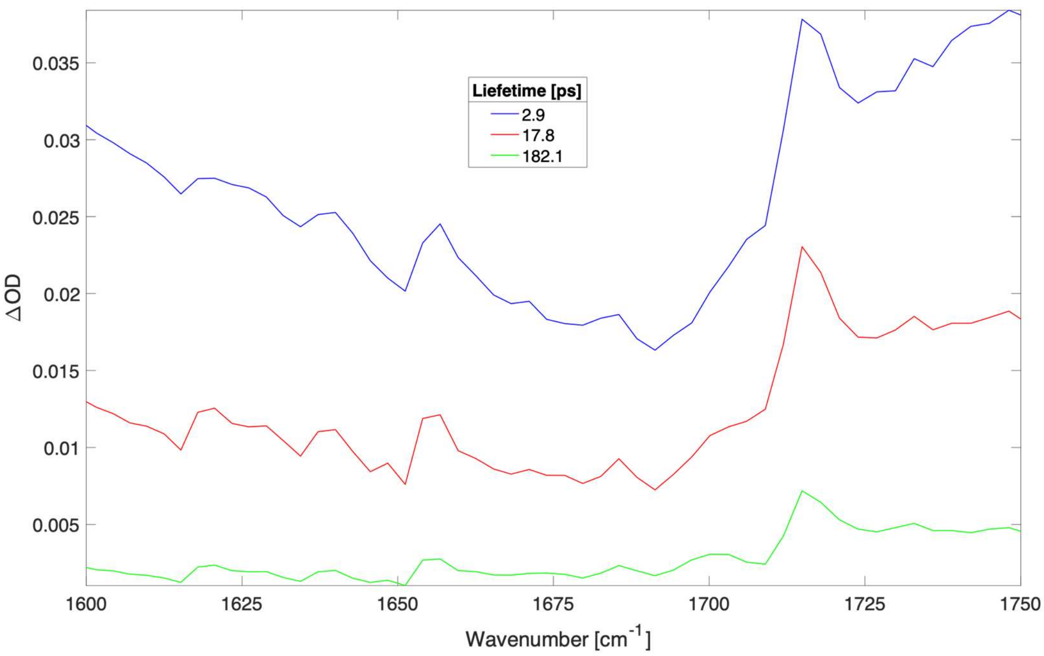

Figure S49.3. Time independent spectra of sequential three compartments global fit to spectra shown in Figure S45.1. The time constants (and contribution to data) were: 2.9 ps (60.4%), 17.8 ps (31.0%), and 182.1 ps (8.6%).

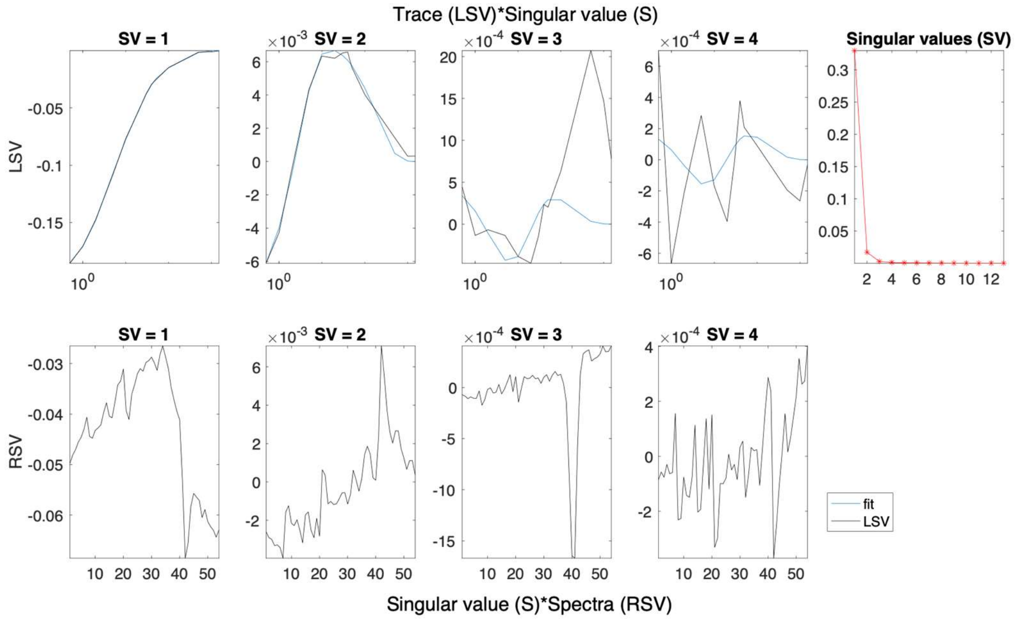

Figure S49.4. Left (LSV) and right (RSV) singular vectors with dominant singular values for the SVD analysis of spectra presented in Figure S49.1.

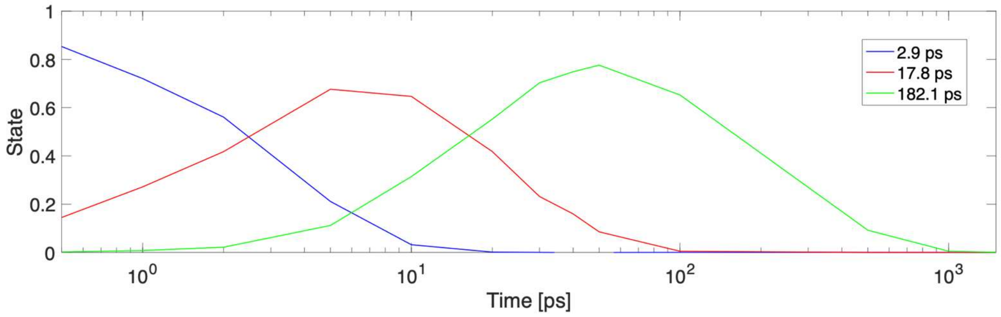

Figure S49.5. Concentration profiles for each time constant fitted to the data in Figure S45.3.

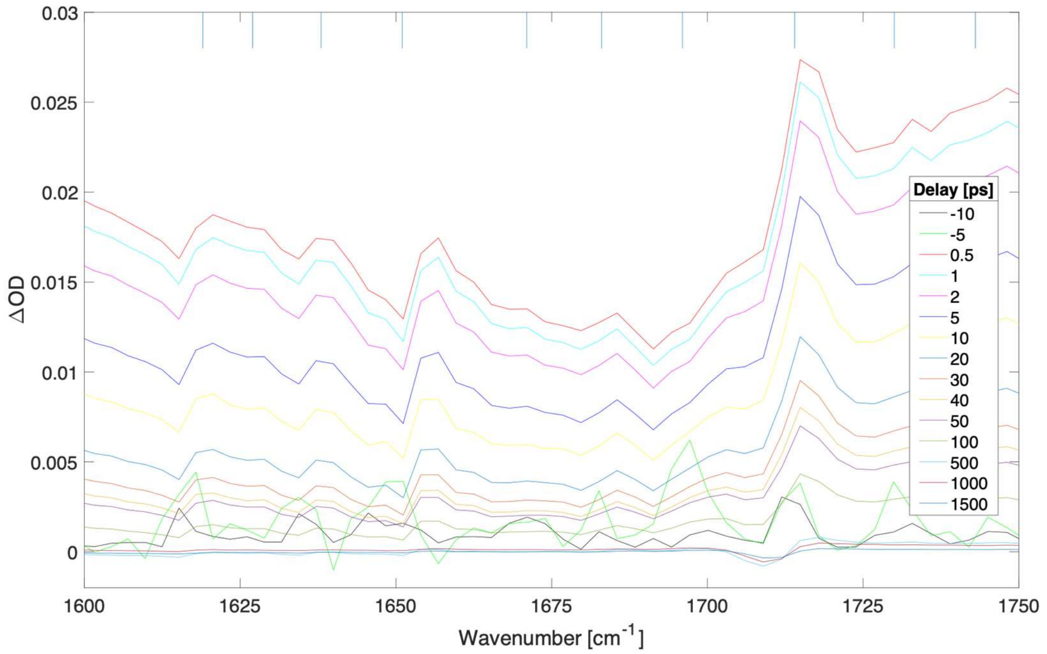

Figure S50.1. Difference transient absorption spectra at selected delays (ps) for a 400 nm thick film of $(FAPbI_3)_{0.97}(MAPbBr_3)_{0.03}$ with pump irradiance of 1.47e9 W/cm^2 (fluence of 367.8 uJ/cm^2) at 539 nm.

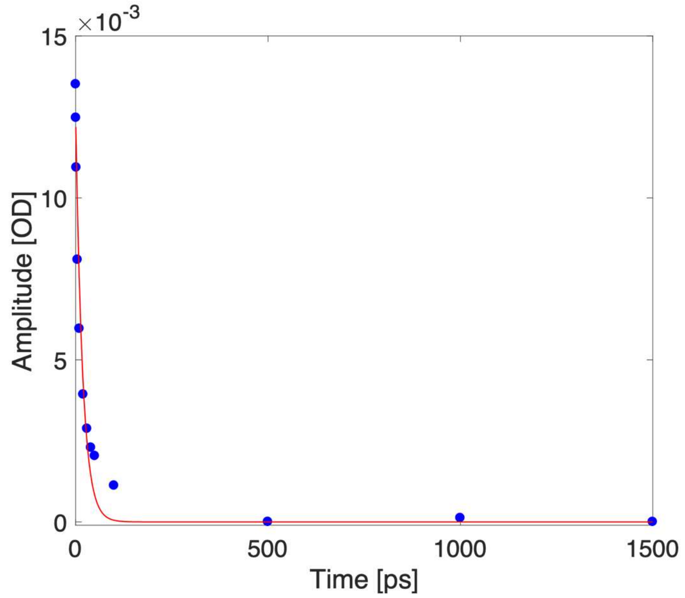

Figure S50.2. Kinetic trace taken at 1670 cm^{-1} (blue dots depict experimental data for selected delays, red curve represents a biexponential fit).

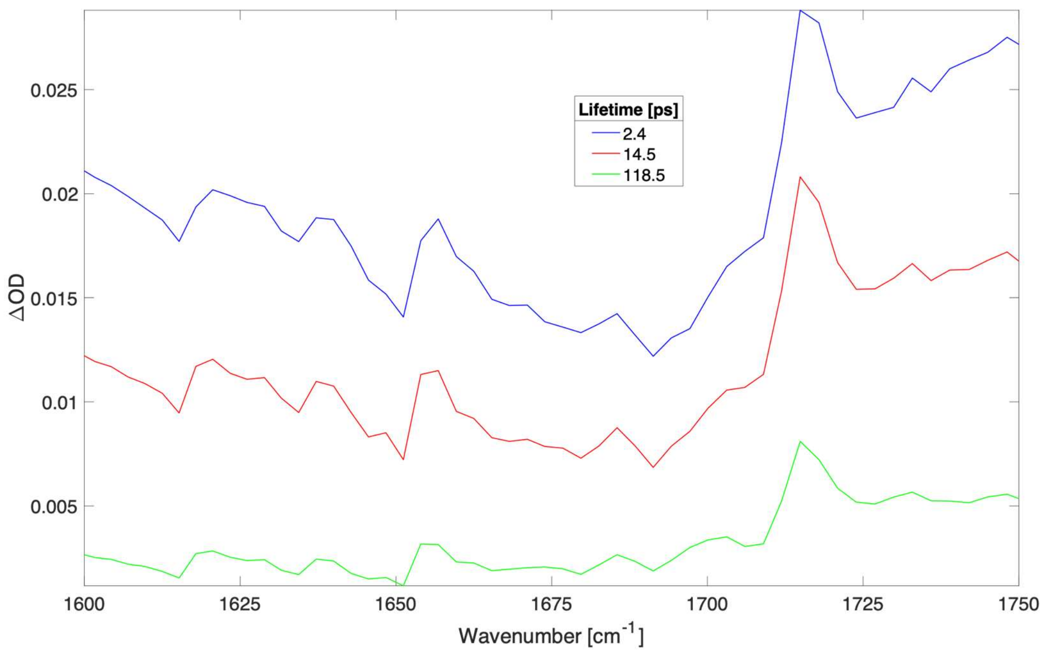

Figure S50.3. Time independent spectra of sequential three compartments global fit to spectra shown in Figure S50.1. The time constants (and contribution to data) were: 2.4 ps (54.1%), 286.7 ps (33.5%), and 1372.0 ps (12.4%).

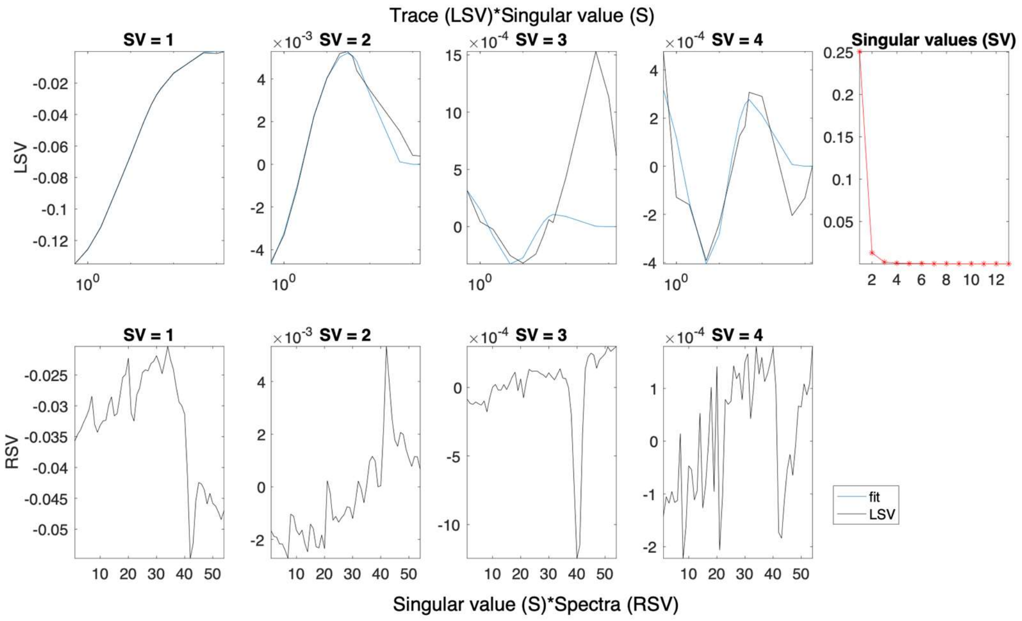

Figure S50.4. Left (LSV) and right (RSV) singular vectors with dominant singular values for the SVD analysis of spectra presented in Figure S50.1.

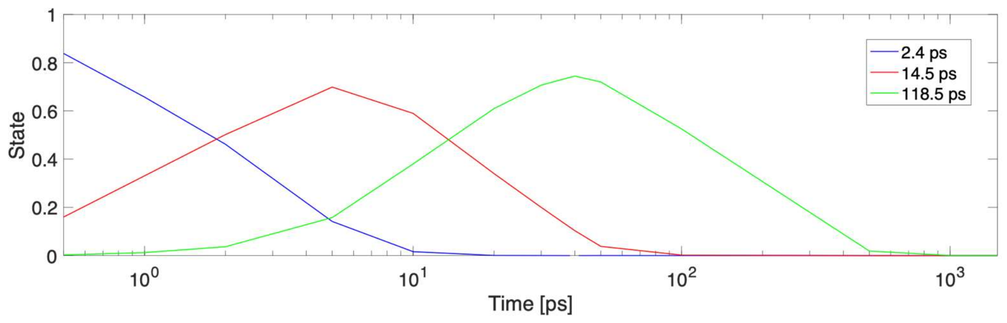

Figure S50.5. Concentration profiles for each time constant fitted to the data in Figure S50.3.

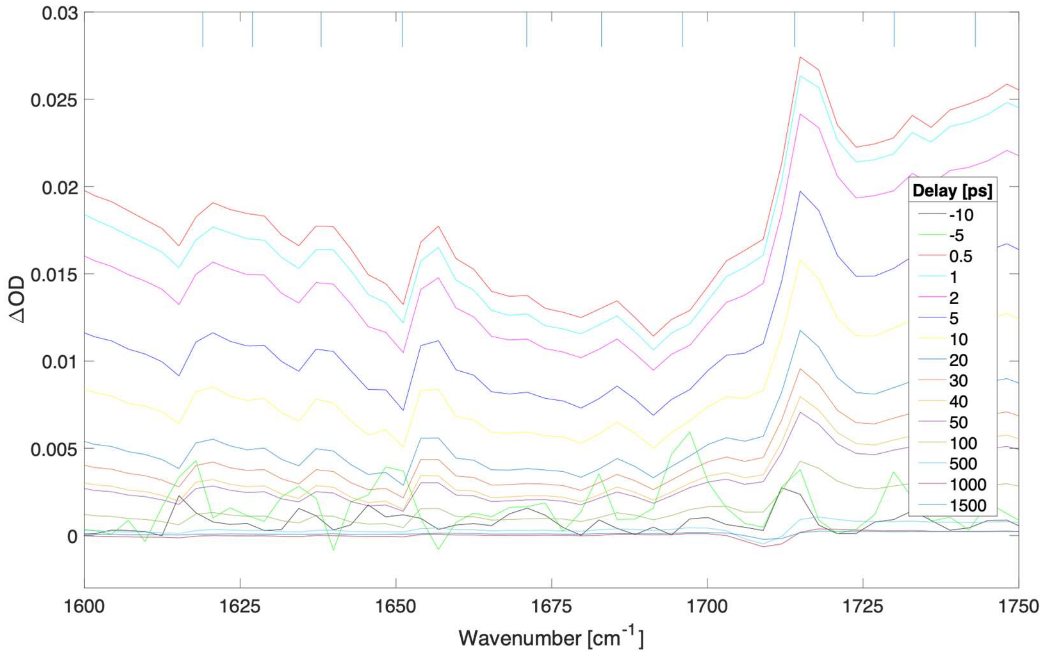

Figure S51.1. Difference transient absorption spectra at selected delays (ps) for a 400 nm thick film of $(FAPbI_3)_{0.97}(MAPbBr_3)_{0.03}$ with pump irradiance of 1.18e9 W/cm^2 (fluence of 294.2 uJ/cm^2) at 539 nm.

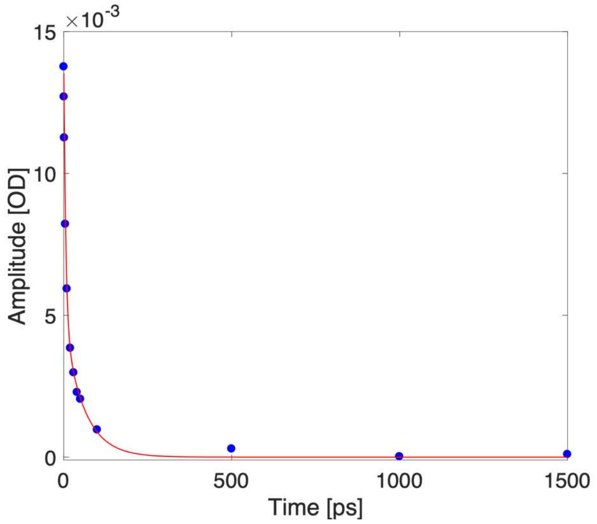

Figure S51.2. Kinetic trace taken at 1670 cm^{-1} (blue dots depict experimental data for selected delays, red curve represents a biexponential fit).

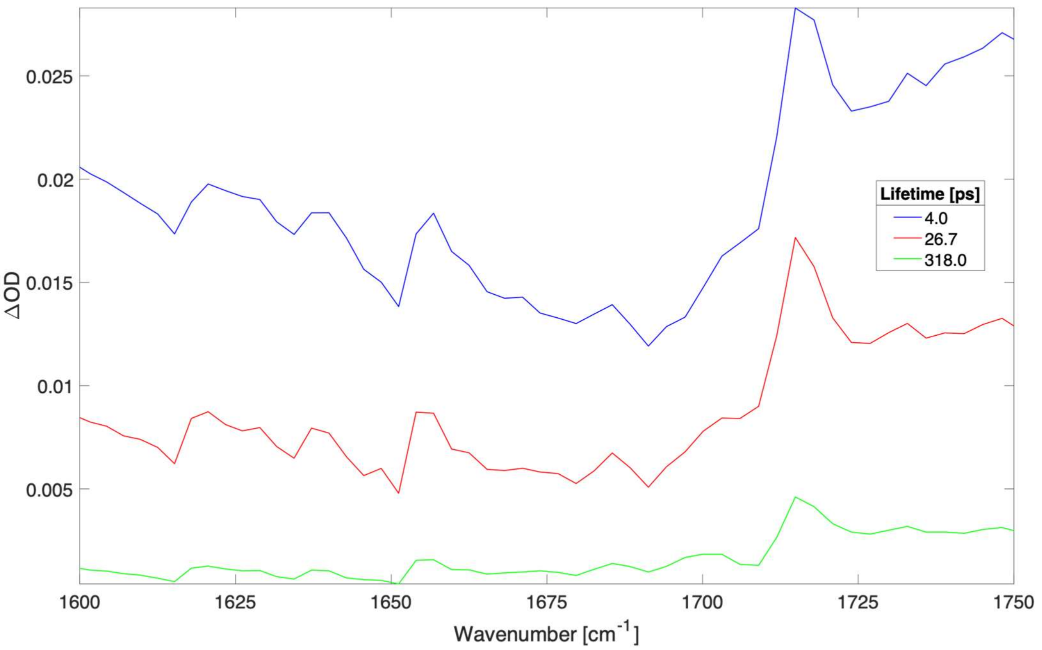

Figure S51.3. Time independent spectra of sequential three compartments global fit to spectra shown in Figure S51.1. The time constants (and contribution to data) were: 4.0 ps (61.5%), 26.7 ps (30.4%), and 318.0 ps (8.1%).

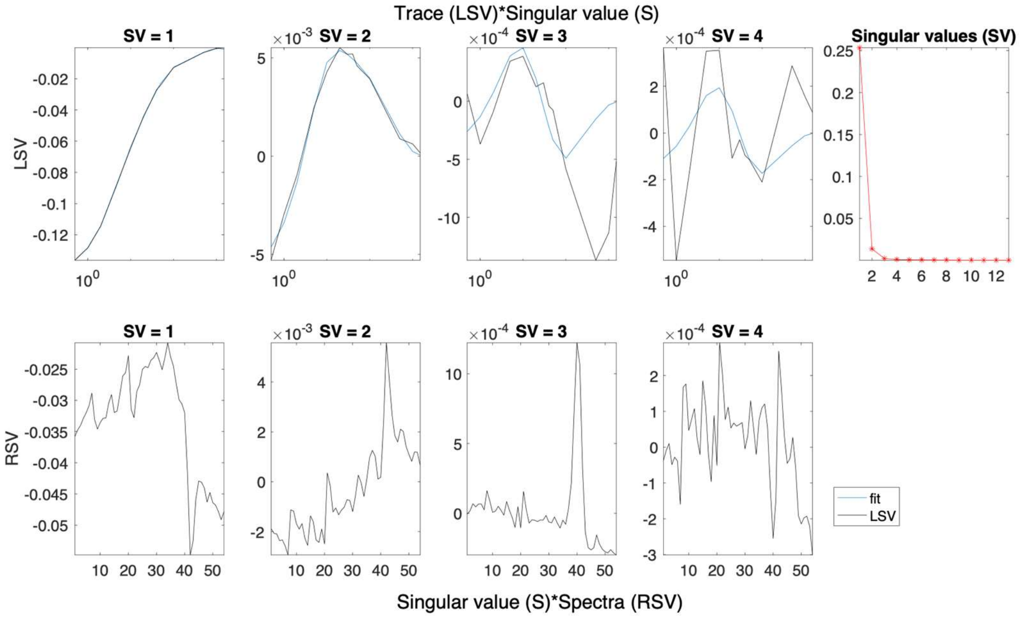

Figure S51.4. Left (LSV) and right (RSV) singular vectors with dominant singular values for the SVD analysis of spectra presented in Figure S51.1.

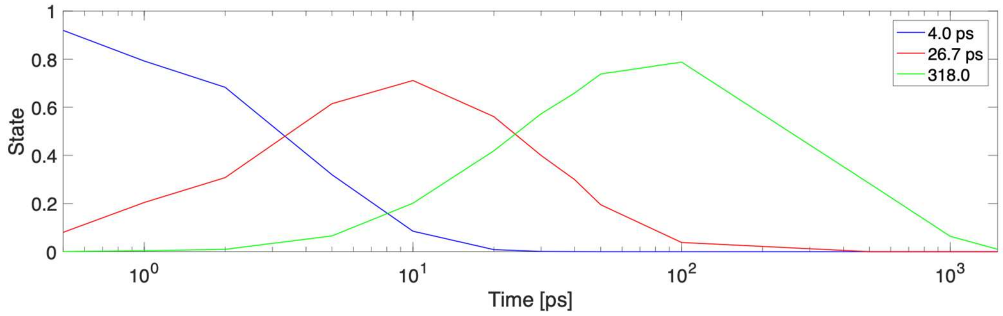

Figure S51.5. Concentration profiles for each time constant fitted to the data in Figure S51.3.

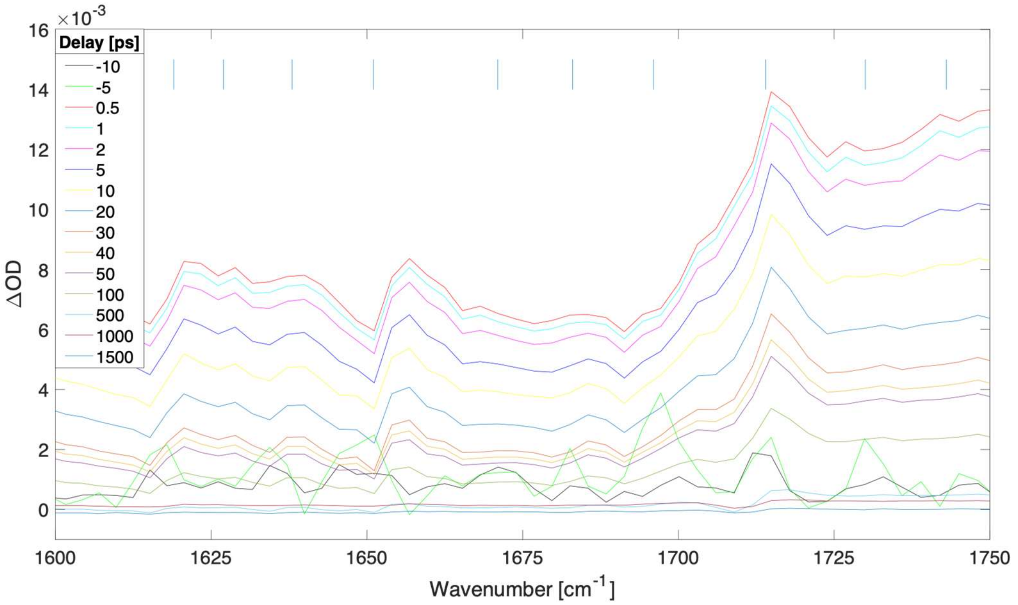

Figure S52.1. Difference transient absorption spectra at selected delays (ps) for a 400 nm thick film of $(FAPbI_3)_{0.97}(MAPbBr_3)_{0.03}$ with pump irradiance of 9.05e8 W/cm^2 (fluence of 226.4 uJ/cm^2) at 539 nm.

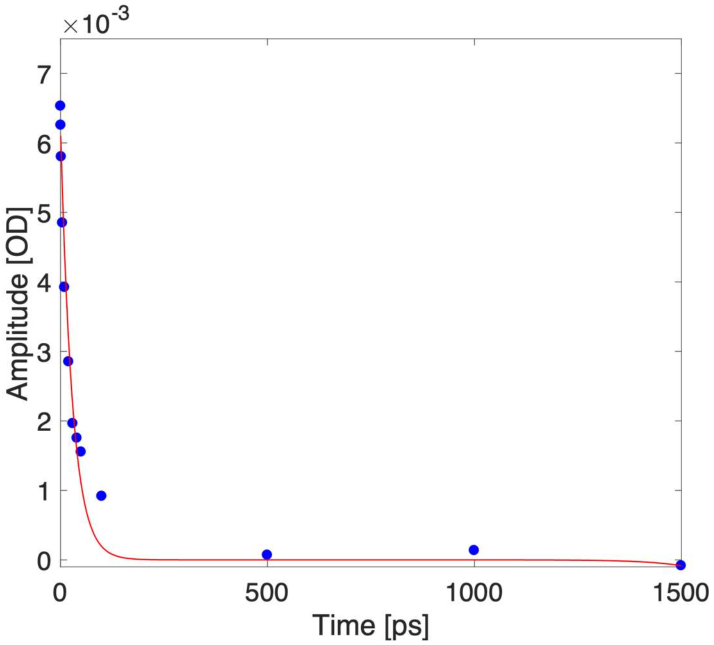

Figure S52.2. Kinetic trace taken at 1670 cm^{-1} (blue dots depict experimental data for selected delays, red curve represents a biexponential fit)

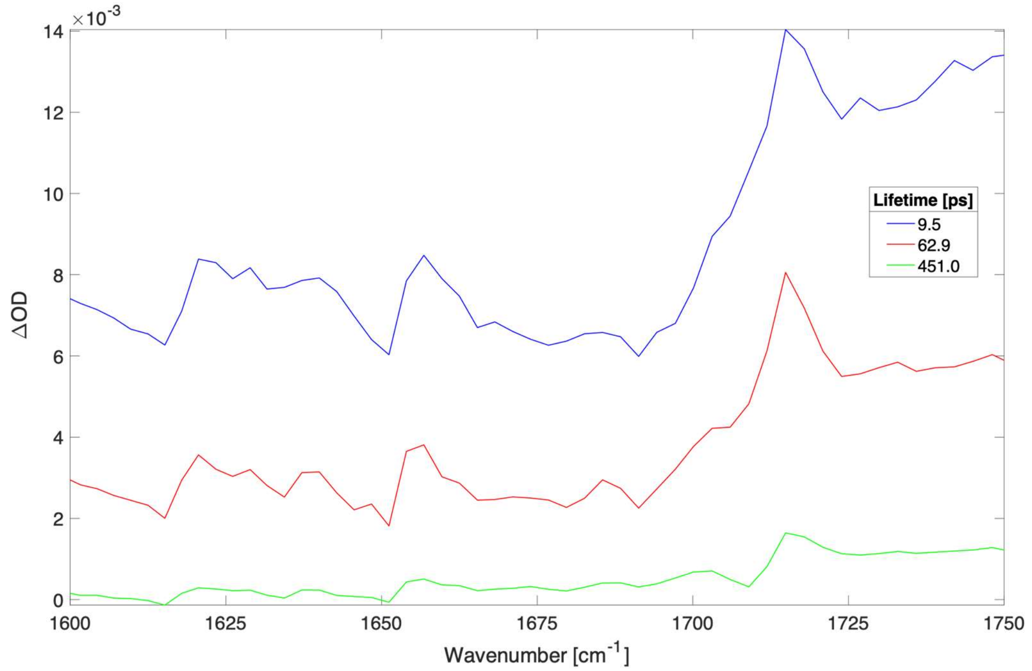

Figure S52.3. Time independent spectra of sequential three compartments global fit to spectra shown in Figure S52.1. The time constants (and contribution to data) were: 9.5 ps (62.5%), 62.9 ps (29.8%), and 451.0 ps (7.7%).

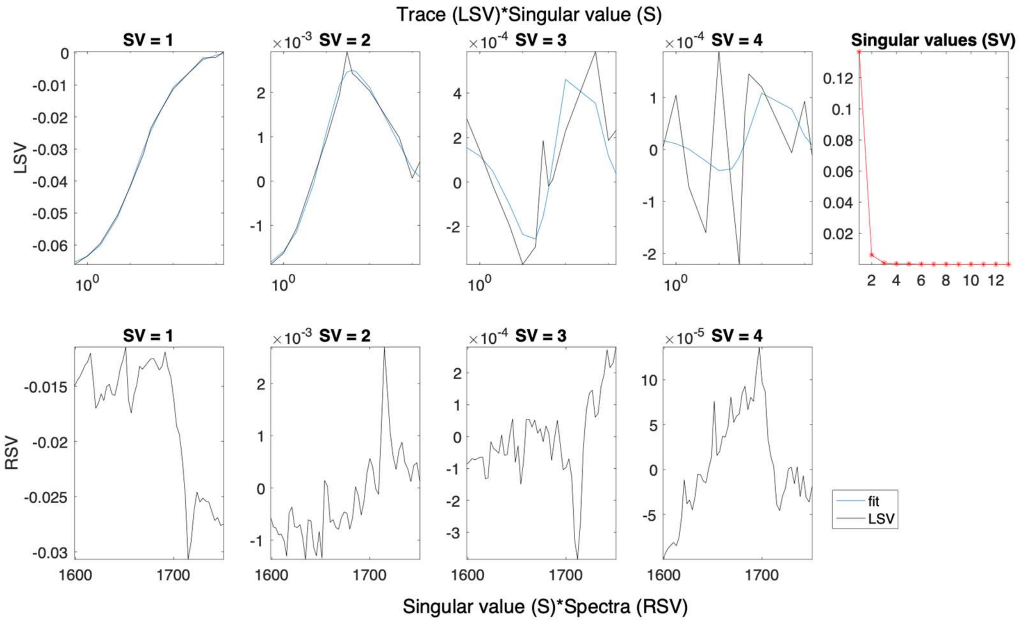

Figure S52.4. Left (LSV) and right (RSV) singular vectors with dominant singular values for the SVD analysis of spectra presented in Figure S52.1.

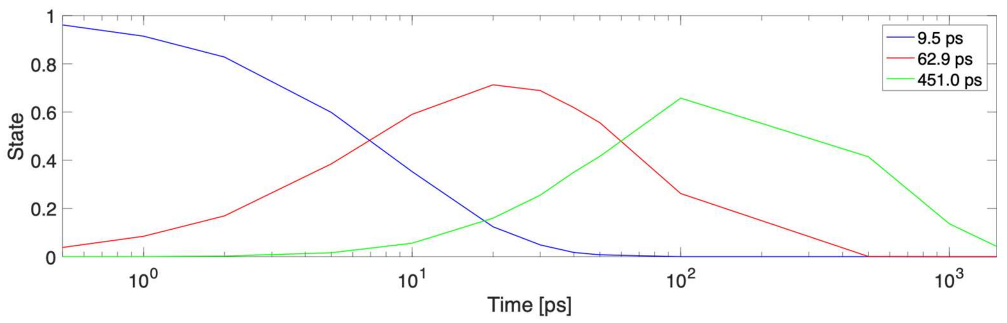

Figure S52.5. Concentration profiles for each time constant fitted to the data in Figure S52.3.

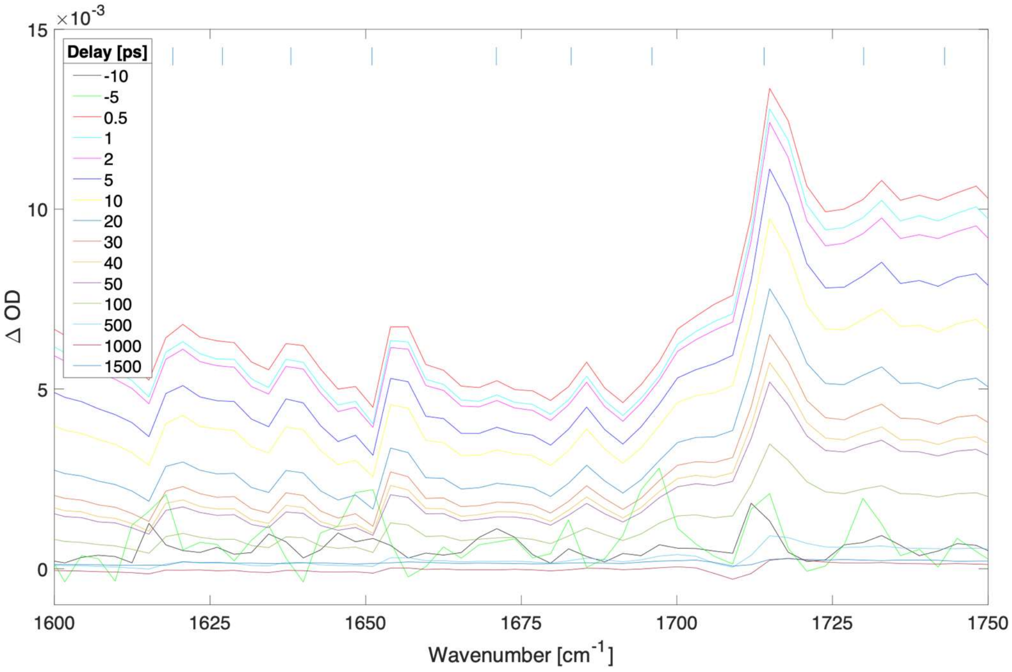

Figure S53.1. Difference transient absorption spectra at selected delays (ps) for a 400 nm thick film of $(FAPbI_3)_{0.97}(MAPbBr_3)_{0.03}$ with pump irradiance of 2.94e8 W/cm^2 (fluence of 73.6 uJ/cm^2) at 539 nm.

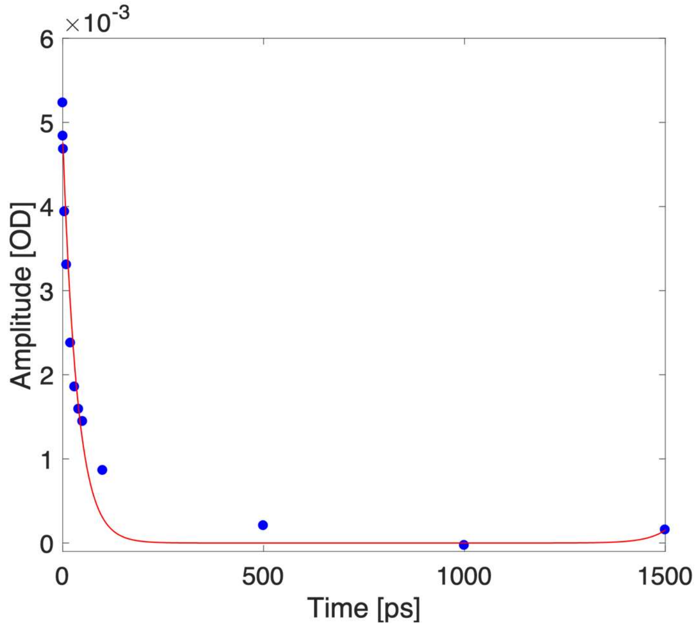

Figure S53.2. Kinetic trace taken at 1670 cm^{-1} (blue dots depict experimental data for selected delays, red curve represents a biexponential fit).

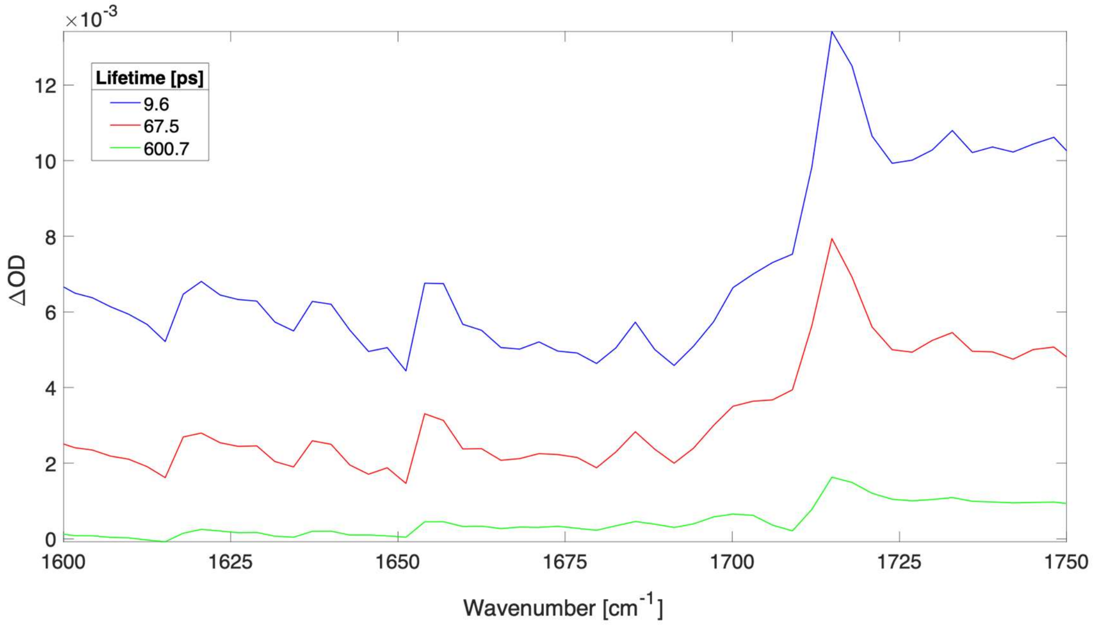

Figure S53.3. Time independent spectra of sequential three compartments global fit to spectra shown in Figure S53.1. The time constants (and contribution to data) were: 9.6 ps (61.1%), 67.5 ps (31.1%), and 600.7 ps (7.8%).

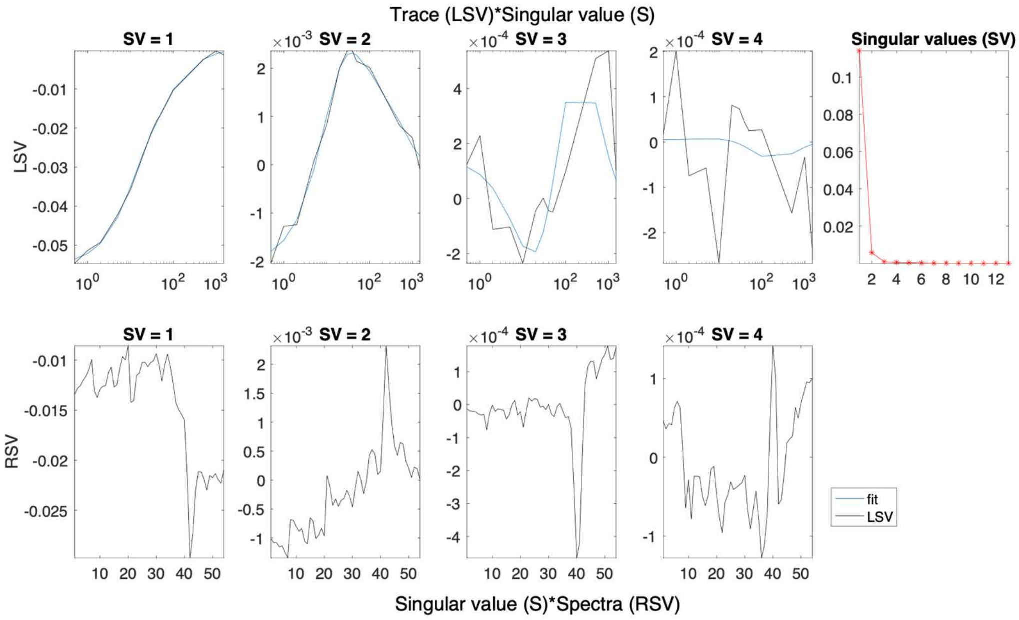

Figure S53.4. Left (LSV) and right (RSV) singular vectors with dominant singular values for the SVD analysis of spectra presented in Figure S53.1.

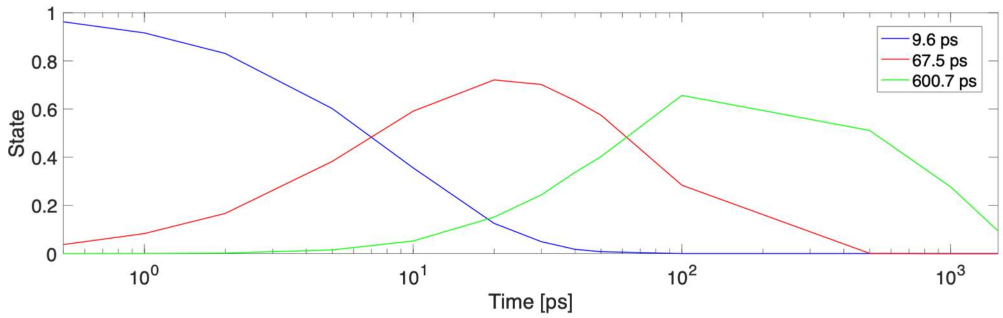

Figure S53.5. Concentration profiles for each time constant fitted to the data in Figure S53.3.

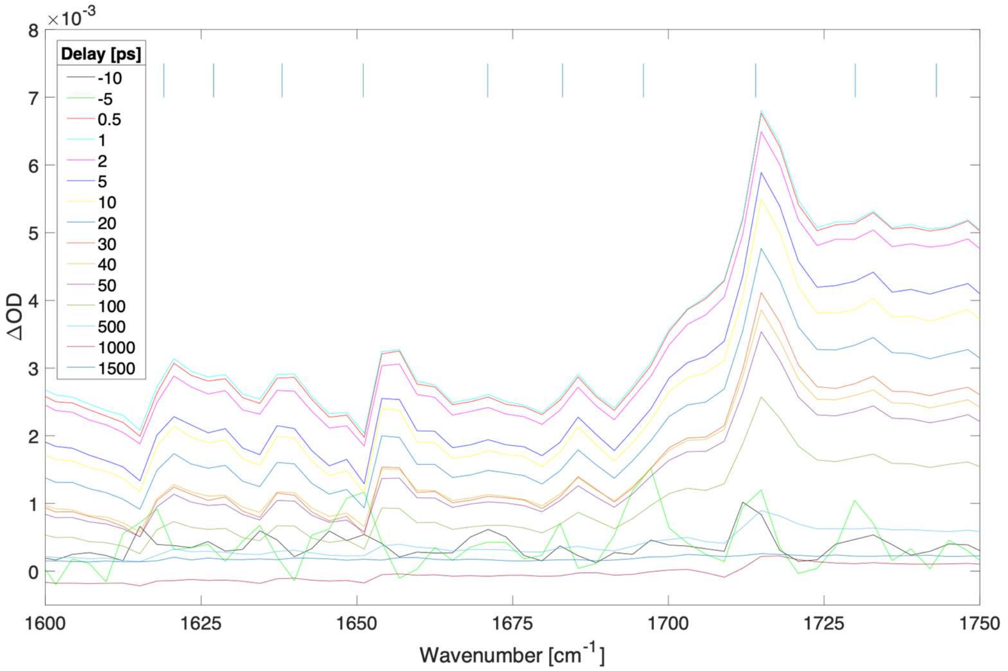

Figure S54.1. Difference transient absorption spectra at selected delays (ps) for a 400 nm thick film of $(FAPbI_3)_{0.97}(MAPbBr_3)_{0.03}$ with pump irradiance of 1.47e8 W/cm^2 (fluence of 36.8 uJ/cm^2) at 539 nm.

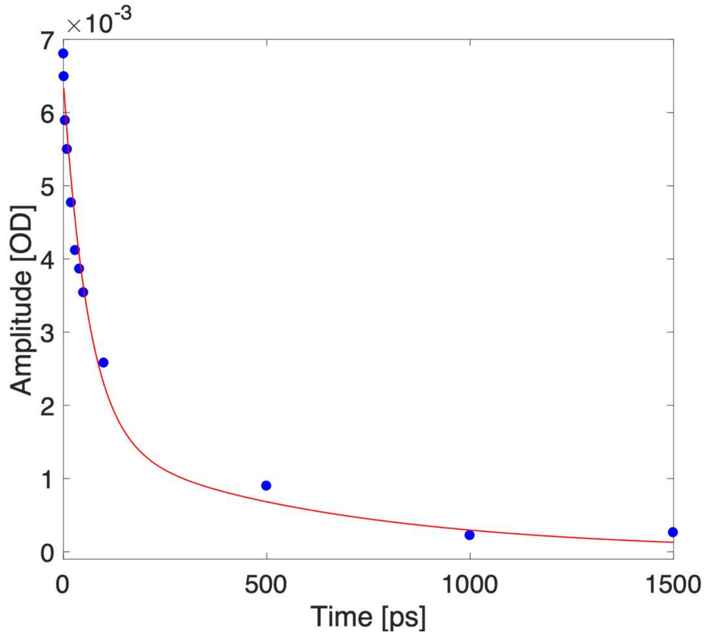

Figure S54.2. Kinetic trace taken at 1670 cm^{-1} (blue dots depict experimental data for selected delays, red curve represents a biexponential fit).

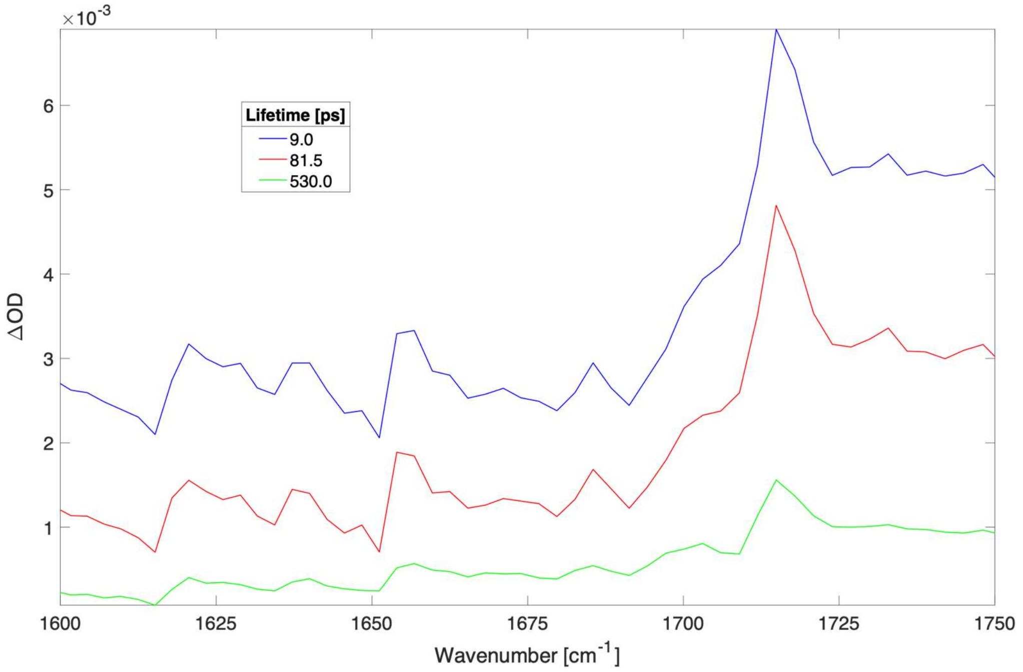

Figure S54.3. Time independent spectra of sequential three compartments global fit to spectra shown in Figure S54.1. The time constants (and contribution to data) were: 19.4 ps (54.6%), 286.7 ps (33.1%), and 530.0 ps (12.3%).

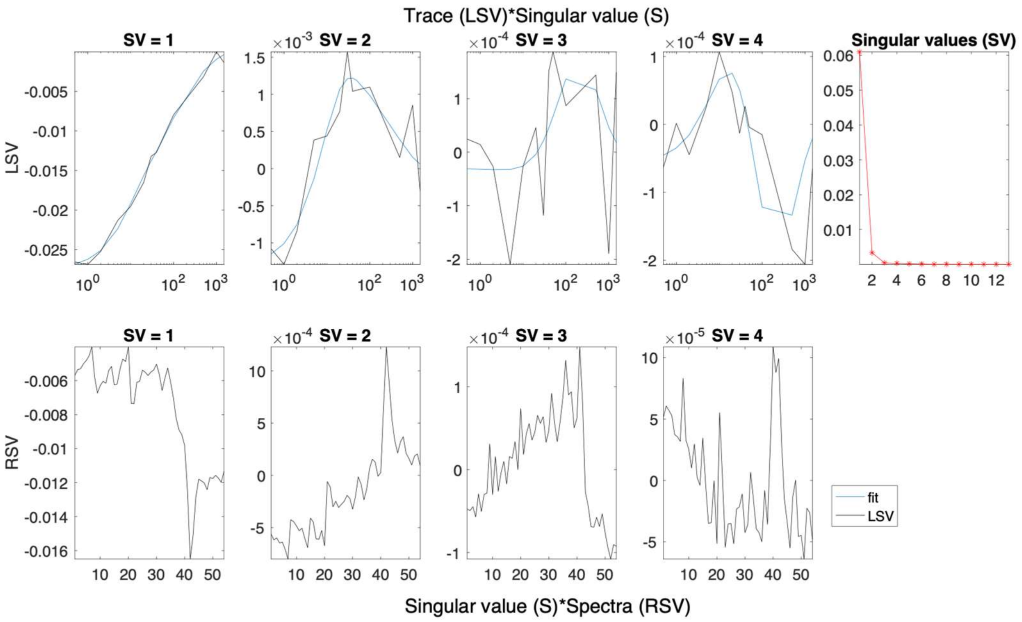

Figure S54.4. Left (LSV) and right (RSV) singular vectors with dominant singular values for the SVD analysis of spectra presented in Figure S54.1.

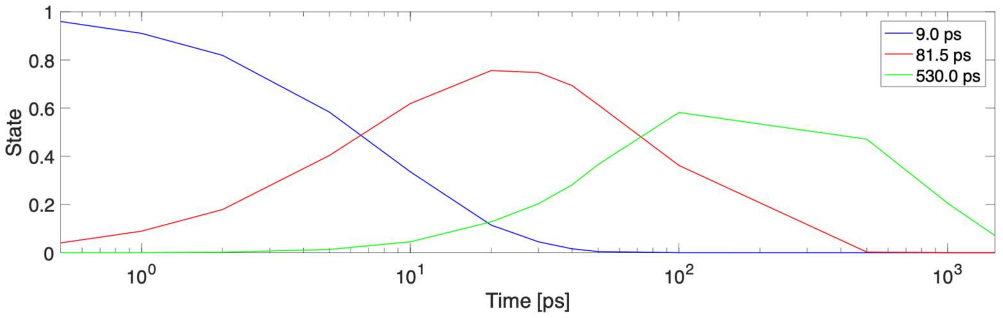

Figure S54.5. Concentration profiles for each time constant fitted to the data in Figure S54.3.

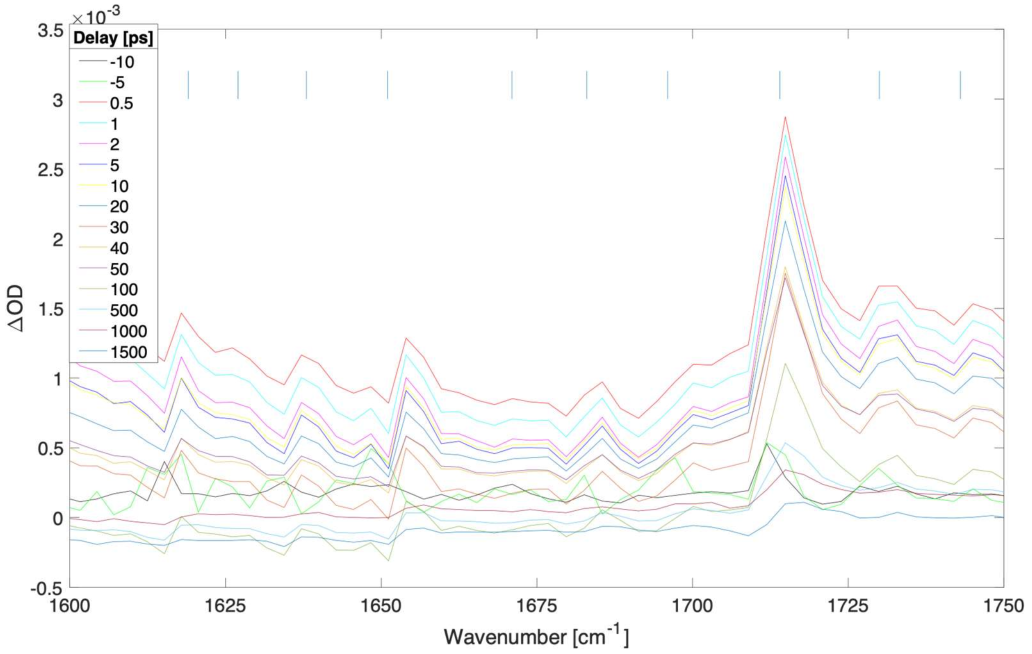

Figure S55.1. Difference transient absorption spectra at selected delays (ps) for a 400 nm thick film of $(FAPbI_3)_{0.97}(MAPbBr_3)_{0.03}$ with pump irradiance of 8.9e7 W/cm^2 (fluence of 22.4 uJ/cm^2) at 539 nm.

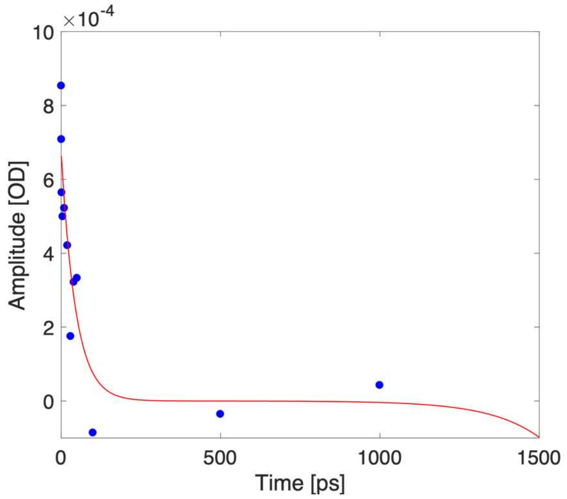

Figure S55.2. Kinetic trace taken at 1670 cm^{-1} (blue dots depict experimental data for selected delays, red curve represents a biexponential fit).

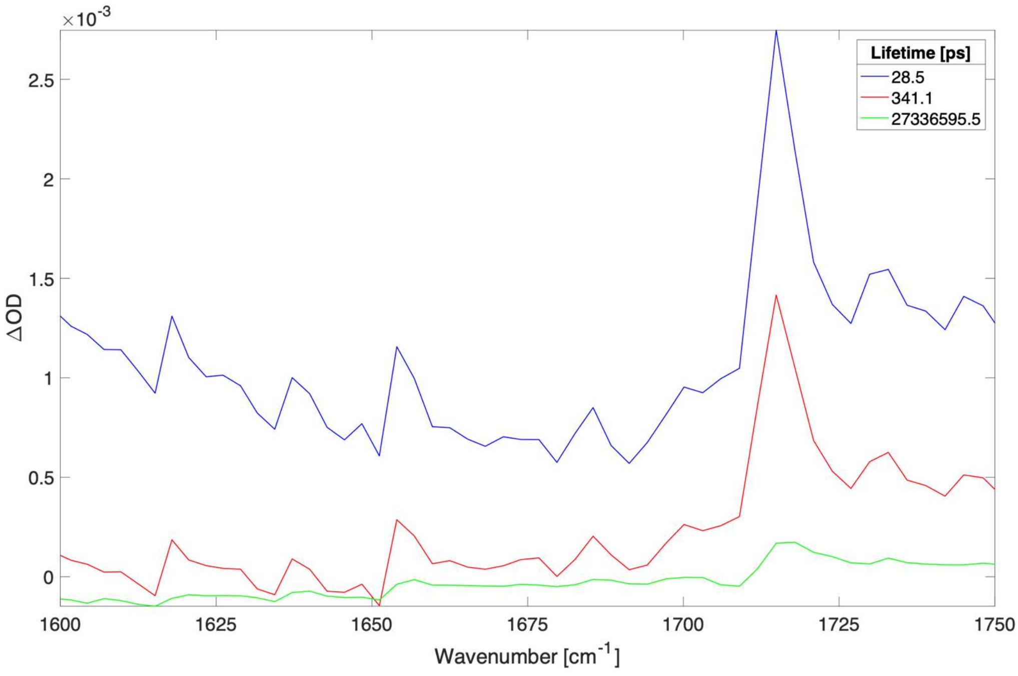

Figure S55.3. Time independent spectra of sequential three compartments global fit to spectra shown in Figure S55.1. The time constants (and contribution to data) were: 28.5 ps (65.0%), 341.5 ps (28.8%), and 27,336,595.5 ps (6.2%).

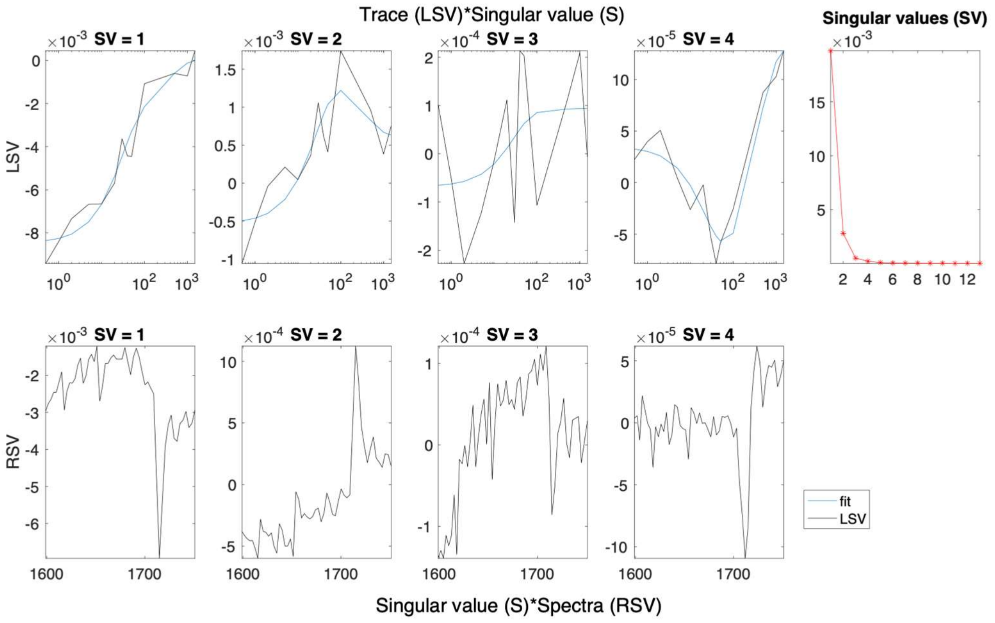

Figure S55.4. Left (LSV) and right (RSV) singular vectors with dominant singular values for the SVD analysis of spectra presented in Figure S55.1.

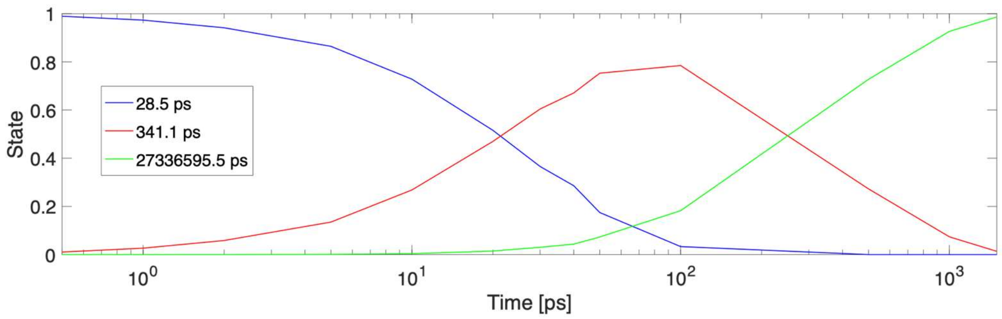

Figure S55.5. Concentration profiles for each time constant fitted to the data in Figure S55.2.

Time-resolved charge recombination analysis upon optical excitation of 200 nm thick film of $MAPbBr_3$

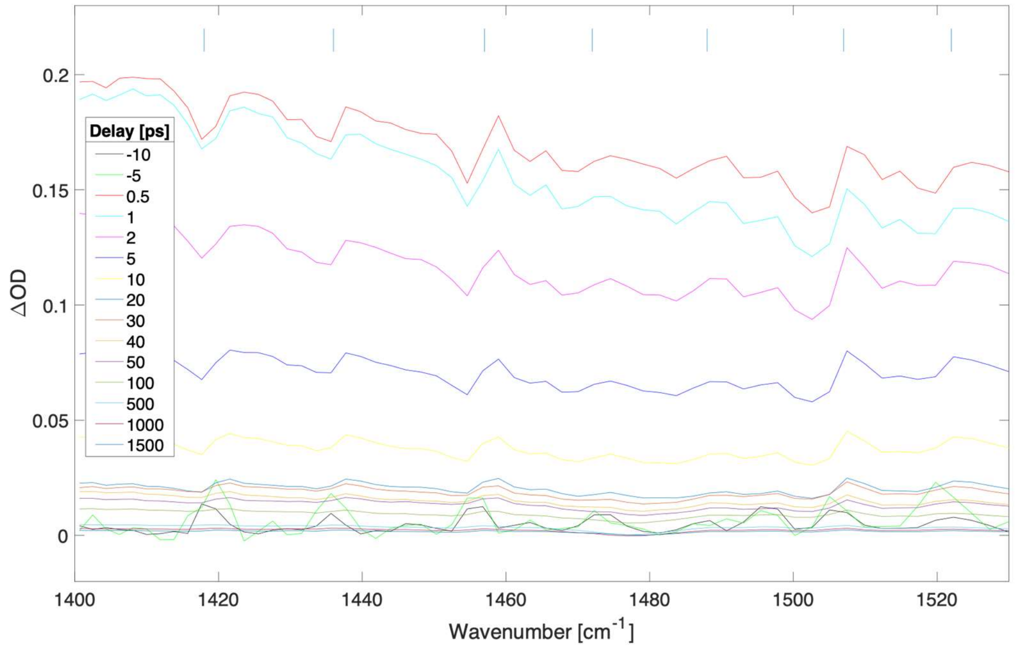

Figure S56.1. Difference transient absorption spectra at selected delays (ps) for a 200 nm thick film of $MAPbBr_3$ with pump irradiance of 7.11e10 W/cm^2 (fluence of 17,768.8 uJ/cm^2) at 539 nm.

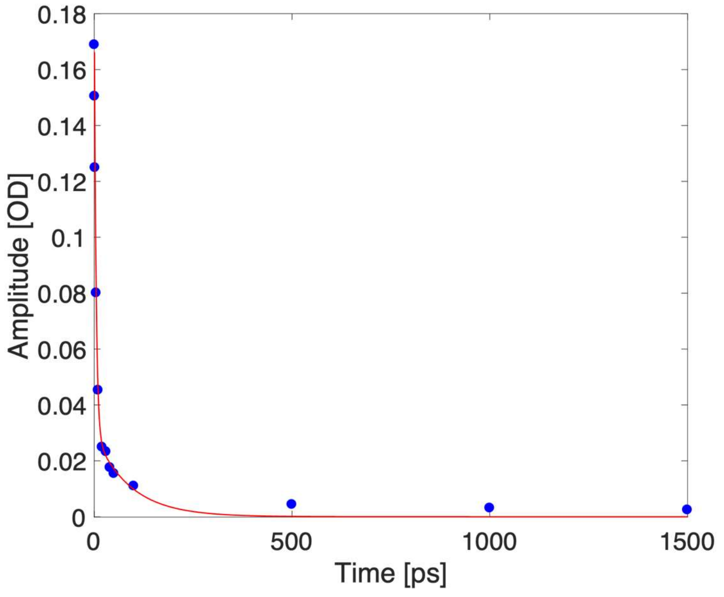

Figure S56.2. Kinetic trace taken at 1444 cm^{-1} (blue dots depict experimental data for selected delays, red curve represents a biexponential fit).

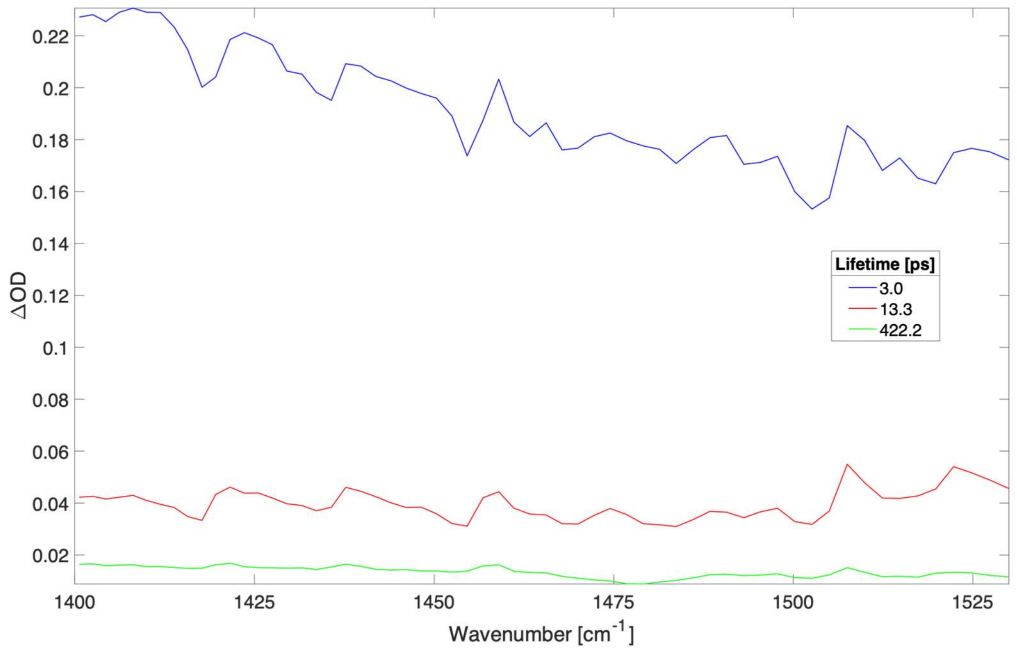

Figure S56.3. Time independent spectra of sequential three compartments global fit to spectra shown in Figure S56.1. The time constants (and contribution to data) were: 3.0 ps (74.8%), 13.3 ps (19.7%), and 422.2 ps (5.5%).

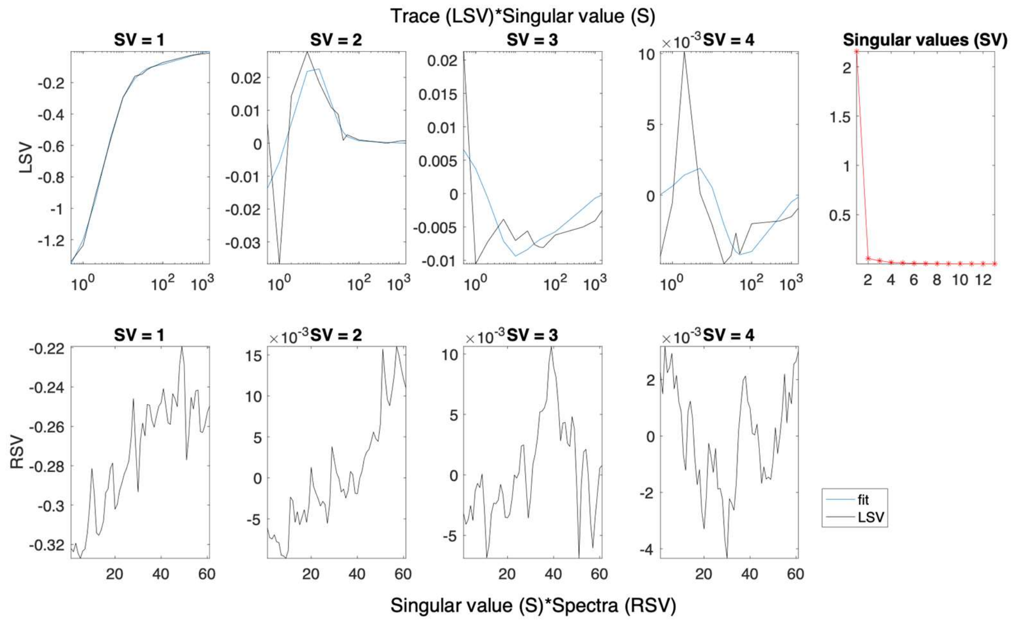

Figure S56.4. Left (LSV) and right (RSV) singular vectors with dominant singular values for the SVD analysis of spectra presented in Figure S56.1.

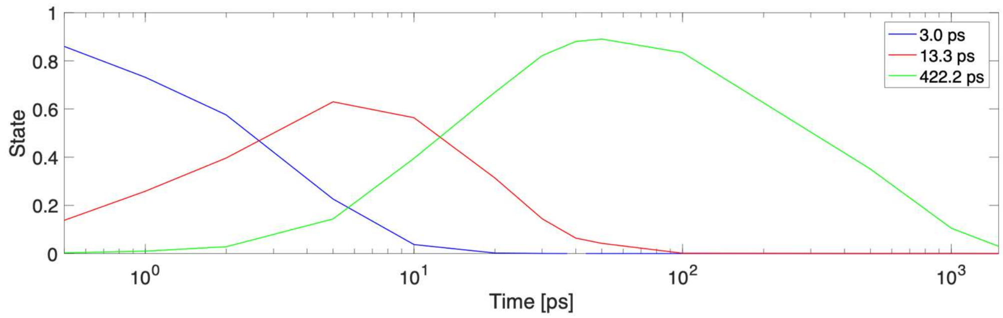

Figure S56.5. Concentration profiles for each time constant fitted to the data in Figure S56.3.

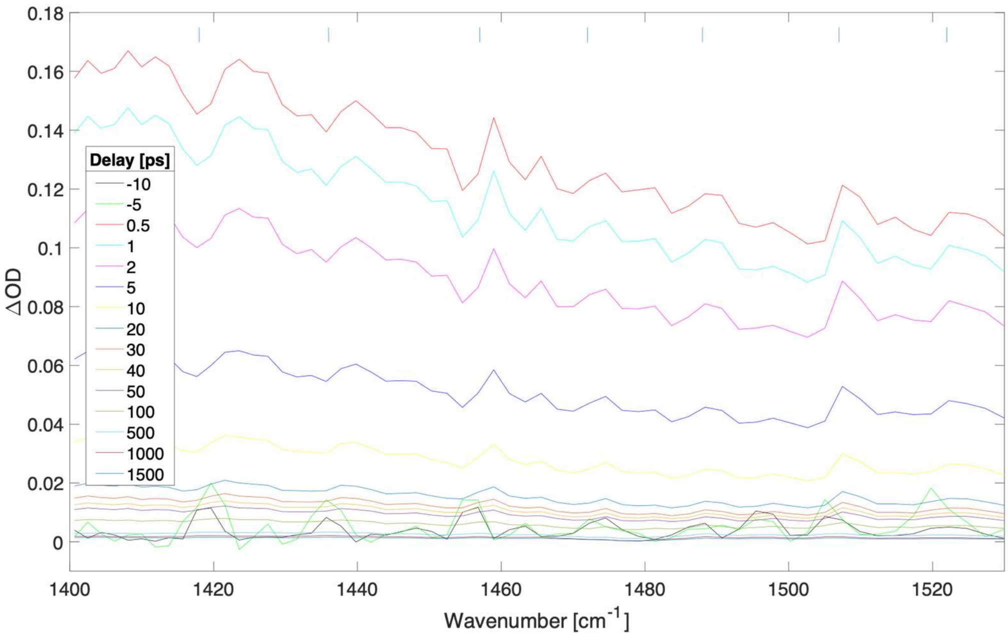

Figure S57.1. Difference transient absorption spectra at selected delays (ps) for a 200 nm thick film of $MAPbBr_3$ with pump irradiance of 4.48e10 W/cm^2 (fluence of 11,204.5 uJ/cm^2) at 539 nm.

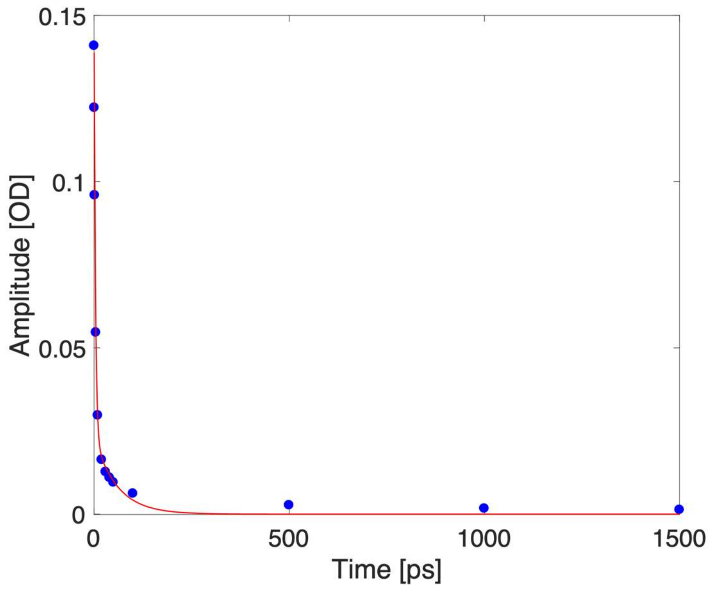

Figure S57.2. Kinetic trace taken at 1444 cm^{-1} (blue dots depict experimental data for selected delays, red curve represents a biexponential fit).

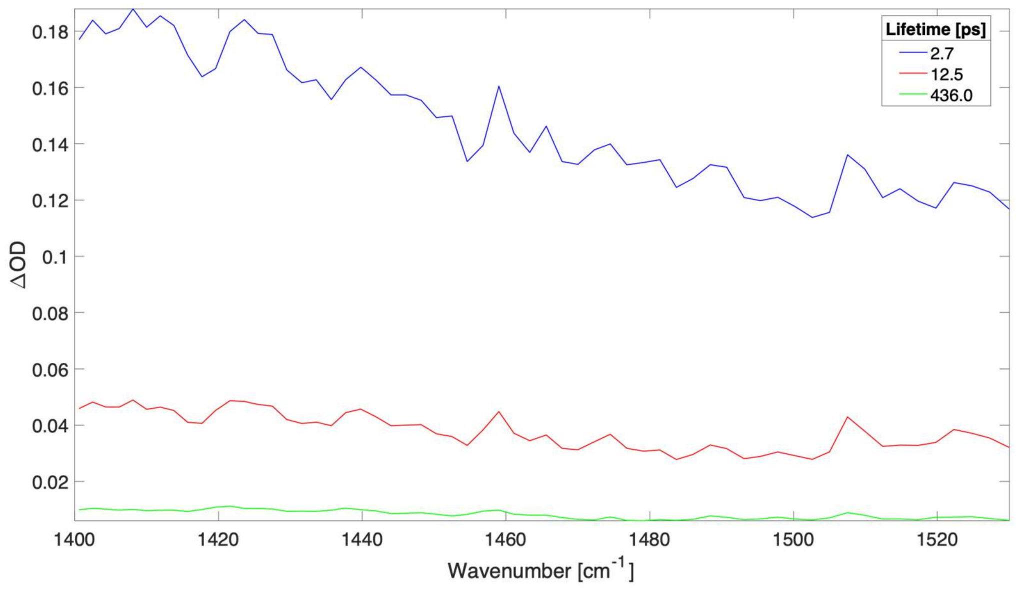

Figure S57.3. Time independent spectra of sequential three compartments global fit to spectra shown in Figure S57.1. The time constants (and contribution to data) were: 2.7 ps (74.8%), 12.5 ps (20.4%), and 436.0 ps (4.8%).

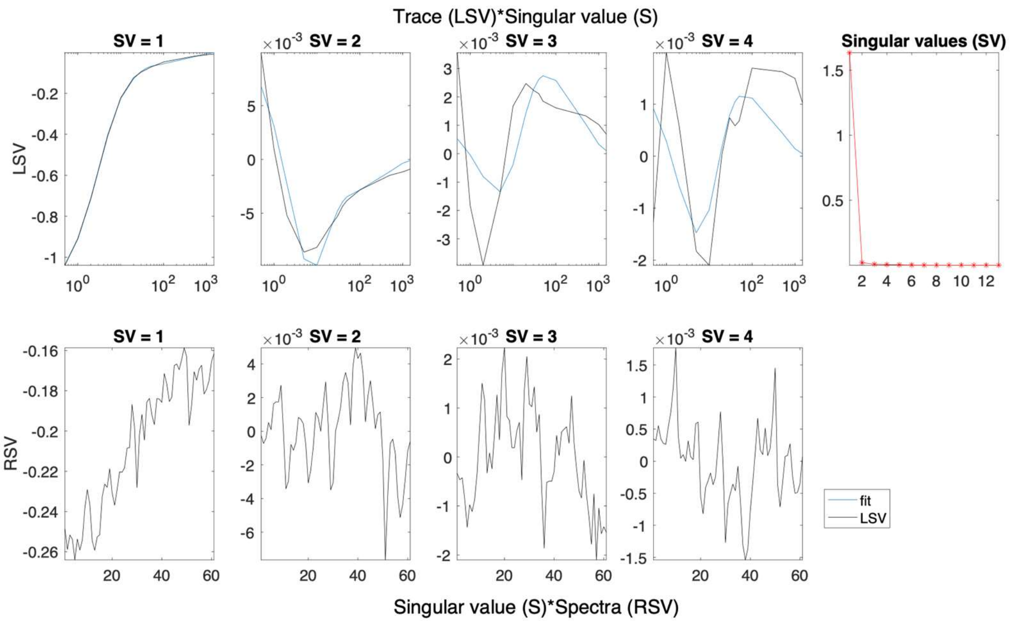

Figure S57.4. Left (LSV) and right (RSV) singular vectors with dominant singular values for the SVD analysis of spectra presented in Figure S57.1.

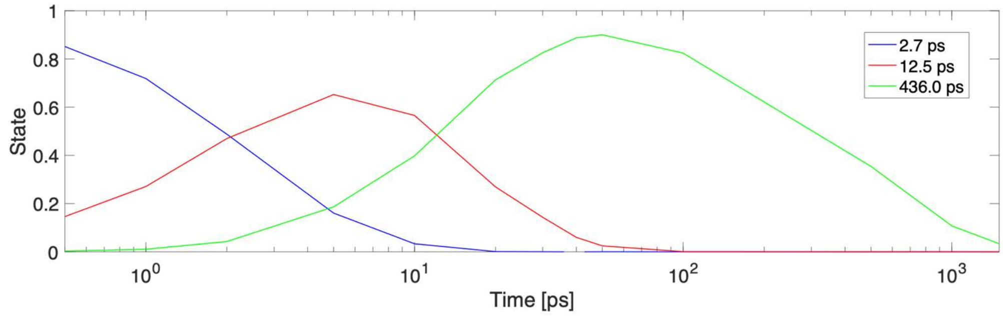

Figure S57.5. Concentration profiles for each time constant fitted to the data in Figure S57.3.

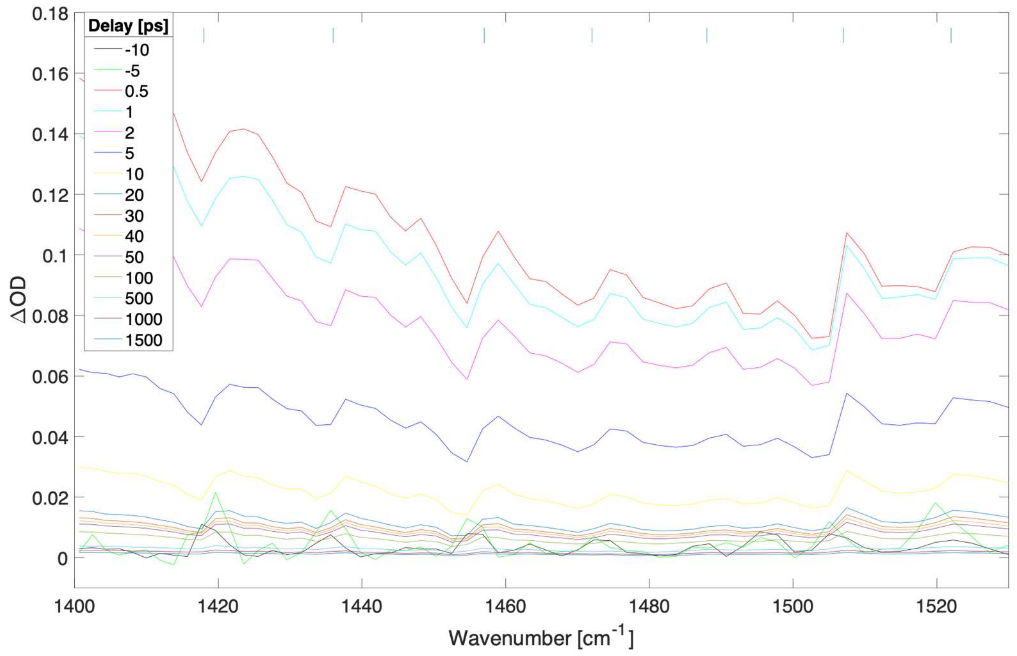

Figure S58.1. Difference transient absorption spectra at selected delays (ps) for a 200 nm thick film of $MAPbBr_3$with pump irradiance of 2.83e10 W/cm^2 (fluence of 7,073.6 uJ/cm^2) at 539 nm.

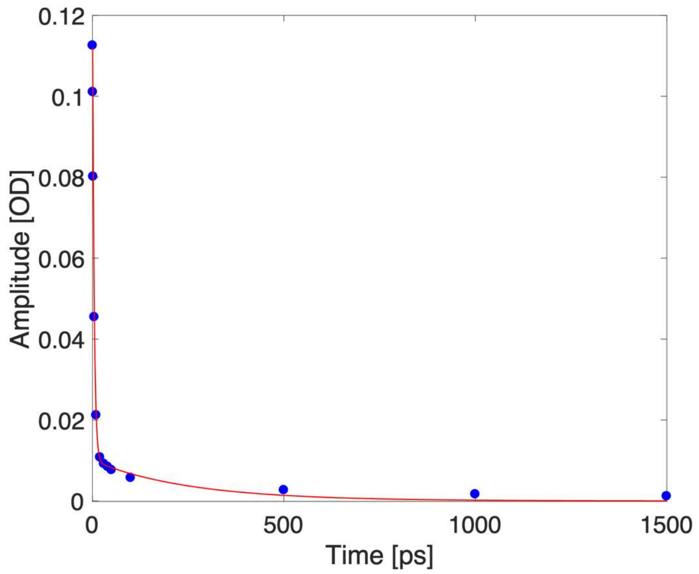

Figure S58.2. Kinetic trace taken at 1444 cm^{-1} (blue dots depict experimental data for selected delays, red curve represents a biexponential fit).

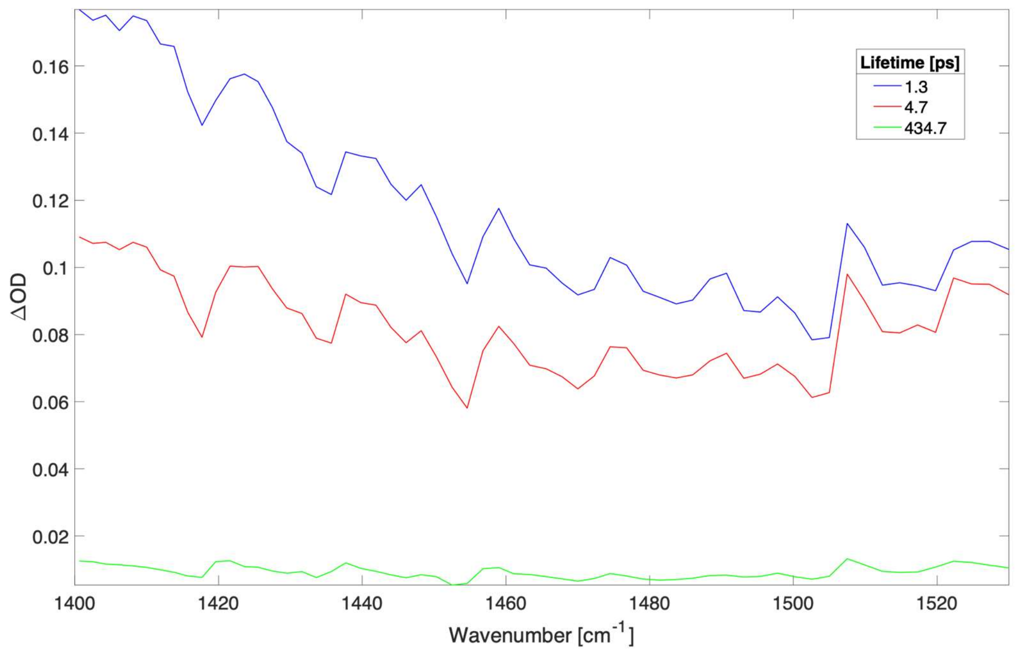

Figure S58.3. Time independent spectra of sequential three compartments global fit to spectra shown in Figure S58.1. The time constants (and contribution to data) were: 1.3 ps (56.2%), 4.7 ps (38.9%), and 434.7 ps (4.9%).

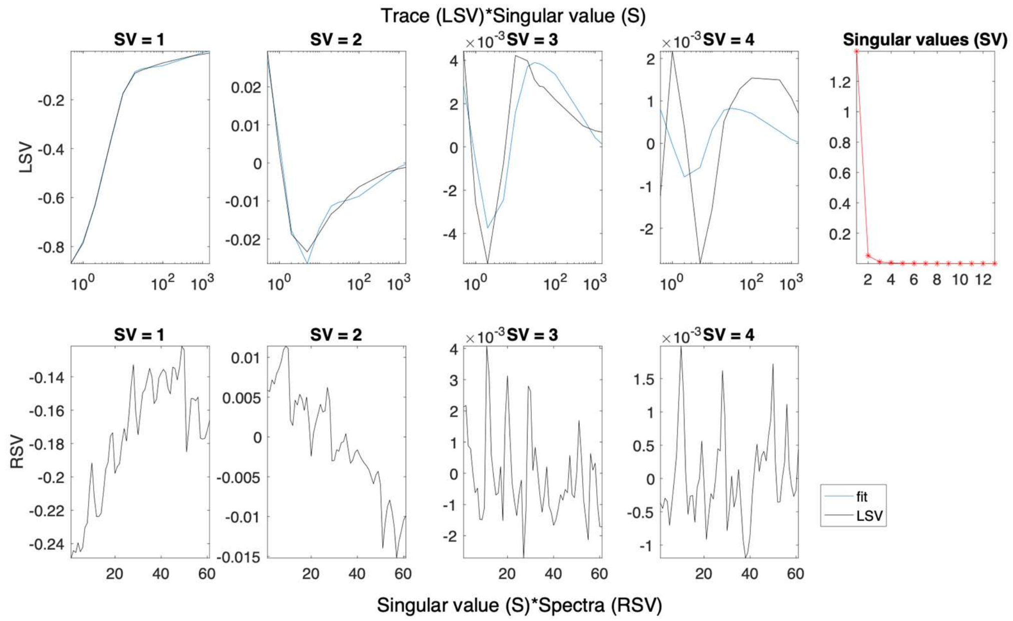

Figure S58.4. Left (LSV) and right (RSV) singular vectors with dominant singular values for the SVD analysis of spectra presented in Figure S58.1.

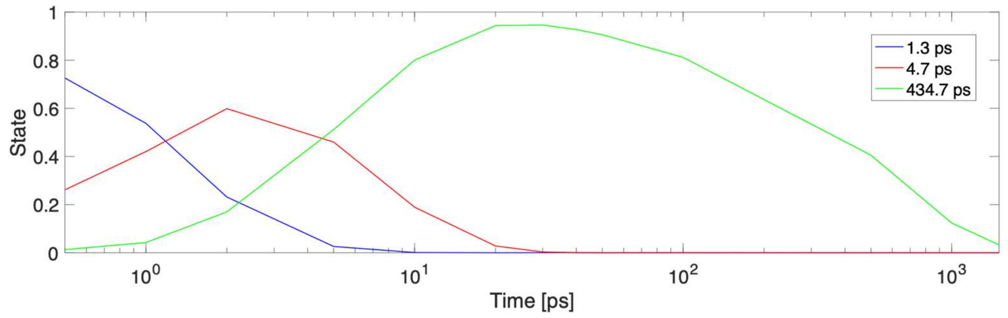

Figure S58.5. Concentration profiles for each time constant fitted to the data in Figure S58.3.

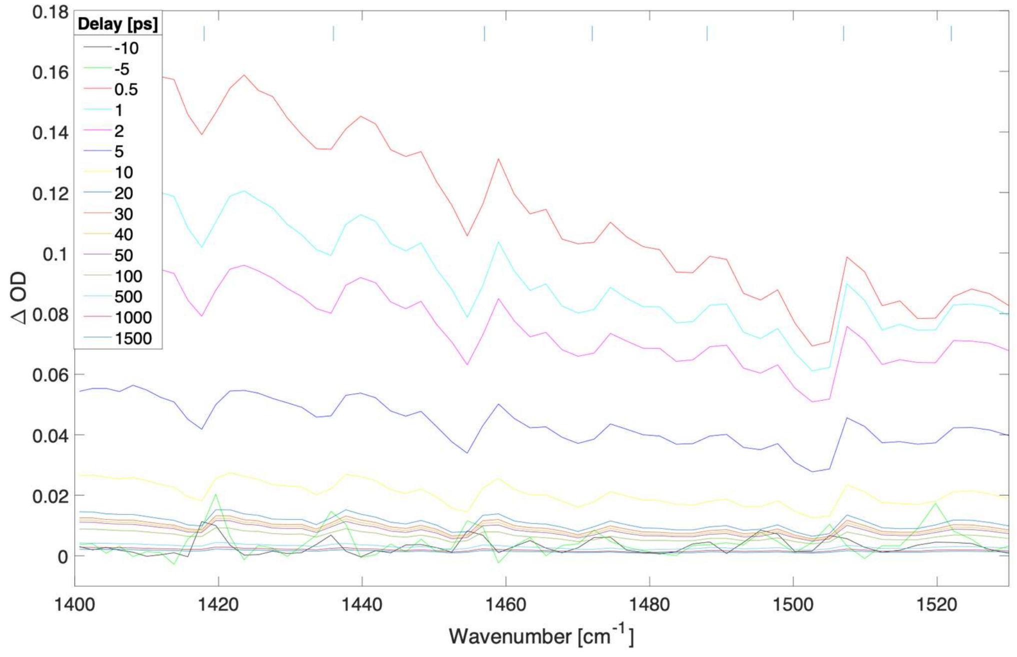

Figure S59.1. Difference transient absorption spectra at selected delays (ps) for a 200 nm thick film of $MAPbBr_3$ with pump irradiance of 1.78.9e10 W/cm^2 (fluence of 4,470.5 uJ/cm^2) at 539 nm.

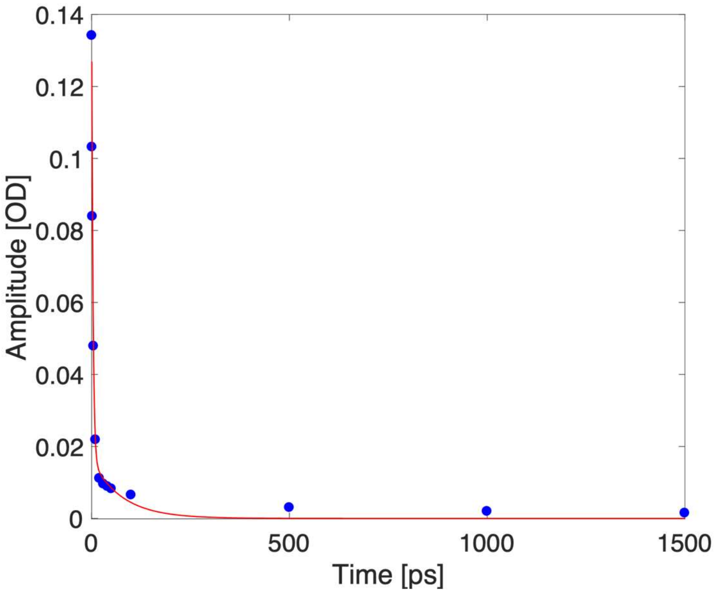

Figure S59.2. Kinetic trace taken at 1444 cm^{-1} (blue dots depict experimental data for selected delays, red curve represents a biexponential fit).

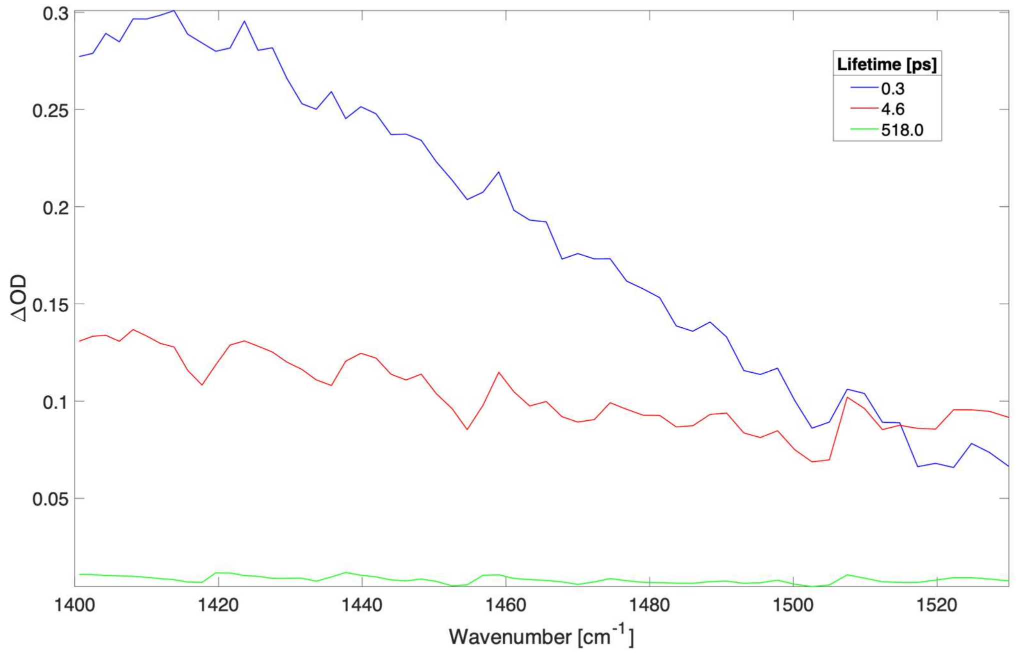

Figure S59.3. Time independent spectra of sequential three compartments global fit to spectra shown in Figure S59.1. The time constants (and contribution to data) were: 0.3 ps (70.5%), 4.6 ps (26.8%), and 518.0 ps (2.7%).

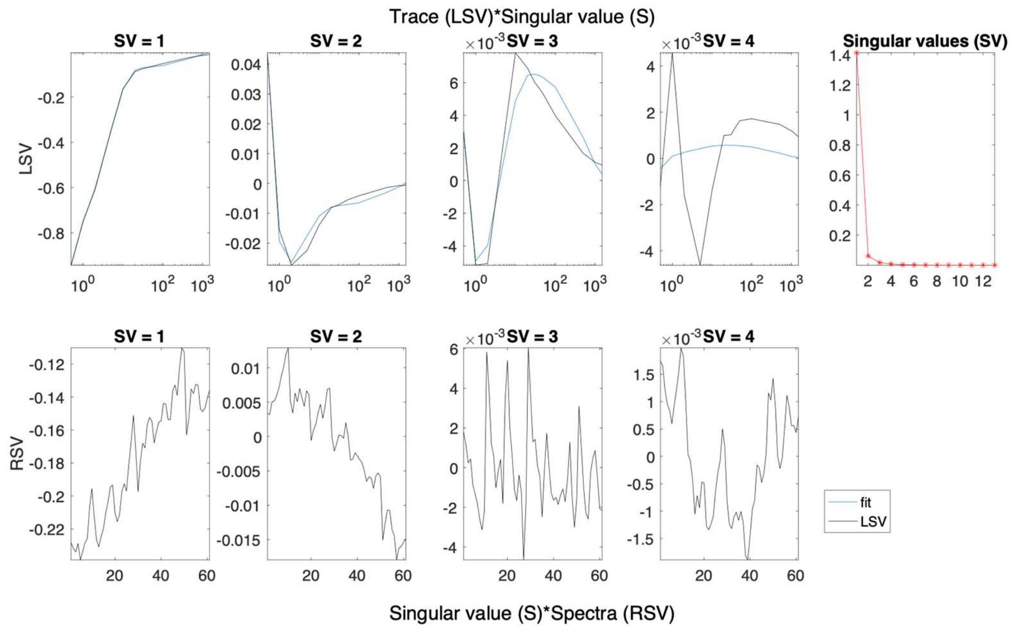

Figure S59.4. Left (LSV) and right (RSV) singular vectors with dominant singular values for the SVD analysis of spectra presented in Figure S59.1.

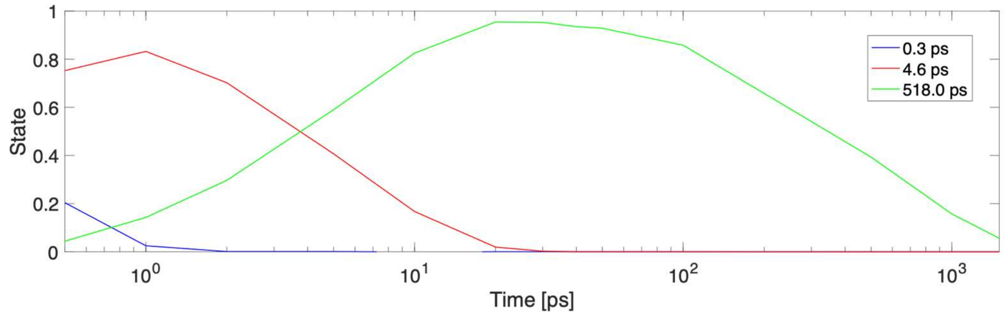

Figure S59.5. Concentration profiles for each time constant fitted to the data in Figure S59.3.

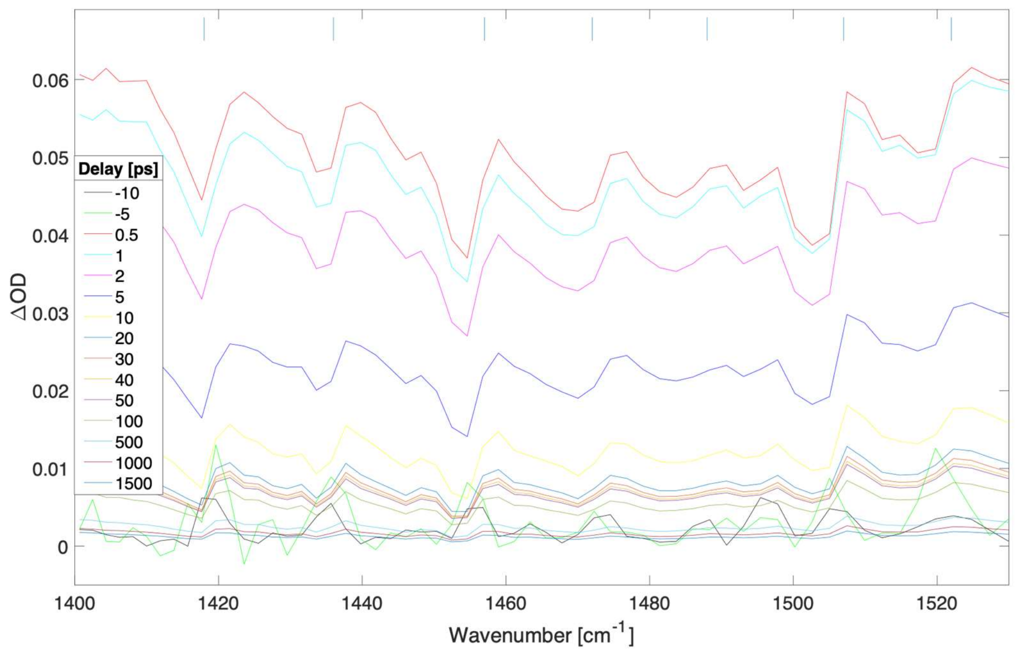

Figure S60.1. Difference transient absorption spectra at selected delays (ps) for a 200 nm thick film of $MAPbBr_3$ with pump irradiance of 1.13e10 W/cm^2 (fluence of 2,829.4uJ/cm^2) at 539 nm.

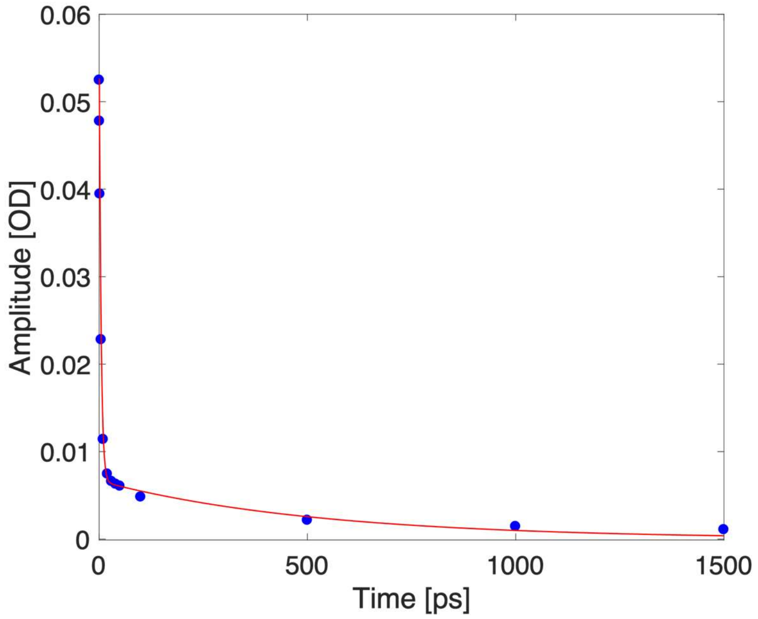

Figure S60.2. Kinetic trace taken at 1444 cm^{-1} (blue dots depict experimental data for selected delays, red curve represents a biexponential fit).

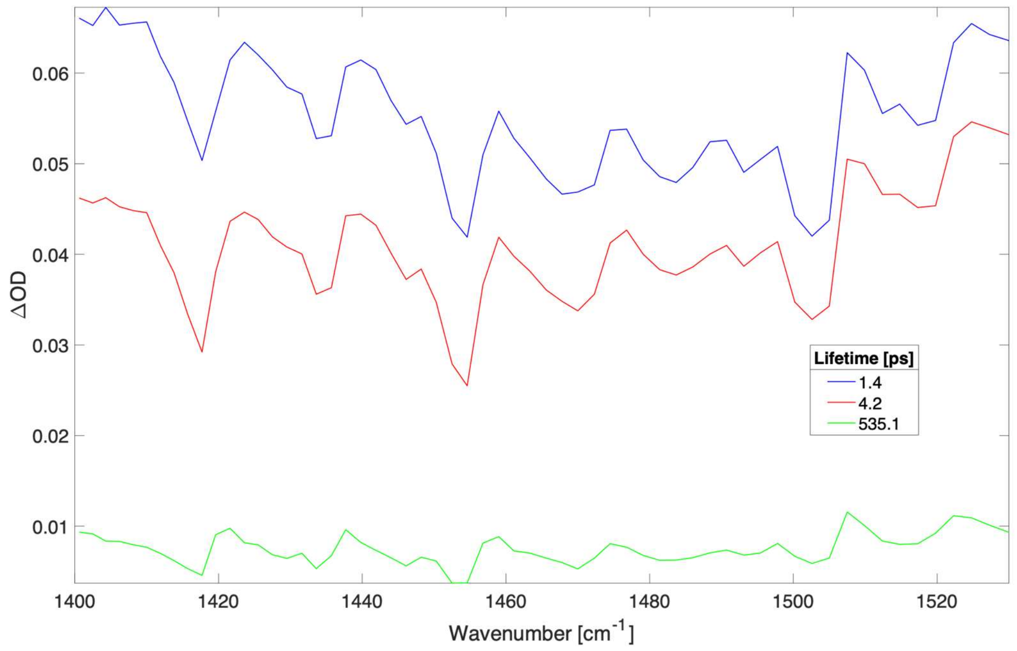

Figure S60.3. Time independent spectra of sequential three compartments global fit to spectra shown in Figure S60.1. The time constants (and contribution to data) were: 1.4 ps (52.1%), 4.2 ps (40.1%), and 535.1 ps (7.8%).

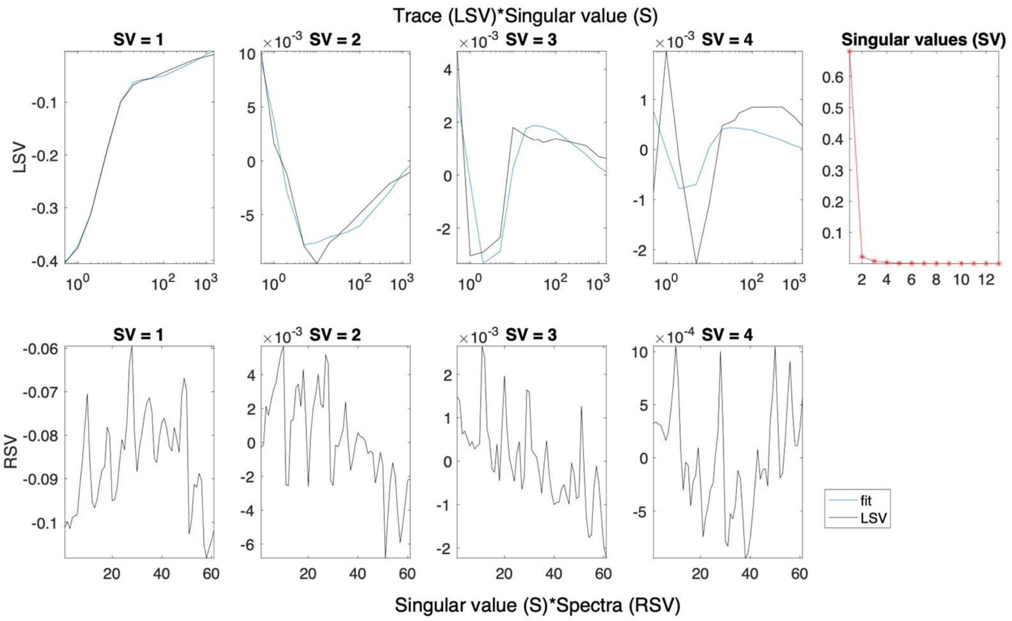

Figure S60.4. Left (LSV) and right (RSV) singular vectors with dominant singular values for the SVD analysis of spectra presented in Figure S60.1.

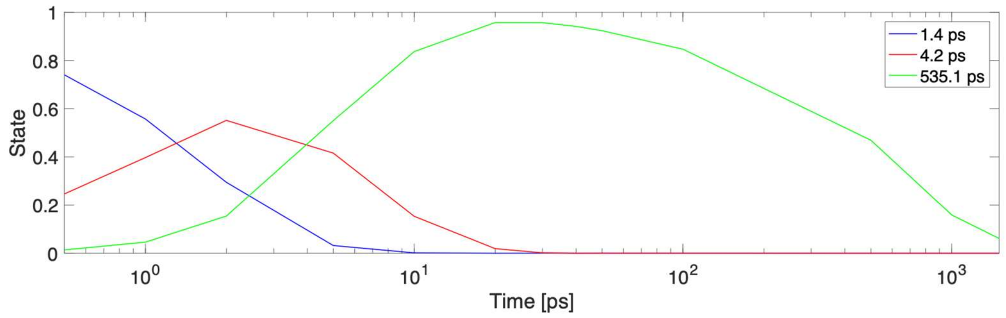

Figure S60.5. Concentration profiles for each time constant fitted to the data in Figure S60.3.

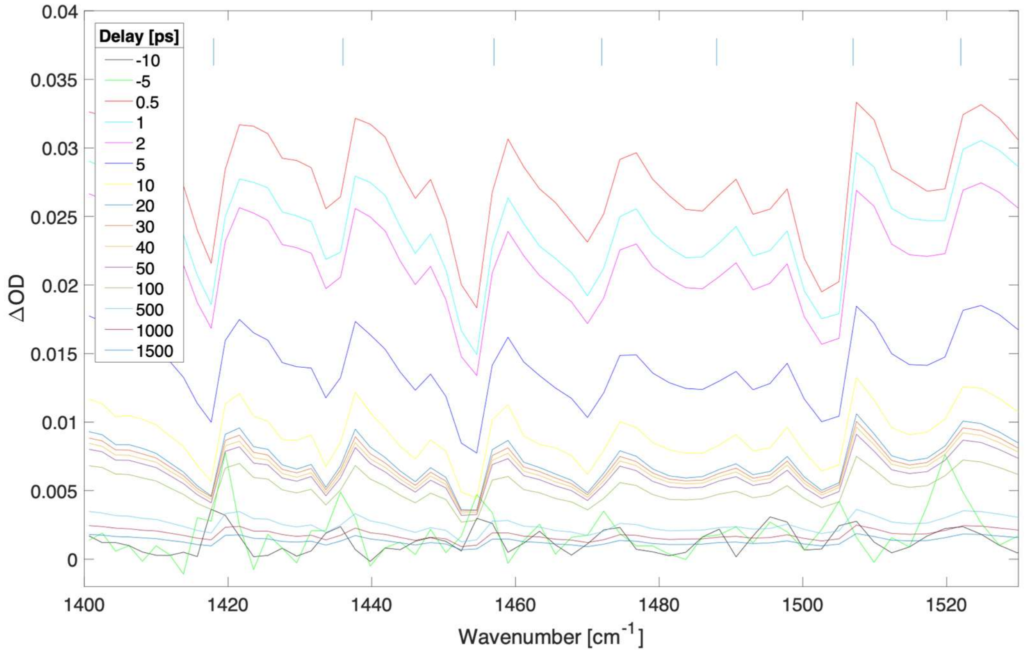

Figure S61.1. Difference transient absorption spectra at selected delays (ps) for a 300 nm thick film of $MAPbBr_3$ with pump irradiance of 7.10e9 W/cm^2 (fluence of 1,774.0 uJ/cm^2) at 539 nm.

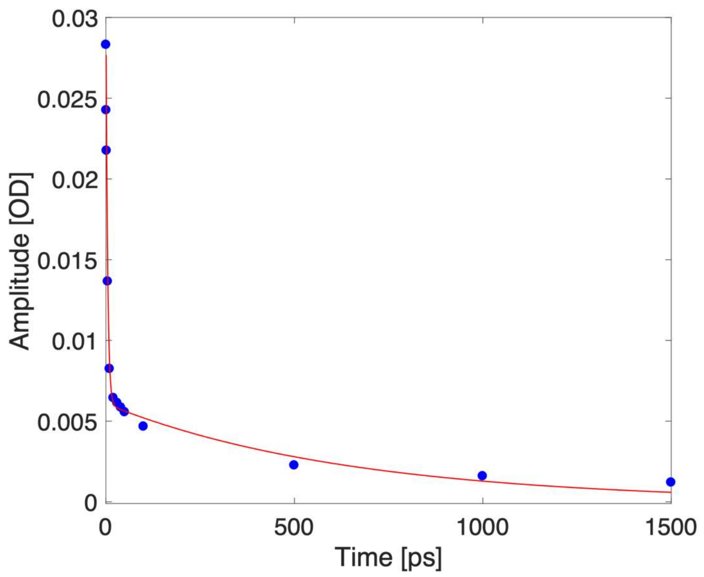

Figure S61.2. Kinetic trace taken at 1444 cm^{-1} (blue dots depict experimental data for selected delays, red curve represents a biexponential fit).

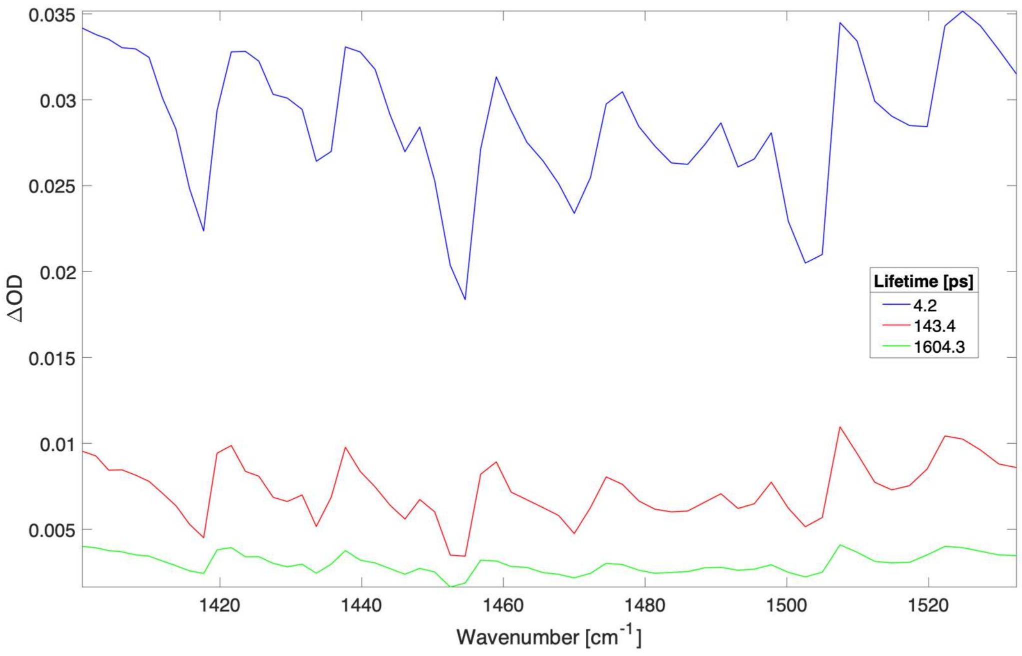

Figure S61.3. Time independent spectra of sequential three compartments global fit to spectra shown in Figure S61.1. The time constants (and contribution to data) were: 4.2 ps (72.4%), 143.3 ps (19.6%), and 1604.3 ps (8.0%).

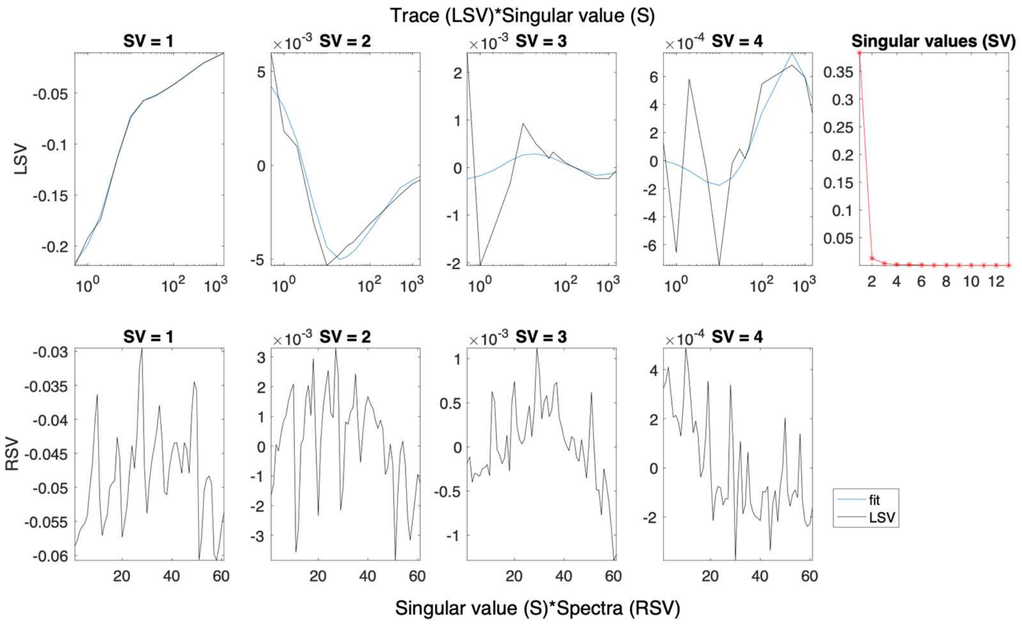

Figure S61.4. Left (LSV) and right (RSV) singular vectors with dominant singular values for the SVD analysis of spectra presented in Figure S61.1.

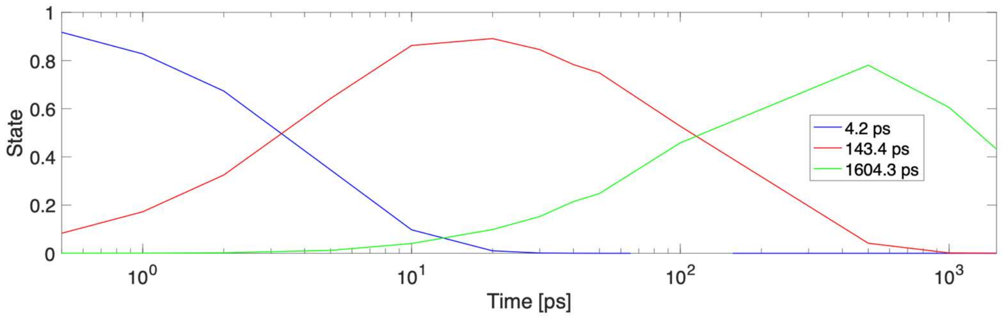

Figure S61.5. Concentration profiles for each time constant fitted to the data in Figure S61.3.

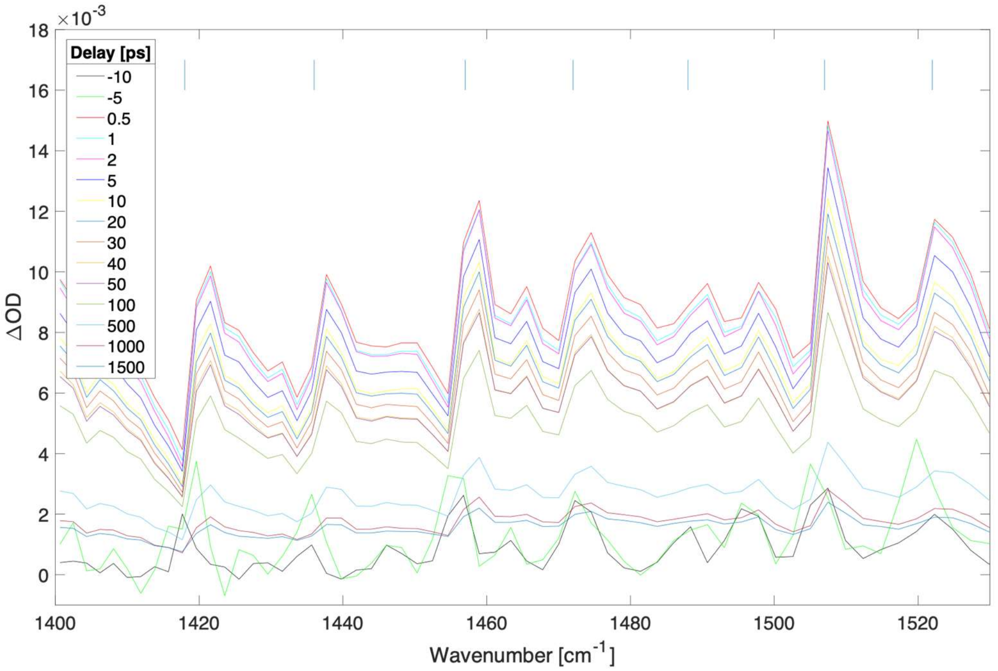

Figure S62.1. Difference transient absorption spectra at selected delays (ps) for a 200 nm thick film of $MAPbBr_3$ with pump irradiance of 2.83e9 W/cm^2 (fluence of 707.4 uJ/cm^2) at 539 nm.

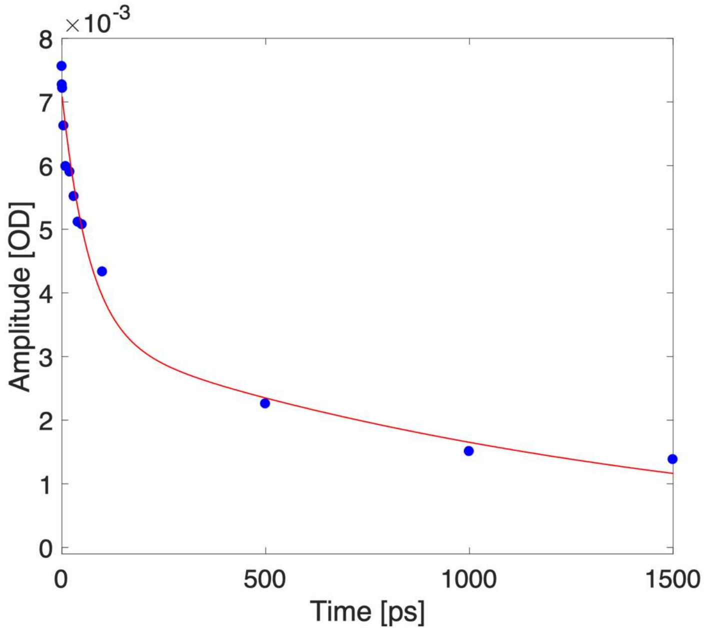

Figure S62.2. Kinetic trace taken at 1444 cm^{-1} (blue dots depict experimental data for selected delays, red curve represents a biexponential fit).

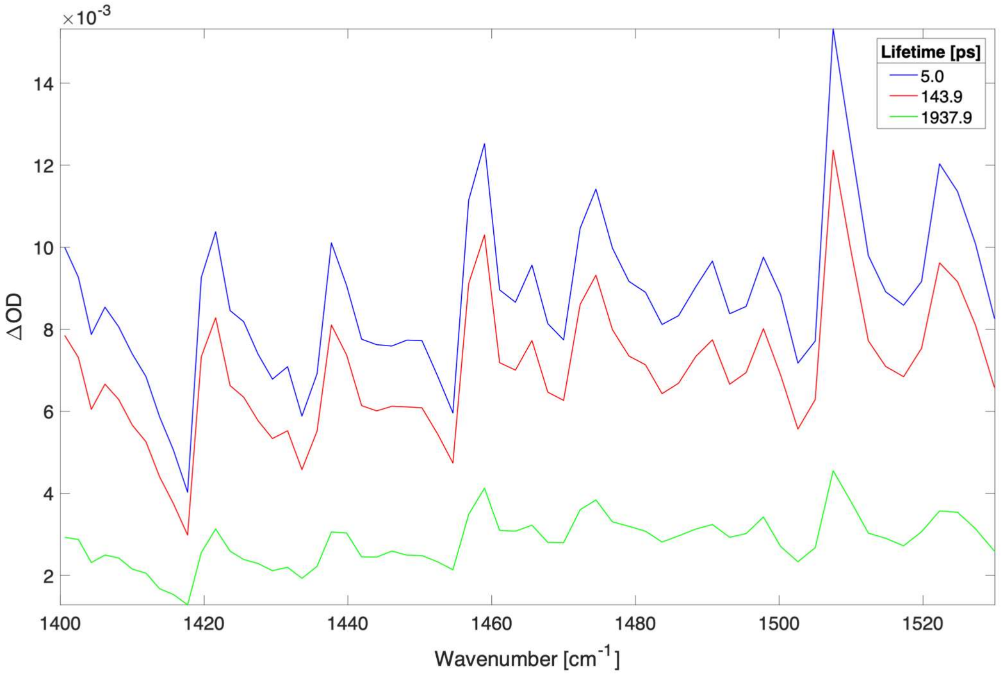

Figure S62.3. Time independent spectra of sequential three compartments global fit to spectra shown in Figure S62.1. The time constants (and contribution to data) were: 5.0 ps (46.8%), 143.9 ps (37.0%), and 1937.9 ps (16.2%).

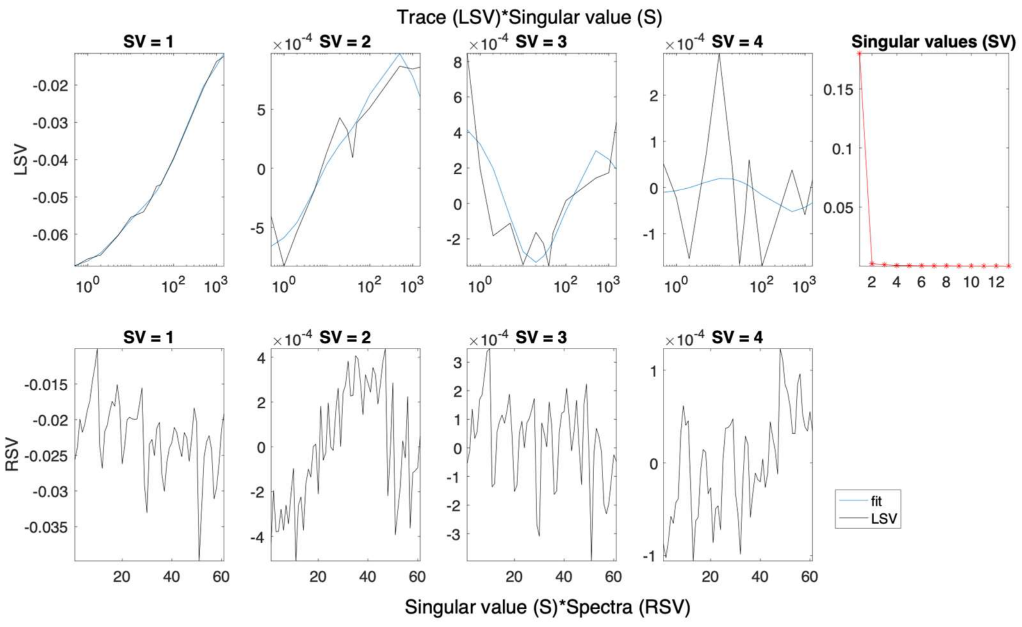

Figure S62.4. Left (LSV) and right (RSV) singular vectors with dominant singular values for the SVD analysis of spectra presented in Figure S62.1.

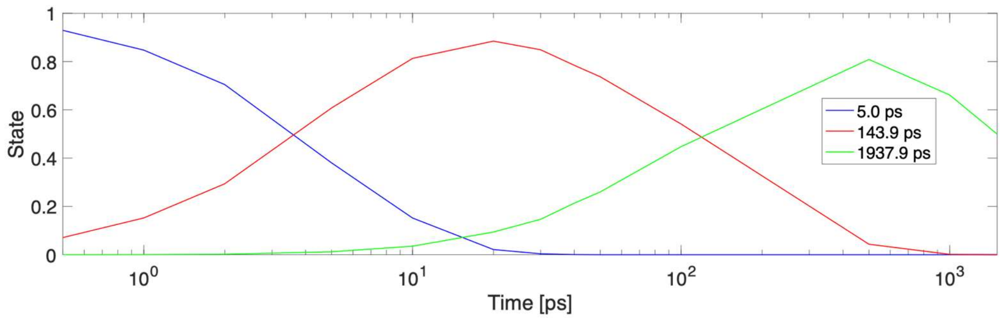

Figure S62.5. Concentration profiles for each time constant fitted to the data in Figure S62.3.

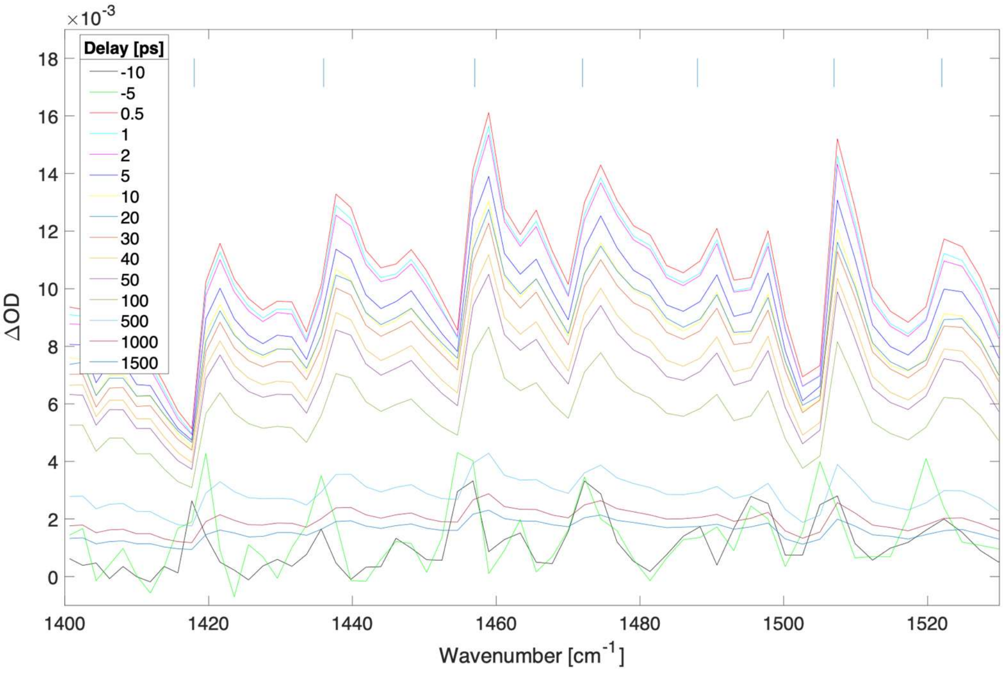

Figure S63.1. Difference transient absorption spectra at selected delays (ps) for a 200 nm thick film of $MAPbBr_3$ with pump irradiance of 2.32 e9 W/cm^2 (fluence of 580.0 uJ/cm^2) at 539 nm.

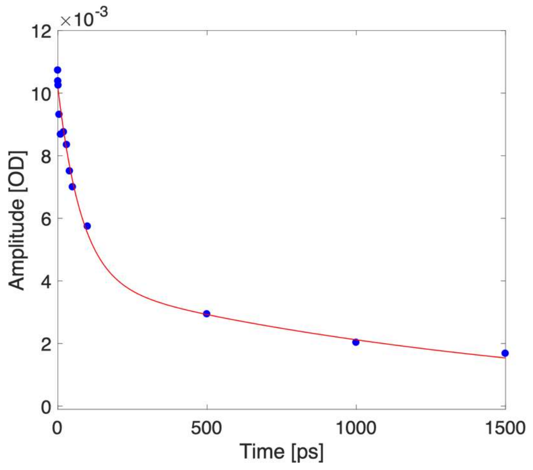

Figure S63.2. Kinetic trace taken at 1444 cm^{-1} (blue dots depict experimental data for selected delays, red curve represents a biexponential fit).

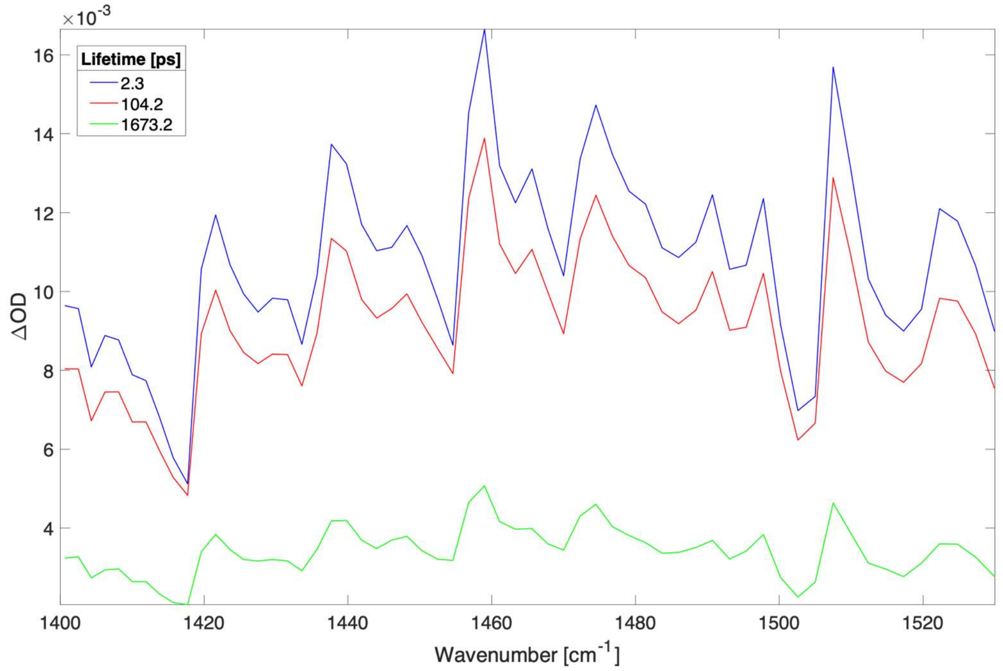

Figure S63.3. Time independent spectra of sequential three compartments global fit to spectra shown in Figure S63.1. The time constants (and contribution to data) were: 2.3 ps (46.0%), 104.2 ps (38.6%), and 1673.2 ps (15.4%).

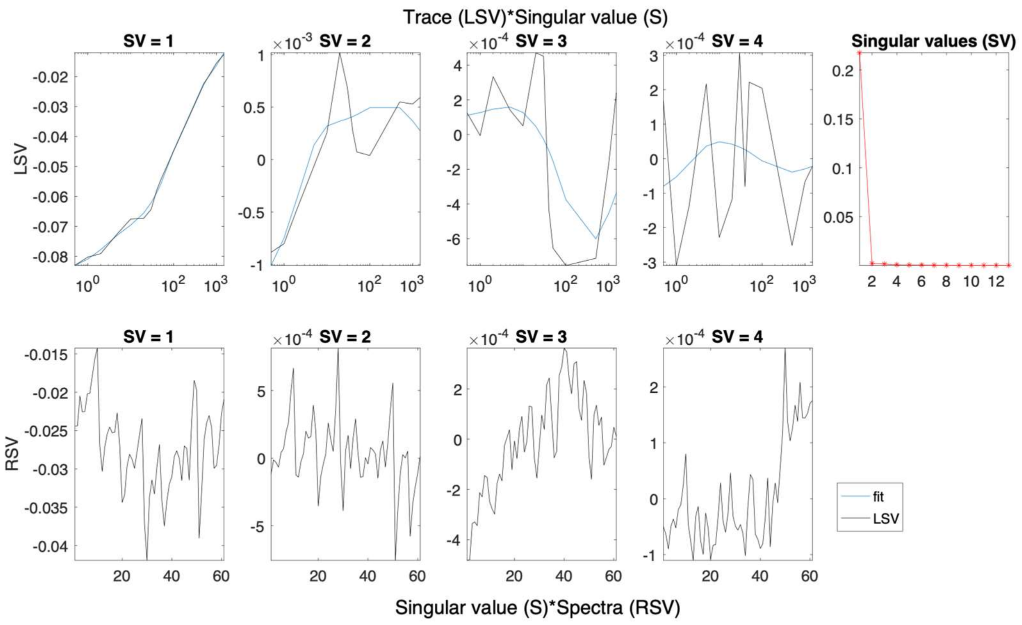

Figure S63.4. Left (LSV) and right (RSV) singular vectors with dominant singular values for the SVD analysis of spectra presented in Figure S63.1.

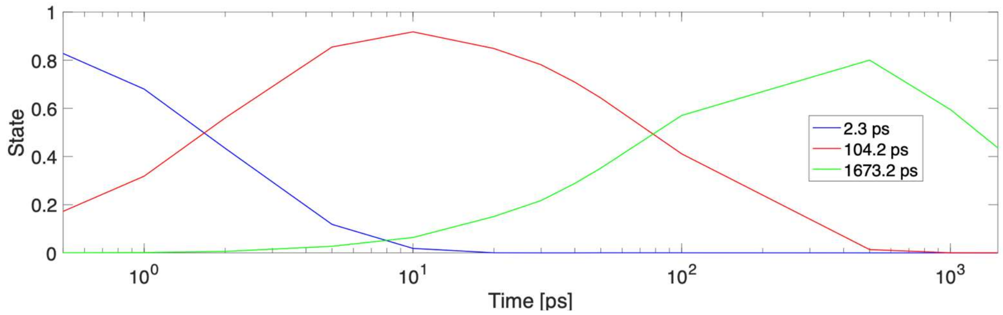

Figure S63.5. Concentration profiles for each time constant fitted to the data in Figure S63.3.

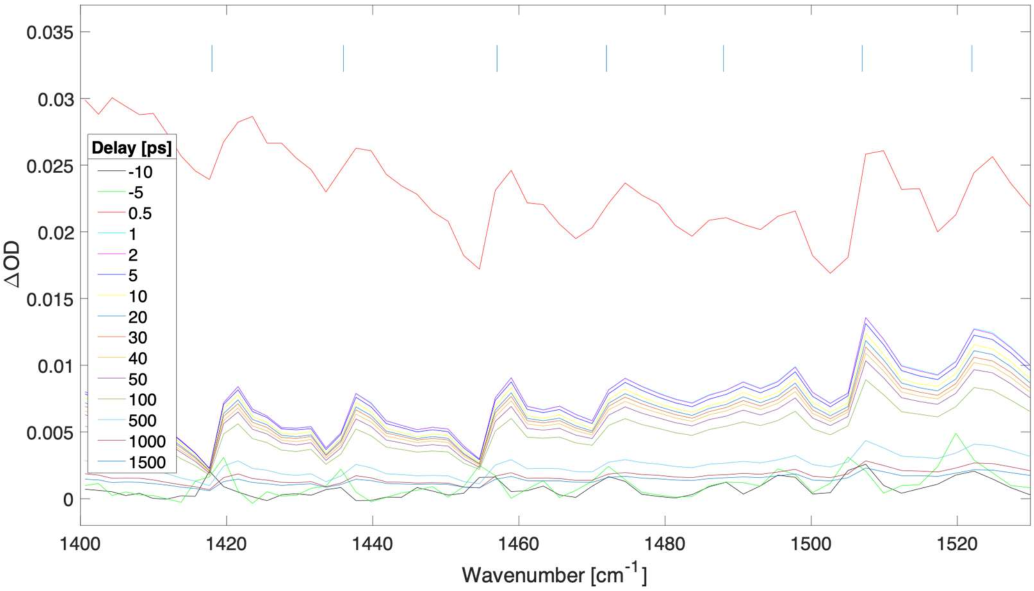

Figure S64.1. Difference transient absorption spectra at selected delays (ps) for a 200 nm thick film of $MAPbBr_3$ with pump irradiance of 1.86e9 W/cm^2 (fluence of 464.0 uJ/cm^2) at 539 nm.

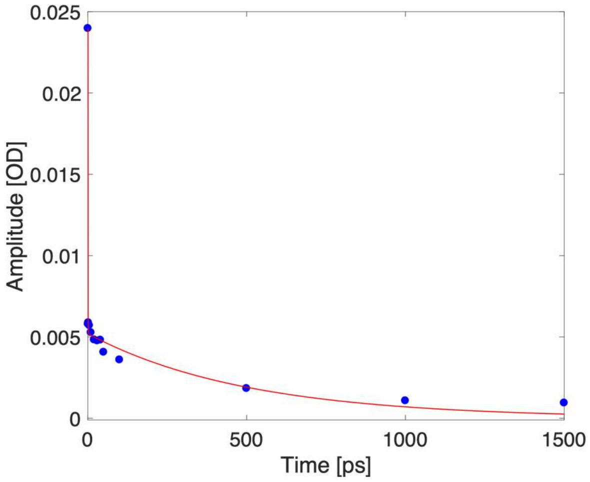

Figure S64.2. Kinetic trace taken at 1444 cm^{-1} (blue dots depict experimental data for selected delays, red curve represents a biexponential fit).

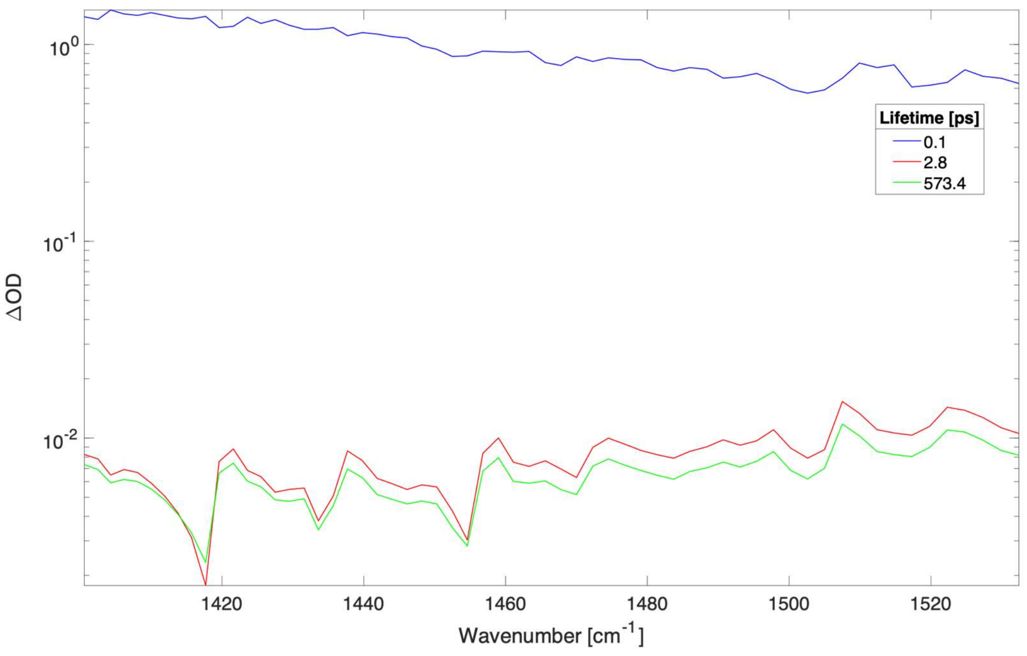

Figure S64.3. Time independent spectra of sequential three compartments global fit to spectra shown in Figure S64.1. The time constants (and contribution to data) were: 0.1 ps (99.0%), 2.8 ps (0.6%), and 573.4 ps (0.4%).

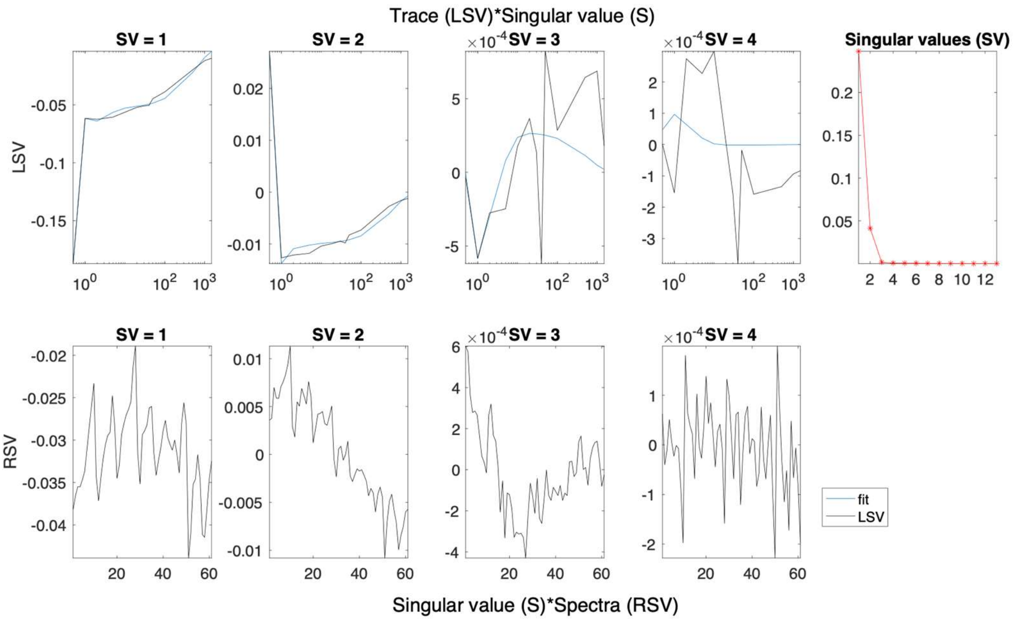

Figure S64.4. Left (LSV) and right (RSV) singular vectors with dominant singular values for the SVD analysis of spectra presented in Figure S64.1.

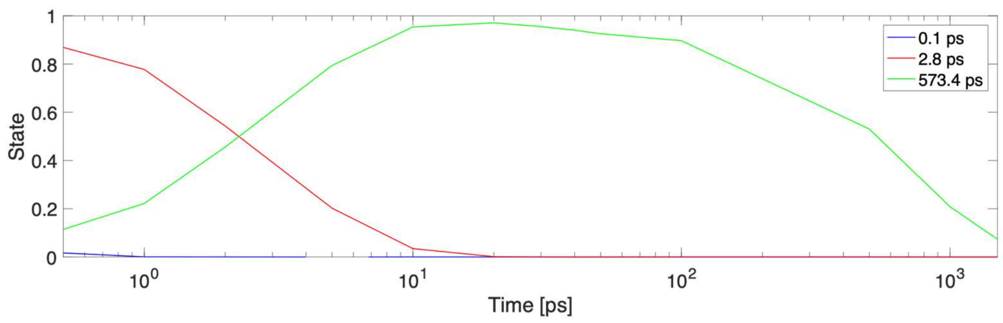

Figure S64.5. Concentration profiles for each time constant fitted to the data in Figure S64.3.

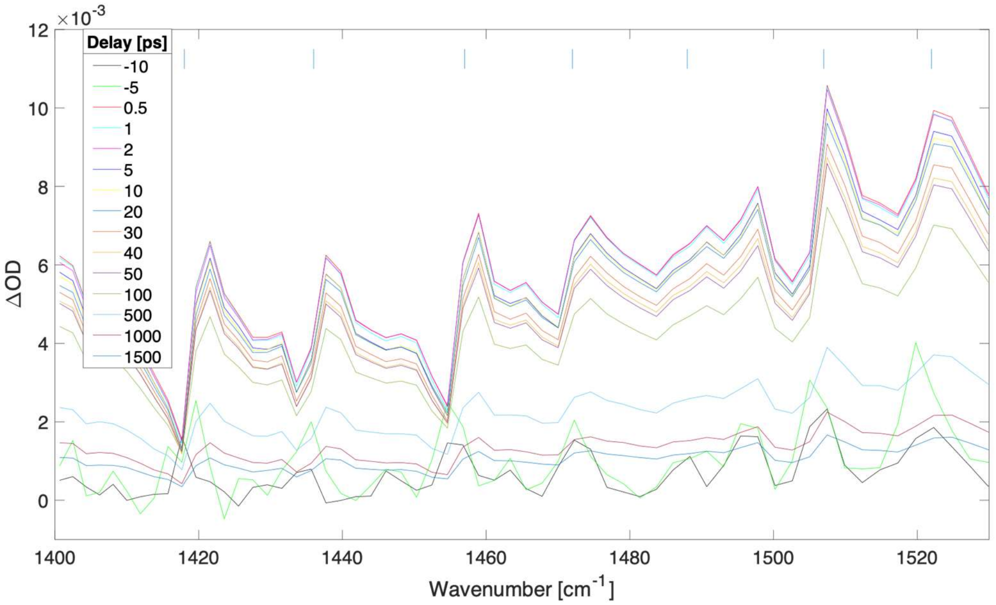

Figure S65.1. Difference transient absorption spectra at selected delays (ps) for a 200 nm thick film of $MAPbBr_3$ with pump irradiance of 1.47e9 W/cm^2 (fluence of 367.8 uJ/cm^2) at 539 nm.

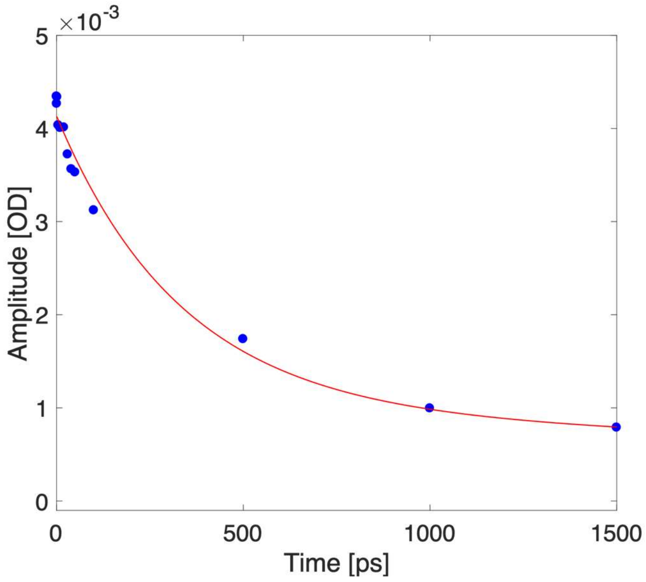

Figure S65.2. Kinetic trace taken at 1444 cm^{-1} (blue dots depict experimental data for selected delays, red curve represents a biexponential fit).

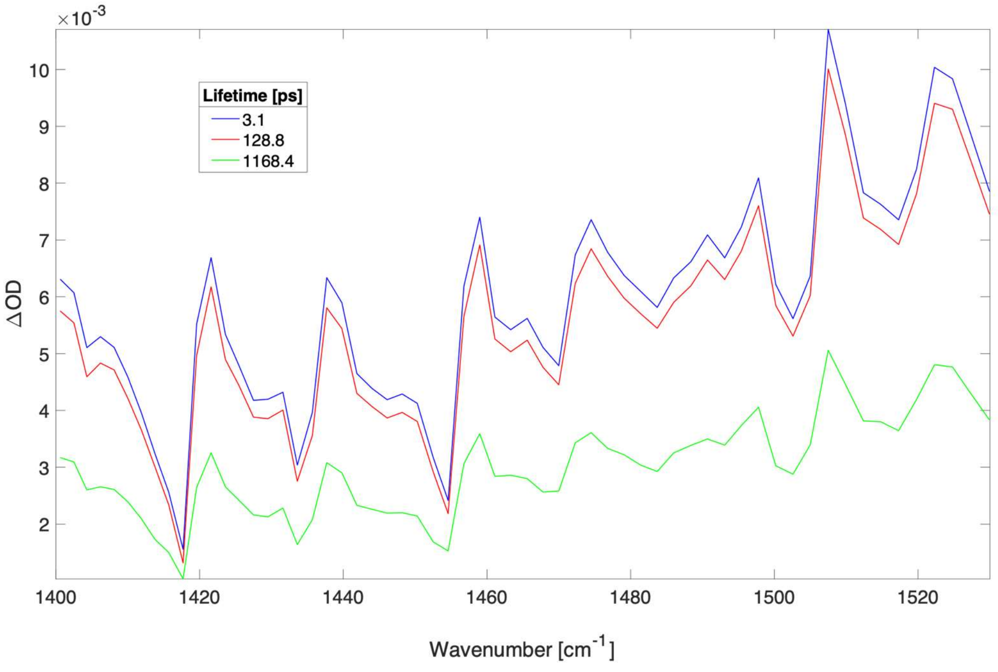

Figure S65.3. Time independent spectra of sequential three compartments global fit to spectra shown in Figure S66.1. The time constants (and contribution to data) were: 3.1 ps (40.6%), 128.8 ps (38.2%), and 1168.4 ps (21.2%).

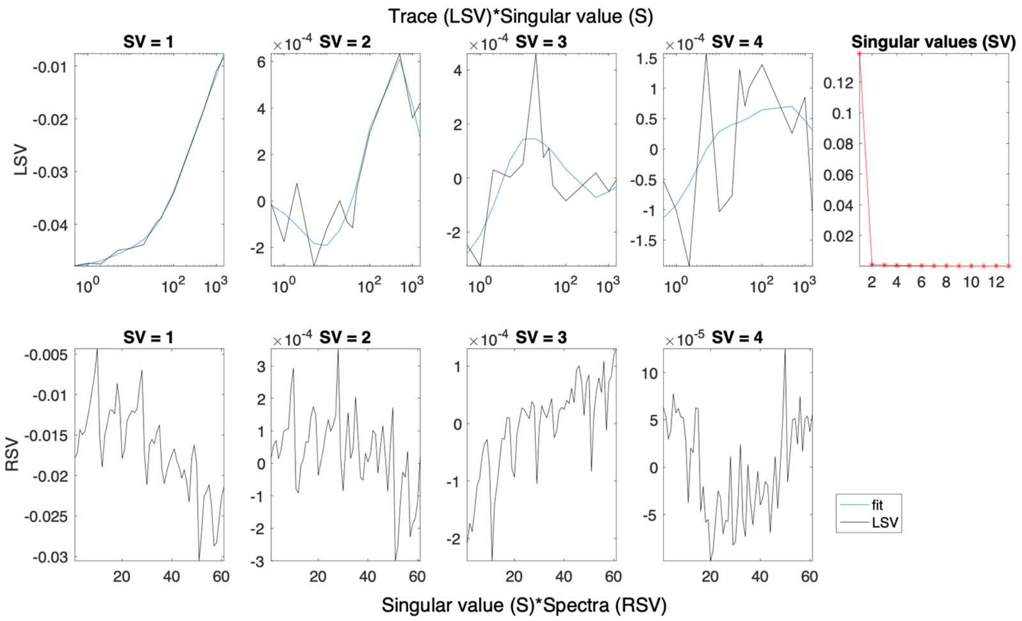

Figure S65.4. Left (LSV) and right (RSV) singular vectors with dominant singular values for the SVD analysis of spectra presented in Figure S66.1.

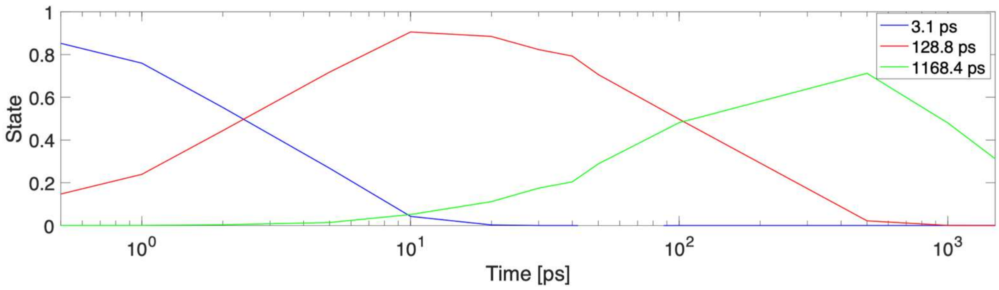

Figure S65.5. Concentration profiles for each time constant fitted to the data in Figure S66.3.

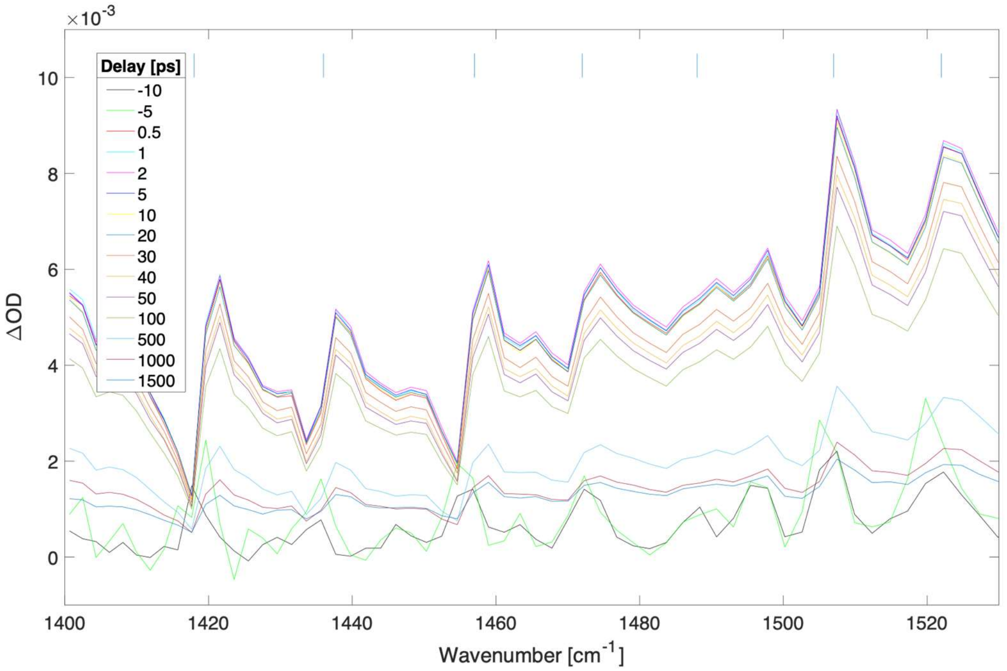

Figure S66.1. Difference transient absorption spectra at selected delays (ps) for a 200 nm thick film of $MAPbBr_3$ with pump irradiance of 1.18e9 W/cm^2 (fluence of 294.2 uJ/cm^2) at 539 nm.

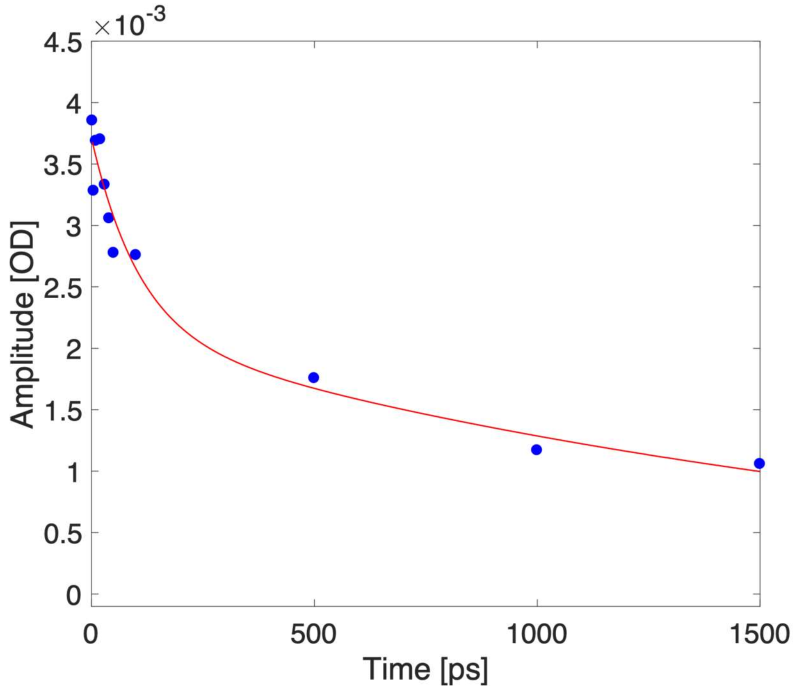

Figure S66.2. Kinetic trace taken at 1444 cm^{-1} (blue dots depict experimental data for selected delays, red curve represents a biexponential fit).

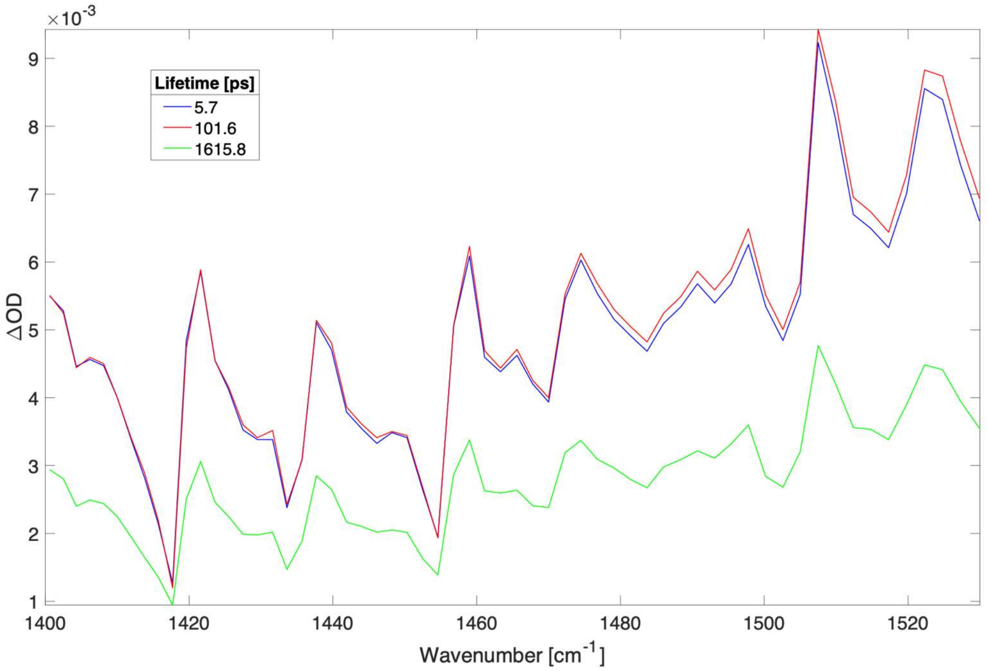

Figure S66.3. Time independent spectra of sequential three compartments global fit to spectra shown in Figure S66.1. The time constants (and contribution to data) were: 5.7 ps (38.1%), 101.6 ps (39.2%), and 1615.8 ps (22.7%).

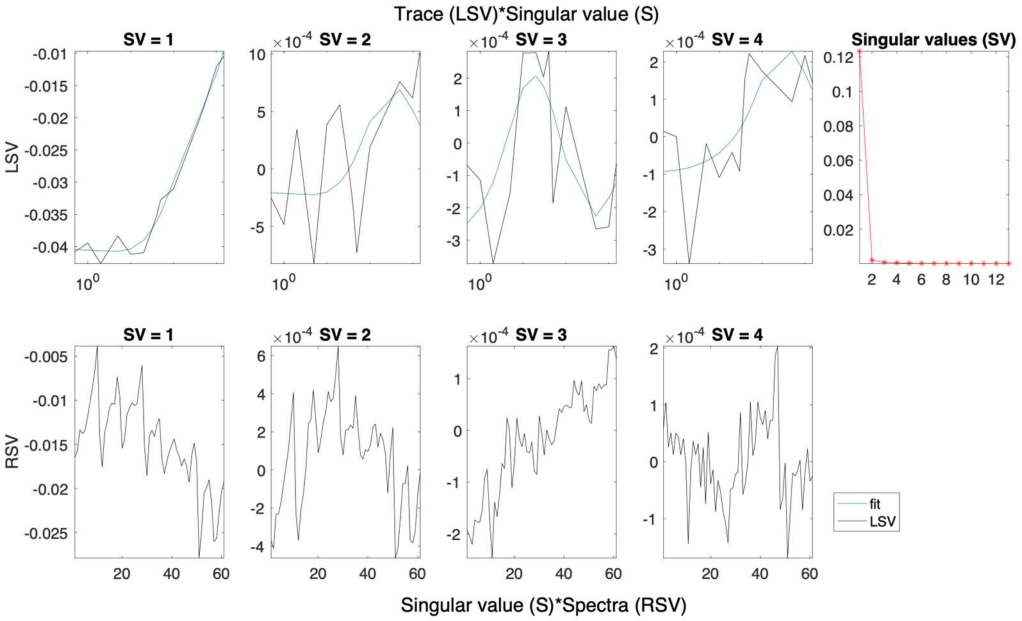

Figure S66.4. Left (LSV) and right (RSV) singular vectors with dominant singular values for the SVD analysis of spectra presented in Figure S66.1.

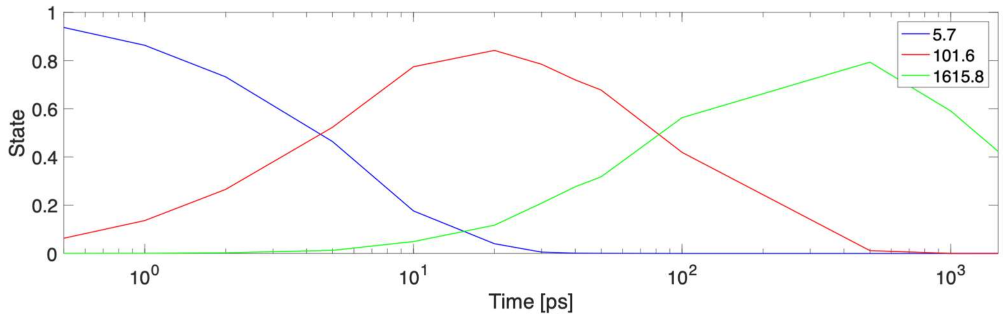

Figure S66.5. Concentration profiles for each time constant fitted to the data in Figure S66.3.

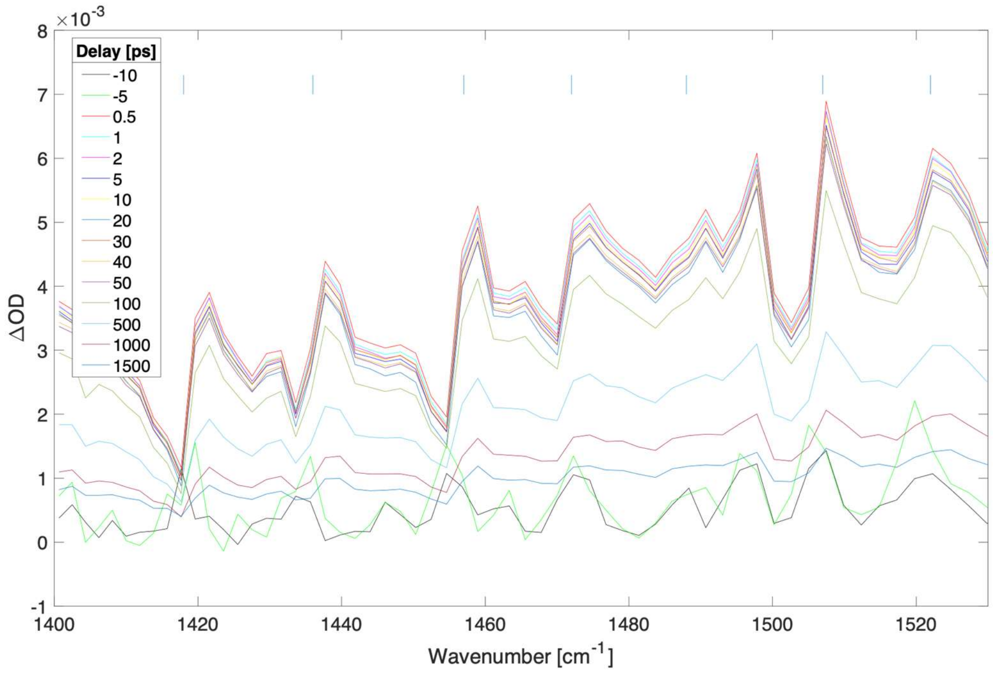

Figure S67.1. Difference transient absorption spectra at selected delays (ps) for a 200 nm thick film of $MAPbBr_3$ with pump irradiance of 9.05e8 W/cm^2 (fluence of 226.4 uJ/cm^2) at 539 nm.

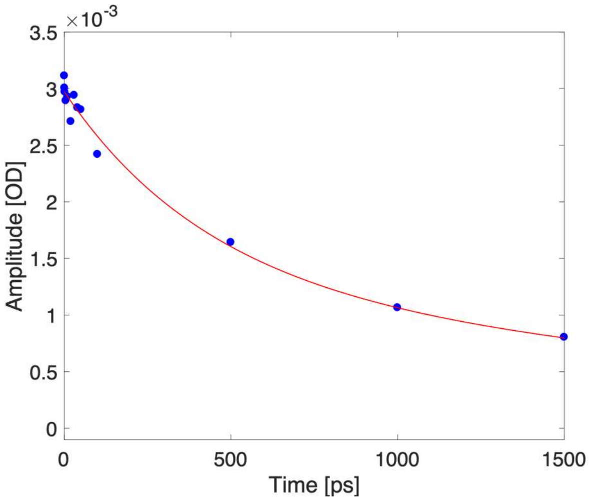

Figure S67.2. Kinetic trace taken at 1444 cm^{-1} (blue dots depict experimental data for selected delays, red curve represents a biexponential fit).

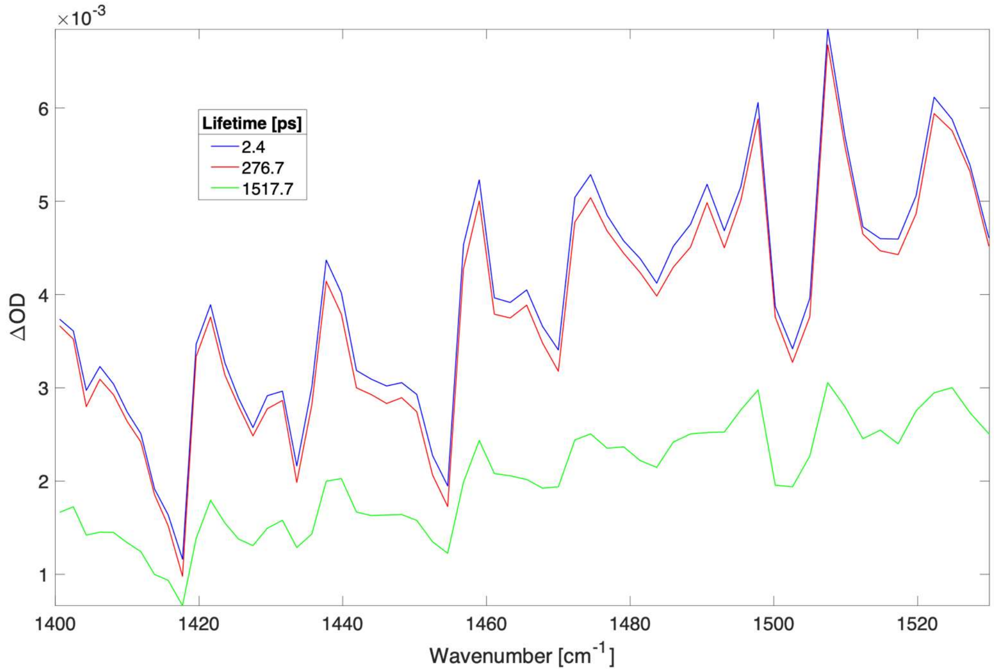

Figure S67.3. Time independent spectra of sequential three compartments global fit to spectra shown in Figure S67.1. The time constants (and contribution to data) were: 2.4 ps (39.8%), 276.7 ps (38.2%), and 1517.7 ps (22.0%).

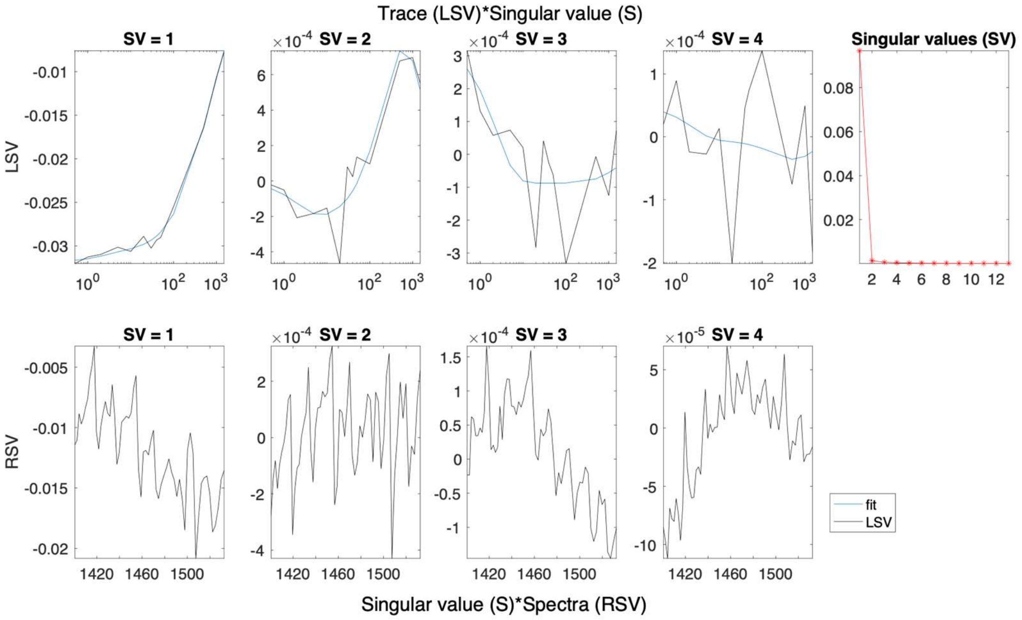

Figure S67.4. Left (LSV) and right (RSV) singular vectors with dominant singular values for the SVD analysis of spectra presented in Figure S67.1.

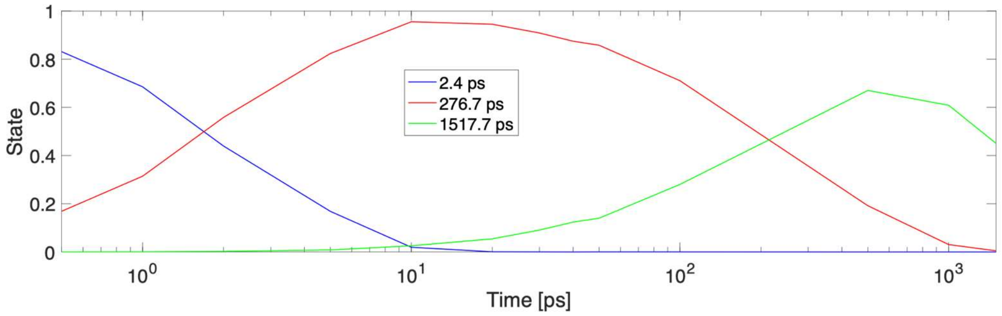

Figure S67.5. Concentration profiles for each time constant fitted to the data in Figure S67.3.

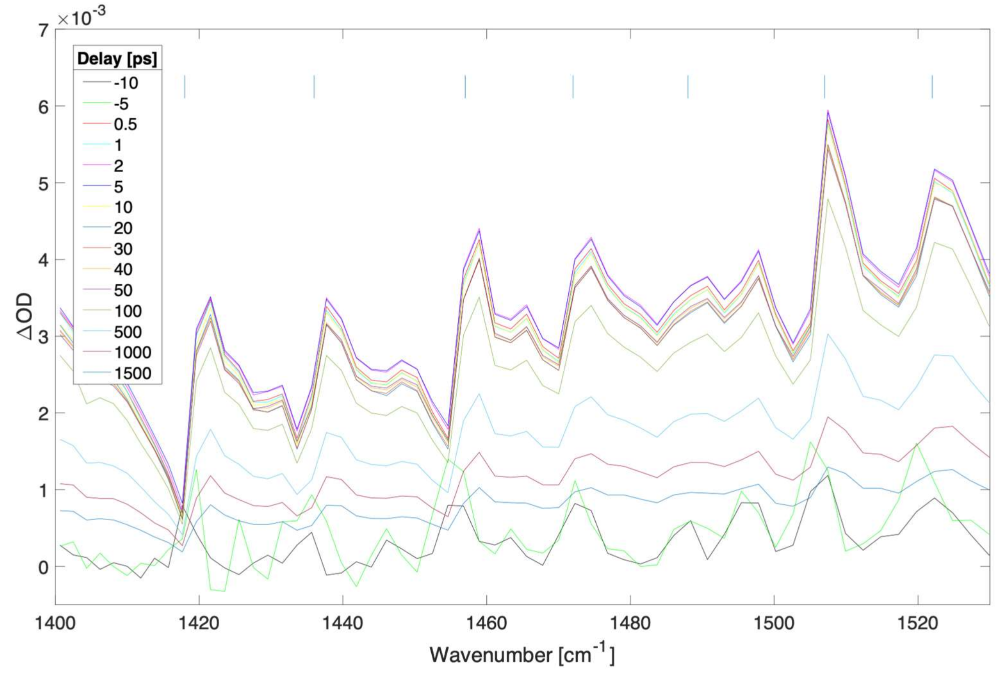

Figure S68.1. Difference transient absorption spectra at selected delays (ps) for a 200 nm thick film of $MAPbBr_3$ with pump irradiance of 2.94e8 W/cm^2 (fluence of 73.6 uJ/cm^2) at 539 nm.

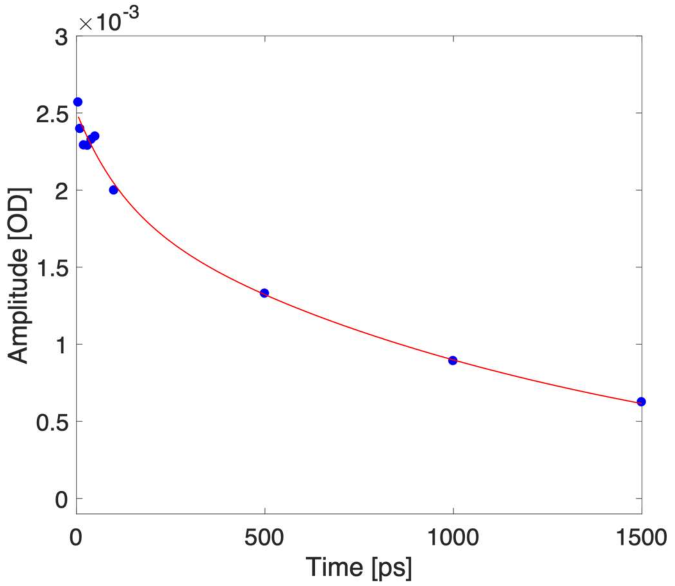

Figure S68.2. Kinetic trace taken at 1444 cm^{-1} (blue dots depict experimental data for selected delays, red curve represents a biexponential fit).

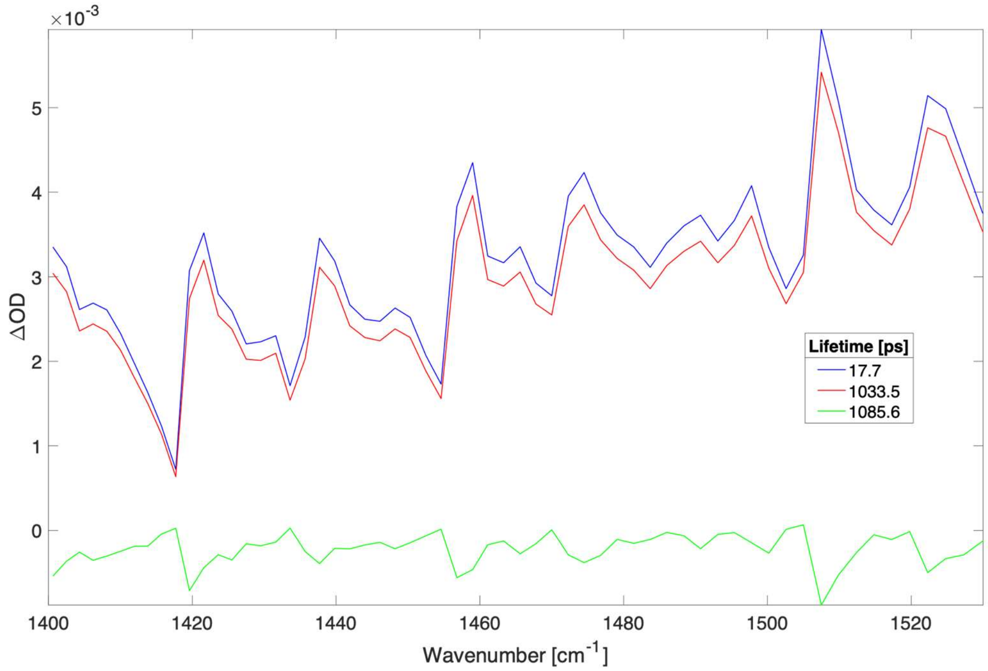

Figure S68.3. Time independent spectra of sequential three compartments global fit to spectra shown in Figure S68.1. The time constants (and contribution to data) were: 17.7 ps (50.4%), 1033.5 ps (46.3%), and 1085.6 ps (3.3%).

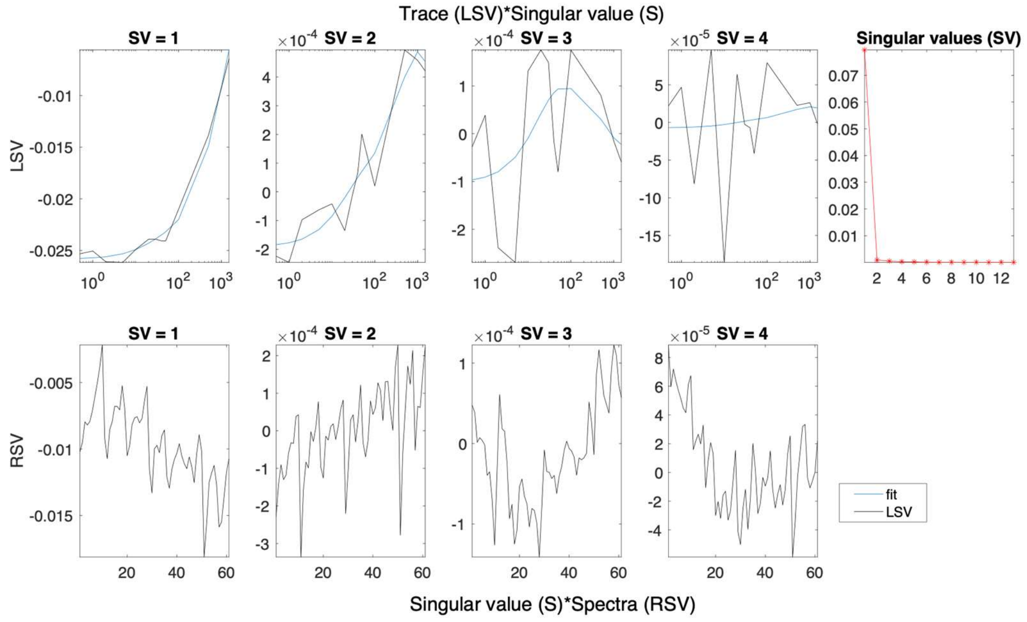

Figure S68.4. Left (LSV) and right (RSV) singular vectors with dominant singular values for the SVD analysis of spectra presented in Figure S68.1.

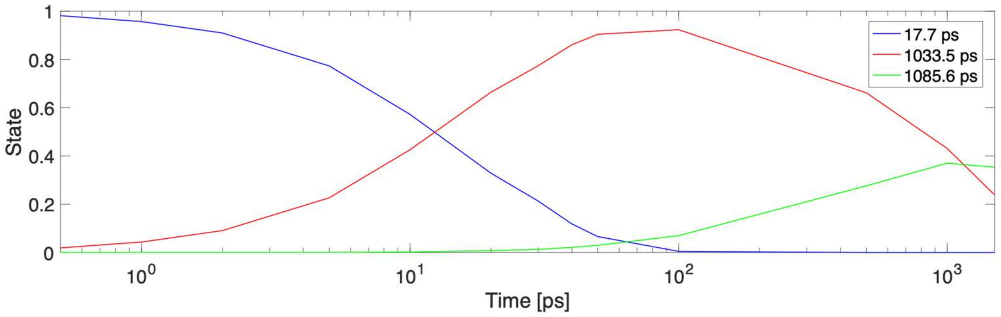

Figure S68.5. Concentration profiles for each time constant fitted to the data in Figure S68.3.

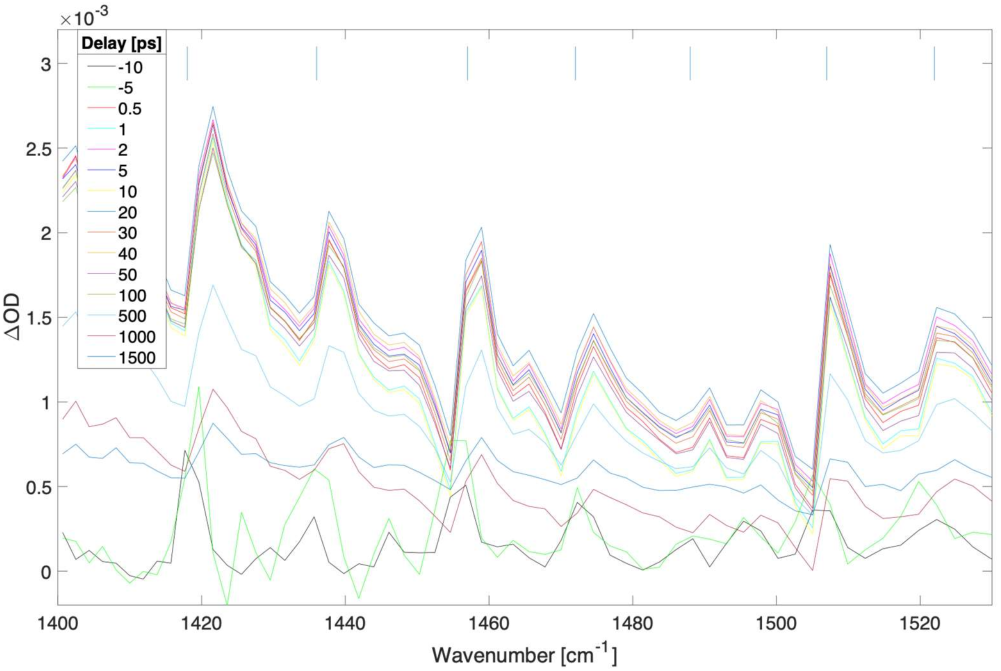

Figure S69.1. Difference transient absorption spectra at selected delays (ps) for a 200 nm thick film of $MAPbBr_3$ with pump irradiance of 1.47e8 W/cm^2 (fluence of 36.8 uJ/cm^2) at 539 nm.

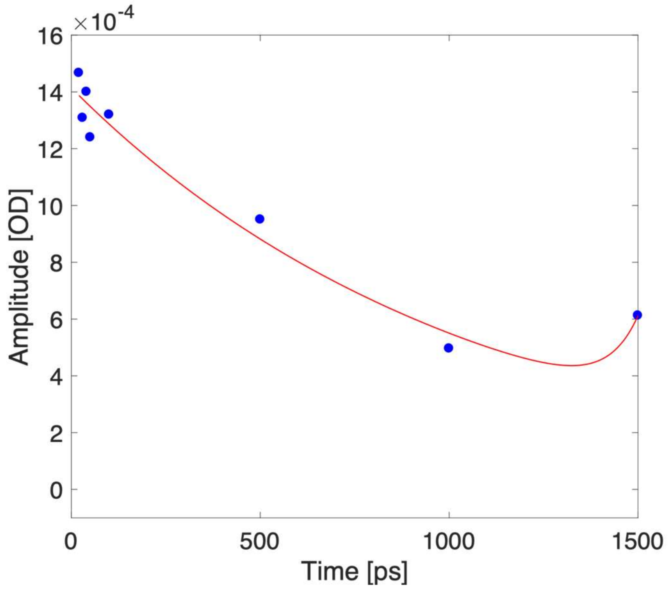

Figure S69.2. Kinetic trace taken at 1444 cm^{-1} (blue dots depict experimental data for selected delays, red curve represents a biexponential fit).

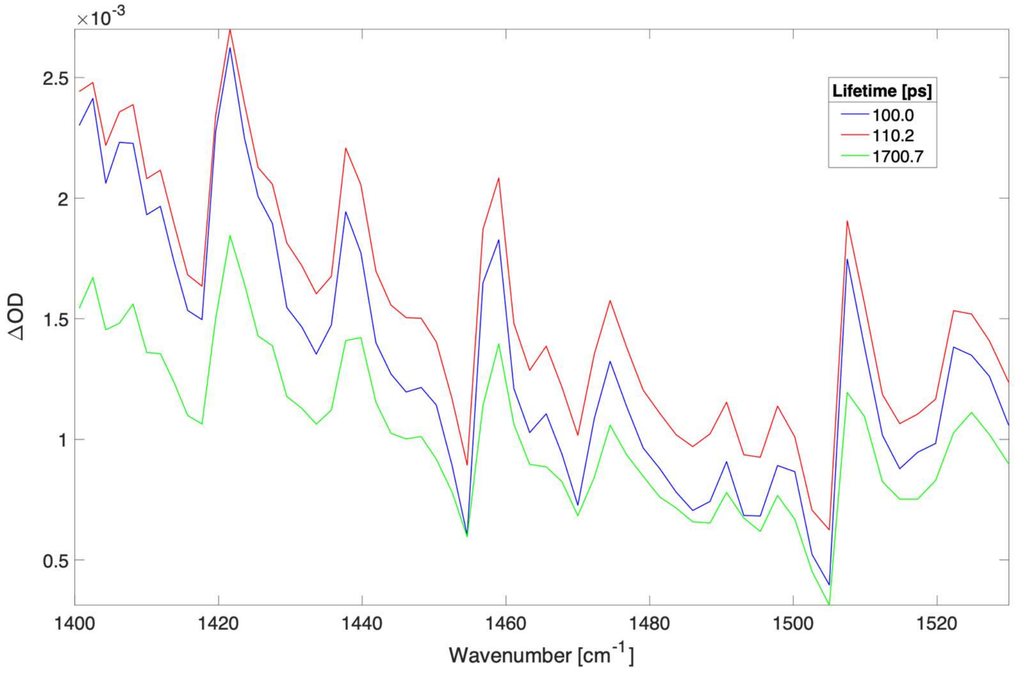

Figure S69.3. Time independent spectra of sequential three compartments global fit to spectra shown in Figure S69.1. The time constants (and contribution to data) were: 100.0 ps (33.4%), 110.2 ps (39.5%), and 1700.7 ps (27.1%).

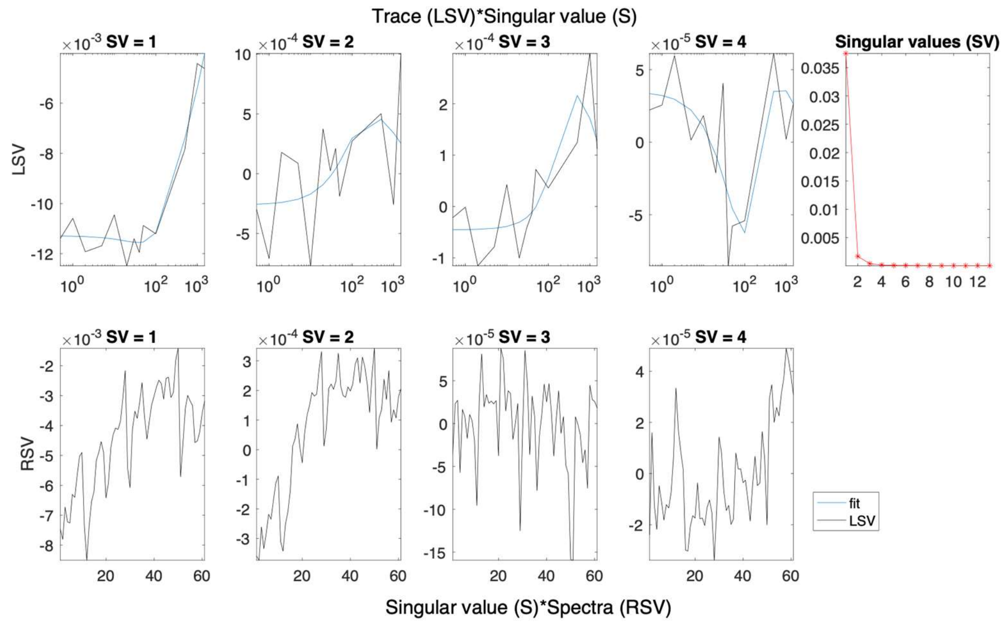

Figure S69.4. Left (LSV) and right (RSV) singular vectors with dominant singular values for the SVD analysis of spectra presented in Figure S69.4.

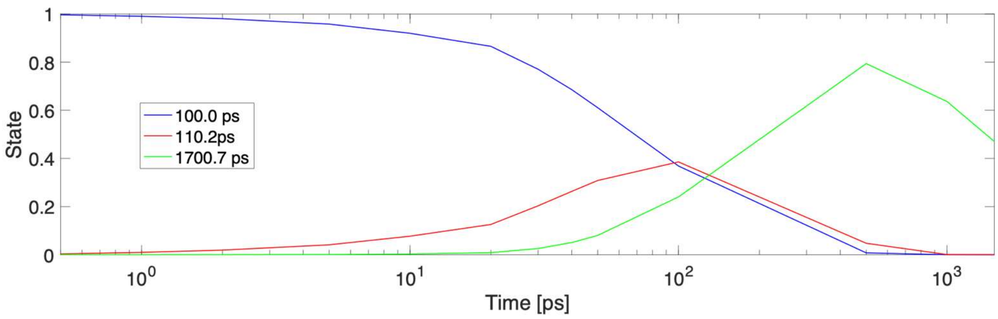

Figure S69.5. Concentration profiles for each time constant fitted to the data in Figure S69.3.

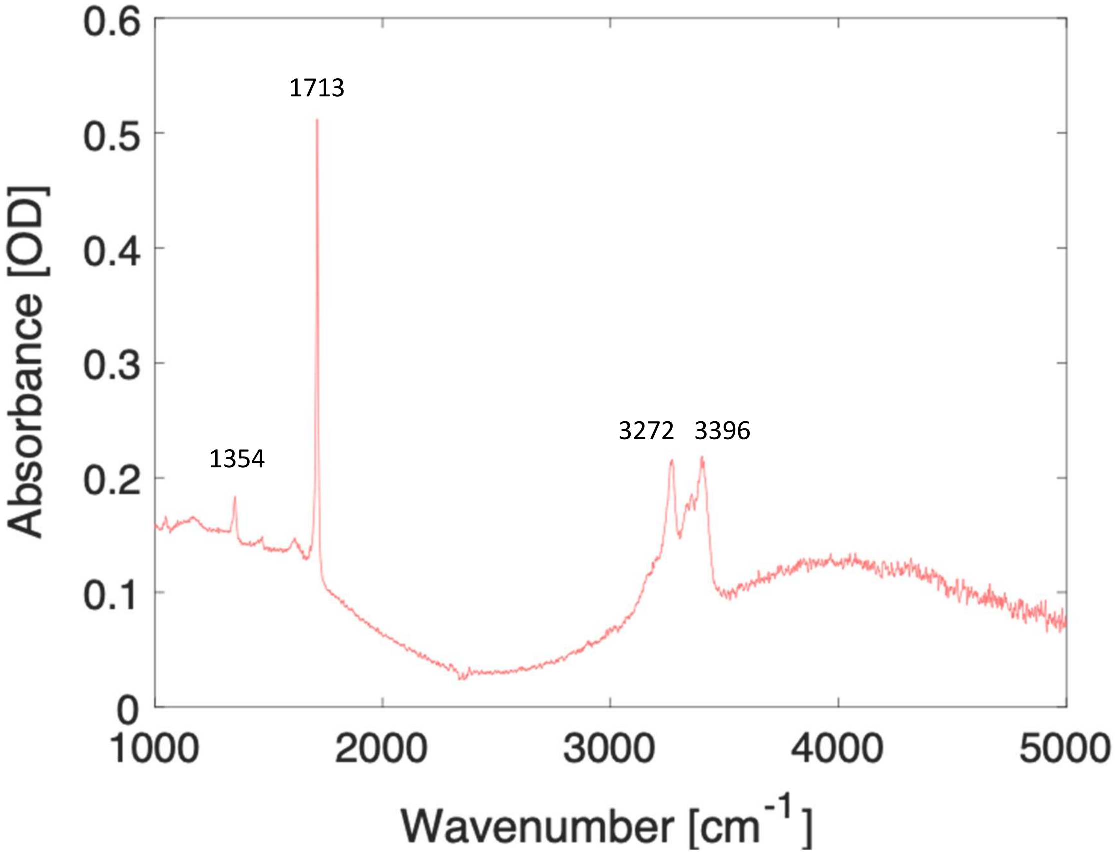

Figure S70. FTIR ground state absorption spectra of 600 nm film of $(FAPbI_3)_{0.97}(MAPbBr_3)_{0.03}$.

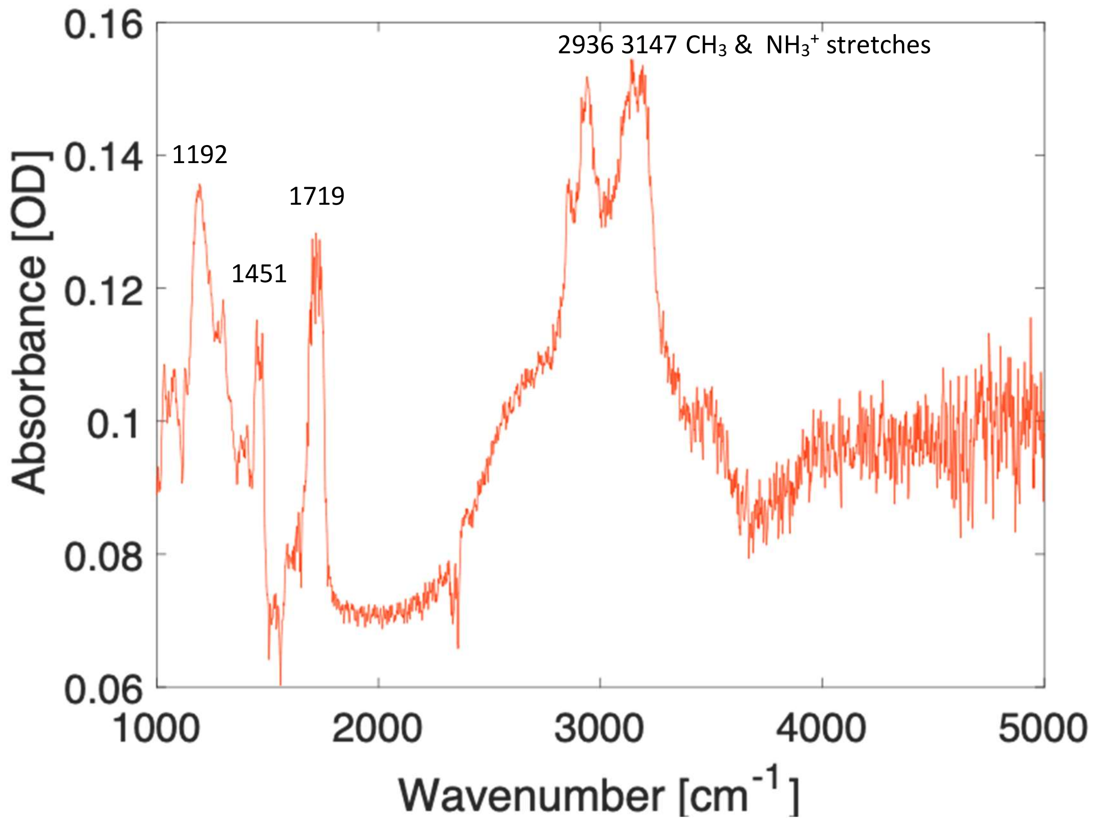

Figure S71. FTIR ground state absorption spectra of 500 nm film of $MAPbBr_3$.

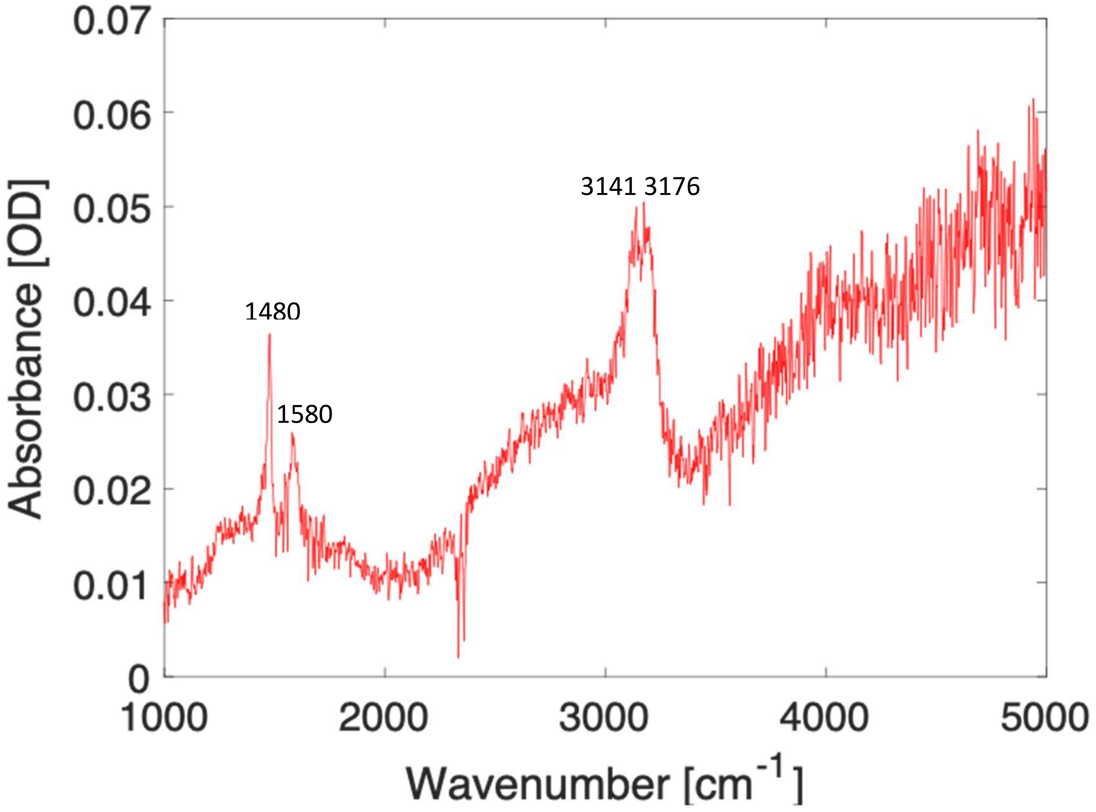

Figure S72. FTIR ground state absorption spectra of 200 nm film of $MAPbBr_3$.

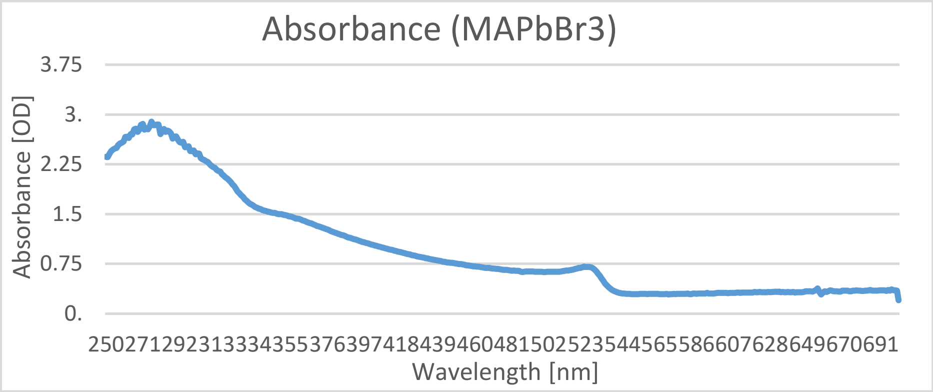

Figure S73. Visible ground state absorption spectra of 200 nm film of $MAPbBr_3$.

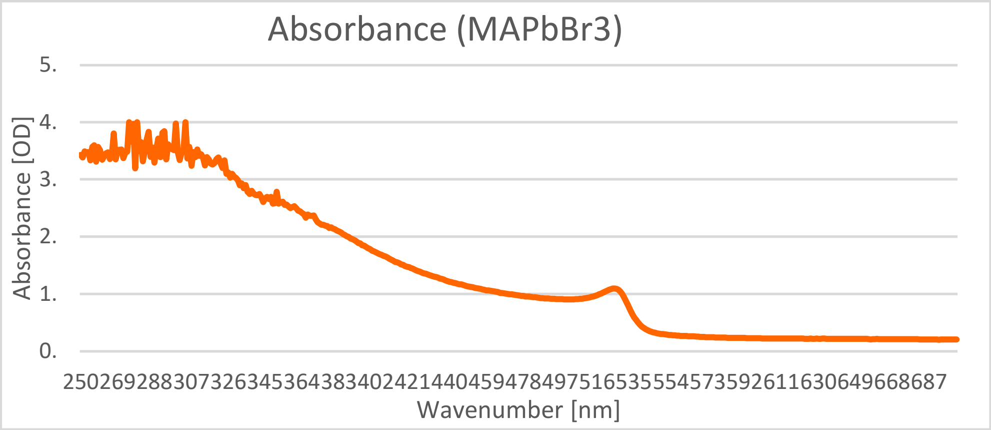

Figure S74. Visible ground state absorption spectra of 500 nm film of $MAPbBr_3$.

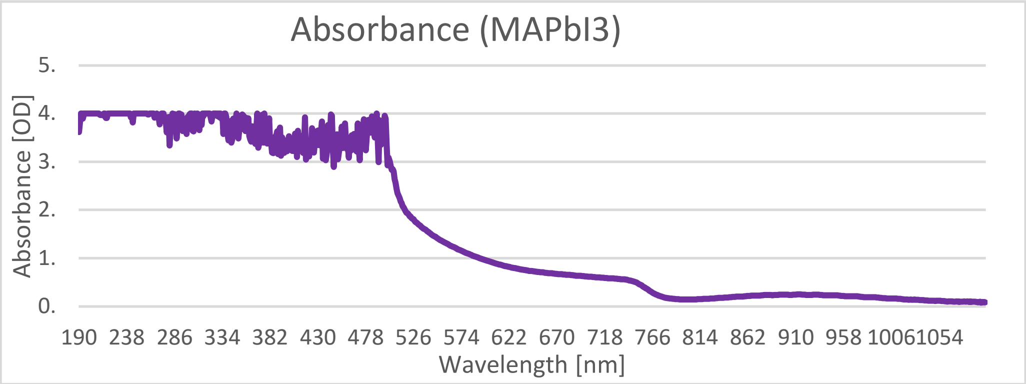

Figure S75. Visible ground state absorption spectra of 300 nm film of $MAPbI_3$.

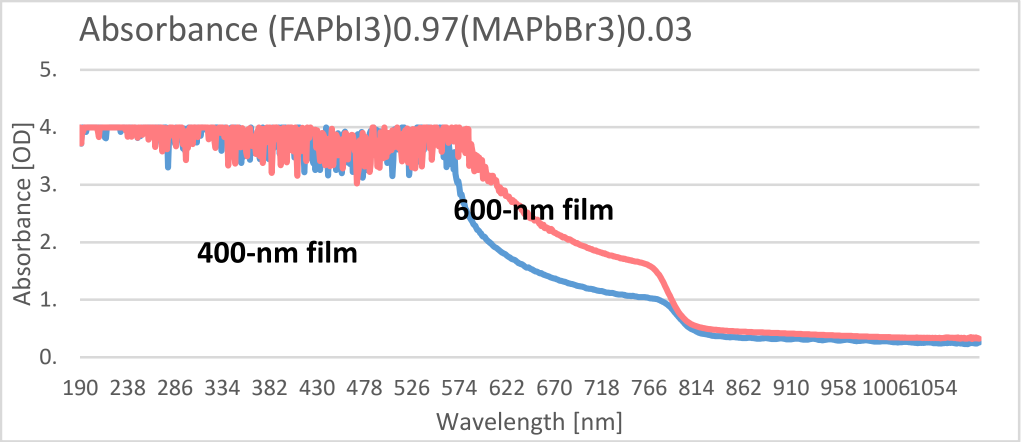

Figure S76. Visible ground state absorption spectra of 400 nm and 600 nm film of $(FAPbI_3)_{0.97}(MAPbBr_3)_{0.03}$.

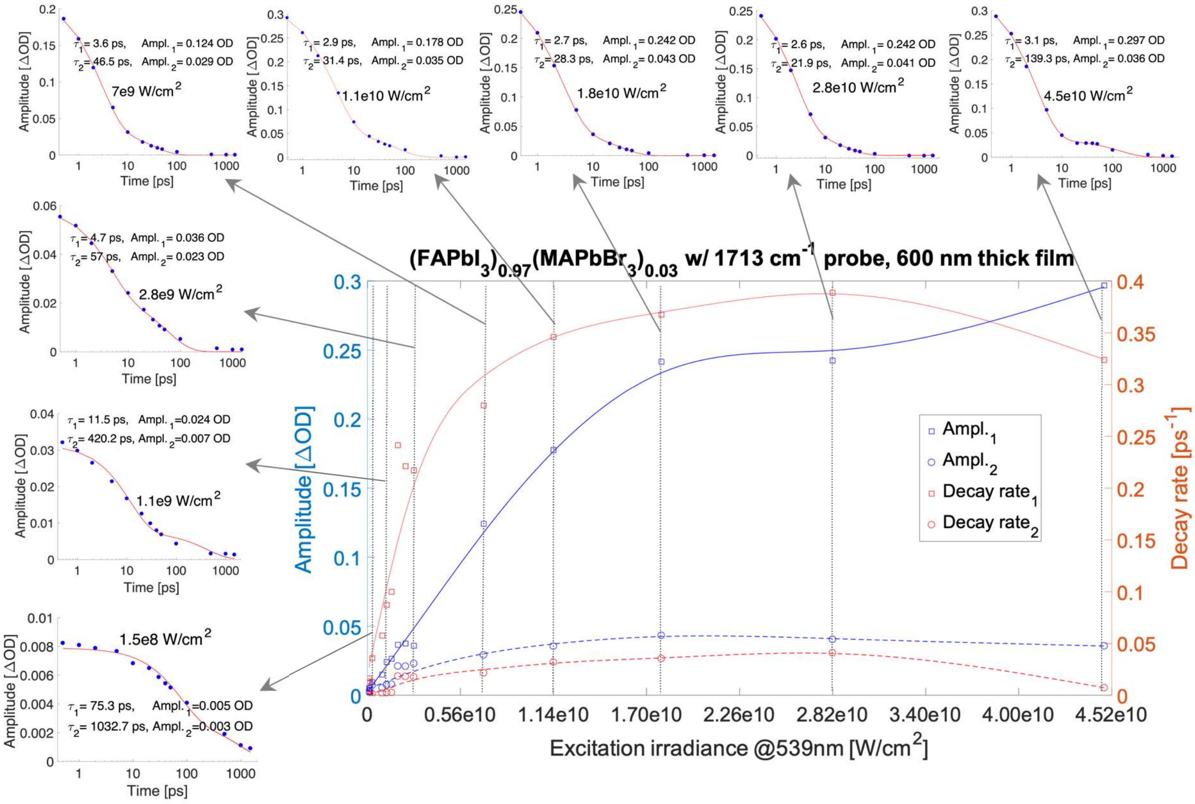

Figure S77. ΔOD amplitudes and decay rates of bi-exponential fitting to difference absorption spectra with a resonant 539 nm pump for $(FAPbI_3)_{0.97}(MAPbBr_3)_{0.03}$ with 1713 cm^{-1} probe – 600 nm thick film, insets are kinetic traces taken at 1713 cm^{-1} for selected irradiances.
